# Nickel-catalyzed trifluoromethylthiolation of Csp^2^–O bonds†
†Electronic supplementary information (ESI) available: Experimental procedures, spectroscopic data, ReactIR studies, computational information and Cartesian coordinates of calculated species as well as full ref. 17 are given. See DOI: 10.1039/c5sc03359d



**DOI:** 10.1039/c5sc03359d

**Published:** 2015-10-30

**Authors:** Alexander B. Dürr, Guoyin Yin, Indrek Kalvet, François Napoly, Franziska Schoenebeck

**Affiliations:** a RWTH Aachen University , Institute of Organic Chemistry , Landoltweg 1 , 52074 Aachen , Germany . Email: franziska.schoenebeck@rwth-Aachen.de

## Abstract

While nickel catalysts have previously been shown to activate even the least reactive Csp^2^–O bonds, *i.e.* aryl ethers, in the context of C–C bond formation, little is known about the reactivity limits and molecular requirements for the introduction of valuable functional groups under homogeneous nickel catalysis. We identified that due to the high reactivity of Ni-catalysts, they are also prone to react with existing or installed functional groups, which ultimately causes catalyst deactivation. The scope of the Ni-catalyzed coupling protocol will therefore be dictated by the reactivity of the functional groups towards the catalyst. Herein, we showed that the application of computational tools allowed the identification of matching functional groups in terms of suitable leaving groups and tolerated functional groups. This allowed for the development of the first efficient protocol to trifluoromethylthiolate Csp^2^–O bonds, giving the mild and operationally simple C–SCF_3_ coupling of a range of aryl, vinyl triflates and nonaflates. The novel methodology was also applied to biologically active and pharmaceutical relevant targets, showcasing its robustness and wide applicability.

## Abstract 2

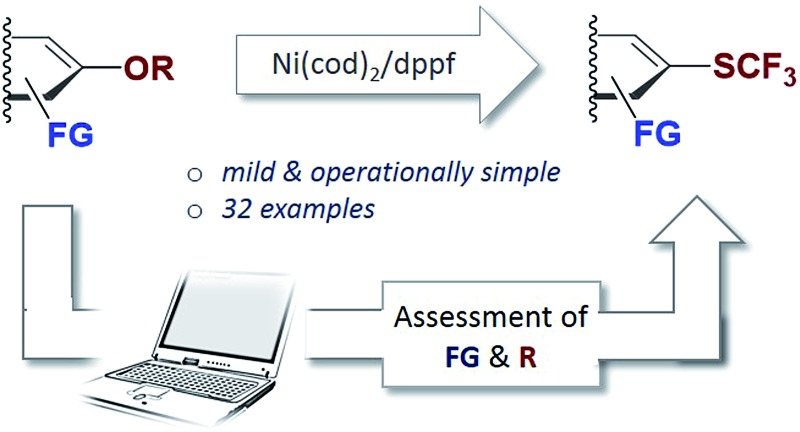
A computationally guided development of the first efficient protocol to trifluoromethylthiolate aryl and vinyl Csp^2^–O bonds is presented, showcasing important reactivity requirements for the introduction of potentially reactive functional groups under homogeneous Ni-catalysis.

## Introduction

Owing to nickel's non-precious nature and its higher reactivity in the first elementary step of cross coupling cycles, *i.e.* the oxidative addition, the field of homogeneous Ni-catalysis has long been considered promising, yet also challenging.^1^ This is because difficulties have frequently been encountered in taming nickel's reactive nature to achieve desired selectivities and scope.^2^ In spite of that, in recent years there has been impressive progress in the activation of the least reactive bonds, such as aromatic ethers or aryl fluorides.^3^ However, these milestones typically featured the conversion of C–OMe (or C–F^4^) to inert C–C or C–H bonds.^5,6^

By contrast, less is known about the reactivity limits and molecular requirements for the installation of *potentially reactive functional groups*. We therefore envisioned that a computationally assisted development^7^ of an unprecedented Ni-catalyzed protocol for C-heteroatom bond formation presents an ideal challenge to (i) identify the general reactivity requirements for efficient Ni-catalysis and (ii) demonstrate the viability of applying computational tools to assess substrate scope.

As a suitable test case, we focused on the nickel-catalyzed trifluoromethylthiolation of Csp^2^–O bonds.^8^

The SCF_3_ group makes molecules more lipophilic, increasing their membrane permeability and bioavailability.^9^ These properties are of considerable interest in a pharmaceutical and agrochemical context. Consequently, numerous efforts have been undertaken to synthesize aryltrifluoromethyl sulfides.^10,11^ In particular the direct catalytic introduction of SCF_3_ is an attractive approach. While aryl halides^12^ or boronic acids^13^ have successfully been converted to C–SCF_3_ *via* metal catalyzed cross-coupling strategies or oxidative protocols,^14^ to date, there is no report of a direct and catalytic trifluoromethylthiolation of Csp^2^–O bonds.

## Results and discussion

Given the widespread abundance of phenols, the trifluoromethylthiolation of phenol derivatives would be highly attractive for synthetic diversity. In this context, the scope could in principle range from more activated derivatives (*e.g.* aryl triflates) to the least reactive derivatives, *i.e.* aryl ethers which are present in biomass feedstocks (such as lignin^15^).^6^ However, while Ni-catalysis has recently been successfully utilized to activate aromatic ethers,^3^ we hypothesized that there might be a fundamental reactivity conflict in introducing SCF_3_: the created SCF_3_-product would be expected to be inherently more reactive towards oxidative addition^16^ which may impede further transformation.

**Fig. 1:**
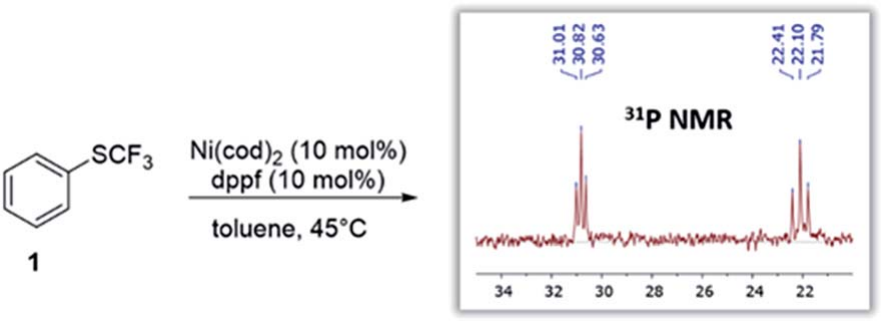
Reaction of catalyst [(dppf)Ni(cod)] with the desired product (ArSCF_3_) leads to catalyst deactivation.

To test this, we subjected Ni(cod)_2_/dppf to PhSCF_3_ **1** (see Fig. 1). We recently showed that this system triggers the mild trifluoromethylthiolation of aryl chlorides, proceeding *via* Ni^(0)^/Ni^(II)^ catalysis with [(dppf)Ni(cod)] formed as the active catalyst.^12*e*^ In accordance with our hypothesis, the reaction of the [Ni^(0)^] catalyst with PhSCF_3_ is indeed seen, even under mild reaction conditions (45 °C), as judged by ^31^P-NMR spectroscopic analysis. A complete disappearance of the characteristic ^31^P-NMR singlet signal of [(dppf)Ni^(0)^(cod)] (33.8 ppm)^12*e*^ occurred, and the formation of a new species was seen that appears as two triplets at 30.8 ppm (with *J* = 23.0 Hz) and at 22.1 ppm (with *J* = 37.6 Hz) by ^31^P-NMR spectroscopic analysis (see Fig. 1). While our efforts to structurally characterize the latter by X-ray crystallography have so far been unsuccessful, the formed species clearly constitutes a catalyst deactivation product. The subjection of this species as a catalyst (or also stoichiometrically) in the trifluoromethylthiolation of aryl chlorides did not yield ArSCF_3_. This indicates that oxidative addition by a [Ni^(0)^] catalyst to the product is facile and eventually leads to catalytically inactive species. To achieve productive catalysis and high overall conversion, it is therefore of utmost importance to avoid this deactivation process.

Our computational assessment^17^ of the oxidative addition of [(dppf)Ni(cod)] to Ph-SCF_3_ **1** suggests an activation free energy barrier of Δ*G* ^‡^ = 19.2 kcal mol^–1^, and it uses the M06L method with a CPCM solvation model to account for toluene and the mixed 6-311++G(d,p) and LANL2DZ (for Ni, Fe) basis set.^17,18^

This value now sets the bar for the possible reaction scope. The ‘to-be-transformed’ bond must show a barrier *lower* than 19.2 kcal mol^–1^ to avoid catalyst loss *via* an unproductive reaction with the product (ArSCF_3_).

### Identification of suitable leaving groups – computational assessment & experimental tests

**Fig. 2:**
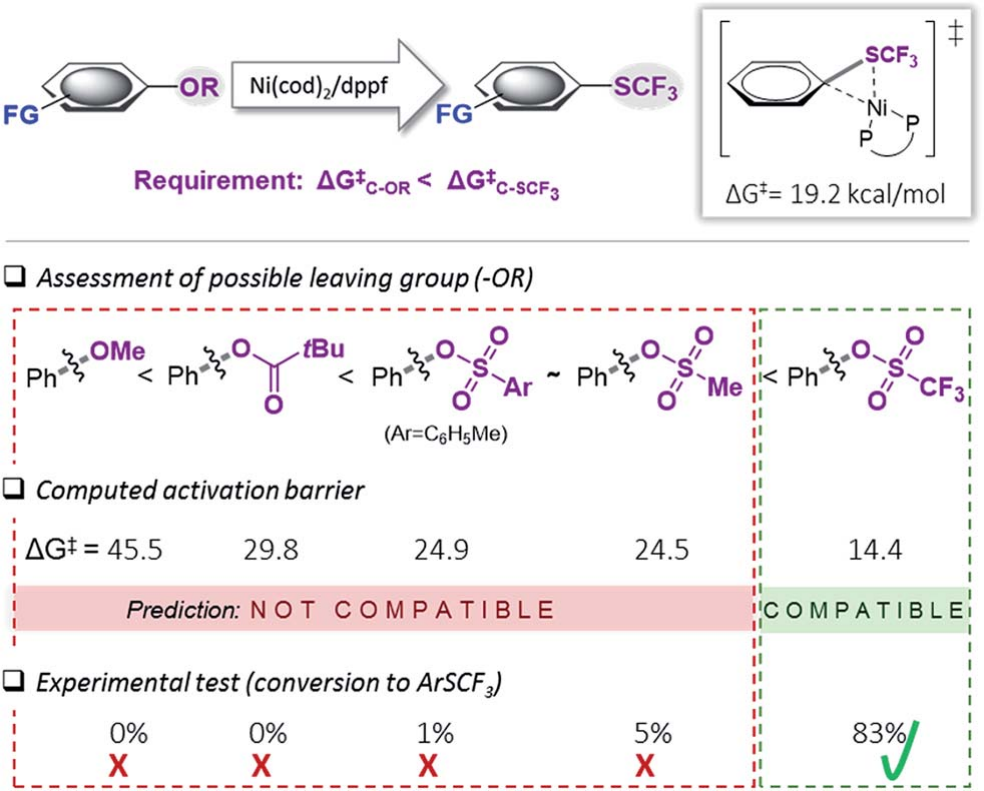
Calculated free energy barrier (Δ*G* ^‡^) for the oxidative addition of [(dppf)Ni^(0)^(cod)] to various Ph–OR and the testing of the prediction. Free energies in kcal mol^–1^, calculated at CPCM (toluene) M06L/6-311++G(d,p) with LANL2DZ (for Ni, Fe).^17^

We subsequently undertook computational studies to identify matching leaving groups ‘*OR*’ (Fig. 2) that would show the desired greater reactivity than the Csp^2^–SCF_3_ bond. For the cleavage of the C–O bonds, mechanistic support for Ni^(0)^/Ni^(II)^ ^5i,6^ and also Ni^(I)^-catalysis^19^ has previously been reported. However, on the basis of our previous mechanistic study^12*e*^ and the observation that (dppf)Ni^(I)^Cl is ineffective as a catalyst in C–SCF_3_ bond formation,^12e,20^ as a first approximation, we calculated the activation barrier of oxidative addition using [(dppf)Ni^(0)^(cod)] to a range of phenol derivatives (Ph–OR), with R = alkyl (ether), R′C=O (pivalate), SO_2_R′′ (sulfonic esters). Fig. 2 presents the results. This computational assessment suggests that in the context of C–O to C–SCF_3_ conversion, the inherently high reactivity of C–SCF_3_ only allows for triflate precursors as suitable starting materials. Alternative C–O leaving groups that have previously been employed in the Ni-catalyzed construction of inert C–C bonds, such as aryl ethers (OMe), mesylates (OMs), tosylates (OTs) or pivalates (OPiv)^3,6^ are predicted to be incompatible with Ni^(0)^-catalyzed trifluoromethylthiolation, as they would generally be less reactive than Ar–SCF_3_, hence favoring catalyst deactivation *via* reaction with the product.^21^

To experimentally test this computationally predicted trend, we subjected Ni(cod)_2_/dppf along with the easily accessible SCF_3_-source (Me_4_N)SCF_3_ to Ar–OR derivatives (in toluene at 45 °C), ranging from the predicted low (aryl ether) to high (aryl triflate) reactivity (Fig. 2). In accordance with expectations, at best, a low conversion was seen for phenyl mesylates (5%), tosylates (1%) or pivalates (0%). In stark contrast, phenyl triflate showed excellent conversion to PhSCF_3_ (83%).

We additionally followed the conversion ArOTf → ArSCF_3_ with ReactIR®. This analysis showed that the transformation was rapid, being essentially complete in 1.5 h with only little increase in conversion over the subsequent hours (see ESI, Fig. S1†). We also analyzed the reactions of those substrates that showed little conversion (≤5%), *i.e.* ArOMs and ArOTs, by ^31^P-NMR spectroscopic analyses. We observed that essentially all of the [Ni^(0)^] catalyst had transformed to the catalytically inactive species described in Fig. 1 within 3 h reaction time. This clearly highlights that while [Ni^(0)^] is in fact capable of reacting with Ph–OMs or –OTs, the catalyst is rapidly consumed as soon as some of the more reactive PhSCF_3_ molecules are generated. This corroborates with the strict requirement of suitably matching functionality and tailored reactivity progression from a “more” to “less reactive” functionality.

### Computational assessment of functional group tolerance

**Fig. 3:**
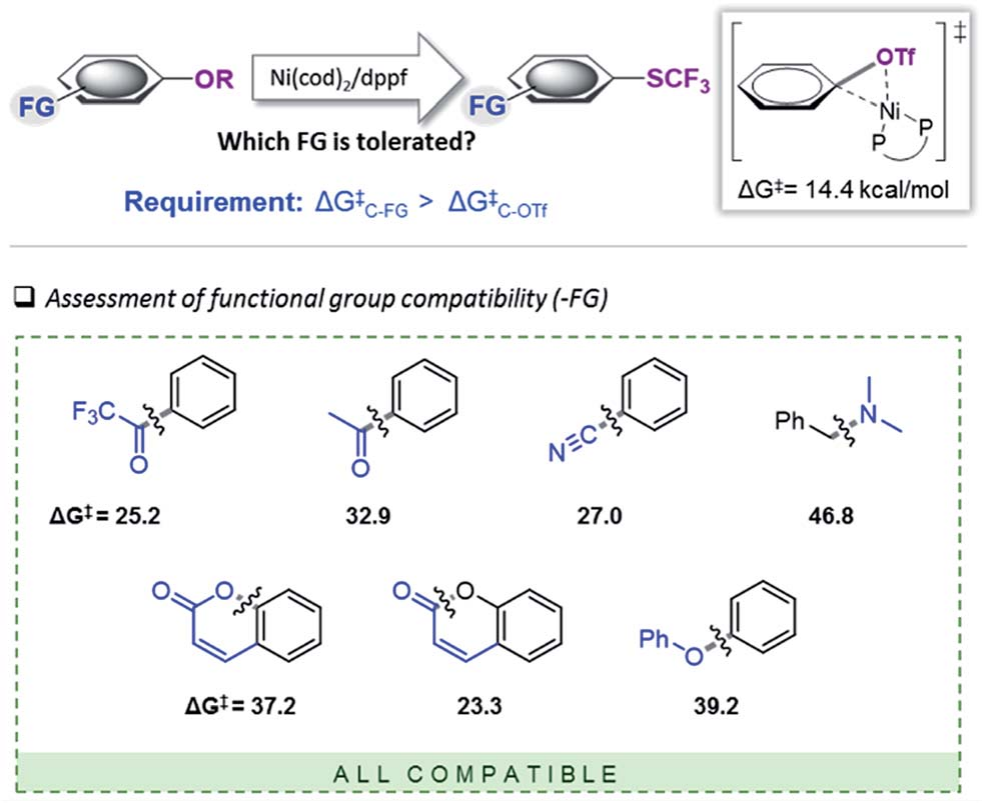
Computational scoping. Activation free energies (in kcal mol^–1^) calculated at CPCM (toluene) M06L/6-311++G(d,p) & LANL2DZ (for Ni, Fe)^17^ for the addition of [(dppf)Ni(cod)].

We subsequently set out to test for the generality of the identified Ni-catalyzed trifluoromethylthiolation of activated C–O bonds and computationally assess the functional group (FG) tolerance (see Fig. 3). As we determined a barrier of Δ*G* ^‡^ = 14.4 kcal mol^–1^ for the oxidative addition of [(dppf)Ni^(0)^(cod)] to Ph–OTf, all additional functional groups (FG) in the substrates will only be compatible if the reactivity of the C–FG bond is lower than that of Ph–OTf.

The computational results depicted in Fig. 3 suggest a tolerance of the protocol to ketone functional groups, C–C or benzylic C–O bonds. In all cases, the requirement of Δ*G*‡C–FG > 14.4 kcal mol^–1^ is fulfilled. Even aromatic C–CN bonds that were previously shown to be reactive under Ni-catalysis conditions^22^ are predicted to be compatible.

### SCF_3_-coupling of aryl triflates

On the basis of this computationally guided substrate scope, we subjected a range of aryl triflates to standard catalysis conditions. Table 1 presents the results. A number of aryl- and heteroaryl triflates were coupled in good to excellent yields. The transformation was compatible with ketone (6, 7 and 8, Table 1), ether (9) and cyano (5) functional groups. Two heterocyclic examples (10, 11) were also trifluoromethylthiolated in good yields (see Table 1).

**Table 1:** Ni(0)-catalyzed trifluoromethylthiolation of Ar-OTf^*a*^

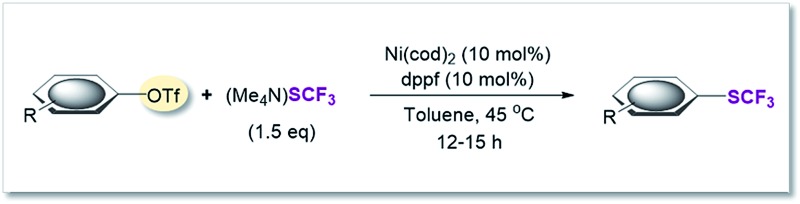
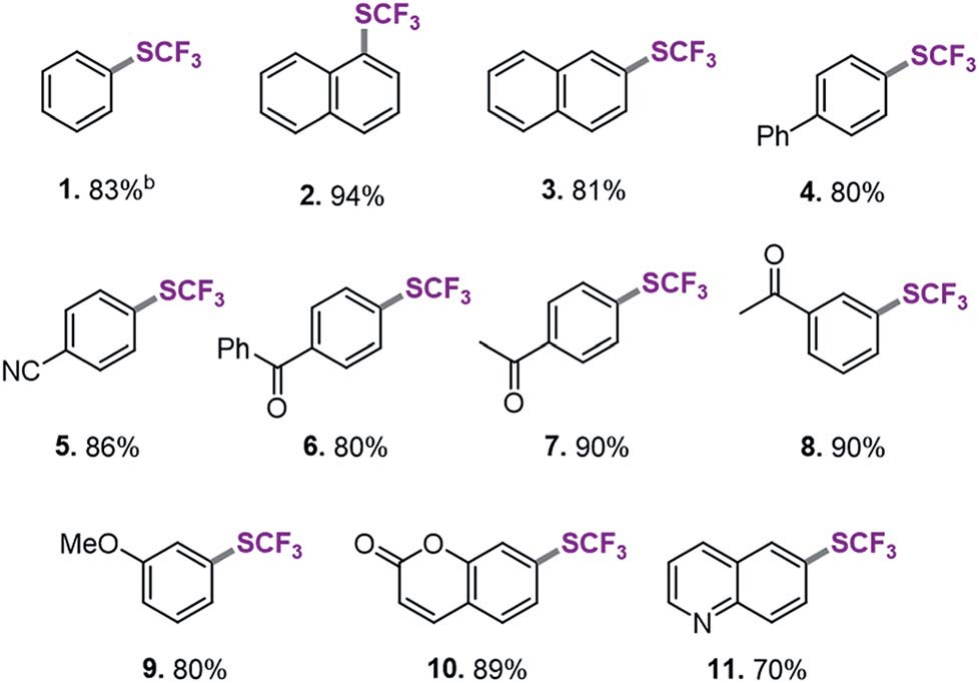

^*a*^Ni(cod)_2_ (11.0 mg, 0.04 mmol), dppf (22.2 mg, 0.04 mmol), aryl triflate (0.4 mmol), (Me_4_N)SCF_3_ (104 mg, 0.6 mmol), toluene (2 mL), under inert atmosphere, isolated yield.

^*b*^Yield determined by ^19^F-NMR analysis using PhCF_3_ as the internal standard.

**Scheme 1:**
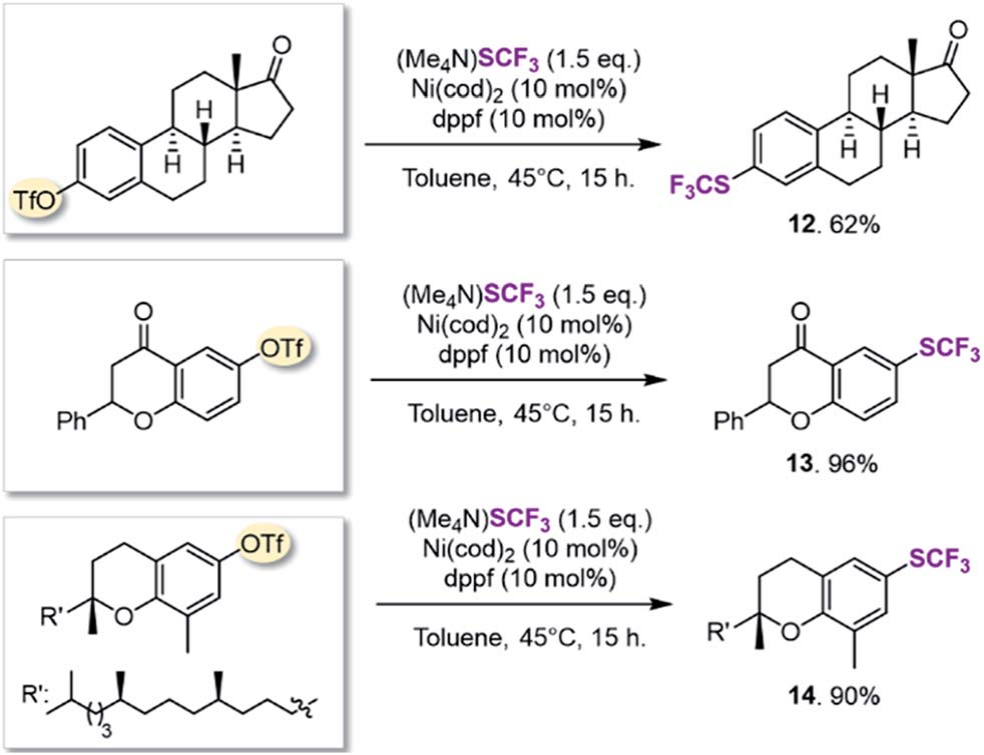
Synthesis of bioactive molecules.

We next searched for bioactive molecules of greater complexity that would fulfil our reactivity requirements and show compatibility with the computationally predicted scope. Estrone (an estrogenic hormone), 6-hydroxy flavanone (a plant secondary metabolite used *inter alia* as an antioxidant) and δ-tocopherol (vitamin E) show an excellent functional group match, containing predominantly ketone and benzylic C–O bonds that are predicted to be less reactive than C–OTf and C–SCF_3_. Trifluoromethylthiolation was successfully accomplished in 62–96% yield, highlighting the potential of this method for pharmaceutical applications (see Scheme 1).

### SCF_3_-coupling of vinyl triflates

Vinyl SCF_3_-compounds are also of significance, finding applications as herbicides for example.^23^ However, the current methodological repertoire to access these compounds relies predominantly on indirect strategies^24^ or requiring stoichiometric amounts of metal.^13b,25^ The direct construction of C_vinyl_–SCF_3_ in a catalytic manner would be a highly attractive approach. It has been accomplished *via* the Cu-catalyzed trifluoromethylthiolation of vinyl boronic acids with electrophilic SCF_3_-sources.^13c–e^ In a nucleophilic context, the catalytic installation of C_vinyl_–SCF_3_ is limited to vinyl iodides and requires harsh reaction conditions (110 °C).^26^

A mild Ni-catalyzed conversion of readily accessible C_vinyl_–OR derivatives to C_vinyl_–SCF_3_ would thus substantially widen the synthetic repertoire.

Our calculation of the barrier for the oxidative addition of [Ni^(0)^] to C_vinyl_–SCF_3_ indicated Δ*G* ^‡^ = 18.8 kcal mol^–1^. This barrier constitutes the upper limit for the reactivity of a potential leaving group (OR). C_vinyl_–OPiv and C_vinyl_–OMs show higher or similarly high barriers for oxidative addition (Δ*G* ^‡^ = 22.1 and 17.7 kcal mol^–1^) and are hence ruled out. C_vinyl_–OTf on the other hand is predicted to be highly reactive (Δ*G* ^‡^ = 5.2 kcal mol^–1^) and should hence be a compatible match.

After applying standard catalysis conditions,^27^ we successfully transformed a number of vinyl triflates to the corresponding trifluoromethylthiolated counterparts (see Table 2).

**Table 2:** Ni(0)-catalyzed trifluoromethylthiolation of vinyl–OTf^*a*^

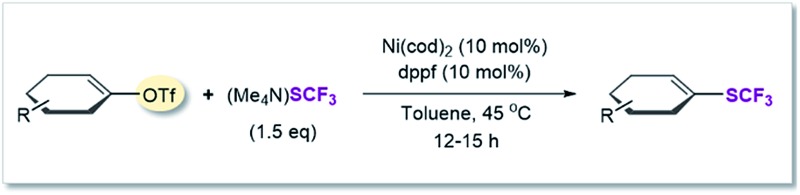
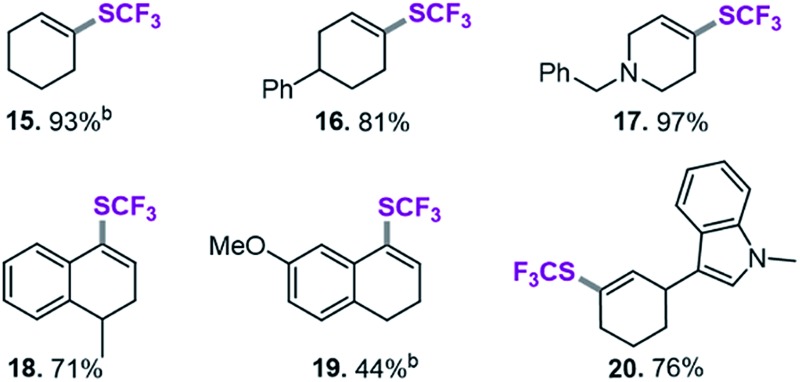

^*a*^Ni(cod)_2_ (5.5 mg, 0.02 mmol), dppf (11.1 mg, 0.02 mmol), vinyl triflate (0.2 mmol), (Me_4_N)SCF_3_ (52 mg, 0.3 mmol), PhCN (20.6 mg, 0.2 mmol),^27^ toluene (1 mL), under inert atmosphere, isolated yield.

^*b*^Yield determined by ^19^F-NMR analysis using PhCF_3_ as the internal standard.

The protocol proved to be compatible with a heterocyclic moiety (**20**, Table 2), a benzyl protecting group (**17**), and was successful for fully aliphatic (**15**) as well as conjugated (**18**, **19**) vinyl triflate derivatives. Compound **19** (Table 2) was afforded in a slightly lower yield (44%). However, upon closer inspection, it became clear that this was related to the inherent instability of the vinyl triflate starting material.

### Assessment of aryl and vinyl nonaflates

We therefore shifted our attention to potentially more stable analogues and considered nonaflates.^28^ Both, aryl and vinyl nonaflates are computationally predicted to be compatible with Ni-catalyzed trifluoromethylthiolation, showing similarly low or even lower barriers for oxidative addition by [Ni^(0)^] than the corresponding triflates (Δ*G* ^‡^ = 4.8 for addition to C_vinyl_–ONf and Δ*G* ^‡^ = 10.6 kcal mol^–1^ for addition to Ph–ONf). In accordance with these computational predictions, excellent conversions to aryl– and C_vinyl_–SCF_3_ were observed (see Table 3). Particularly notable is the synthesis of **19′** (Table 3) which was now high-yielding (as opposed to its preparation in Table 2), reflecting the greater robustness of vinyl nonaflates over vinyl triflates.^29^

**Table 3:** Ni(0)-catalyzed trifluoromethylthiolation of vinyl and aryl nonaflates^*a*^ ^,^ ^*b*^

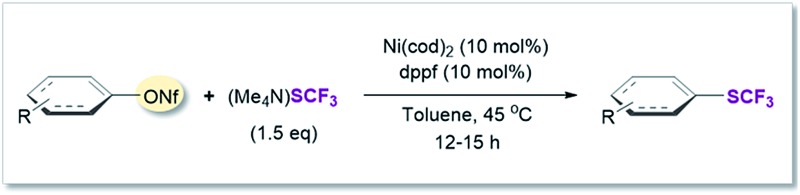
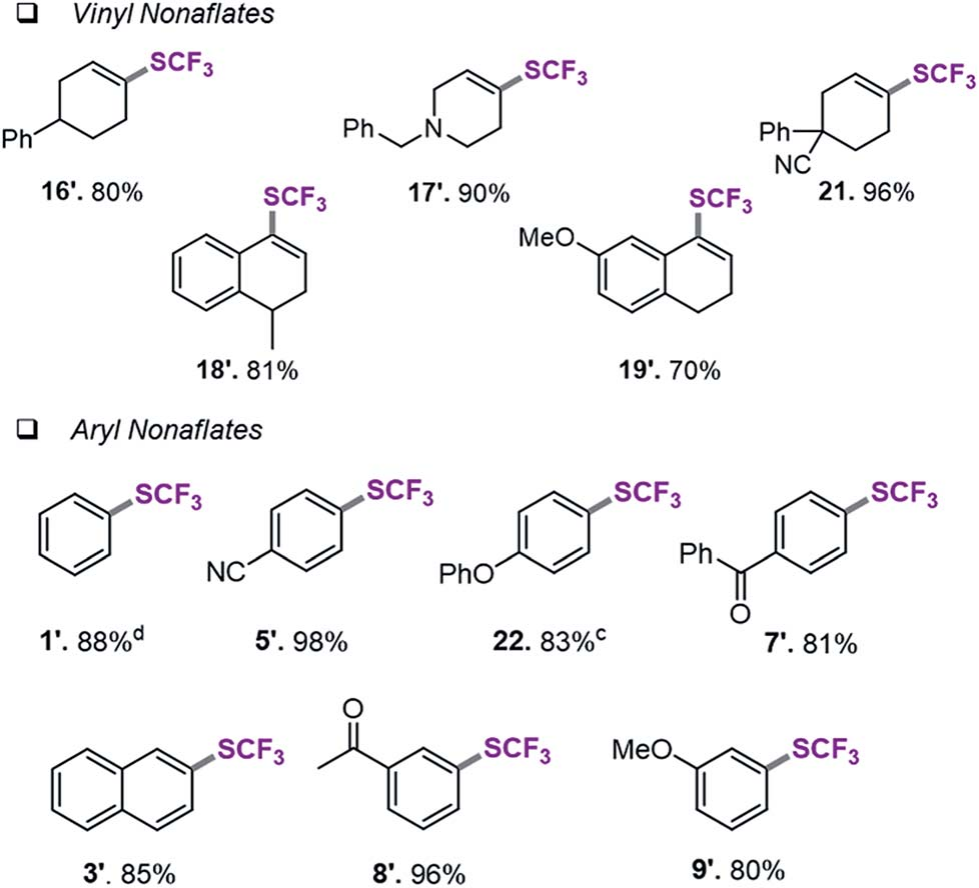

^*a*^Conditions for the coupling of vinyl nonaflates: Ni(cod)_2_ (5.5 mg, 0.02 mmol), dppf (11.1 mg, 0.02 mmol), vinyl nonaflate (0.2 mmol), (Me_4_N)SCF_3_ (52 mg, 0.3 mmol), PhCN (20.6 mg, 0.2 mmol),^27^ toluene (1 mL), under inert atmosphere, isolated yield.

^*b*^Conditions for the coupling of aryl nonaflates Ni(cod)_2_ (11.0 mg, 0.04 mmol), dppf (22.2 mg, 0.04 mmol), aryl nonaflate (0.4 mmol), (Me_4_N)SCF_3_ (104 mg, 0.6 mmol), toluene (2 mL), under inert atmosphere, isolated yield.

^*c*^Reaction performed with MeCN (16.4 mg, 0.4 mmol).

^*d*^Yield determined by ^19^F-NMR analysis using PhCF_3_ as the internal standard.

## Conclusions

The inherently high reactivities of Ni-catalysts may be fundamentally at conflict with introducing a wide range of functional groups, as shown here for the introduction of the pharmaceutically and agrochemically valuable SCF_3_ group. We identified that the reaction of the Ni-catalyst with the desired product, ArSCF_3_, triggers undesirable catalyst deactivation reactions that ultimately inhibit catalysis. The overall substrate scope is therefore dictated by the reactivity of the desired functionality towards the catalyst (here: C–SCF_3_). The application of computational tools allowed for the identification of matching functional groups in terms of suitable leaving groups and tolerated functional groups. As a result, the first Ni-catalyzed C–SCF_3_ coupling of aryl and vinyl C–O bonds has been developed. Given the highly reactive nature of C–SCF_3_, only those C–OR derivatives of even greater reactivity, *i.e.* triflates and nonaflates, allow for efficient C–SCF_3_ coupling. The protocol is mild, general and operationally simple.

Given that computational methods, software and hardware have evolved to a level, at which calculations can nowadays frequently be done faster than experiments,^30^ we anticipate that the herein applied approach will find applications in the development of, but not limited to, homogeneous Ni-catalysis.
