# Supplementary material for: Nickel-catalyzed trifluoromethylthiolation of Csp2–O bonds
Source: Chem Sci. 2015 Oct 30;7(2):1076–81. doi: 10.1039/c5sc03359d (PMC5635847; doi:10.1039/c5sc03359d)
Supplement: Supplementary file 1 [file SC-007-C5SC03359D-s001.pdf]

Electronic Supplementary Material (ESI) for Chemical Science.
This journal is © The Royal Society of Chemistry 2015

Supporting Information

Alexander B. Dürr[+], Guoyin Yin[+], Indrek Kalvet, Francois Napoly and Franziska Schoenebeck*

Contents

1. General Information 3

1.1. Reaction Monitoring by ReactIR 4

2. Synthetic procedures 6

2.1. Synthesis of aryl triflates 6

2.2. Synthesis of aryl nonaflates 9

2.3. Synthesis of vinyl triflates 11

2.4. Synthesis of vinyl nonaflates. 14

2.4.1. General procedure for the synthesis of vinyl nonaflates. 14

2.4.2. Characterization data of vinyl nonaflates. 14

2.5. Nickel-catalyzed trifluoromethylthiolation of aryl-LG 16

2.6. Nickel-catalyzed trifluoromethylthiolation of vinyl-OTf/ONf 20

2.6.1. General procedure for nickel-catalyzed trifluoromethylthiolation of vinyl-OTf/ONf 20

2.6.2. Characterization data of vinyl-SCF_3 20

3. Computational details 22

4. References 67

5. NMR Spectra 68

1. General Information

^{1}H, ^{13}C and ^{19}F NMR spectra were recorded either on Varian V-NMRS 600, Varian V-NMRS 400 or Varian Mercury 300 spectrometer. ^{1}H and ^{13}C spectra are referenced to residual solvent signals; $CDCl_3$ 7.26 ppm for ^{1}H and 77.2 ppm for ^{13}C; CD_2Cl_2 5.30 ppm for ^{1}H and 54.0 ppm for ^{13}C. Chemical shifts (δ) of ^{19}F NMR spectra are reported in ppm relative to trifluorotoluene (-62.80 ppm). Chemical shifts (δ) of ^{31}P NMR spectra are given in ppm relative to trimethyl phosphate (2.36 ppm). Coupling constants (*J*) are reported in Hz and coupling patterns are described as br = broad, s = singlet, d = doublet, t = triplet, q = quartet, m = multiplet. For the nonaflate substrates, the ^{13}C NMR signals of the nonaflate functionality are not presented due to their complex C-F splitting.[1] High resolution mass spectra (HRMS) were recorded on a Thermo Scientific LTQ Orbitrap XL spectrometer with positive ion mode. Melting points were measured with a Büchi Melting Point B-540 apparatus. Flash column chromatography was performed with Merck silica gel 60 (35–70 mesh). Reactions were monitored by thin layer chromatography (TLC) with aluminum sheets silica gel 60 F254 from Merck with detection by UV light.

In-situ FTIR reaction analysis was performed using a Mettler-Toledo ReactIR™ 15 instrument fitted with a DST Series AgX Fiber Conduit probe.

$Ni(COD)_2$, dppf, MeCN, *N*-(5-Chloro-2-pyridyl)bis(trifluoromethanesulfonimide) (Reagent A) and PhCN were purchased from Sigma Aldrich. Toluene, DCM and THF were dried by solvent purification system (Innovative Technology PS-MD-5). Unless stated otherwise, all starting materials were commercially available and used as received.

$(Me_4N)SCF_3$ was prepared according to its corresponding literature procedure.[2,3] Tetramethylammonium fluoride and sulfur were purchased from Sigma Aldrich and trifluoromethyltrimethylsilane from TCI.

1.1. Reaction Monitoring by ReactIR

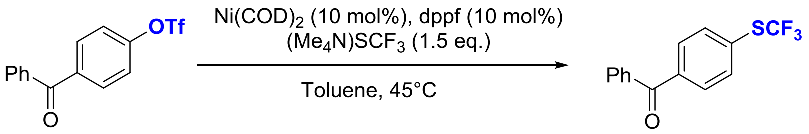

In a nitrogen filled glovebox the solution of the catalyst was prepared by mixing $Ni(COD)_2$ (5.5 mg, 0.02 mmol, 10 mol%) and dppf (11.1 mg, 0.02 mmol, 10 mol%) in 0.5 ml dry toluene and stirred at room temperature. Outside the glovebox, 4-benzoylphenyl trifluoromethansulfonate (66 mg, 0.2 mmol, 1 eq.) and $(Me_4N)SCF_3$ (52 mg, 0.3 mmol, 1.5 eq.) were placed in an oven dried, 3x evacuated and N_2 flushed 5ml Schlenk tube equipped with a stir bar. The tube was sealed with a rubber septum and 0.5 ml dry toluene were added. The mixture was stirred and heated to 45°C. After the suspension reached the desired temperature, the rubber septum was removed under N_2 flow and the FTIR probe was inserted, fitted to a plastic septum. Once a stable FTIR spectrum was observed, the beforehand prepared solution of the catalyst was added and the reaction was monitored.

Reaction progress was monitored by FTIR using a single peak at 1164 cm^{-1} for the product and a single peak at 1217 cm^{-1} for the starting material.

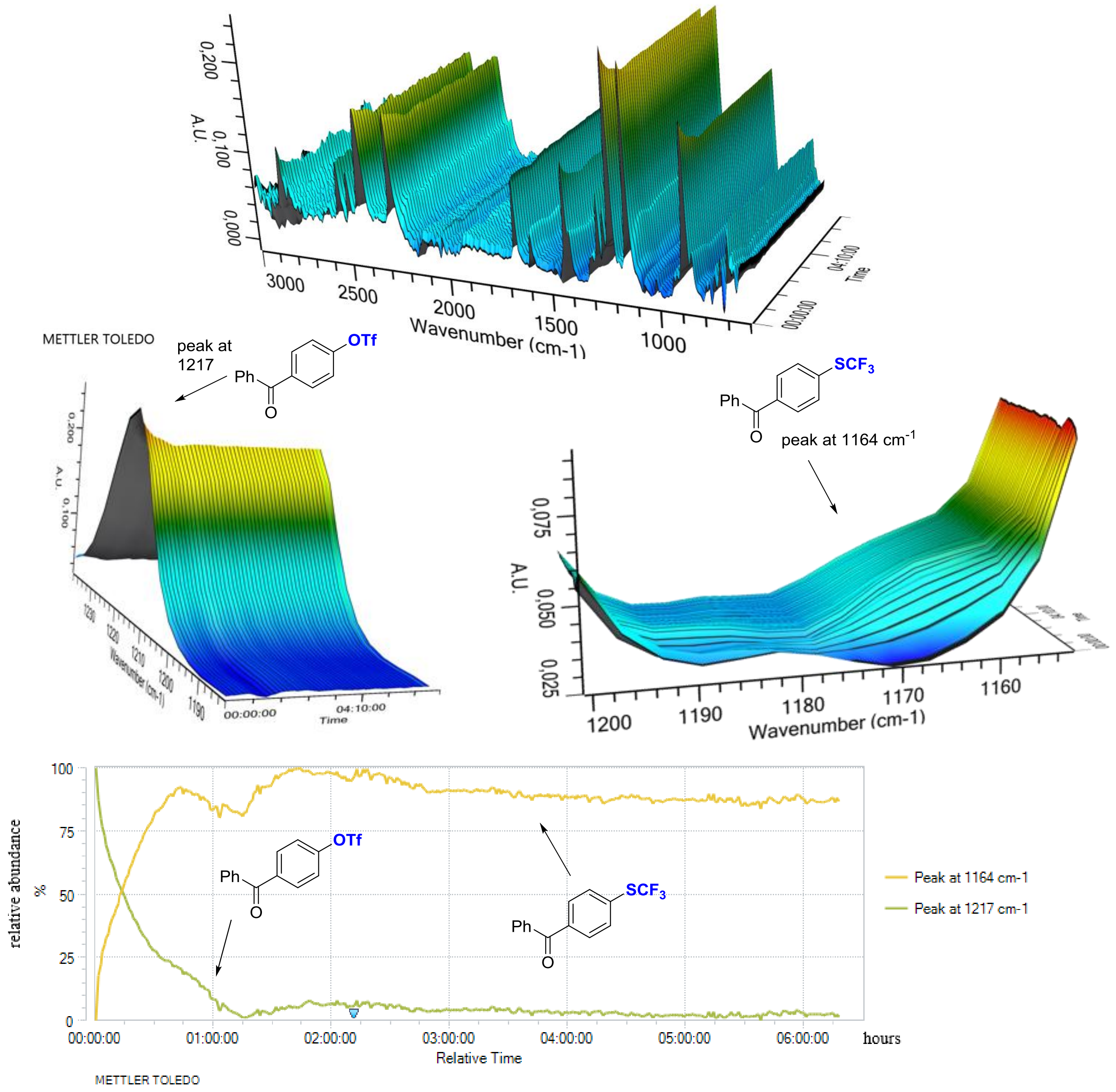

Figure S1. In situ ReactIR™ profiles for the trifluoromethylthiolation of 4-benzoylphenyl trifluoromethanesulfonate.

2. Synthetic procedures

2.1. Synthesis of aryl triflates

2.1.1. General procedure for the synthesis of aryl triflates

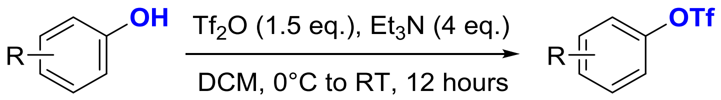

The phenol substrate (1.0 eq.) and triethylamine (4.0 eq.) were added to dry DCM (to give a saturated solution of phenol). The solution was then cooled to 0°C. Trifluoromethanesulfonic anhydride (1.5 eq.) was added dropwise. After 2 h, the solution was allowed to warm to room temperature and stirred for 12 hours. Thereafter, H_2O (50 mL) was added, the organic layer was separated, washed with saturated brine and dried over anhydrous Na_2SO_4. The solution was concentrated under reduced pressure and the residue was directly subjected to purification by flash column chromatography to give the pure product.[4]

2.1.2. Characterization data of aryl triflates

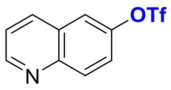

Quinolin-6-yl trifluoromethanesulfonate: The title product was obtained after column chromatography (hexane/Et_2O = 7/3) as a yellow solid with 81% yield. ^{1}H NMR (600 MHz, $CDCl_3$) δ (ppm) 9.00 (d, *J* = 2.8 Hz, 1H), 8.22-8.20 (m, 2H), 7.76 (d, *J* = 2.7 Hz, 1H), 7.61 (dd, *J* = 9.2, 2.7 Hz, 1H), 7.52-7.50 (m, 1H). ^{13}C NMR (151 MHz, $CDCl_3$) δ (ppm) 151.6, 147.0, 136.1, 132.4, 128.3, 123.1, 122.4, 119.2 (q, *J* = 320 Hz), 118.7, 115.6. ^{19}F NMR (564 MHz, $CDCl_3$) δ (ppm) -72.74 (s, 3F). These data are in agreement with those reported previously in the literature.[5]

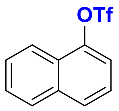

Naphthalen-1-yl trifluoromethanesulfonate: The title product was obtained after column chromatography (hexane/Et_2O = 95/5) as a colourless oil with 95% yield. ^{1}H NMR (600 MHz, $CDCl_3$) δ (ppm) 8.10 (d, *J* = 8.3 Hz, 1H), 7.93 (d, *J* = 7.6 Hz, 1H), 7.88 (dd, *J* = 6.6, 2.6 Hz, 1H), 7.68-7.59 (m, 2H), 7.52-7.45 (m, 2H). ^{13}C NMR (151 MHz, $CDCl_3$) δ (ppm) 145.8, 135.0, 128.7, 128.2, 128.0, 127.5, 126.5, 125.3, 120.9, 118.9 (q, *J* = 320 Hz), 117.3. ^{19}F NMR (564 MHz, $CDCl_3$) δ (ppm) -73.40 (s, 3F). These data are in agreement with those reported previously in the literature.[6]

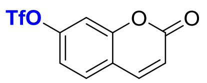

2-oxo-2H-chromen-7-yl trifluoromethanesulfonate: The title product was obtained after column chromatography (hexane/Et_2O = 1/1) as a white solid with 95% yield. ^{1}H NMR (600 MHz, $CDCl_3$) δ (ppm) 7.72 (d, *J* = 9.6 Hz, 1H), 7.59 (d, *J* = 8.6 Hz, 1H), 7.29 (d, *J* = 2.2 Hz, 1H), 7.23 (dd, *J* = 8.6, 2.4 Hz, 1H), 6.50 (d, *J* = 9.6 Hz, 1H). ^{13}C NMR (151 MHz, CD_2Cl_2) δ (ppm) 159.7, 155.2, 151.3, 142.7, 130.2, 119.6 (q, *J* = 320 Hz), 119.3, 118.3, 118.1, 110.9. ^{19}F NMR (564 MHz, $CDCl_3$) δ (ppm) -72.58 (s, 3F). These data are in agreement with those reported previously in the literature.[7]

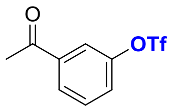

3-acetylphenyl trifluoromethanesulfonate: The title product was obtained after column chromatography (hexane/Et_2O = 9/1) as a brown oil with 96% yield. ^{1}H NMR (600 MHz, $CDCl_3$) δ (ppm) 7.98-7.97 (m, 1H), 7.85 (s, 1H), 7.58 (dd, *J* = 7.9, 7.9 Hz, 1H), 7.49-7.47 (m, 1H), 2.63 (s, 3H). ^{13}C NMR (151 MHz, $CDCl_3$) δ (ppm) 195.9, 150.0, 139.4, 130.8, 128.3, 125.9, 121.1, 118.9 (q, *J* = 320 Hz), 26.8. ^{19}F NMR (564 MHz, $CDCl_3$) δ (ppm) -72.83 (s, 3F). These data are in agreement with those reported previously in the literature.[5]

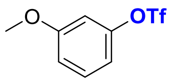

3-methoxyphenyl trifluoromethanesulfonate: The title product was obtained after column chromatography (hexane/Et_2O = 95/5) as a colourless oil with 92% yield. ^{1}H NMR (600 MHz, $CDCl_3$) δ (ppm) 7.34 (dd, *J* = 8.3, 8.3 Hz, 1H), 6.93 (dd, *J* = 8.4, 1.9 Hz, 1H), 6.87 (dd, *J* = 8.2, 2.0 Hz, 1H), 6.81 (dd, *J* = 2.3, 2.3 Hz, 1H), 3.83 (s, 3H). ^{13}C NMR (151 MHz, $CDCl_3$) δ (ppm) 161.0, 150.4, 130.7, 118.9 (q, *J* = 320 Hz), 114.3, 113.4, 107.6, 55.8. ^{19}F NMR (564 MHz, $CDCl_3$) δ (ppm) -72.94 (s, 3F). These data are in agreement with those reported previously in the literature.[8]

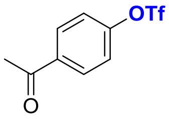

4-acetylphenyl trifluoromethanesulfonate: The title product was obtained after column chromatography (hexane/Et_2O = 8/2) as a colourless oil with 87% yield. ^{1}H NMR (600 MHz, $CDCl_3$) δ (ppm) 8.06 (d, *J* = 8.9 Hz, 2H), 7.38 (d, *J* = 8.8 Hz, 2H), 2.63 (s, 3H). ^{13}C NMR (151 MHz, $CDCl_3$) δ (ppm) 196.0, 152.4, 136.8, 130.6, 121.6, 118.7 (q, *J* = 320 Hz), 26.6. ^{19}F NMR (564 MHz, $CDCl_3$) δ (ppm) -72.79 (s, 3F). These data are in agreement with those reported previously in the literature.[8]

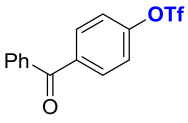

4-benzoylphenyl trifluoromethanesulfonate: The title product was obtained after column chromatography (hexane/Et_2O = 90/10) as a yellow solid with 95% yield. ^{1}H NMR (400 MHz, $CDCl_3$) δ (ppm) 7.95-7.87 (m, 2H), 7.80-7.78 (m, 2H), 7.68-7.58 (m, 1H), 7.51 (dd, *J* = 7.6, 7.6 Hz, 2H), 7.42-7.39 (m, 2H). ^{13}C NMR (101 MHz, $CDCl_3$) δ (ppm) 194.9, 152.1, 137.7, 136.9, 133.2, 132.3, 130.1, 128.7, 121.5, 118.9 (q, *J* = 320 Hz). ^{19}F NMR (376 MHz, $CDCl_3$) δ (ppm) -72.77 (s, 3F). These data are in agreement with those reported previously in the literature.[9]

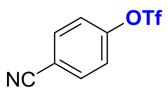

4-cyanophenyl trifluoromethanesulfonate: The title product was obtained after column chromatography (hexane/Et_2O = 7/3) as a colourless oil with 93% yield. ^{1}H NMR (400 MHz, $CDCl_3$) δ (ppm) 7.79 (d, *J* = 8.9 Hz, 2H), 7.43 (d, *J* = 8.8 Hz, 2H). ^{13}C NMR (101 MHz, $CDCl_3$) δ (ppm) 152.1, 134.6, 122.8, 119.1 (q, *J* = 320 Hz), 117.2, 113.1. ^{19}F NMR (376 MHz, $CDCl_3$) δ (ppm) -72.65 (s, 3F). These data are in agreement with those reported previously in the literature.[10]

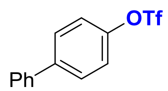

[1,1'-biphenyl]-4-yl trifluoromethanesulfonate: The title product was obtained after column chromatography (hexane/Et_2O = 9/1) as a colourless oil with 85% yield. ^{1}H NMR (400 MHz, $CDCl_3$) δ (ppm) 7.65 (d, *J* = 8.8 Hz, 2H), 7.56 (d, *J* = 8.3 Hz, 2H), 7.47 (dd, *J* = 7.7, 7.7 Hz, 2H), 7.41-7.38 (m, 1H) 7.35 (d, *J* = 8.7 Hz, 2H). ^{13}C NMR (101 MHz, $CDCl_3$) δ (ppm) 148.9, 141.7, 139.3, 129.0, 128.9, 128.0, 127.2, 121.6, 118.8 (q, *J* = 320 Hz). ^{19}F NMR (376 MHz, $CDCl_3$) δ (ppm) -72.81 (s, 3F). These data are in agreement with those reported previously in the literature.[4]

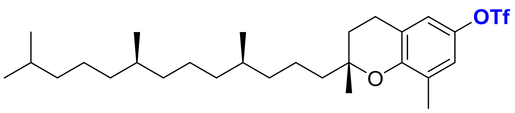

(*R*)-2,8-dimethyl-2-((4*R*,8*R*)-4,8,12-trimethyltridecyl)chroman-6-yl trifluoromethanesulfonate: The title product was obtained after column chromatography (hexane) as a colourless oil with 87% yield. ^{1}H NMR (400 MHz, $CDCl_3$) δ (ppm) 6.85 (d, *J* = 2.8 Hz, 1H), 6.81 (d, *J* = 2.8 Hz, 1H), 2.78-2.73 (m, 2H), 2.17 (s, 3H), 1.86-1.72 (m, 2H), 1.60-1.01 (m, 24H), 0.88-0.84 (m, 12H). ^{13}C NMR (101 MHz, $CDCl_3$) δ (ppm) 151.8, 141.6, 128.5, 121.8, 120.9; 119.2, 118.9 (q, *J* = 320 Hz), 77.0, 40.3, 39.5, 37.6, 37.6, 37.5, 37.4, 33.0, 32.8, 30.8, 28.1, 25.0, 24.6, 24.3, 22.9, 22.8, 22.6, 21.1, 19.9, 19.8, 16.3. ^{19}F NMR (376 MHz, $CDCl_3$) δ (ppm) -73.05 (s, 3F). These data are in agreement with those reported previously in the literature.[11]

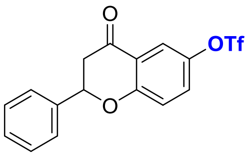

4-oxo-2-phenylchroman-6-yl trifluoromethanesulfonate: The title product was obtained after column chromatography (pentane/EtOAc = 7/1) as a yellowish solid with 94% yield. M.p. 131 - 133 °C. ^{1}H NMR (400 MHz, $CDCl_3$) δ (ppm) 7.83 (d, *J* = 3.1 Hz, 1H), 7.51-7.38 (m, 6H), 7.15 (d, *J* = 9.1 Hz, 1H), 5.53 (dd, *J* = 13.3, 2.9 Hz, 1H), 3.12 (dd, *J* = 17.0, 13.3 Hz, 1H), 2.96 (dd, *J* = 17.1, 3.0 Hz, 1H). ^{13}C NMR (151 MHz, $CDCl_3$) δ (ppm) 190.4, 160.8, 143.8, 138.0, 129.3, 129.2, 129.2, 126.3, 121.6, 120.6, 119.6 (q, *J* = 320 Hz), 119.6, 80.3, 44.2. ^{19}F NMR (376 MHz, $CDCl_3$) δ (ppm) -72.71 (s, 3F). HRMS (ESI) calculated for $C_{16}H_{11}O_5F_3SNa$: 395.0172, found: 395.0165.

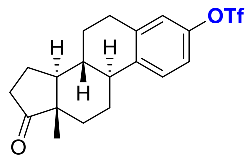

(8*R*,9*S*,13*S*,14*S*)-13-methyl-17-oxo-7,8,9,11,12,13,14,15,16,17-decahydro-6H cyclopenta[a]phenanthren-3-yl trifluoromethanesulfonate: The title product was obtained after column chromatography (pentane/EtOAc = 5/1) as a colourless oil with

96% yield. ^{1}H NMR (600 MHz, $CDCl_3$) δ (ppm) 7.34 (d, J = 8.7 Hz, 1H), 7.04 (dd, J = 8.7, 2.4 Hz, 1H), 6.99 (d, J = 2.4 Hz, 1H), 2.94 (dd, J = 8.5, 3.7 Hz, 2H), 2.52 (dd, J = 19.1, 8.8 Hz, 1H), 2.41-2.39 (m, 1H), 2.29 (td, J = 11.0, 4.0 Hz, 1H), 2.19-2.13 (m, 1H), 2.09-2.03 (m, 2H), 2.00-1.97 (m, 1H), 1.68-1.44 (m, 6H), 0.92 (s, 3H). ^{13}C NMR (151 MHz, $CDCl_3$) δ (ppm) 220.4, 148.1, 141.3, 140.3, 127.8, 121.7, 119.8 (q, J = 320 Hz), 118.8, 50.9, 48.3, 44.7, 38.3, 36.6, 32.1, 30.0, 26.6, 26.3, 22.0, 14.2. ^{19}F NMR (564 MHz, $CDCl_3$) δ (ppm) -72.99 (3F). These data are in agreement with those reported previously in the literature.[12]

2.2. Synthesis of aryl nonaflates

2.2.1. General procedure for the synthesis of aryl nonaflates

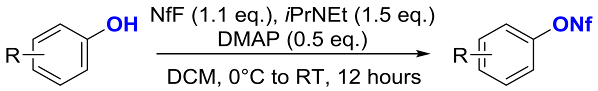

The phenol substrate (1.0 eq.) was dissolved in DCM (to yield a saturated solution). Under stirring, DMAP (0.5 eq.) and *i*Pr_2NEt (1.5 eq.) were added and the solution was cooled to 0°C. Then nonafluorobutanesulfonic fluoride (1.1 eq.) was added dropwise. After the addition, the solution was allowed to warm to room temperature and stirred overnight. The reaction mixture was washed with H_2O (20 ml/mmol phenol). The aqueous phase was extracted with DCM and the combined organic layers were washed with brine, dried over $MgSO_4$ and evaporated. The residue was purified by flash column chromatography to give the product.[13]

2.2.2. Characterization data of aryl nonaflates

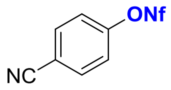

4-cyanophenyl 1,1,2,2,3,3,4,4,4-nonafluorobutane-1-sulfonate: The title product was obtained after column chromatography (hexane/EtOAc = 7/1) as a white solid with 79% yield. ^{1}H NMR (400 MHz, $CDCl_3$) δ (ppm) 7.79 (d, *J* = 9.0 Hz, 2H), 7.44 (d, *J* = 8.9 Hz, 2H). ^{13}C NMR (101 MHz, $CDCl_3$) δ (ppm) 152.4, 134.7, 122.8, 117.3, 113.1. ^{19}F NMR (376 MHz, $CDCl_3$) δ (ppm) -80.62 (t, *J* = 9.5 Hz, 3F), -108.39 to -108.47 (m, 2F), -120.75 to -120.88 (m 2F), -125.73 to -125.88 (m, 2F). These data are in agreement with those reported previously in the literature.[13]

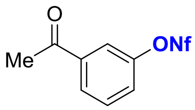

3-acetophenyl 1,1,2,2,3,3,4,4,4-nonafluorobutane-1-sulfonate: The title product was obtained after column chromatography (hexane/EtOAc = 5/1) as a colourless oil with 77% yield. ^{1}H NMR (400 MHz, $CDCl_3$) δ 7.99-7.96 (m, 1H), 7.86 (dd, *J* =2.1, 2.1 Hz, 1H), 7.59 (dd, *J* = 8.0, 8.0 Hz, 1H), 7.49 (dd, *J* = 8.0, 2.1 Hz, 1H), 2.63 (s, 3H). ^{13}C NMR (101 MHz, $CDCl_3$) δ (ppm) 195.9, 150.2, 139.4, 130.8, 128.3, 125.9, 121.2, 26.8. ^{19}F NMR (376 MHz, $CDCl_3$) δ -80.68 (t, *J* = 9.7 Hz, 3F), -108.71 to -108.91 (m, 2F), -120.81 to -120.97 (m, 2F), -125.79 to -125.92 (m, 2F). HRMS (ESI) calculated for $C_{12}H_7O_4F_9SNa$: 440.98135, found 440.98035.

.

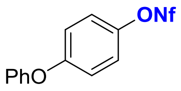

4-phenoxyphenyl 1,1,2,2,3,3,4,4,4-nonafluorobutane-1-sulfonate: The title product obtained after column chromatography (pentane/Et_2O = 4/1) as a colourless oil with 82% yield. ^{1}H NMR (600 MHz, $CDCl_3$) δ (ppm) 7.36 (dd, *J* = 8.0, 8.0 Hz, 2H), 7.21 (d, *J* = 9.2 Hz, 2H), 7.16 (dd, *J* = 7.4, 7.4 Hz, 1H), 7.03-6.99 (m, 4H). ^{13}C NMR (151 MHz, $CDCl_3$) δ (ppm) 157.5, 156.2, 144.8, 130.2, 124.5, 122.8, 119.8, 119.5. ^{19}F NMR (564 MHz, $CDCl_3$) δ (ppm) -80.74 (t, *J* = 8.6 Hz, 3F), -109.00 (m, 2F), -120.16 to -121.55 (m, 2F), -125.92 to -125.94 (m, 2F). HRMS (ESI) calculated for $C_{16}H_9O_4F_9S$: 468.0072, found 468.0072.

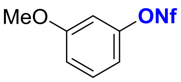

3-methoxyphenyl 1,1,2,2,3,3,4,4,4-nonafluorobutane-1-sulfonate: The title product was obtained after column chromatography (pentane/Et_2O = 4/1) as a colourless oil with 88% yield. ^{1}H NMR (400 MHz, $CDCl_3$) δ (ppm) 7.37 (dd, *J* = 8.3, 8.3 Hz, 1H), 6.96 (ddd, *J* = 8.5, 2.4, 0.8 Hz, 1H), 6.90 (dd, *J* = 8.2, 2.3 Hz, 1H), 6.86 (dd, *J* = 2.4, 2.4 Hz, 1H), 3.83 (s, 3H). ^{13}C NMR (101 MHz, $CDCl_3$) δ (ppm) 161.5, 151.0, 131.2, 114.6, 113.7, 108.1, 56.3. ^{19}F NMR (376 MHz, $CDCl_3$) δ (ppm) -81.09 (t, *J* = 9.8 Hz, 3F), -109.23 to -109.30 (m, 2F), -121.12 to -121.29 (m, 2F), -126.06 to -126.23 (m, 2F). HRMS (ESI) calculated for $C_{11}H_8O_4F_9S$: 406.9994, found 406.9993.

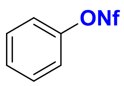

1-phenyl 1,1,2,2,3,3,4,4,4-nonafluorobutane-1-sulfonate: The title product was obtained after column chromatography (pentane/Et_2O = 9/1) as a colourless oil with 77% yield. ^{1}H NMR (400 MHz, CD_2Cl_2) δ (ppm) 7.52-7.40 (m, 3H), 7.34-7.28 (m, 2H). ^{13}C NMR (101 MHz, CD_2Cl_2) δ (ppm) 150.5, 130.9, 129.1, 121.9. ^{19}F NMR (376 MHz, $CDCl_3$) δ (ppm) -81.01 (t, J = 9.8 Hz, 3F), -109.22 to -109.35 (m, 2F), -121.12 to -121.16 (m 2F), -126.02 to -126.22 (m, 2F). These data are in agreement with those reported previously in the literature.[14]

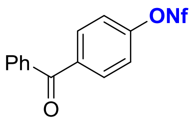

4-benzoylphenyl 1,1,2,2,3,3,4,4,4-nonafluorobutane-1-sulfonate: The title product was obtained after column chromatography (hexane/EtOAc = 5/1) as a white solid with 95% yield. M.p. 53 - 55 °C. ^{1}H NMR (400 MHz, CD_2Cl_2) δ 7.94 – 7.88 (m, 2H), 7.82 – 7.76 (m, 2H), 7.69 – 7.62 (m, 1H), 7.56 – 7.49 (m, 2H), 7.47 – 7.41 (m, 2H). ^{13}C NMR (101 MHz, CD_2Cl_2) δ 195.1, 152.8, 138.3, 137.4, 133.5, 132.7, 130.5, 129.1, 121.9. ^{19}F NMR (376 MHz, CD_2Cl_2) δ -81.01 (t, J = 9.8 Hz, 3H), -108.81 to -108.98 (m, 3H), -121.00 to -121.17 (m, 3H), -125.90 to -126.14 (m, 3H). HRMS (ESI) calculated for $C_{17}H_9O_4F_9SNa$: 502.99700, found 502.99585.

2.3. Synthesis of vinyl triflates

Synthesis of 1-benzyl-1,2,3,6-tetrahydropyridin-4-yl trifluoromethanesulfonate.

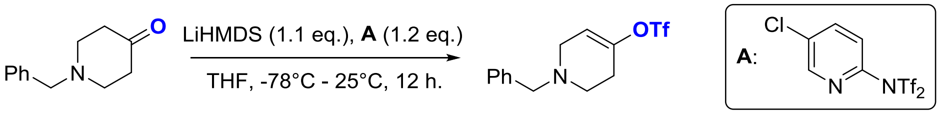

The reaction was performed under inert atmosphere. 1-benzylpiperidin-4-one (500 mg, 2.6 mmol, 1.0 eq.) and reagent **A** (1.25 g, 3.2 mmol, 1.2 eq.) were dissolved in dry THF (30 ml) and cooled to -78°C. LiHMDS (2.9 mL, 2.9 mmol, 1 M solution in THF, 1.1 eq.) was added dropwise. After completion of the addition, the mixture was allowed to warm to room temperature and stirred overnight. Subsequently the reaction was quenched with cold (~5°C), freshly prepared NaOH solution (5 wt% in water). The layers were separated, the aqueous layer was extracted with diethyl ether and the combined organic layers were washed with brine, dried over Na_2SO_4 and concentrated under reduced pressure. The title product was obtained with sufficient purity without further purification in 88% yield (735 mg).[15] ^{1}H NMR (400 MHz, $CDCl_3$) δ (ppm) 7.35-7.33 (m, 4H), 7.32-7.27 (m, 1H), 5.74-5.72 (m, 1H), 3.63 (s, 2H), 3.14-3.12 (m, 2H), 2.73 (t, J = 5.7 Hz, 2H), 2.48-2.44 (m, 2H). ^{13}C NMR (101 MHz, $CDCl_3$) δ (ppm) 147.4, 137.8, 129.1, 128.5, 127.5, 118.6 (q, J = 320 Hz), 116.3, 61.4, 50.6, 49.2, 28.5. ^{19}F NMR (376 MHz, $CDCl_3$) δ (ppm) -73.96 (s, 3F). These data are in agreement with those reported previously in the literature.[16]

Synthesis 1,2,3,6-tetrahydro-[1,1'-biphenyl]-4-yl trifluoromethanesulfonate

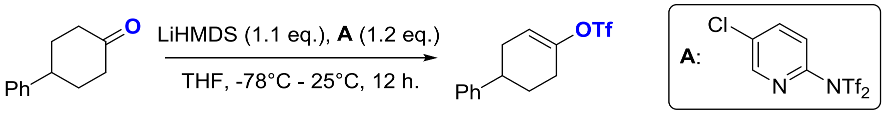

The reaction was performed under inert atmosphere. 4-phenylcyclohexan-1-one (500 mg, 2.87 mmol, 1.0 eq.) and **A** (1.24 g, 3.16 mmol, 1.1 eq.) were dissolved in dry THF (40 mL) and cooled to -78 °C. LiHMDS (3.2 mL, 1 M solution in THF, 1.1 eq.) was added dropwise. After completion of the addition, the mixture was allowed to warm to room temperature and stirred overnight. Subsequently the reaction was quenched with cold, freshly prepared NaOH solution (5 wt% in water). The layers were separated, the aqueous layer was extracted with Et_2O and the combined organic layers were washed with brine, dried over Na_2SO_4 and concentrated under reduced pressure. The title product was obtained after column chromatography (hexane/Et_2O = 9/1) as a colourless oil (870 mg) with 90% yield.[15] ^{1}H NMR (400 MHz, CD_2Cl_2) δ (ppm) 7.34-7.30 (m, 2H), 7.24-7.20 (m, 3H), 5.88-5.86 (m, 1H), 2.90-2.82 (m, 1H), 2.61-2.40 (m, 3H), 2.39-2.29 (m, 1H), 2.10-2.04 (m, 1H), 2.02-1.91 (m, 1H). ^{13}C NMR (101 MHz, CD_2Cl_2) δ (ppm) 149.1, 144.7, 128.8, 126.9, 126.8, 118.7 (q, J = 320 Hz), 117.6, 38.9, 31.7, 29.8, 28.0. ^{19}F NMR (376 MHz, CD_2Cl_2) δ (ppm) -73.99 (s, 3F). These data are in agreement with those reported previously in the literature.[15]

Synthesis of the 3-(1-methyl-1H-indol-3-yl)cyclohex-1-en-1-yl trifluoromethanesulfonate

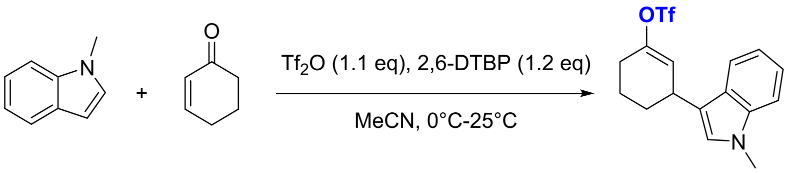

The reaction was performed under inert atmosphere. 1-methyl-1H-indole (3.93 g, 30 mmol, 3 eq.), cyclohex-2-en-1-one (0.96 g, 0.97 mL, 10 mmol, 1 eq.) and DTBP (2.30 g, 12 mmol, 1.2 eq.) were placed in a Schlenk flask and dissolved in acetonitrile (50 ml) at room temperature. The mixture was cooled to 0 ºC under vigorous stirring. Then triflic anhydride (3.10 g, 1.80 mL, 11 mmol, 1.1 eq.) was added dropwise. After the addition was finished, the stirring was continued for 10 minutes. Subsequently a concentrated $NaHCO_3$ solution (20 mL) was added. The reaction mixture was stirred for 1 hour and its temperature was kept constant during this period before it was allowed to warm to room temperature over 30 minutes. After stirring an additional 15 minutes at room temperature, dichloromethane (250 mL) was added to the mixture, the layers were separated, and the aqueous layer was extracted again with the same amount of dichloromethane. The combined organic layers were washed with brine (50 mL), dried over Na_2SO_4, concentrated. The title product was obtained after column chromatography (826 mg) as a yellow oil with 23% yield.[17] ^{1}H NMR (400 MHz, $CDCl_3$) δ 7.60 (d, J = 7.9 Hz, 1H), 7.32 (d, J = 8.2 Hz, 1H), 7.28-7.22 (m, 1H), 7.15-7.11 (m, 1H), 6.81 (s, 1H), 5.97-5.95 (m, 1H), 3.96 (d, J =

3.6 Hz, 1H), 3.76 (s, 3H), 2.44-2.41 (m, 2H), 2.07-2.04 (m, 1H), 2.04-1.74 (m, 3H). ^{13}C NMR (101 MHz, $CDCl_3$) δ (ppm) 150.0, 137.4, 126.9, 126.6, 122.0, 121.8, 119.1, 118.9, 118.4 (q, J = 320 Hz), 116.8, 109.6, 32.9, 32.3, 29.0, 27.9, 20.6. ^{19}F NMR (376 MHz, $CDCl_3$) δ -74.11 (s, 3F). These data are in agreement with those reported previously in the literature.[17]

Synthesis of the 7-methoxy-3,4-dihydronaphthalen-1-yl trifluoromethanesulfonate.

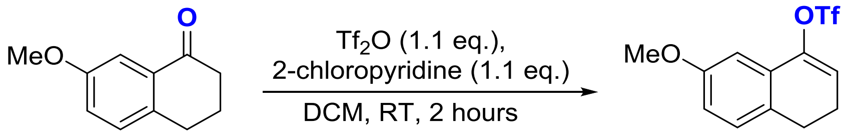

The reaction was performed under inert atmosphere. To a solution of 7-methoxy-3,4-dihydronaphthalen-1(2H)-one (1.00 g, 5.68 mmol, 1.0 eq.) in DCM (25 mL) at room temperature was added 2-chloropyridine (708 mg, 6.24 mmol, 1.1 eq.) and triflic anhydride (1.76 g, 6.24 mmol, 1.1 eq.). The reaction was stirred at room temperature for 2 hours. The cherry-coloured solution was then concentrated under reduced pressure. The title product was obtained after column chromatography (pentane/Et_2O = 95/5) as a colourless oil (1.71 g) with 98% yield.[18] ^{1}H NMR (400 MHz, $CDCl_3$) δ (ppm) 7.09 (d, J = 8.2 Hz, 1H), 6.91 (d, J = 2.6 Hz, 1H), 6.80 (dd, J = 8.2, 2.6 Hz, 1H), 6.03 (t, J = 4.8 Hz, 1H), 3.81 (s, 3H), 2.80 (t, J = 8.1 Hz, 2H), 2.49 (td, J = 8.1, 4.8 Hz, 2H). ^{13}C NMR (101 MHz, $CDCl_3$) δ (ppm) 158.7, 146.4, 129.7, 128.8, 128.3, 118.7 (q, J = 320 Hz), 118.5, 114.6, 107.2, 55.5, 26.1, 22.9. ^{19}F NMR (376 MHz, $CDCl_3$) δ (ppm) -73.69 (s, 3F). These data are in agreement with those reported previously in the literature.[19]

Synthesis of the 4-methyl-3,4-dihydronaphthalen-1-yl trifluoromethanesulfonate.

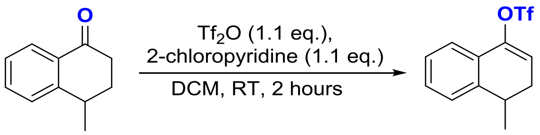

The reaction was performed under inert atmosphere. To a solution of 4-methyl-3,4-dihydronaphthalen-1(2H)-one (1.00 g, 6.24 mmol, 1.0 eq.) in DCM (25 mL) at room temperature was added 2-chloropyridine (780 mg, 6.87 mmol, 1.1 eq.) and triflic anhydride (1.94 g, 6.87 mmol, 1.1 eq.). The reaction was stirred at room temperature for 2 hours. The cherry-coloured solution was then concentrated under reduced pressure. The title product was obtained after column chromatography (pentane/Et_2O = 95/5) as a colourless oil (1.73 g) with 95% yield.[18] ^{1}H NMR (400 MHz, $CDCl_3$) δ (ppm) 7.37 (dd, J = 7.3, 1.6 Hz, 1H), 7.34-7.25 (m, 2H), 7.22 (dd, J = 7.3, 1.6 Hz, 1H), 5.96 (t, J = 5.2 Hz, 1H), 3.06-2.97 (m, 1H), 2.73-2.61 (m, 1H), 2.35-2.27 (m, 1H), 1.28 (d, J = 7.0 Hz, 3H). ^{13}C NMR (101 MHz, $CDCl_3$) δ (ppm) 145.8, 141.4, 129.6, 127.9, 126.9, 126.7, 121.5, 118.8, (q, J = 320 Hz), 116.5, 31.6, 30.2, 20.2. ^{19}F NMR (376 MHz, $CDCl_3$) δ (ppm) -73.71 (s, 3F). This substrate was previously described and characterized.[20]

2.4. Synthesis of vinyl nonaflates.

2.4.1. General procedure for the synthesis of vinyl nonaflates.

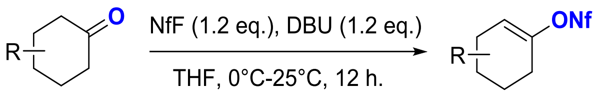

To a solution of the starting ketone (1.0 eq.) in THF (8 ml/mmol of ketone), was added DBU (1.2 eq.) and NfF (1.2 eq.) at 0°C. The mixture was stirred vigorously and let to warm at room temperature during 12 hours. After a TLC control, the mixture was washed with saturated $NaHCO_3$, and the obtained organic layer was dried with Na_2SO_4, filtered and concentrated under reduced pressure. The obtained residue was purified by flash chromatography.[1]

2.4.2. Characterization data of vinyl nonaflates.

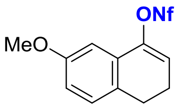

7-methoxy-3,4-dihydronaphthalen-1-yl 1,1,2,2,3,3,4,4,4-nonafluorobutane-1-sulfonate: The title product was obtained after column chromatography (hexane/Et_2O = 9/1) to give a colourless oil with 91% yield. ^{1}H NMR (400 MHz, $CDCl_3$) δ (ppm) 7.09 (d, J = 8.3 Hz, 1H), 6.93 (d, J = 2.6 Hz, 1H), 6.80 (dd, J = 8.3, 2.6 Hz, 1H), 6.05 (t, J = 4.8, 1H), 3.81 (s, 3H), 2.79 (d, J = 8.2 Hz, 2H), 2.50 (dt, J = 7.9, 4.0 Hz, 2H). ^{13}C NMR (101 MHz, $CDCl_3$) δ (ppm) 158.8, 146.6, 129.8, 128.8, 128.3, 118.5, 114.7, 107.3, 55.5, 26.1, 22.9. ^{19}F NMR (376 MHz, $CDCl_3$) δ (ppm) -80.69 (t, J = 9.7 Hz, 3F), -109.61 to -109.86 (m, 2F), -120.77 to -121.01 (m, 2F), -125.80 to -126.01 (m, 2F). HRMS (ESI) calculated for $C_{15}H_{12}F_9O_4S$: 459.0307, found 459.0304.

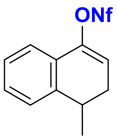

4-methyl-3,4-dihydronaphthalen-1-yl 1,1,2,2,3,3,4,4,4-nonafluorobutane-1-sulfonate. The title product was obtained after column chromatography (hexane/Et_2O = 9/1) to give a colourless oil with 97% yield. ^{1}H NMR (600 MHz, $CDCl_3$) δ (ppm) 7.39 (dd, J = 7.6, 7.6, 1H), 7.33-7.25 (m, 2H), 7.22 (dd, J = 7.3, 1.6 Hz, 1H), 5.97 (t, J = 4.8 Hz, 1H), 3.05-2.99 (m, 1H), 2.70-2.65 (m, 1H), 2.34-2.29 (m, 1H), 1.28 (d, J = 7.0 Hz, 3H). ^{13}C NMR (151 MHz, $CDCl_3$) δ (ppm) 146.1, 141.4, 129.6, 128.0, 126.9, 126.7, 121.6, 116.5, 31.6, 30.2, 20.2. ^{19}F NMR (564 MHz, $CDCl_3$) δ (ppm) -80.68 (s, 3F), -109.63 to -109.75 (m, 2F), -120.80 to -120.94 (m, 2F), -125.81 to -125.96 (m, 2F). HRMS (ESI) calculated for $C_{15}H_{11}F_9O_3SNa$: 465.01774, found 465.0177.

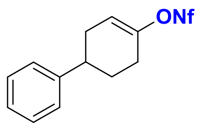

1,2,3,6-tetrahydro-[1,1'-biphenyl]-4-yl 1,1,2,2,3,3,4,4,4-nonafluorobutane-1-sulfonate. The title product was obtained after column chromatography (hexane/Et_2O = 95/5) to give a colourless oil with 57% yield. ^1H NMR (400 MHz, $CDCl_3$) δ (ppm) 7.35-7.32 (m, 2H), 7.27-7.22 (m, 3H), 5.89-5.87 (m, 1H), 2.90-2.83 (m, 1H), 2.60-2.31 (m, 4H), 2.11-2.05 (m, 1H), 2.03-1.92 (m, 1H). ^{13}C NMR (101 MHz, $CDCl_3$) δ (ppm) 149.3, 144.7, 128.8, 126.9, 126.8, 118.3, 38.9, 31.8, 29.9, 28.1. ^{19}F NMR (376 MHz, $CDCl_3$) δ (ppm) -80.70 (t, J = 9.8 Hz, 3F), -110.01 to -110.14 (m, 2F), -120.97 to -121.08 (m, 2F), -125.82 to -125.99 (m, 2F). This substrate was previously described and characterized.[1]

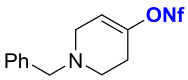

1-benzyl-1,2,3,6-tetrahydropyridin-4-yl 1,1,2,2,3,3,4,4,4-nonafluorobutane-1-sulfonate. The title product was obtained after column chromatography (hexane/Et_2O = 85/15) to give a colourless oil with 76% yield. ^1H NMR (400 MHz, $CDCl_3$) δ (ppm) 7.34-7.27 (m, 5H), 5.75-5.73 (m, 1H), 3.64 (2H), 3.15-3.13 (m, 2H), 2.73 (t, J = 5.7 Hz, 2H), 2.47-2.44 (m, 2H). ^{13}C NMR (151 MHz, $CDCl_3$) δ (ppm) 147.6, 137.8, 129.1, 128.6, 127.6, 116.4, 61.4, 50.7, 49.2, 28.5. ^{19}F NMR (376 MHz, $CDCl_3$) δ (ppm) -80.68 (t, J = 9.7 Hz, 3F), -109.91 to -110.12 (m, 2F), -120.89 to -121.25 (m, 2F), -125.73 to -126.03 (m, 2F). HRMS (ESI) calculated for $C_{16}H_{15}O_3NF_9S$: 472.0623, found 472.0618.

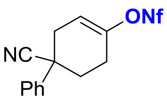

1-cyano-1,2,3,6-tetrahydro-[1,1'-biphenyl]-4-yl 1,1,2,2,3,3,4,4,4-nonafluorobutane-1-sulfonate. The title product was obtained after column chromatography (hexane/Et_2O = 85/15) to give a colourless oil with 99 % yield. ^1H NMR (400 MHz, $CDCl_3$) δ (ppm) 7.50-7.47 (m, 2H), 7.45-7.41 (m, 2H), 7.40-7.35 (m, 1H), 5.91-5.90 (m, 1H), 2.89-2.83 (m, 3H) 2.51-2.44 (m, 1H), 2.39-2.29 (m, 2H). ^{13}C NMR (151 MHz, $CDCl_3$) δ (ppm) 148.4, 138.3, 129.4, 128.8, 125.7, 121.5, 115.4, 40.2, 35.8, 33.0, 26.0. ^{19}F NMR (376 MHz, $CDCl_3$) δ (ppm) -80.68 (t, J = 9.7 Hz, 3F), -109.61 to -109.76 (m, 2F), -120.86 to -121.02 (m, 2F), -125.81 to -125.93 (m, 2F). HRMS (ESI) calculated for $C_{17}H_{12}O_3NF_9SNa$: 504.0286, found 504.0281.

2.5. Nickel-catalyzed trifluoromethylthiolation of aryl-LG

2.5.1. General procedure for nickel-catalyzed trifluoromethylthiolation of aryl-LG

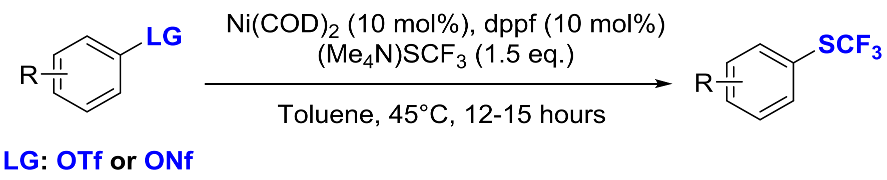

Under nitrogen atmosphere, a solution of Ni(COD)$_2$ (11.1 mg, 0.04 mmol, 10 mol%), dppf (22.2 mg, 0.04 mmol, 10 mol%) and (Me$_4$N)SCF$_3$ (104 mg, 0.6 mmol, 1.5 eq.) in dry toluene (2 mL) was stirred at room temperature for 5 min, then Ar-LG (0.4 mmol) was added. The suspension was stirred at 45°C for 12-15 h. The mixture was filtered. The solvent was removed under reduced pressure, and the residue was directly subjected to purification flash column chromatography to give the corresponding product.

2.5.2. Characterization data of aryl-SCF$_3$

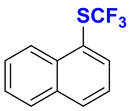

Naphthalen-1-yl(trifluoromethyl)sulfane (2): The title product was obtained after column chromatography (hexane) as a colourless oil with 94% yield. ^{1}H NMR (400 MHz, CDCl$_3$) δ (ppm) 8.56 (d, *J* = 8.5 Hz, 1H), 8.01 (dd, *J* = 14.8, 7.7 Hz, 2H), 7.91 (d, *J* = 8.1 Hz, 1H), 7.69-7.65 (m, 1H), 7.61-7.57 (m, 1H), 7.52 (dd, *J* = 8.2, 7.2 Hz, 1H). ^{13}C NMR (101 MHz, CDCl$_3$) δ (ppm) 138.0, 135.5, 134.4, 132.5, 129.8 (q, *J* = 309 Hz), 128.7, 127.8, 126.9, 126.0, 125.7, 121.8. ^{19}F NMR (376 MHz, CDCl$_3$) δ (ppm) -42.24 (s, 3F). These data are in agreement with those reported previously in the literature.[21]

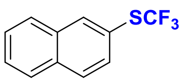

Naphthalen-2-yl(trifluoromethyl)sulfane (3): The title product was obtained after column chromatography (hexane) as a colourless oil with 81% yield. ^{1}H NMR (400 MHz, CD$_2$Cl$_2$) δ (ppm) 8.24 (d, *J* = 1.1 Hz, 1H), 7.93-7.90 (m, 3H), 7.68 (dd, *J* = 8.7, 1.6 Hz, 1H), 7.64-7.57 (m, 2H). ^{13}C NMR (101 MHz, CD$_2$Cl$_2$) δ (ppm) 137.6, 134.5, 134.0, 132.3, 130.9 (q, *J* = 308 Hz), 129.8, 128.7, 128.6, 128.3, 127.6, 121.9. ^{19}F NMR (376 MHz, CD$_2$Cl$_2$) δ (ppm) -42.67 (s, 3F). These data are in agreement with those reported previously in the literature.[22]

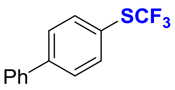

[1,1'-biphenyl]-4-yl(trifluoromethyl)sulfane (4): The title product was obtained after column chromatography (hexane) as a colourless oil with 80% yield. ^{1}H NMR (400 MHz, CDCl$_3$) δ (ppm) 7.75 (d, *J* = 8.3 Hz, 2H), 7.66 (d, *J* = 8.4, 2H), 7.62 (d, *J* = 7.2 Hz, 2H), 7.50 (dd, *J* = 7.2, 7.2 Hz, 2H), 7.43

(dd, J = 7.2, 7.2 Hz, 1H). ^{13}C NMR (101 MHz, $CDCl_3$) δ (ppm) 144.0, 139.8, 136.9, 129.8 (q, J = 308 Hz), 129.1, 128.3, 128.3, 127.3, 123.2. ^{19}F NMR (376 MHz, $CDCl_3$) δ (ppm) -42.73 (s, 3F). These data are in agreement with those reported previously in the literature.[22]

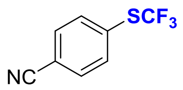

4-((trifluoromethyl)thio)benzonitrile (5): The title product was obtained after column chromatography (pentane/Et_2O = 95/5) as a yellowish oil with 86% yield. ^{1}H NMR (600 MHz, $CDCl_3$) δ 7.77 (d, J = 8.3 Hz, 2H), 7.72 (d, J = 8.4 Hz, 2H). ^{13}C NMR (151 MHz, $CDCl_3$) δ (ppm) 136.2, 133.1, 130.7, 129.2 (q, J = 308 Hz), 117.8, 114.8. ^{19}F NMR (564 MHz, $CDCl_3$) δ -41.52 (s, 3F). These data are in agreement with those reported previously in the literature.[23]

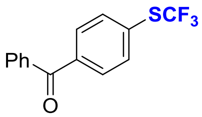

Phenyl(4-((trifluoromethyl)thio)phenyl)methanone (6): The title product was obtained after column chromatography (pentane/Et_2O = 95/5) as a colourless liquid with 80% yield. ^{1}H NMR (400 MHz, $CDCl_3$) δ 7.84-7.80 (m, 4H), 7.77 (d, J = 8.2 Hz, 2H), 7.63 (dd, J = 7.4, 7.4 Hz, 1H), 7.51 (dd, J = 7.8, 7.8 Hz, 2H). ^{13}C NMR (101 MHz, $CDCl_3$) δ (ppm) 195.7, 139.6, 136.9, 135.7, 133.1, 130.8, 130.2, 129.5 (q, J = 308 Hz), 129.3, 128.7. ^{19}F NMR (376 MHz, $CDCl_3$) δ -41.81 (s, 3F). These data are in agreement with those reported previously in the literature.[23]

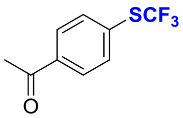

1-(4-((trifluoromethyl)thio)phenyl)ethan-1-one (7): The title product was obtained after column chromatography (pentane/Et_2O = 95/5) as a colourless liquid with 90% yield. ^{1}H NMR (400 MHz, $CDCl_3$) δ 7.98 (d, J = 6.9 Hz, 2H), 7.74 (d, J = 7.0 Hz, 2H), 2.62 (s, 3H). ^{13}C NMR (101 MHz, $CDCl_3$) δ (ppm) 197.2, 138.6, 135.9, 130.2, 129.2, 129.2 (q, J = 308 Hz), 26.9. ^{19}F NMR (376 MHz, $CDCl_3$) δ -41.81 (s, 3F). These data are in agreement with those reported previously in the literature.[23]

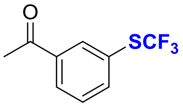

1-(3-((trifluoromethyl)thio)phenyl)ethan-1-one (8): The title product was obtained after column chromatography (pentane) as a yellowish oil with 90% yield. ^{1}H NMR (400 MHz, $CDCl_3$) δ 8.23 (s, 1H), 8.07 (d, J = 7.8 Hz, 1H), 7.85 (d, J = 7.7 Hz, 1H), 7.55 (dd, J = 7.8, 7.8 Hz, 1H), 2.63 (s, 3H). ^{13}C NMR (101 MHz, $CDCl_3$) δ 196.7, 140.6, 138.4, 136.2, 130.7, 130.7 (q, J = 308 Hz), 130.0, 125.6, 26.8. ^{19}F NMR (376 MHz, $CDCl_3$) δ -42.47 (s, 3F). These data are in agreement with those reported previously in the literature.[23]

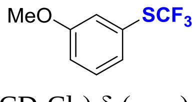

(3-methoxyphenyl)(trifluoromethyl)sulfane (9): The title product was obtained after column chromatography (pentane) as a colourless oil with 80% yield. ^{1}H NMR (600 MHz, CD_2Cl_2) δ (ppm) 7.36 (dd, J = 7.9, 7.9 Hz, 1H), 7.26-7.24 (m, 1H), 7.19 (dd, J = 2.2, 2.2 Hz, 1H), 7.05 (ddd, J =

8.0, 2.2, 1.0 Hz, 1H), 3.83 (s, 3H). ^{13}C NMR (101 MHz, CD_2Cl_2) δ (ppm) 160.6, 130.8, 128.9, 128.8 (q, *J* = 308 Hz), 125.6, 121.8, 117.4, 56.1. ^{19}F NMR (564 MHz, CD_2Cl_2) δ (ppm) -43.12 (s, 3F). These data are in agreement with those reported previously in the literature.[24]

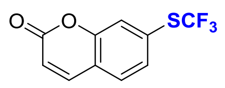

7-((trifluoromethyl)thio)-2H-chromen-2-one (10): The title product was obtained after column chromatography (hexane/Et_2O = 25/1) as white solid with 89% yield. M.p. 78 - 79 °C. ^{1}H NMR (400 MHz, $CDCl_3$) δ (ppm) 7.72 (d, *J* = 9.6 Hz, 1H), 7.63 (s, 1H), 7.54-7.54 (m, 2H), 6.52 (d, *J* = 9.6 Hz, 1H). ^{13}C NMR (151 MHz, $CDCl_3$) δ (ppm) 159.7, 153.9, 142.4, 131.3, 129.3 (q, *J* = 309 Hz), 128.6, 128.3, 124.0, 120.6, 118.8. ^{19}F NMR (376 MHz, $CDCl_3$) δ (ppm) -41.78 (s, 3F). HRMS (ESI) for $C_{10}H_5O_2F_3SNa$: 268.9855, found: 268.9854.

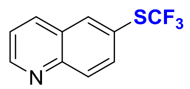

6-((trifluoromethyl)thio)quinolone (11): The title product was obtained after column chromatography (hexane/Et_2O = 15/1) as a colourless oil with 70% yield. ^{1}H NMR (600 MHz, $CDCl_3$) δ (ppm) 9.00 (d, *J* = 3.4 Hz, 1H), 8.20 - 8.17 (m, 2H), 8.14 (d, *J* = 8.8 Hz, 1H), 7.89 (d, *J* = 8.8 Hz, 1H), 7.47 (dd, *J* = 8.3, 4.1, 1H). ^{13}C NMR (151 MHz, $CDCl_3$) δ (ppm) 152.4, 148.7, 136.9, 136.3, 135.6, 131.0, 129.8 (q, *J* = 308 Hz), 128.4, 122.8, 122.2. ^{19}F NMR (564 MHz, $CDCl_3$) δ (ppm) -42.69 (s, 3F). These data are in agreement with those reported previously in the literature.[23]

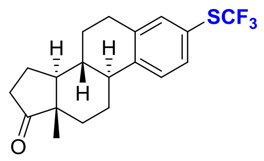

(8*R*,9*S*,13*S*,14*S*)-13-methyl-3-((trifluoromethyl)thio)-7,8,9,11,12,13,15,16-octahydro-6*H*-cyclopenta[a]phenanthren-17(14*H*)-one (12): The title product was obtained after column chromatography (hexane/Et_2O = 20/1) as colourless oil with 62%. ^{1}H NMR (600 MHz, $CDCl_3$) δ 7.41 (d, *J* = 8.2 Hz, 1H), 7.37 (s, 1H), 7.32 (d, *J* = 8.2 Hz, 1H), 2.94 - 2.92 (m, 2H), 2.51 (dd, *J* = 19.0, 8.9 Hz, 1H), 2.42 - 2.39 (m, 1H), 2.33 - 2.29 (m, 1H), 2.18 - 2.11 (m, 1H), 2.09 – 2.03 (m, 2H), 1.98 (dd, *J* = 12.6, 3.3 Hz, 1H), 1.67 - 1.59 (m, 2H), 1.54 - 1.44 (m, 4H), 0.91 (s, 3H). ^{13}C NMR (151 MHz, $CDCl_3$) δ 220.6, 143.1, 138.2, 136.8, 133.6, 129.8 (q, *J* = 308.0 Hz), 126.7, 121.2, 50.5, 48.0, 44.4, 37.8, 35.9, 31.6, 29.2, 26.3, 25.6, 21.6, 13.9. ^{19}F NMR (564 MHz, $CDCl_3$) δ -42.88 (s, 3F). HRMS (ESI) for $C_{19}H_{21}OF_3SNa$: 377.1157, Found 377.1157.

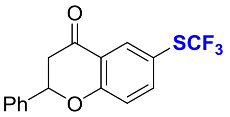

2-phenyl-6-((trifluoromethyl)thio)chroman-4-one (13): The title product was obtained after column chromatography (hexane/Et_2O = 20/1) as white solid with 96% yield. M.p. 280 °C (decomp). ^{1}H NMR (600 MHz, $CDCl_3$) δ (ppm) 8.24 (d, *J* = 2.0 Hz, 1H), 7.76 (dd, *J* = 8.6, 2.0 Hz, 1H), 7.49 -7.41 (m, 5H), 7.12 (d, *J* = 8.6 Hz, 1H), 5.54 (dd, *J* = 13.4, 2.7 Hz, 1H), 3.12 (dd, *J* = 16.9, 13.4 Hz, 1H), 2.95 (dd, *J* = 16.9, 2.9 Hz, 1H). ^{13}C NMR (151 MHz, $CDCl_3$) δ (ppm) 190.7, 163.4, 143.7,

138.1, 136.1, 129.5 (q, J = 308 Hz), 129.3, 129.1, 126.3, 121.7, 120.0, 117.2, 80.2, 44.3. ^{19}F NMR (564 MHz, $CDCl_3$) δ (ppm) - 43.32 (s, 3F). HRMS (ESI) for $C_{16}H_{12}O_2F_3S$: 325.0505, Found: 325.0509.

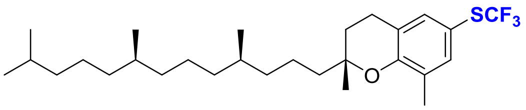

(*R*)-2,8-dimethyl-6-((trifluoromethyl)thio)-2-((4*R*,8*R*)-4,8,12-trimethyltridecyl)chroman (14): The title product was obtained after column chromatography (hexane/Et_2O = 25/1) as colourless oil with 90% yield. ^{1}H NMR (600 MHz, $CDCl_3$) δ 7.22 (s, 1H), 7.20 (s, 1H), 2.80 - 2.72 (m, 2H), 2.16 (s, 3H), 1.86 - 1.81 (m, 1H), 1.78 - 1.74 (m, 1H), 1.61 – 1.02 (m, 24H), 0.88 - 0.84 (m, 12H). ^{13}C NMR (151 MHz, $CDCl_3$) δ 154.9, 136.6, 135.8, 129.9 (q, J = 308 Hz) 127.9, 121.7, 112.3, 77.2, 40.4, 39.5, 37.6, 37.5, 37.5, 37.4, 33.0, 32.8, 30.9, 28.1, 25.0, 24.6, 24.4, 22.9, 22.8, 22.2, 21.1, 19.9, 19.8, 16.1. ^{19}F NMR (564 MHz, $CDCl_3$) δ -43.99 (s, 3F). HRMS (ESI) for $C_{28}H_{46}OF_3S$: 487.3216, Found: 487.3214.

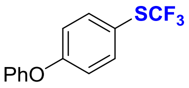

(4-phenoxyphenyl)(trifluoromethyl)sulfane (22): The catalysis was performed under the above mentioned conditions with addition of MeCN (16.4 mg, 0.4 mmol). The title product was obtained after column chromatography (pentane/Et_2O = 95/5) as a colourless oil with 83% yield. ^{1}H NMR (400 MHz, $CDCl_3$) δ (ppm) 7.59 (d, J = 8.7 Hz, 2H), 7.39 (dd, J = 8.5, 8.5 Hz, 2H), 7.20 (dd, J = 7.2, 7.2 Hz, 1H), 7.07 (d, J = 8.5 Hz, 2H), 7.00 (d, J = 8.9 Hz, 2H). ^{13}C NMR (101 MHz, $CDCl_3$) δ (ppm) 160.5, 155.7, 138.5, 130.2, 129.7 (q, J = 308 Hz), 124.7, 120.2, 118.8, 117.4. ^{19}F NMR (376 MHz, $CDCl_3$) δ (ppm) -43.58 (s, 3F). These data are in agreement with those reported previously in the literature.[25]

2.6. Nickel-catalyzed trifluoromethylthiolation of vinyl-OTf/ONf

2.6.1. General procedure for nickel-catalyzed trifluoromethylthiolation of vinyl-OTf/ONf

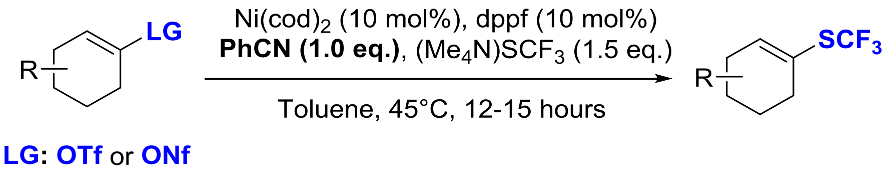

Under nitrogen atmosphere, a solution of $Ni(COD)_2$ (5.5 mg, 0.02 mmol, 10 mol%), dppf (11.1 mg, 0.02 mmol, 10 mol%) and $(Me_4N)SCF_3$ (52 mg, 0.3 mmol, 1.5 eq.) in dry toluene (1 mL) was stirred at room temperature for 5 min, then vinyl-LG (0.2 mmol, 1 eq.) was added. The suspension was stirred at 45 ^{o}C for 12-15 h. The mixture was filtered. The solvent was removed under reduced pressure, and the residue was directly subjected to purification flash column chromatography to give the corresponding product.

2.6.2. Characterization data of vinyl-SCF_3

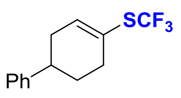

(1,2,3,6-tetrahydro-[1,1'-biphenyl]-4-yl)(trifluoromethyl)sulfane (16): The title product was obtained after preparative TLC (pentane) as a colourless oil with 81% yield. ^{1}H NMR (600 MHz, CD_2Cl_2) δ (ppm) 7.32-7.29 (m, 2H), 7.23-7.19 (m, 3H), 6.51-6.49 (m, 1H), 2.86-2.81 (m, 1H), 2.59-2.45 (m, 3H), 2.36-2.30 (m, 1H), 2.05-2.01 (m, 1H), 1.93-1.87 (m, 1H). ^{13}C NMR (151 MHz, CD_2Cl_2) δ (ppm) 146.2, 142.7, 130.8 (q, J = 309 Hz), 129.0, 127.3, 126.9, 124.9, 39.1, 35.6, 33.3, 31.2. ^{19}F NMR (564 MHz, CD_2Cl_2) δ (ppm) -41.68 (s, 3F). HRMS (ESI) calculated for $C_{13}H_{13}F_3S$: 258.0685, found 258.0686.

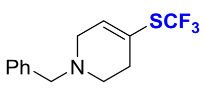

1-benzyl-4-((trifluoromethyl)thio)-1,2,3,6-tetrahydropyridine (17): The title product was obtained after preparative TLC (toluene) as a colourless oil with 97% yield. ^{1}H NMR (400 MHz, $CDCl_3$) δ (ppm) 7.34-7.26 (m, 5H), 6.32-6.30 (m, 1H), 3.60 (s, 2H), 3.12-3.10 (m, 2H), 2.67 (t, J = 5.6 Hz, 2H), 2.50-2.47 (m, 2H). ^{13}C NMR (101 MHz, $CDCl_3$) δ (ppm) 139.4, 137.9, 130.0 (q, J = 308 Hz), 129.2, 128.5, 127.4, 123.1, 62.2, 53.8, 50.0, 32.9. ^{19}F NMR (376 MHz, $CDCl_3$) δ (ppm) -40.88 (s, 3F). HRMS (ESI) calculated for $C_{13}H_{15}F_3SN$: 274.0872, found: 274.0873.

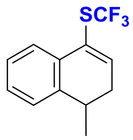

(4-methyl-3,4-dihydronaphthalen-1-yl)(trifluoromethyl)sulfane (18'): The title product was obtained after preparative TLC (pentane) as a colourless oil with 81% yield. ^{1}H NMR (600 MHz, $CDCl_3$) δ (ppm) 7.75-7.73 (m, 1H), 7.27-7.23 (m, 2H), 7.18-7.17 (m, 1H), 6.76 (t, *J* = 4.7 Hz, 1H), 3.01-2.95 (m, 1H), 2.63 (ddd, *J* = 17.5, 6.8, 4.0 Hz, 1H), 2.31 (ddd, *J* = 17.5, 5.9, 5.9 Hz; 1H), 1.24 (d, *J* = 7.1 Hz, 3H). ^{13}C NMR (151 MHz, $CDCl_3$) δ (ppm) 144.3, 141.1, 132.4, 128.7 (q, *J* = 308 Hz), 128.7, 126.8, 126.5, 125.7, 123.6, 32.9, 31.7, 20.3. ^{19}F NMR (564 MHz, $CDCl_3$) δ (ppm) -42.30 (s, 3F). HRMS (ESI) calculated for $C_{12}H_{11}F_3S$: 244.0528, found: 244.0522.

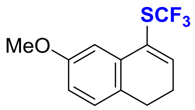

(7-methoxy-3,4-dihydronaphthalen-1-yl)(trifluoromethyl)sulfane (19'): The title product was obtained after column chromatography (pentane) as a colourless oil with 70% yield. ^{1}H NMR (400 MHz, CD_2Cl_2) δ (ppm) 7.29 (d, *J* = 2.6 Hz, 1H), 7.08 (d, *J* = 8.2 Hz, 1H), 6.88 (t, *J* = 4.7 Hz, 1H), 6.76 (dd, *J* = 8.2, 2.7 Hz, 1H), 3.80 (s, 3H), 2.78 (t, *J* = 8.1 Hz, 2H), 2.46 (dt, *J* = 8.1, 4.8 Hz, 2H). ^{13}C NMR (101 MHz, CD_2Cl_2) δ (ppm) 159.2, 147.2, 134.7, 130.3 (q, *J* = 308 Hz), 129.0, 128.6, 124.2, 113.7, 112.0, 55.9, 26.7, 26.1. ^{19}F NMR (376 MHz, CD_2Cl_2) δ (ppm) -42.89 (s, 3F). HRMS (ESI) calculated for $C_{12}H_{11}OF_3S$: 260.0477, found: 260.0472.

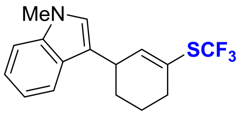

1-methyl-3-(3-((trifluoromethyl)thio)cyclohex-2-en-1-yl)-1H-indole (20): The title product was obtained after preparative TLC (pentane/Et_2O = 400/1) as a yellow oil with 76% yield. ^{1}H NMR (600 MHz, $CDCl_3$) δ 7.59 (d, *J* = 7.9 Hz, 1H), 7.31 (d, *J* = 8.2 Hz, 1H), 7.28-7.21 (m, 1H), 7.16 – 7.08 (m, 1H), 6.77 (s, 1H), 6.60-6.54 (m, 1H), 3.94-3.85 (m, 1H), 3.76 (s, 3H), 2.54-2.40 (m, 3H), 2.09-1.98 (m, 1H), 1.91-1.69 (m, 3H). ^{13}C NMR (151 MHz, $CDCl_3$) δ 145.2, 137.4, 130.5 (q, *J* = 309 Hz), 126.8, 126.6, 125.2, 121.9, 119.1, 119.1, 117.2, 109.6, 35.0, 32.9, 32.6, 28.9, 21.8. ^{19}F NMR (376 MHz, $CDCl_3$) δ (ppm) -40.98. HRMS (ESI) calculated for $C_{16}H_{17}NF_3S$: 312.1028, found: 312.1026.

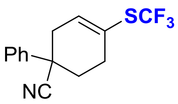

4-((trifluoromethyl)thio)-3,6-dihydro-[1,1'-biphenyl]-1(2H)-carbonitrile (21): The title product was obtained after column chromatography (pentane) as a colourless oil with 96% yield. ^{1}H NMR (400 MHz, CD_2Cl_2) δ (ppm) 7.50-7.49 (m, 2H), 7.48-7.45 (m, 2H), 7.41-7.25 (m, 1H), 6.50-6.48 (m, 1H), 2.89-2.72 (m, 3H), 2.59-2.50 (m, 1H), 2.35-2.22 (m, 2H). ^{13}C NMR (101 MHz, CD_2Cl_2) δ (ppm) 139.7, 138.8, 130.5 (q, *J* = 308 Hz), 129.7, 128.9, 126.2, 125.5, 122.4, 40.3, 39.1, 34.1, 30.9. ^{19}F NMR (564 MHz, CD_2Cl_2) δ (ppm) -41.34 (s, 3F). HRMS (ESI) calculated for $C_{14}H_{12}NF_3SNa$: 306.0535, found: 306.0535.

3. Computational details

All calculations were performed using Gaussian 09 software.[26] Structural optimizations and frequency calculations were performed with B3LYP functional along with 6-31G(d) basis set and the LANL2DZ ECP on Ni and Fe atoms. Single point energy calculations were performed with M06L functional and 6-311++G(d,p) basis set and LANL2DZ ECP on Ni and Fe atoms. Solvent effects of toluene were included in the single point calculations using the CPCM solvation model. Frequency calculations were performed to confirm whether the structure is a minimum or a transition state.

[(dppf)Ni$^{(0)}$(cod)]

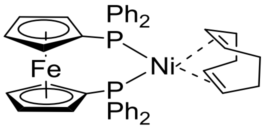

Ni	0.001151000	-1.365258000	-0.000714000
Fe	-0.001573000	2.915930000	-0.007888000
P	-1.813089000	0.004550000	0.041390000
P	1.812956000	0.007297000	-0.040186000
C	-1.591777000	1.751670000	0.596172000
C	1.590460000	1.750946000	-0.604338000
C	3.299082000	-0.489676000	-1.052528000
C	4.214974000	-1.432179000	-0.552330000
H	4.101164000	-1.812646000	0.458952000
C	5.283832000	-1.879148000	-1.330272000
H	5.983609000	-2.601412000	-0.917169000
C	5.454943000	-1.402132000	-2.631711000
H	6.287146000	-1.749713000	-3.238013000
C	4.548339000	-0.473565000	-3.144608000
H	4.672892000	-0.089989000	-4.154223000
C	3.481987000	-0.021975000	-2.363171000
H	2.798094000	0.712953000	-2.776302000
C	2.631050000	0.312369000	1.613145000
C	3.923048000	0.851643000	1.737195000
H	4.501935000	1.076951000	0.846615000
C	4.479394000	1.097586000	2.993743000
H	5.480370000	1.515382000	3.066852000
C	3.755693000	0.806377000	4.153025000
H	4.190977000	0.994939000	5.130901000
C	2.474588000	0.263937000	4.046547000
H	1.905868000	0.024598000	4.941470000
C	1.921740000	0.017833000	2.786761000
H	0.928906000	-0.415289000	2.705289000
C	0.795352000	2.137817000	-1.739904000
H	0.243273000	1.464429000	-2.378328000
C	0.788588000	3.559740000	-1.831511000
H	0.241294000	4.144684000	-2.559404000
C	1.573389000	4.072205000	-0.757161000
H	1.727984000	5.117824000	-0.522985000
C	2.071328000	2.967725000	-0.006512000
H	2.665212000	3.034033000	0.893606000
C	-0.796786000	2.146875000	1.728911000
H	-0.243109000	1.478486000	2.371201000
C	-0.792497000	3.569377000	1.811755000
H	-0.245953000	4.159697000	2.535865000
C	-1.578500000	4.073867000	0.734512000
H	-1.734936000	5.117753000	0.493919000
C	-2.074825000	2.963912000	-0.009057000
H	-2.669538000	3.023425000	-0.909107000
C	-2.640525000	0.302383000	-1.608714000

C	-3.935976000	0.834441000	-1.727715000
H	-4.512145000	1.057649000	-0.834852000
C	-4.499013000	1.076301000	-2.982078000
H	-5.502612000	1.488443000	-3.051197000
C	-3.778588000	0.788449000	-4.144228000
H	-4.219052000	0.973856000	-5.120385000
C	-2.493911000	0.253571000	-4.042762000
H	-1.927482000	0.017084000	-4.939893000
C	-1.934435000	0.011440000	-2.785157000
H	-0.938772000	-0.415792000	-2.707745000
C	-3.292759000	-0.491338000	1.063820000
C	-4.207669000	-1.439768000	0.573094000
H	-4.097209000	-1.825288000	-0.436639000
C	-5.271634000	-1.885868000	1.358213000
H	-5.970805000	-2.612752000	0.952250000
C	-5.438646000	-1.402046000	2.657663000
H	-6.266999000	-1.748964000	3.269593000
C	-4.532989000	-0.467484000	3.161214000
H	-4.654441000	-0.078493000	4.169135000
C	-3.471634000	-0.016706000	2.372512000
H	-2.788700000	0.723092000	2.778413000
C	0.432929000	-2.804729000	-1.598936000
H	0.690933000	-2.179618000	-2.453671000
C	-0.902241000	-2.940574000	-1.275598000
H	-1.622886000	-2.409859000	-1.889839000
C	-1.466693000	-4.059216000	-0.411398000
H	-0.880699000	-4.971631000	-0.569754000
H	-2.480661000	-4.297801000	-0.757401000
C	-1.531203000	-3.725359000	1.102743000
H	-2.486616000	-3.238684000	1.315210000
H	-1.535678000	-4.664638000	1.681798000
C	-0.425468000	-2.811084000	1.590309000
H	-0.686117000	-2.191069000	2.447954000
C	0.910680000	-2.939769000	1.266835000
H	1.628488000	-2.408752000	1.884207000
C	1.480839000	-4.053072000	0.399365000
H	0.900106000	-4.969165000	0.555875000
H	2.496331000	-4.286814000	0.744134000
C	1.542827000	-3.715408000	-1.114154000
H	2.496127000	-3.224482000	-1.326113000
H	1.550613000	-4.653326000	-1.695342000

```
Zero-point correction=                           0.700959 (Hartree/Particle)
 Thermal correction to Energy=                    0.742647
 Thermal correction to Enthalpy=                  0.743591
 Thermal correction to Gibbs Free Energy=         0.626281
 Sum of electronic and zero-point Energies=          -2599.270508
 Sum of electronic and thermal Energies=             -2599.228819
 Sum of electronic and thermal Enthalpies=           -2599.227875
 Sum of electronic and thermal Free Energies=        -2599.345185
```

CPCM (Toluene) M06L/6-311++G(d,p) with LANL2DZ (for Ni, Fe) E = -2600.3670714

Cod

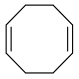

C	-1.083205000	-1.107768000	0.665235000
C	1.923352000	0.011316000	-0.024461000
C	0.007978000	1.704749000	-0.218086000
C	-1.923415000	-0.010876000	-0.024290000
C	-1.215227000	1.236097000	-0.495433000
H	-1.771795000	-1.910180000	0.958605000

H	-2.741047000	0.283487000	0.653768000
H	-0.666355000	-0.724888000	1.600205000
H	0.291717000	2.634824000	-0.711800000
H	-2.425665000	-0.455621000	-0.896380000
C	-0.007776000	-1.704827000	-0.218204000
H	-0.290785000	-2.635346000	-0.711489000
C	1.215478000	-1.236011000	-0.495150000
H	1.822532000	-1.841673000	-1.170423000
H	2.424611000	0.456531000	-0.896905000
H	2.741756000	-0.282836000	0.652758000
H	-1.821805000	1.841441000	-1.171403000
C	1.082851000	1.107462000	0.665827000
H	0.665549000	0.723609000	1.600171000
H	1.771066000	1.909803000	0.960263000

```
Zero-point correction=                           0.181195 (Hartree/Particle)
 Thermal correction to Energy=                    0.188619
 Thermal correction to Enthalpy=                  0.189563
 Thermal correction to Gibbs Free Energy=         0.149669
 Sum of electronic and zero-point Energies=           -311.843270
 Sum of electronic and thermal Energies=              -311.835846
 Sum of electronic and thermal Enthalpies=            -311.834902
 Sum of electronic and thermal Free Energies=         -311.874796

CPCM (Toluene) M06L/6-311++G(d,p) E = -312.06357723
```

TS-oa-Ph-SCF$_3$

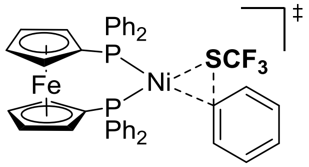

C	-0.596649000	2.596529000	-0.772316000
C	-1.926559000	2.808551000	-1.181757000
H	-2.745636000	2.634300000	-0.496551000
C	-2.195458000	3.210685000	-2.490345000
H	-3.229446000	3.339902000	-2.800070000
C	-1.158323000	3.420716000	-3.403942000
C	0.165093000	3.248536000	-2.991320000
H	0.983812000	3.437856000	-3.681586000
C	0.451503000	2.875491000	-1.675875000
H	1.482839000	2.821936000	-1.345336000
H	-1.379085000	3.737811000	-4.419700000
Ni	-0.104199000	0.968903000	0.231170000
S	0.024427000	2.893856000	1.244154000
C	-1.543865000	3.615201000	1.846848000
F	-1.355013000	3.976245000	3.132935000
F	-1.929476000	4.715217000	1.175220000
F	-2.593896000	2.756836000	1.825087000
Fe	0.783426000	-2.671858000	-1.556539000
P	-1.628775000	-0.751050000	0.088318000
P	2.021267000	0.075481000	0.098359000
C	-3.123668000	-0.541218000	-0.989615000
C	-2.343498000	-1.269353000	1.720501000
C	-2.923907000	-0.203853000	-2.339707000
H	-1.915492000	-0.063726000	-2.718603000
C	2.135079000	-1.131877000	-1.271348000
C	1.768396000	-1.097867000	2.635079000
H	0.748643000	-0.726635000	2.577434000
C	3.509380000	-2.254296000	3.853263000
H	3.843401000	-2.794678000	4.735097000
C	-1.003259000	-2.386437000	-0.513623000
C	-3.456340000	-2.881740000	3.163185000
H	-3.910073000	-3.859982000	3.300085000
C	-4.438796000	-0.655350000	-0.515950000

H	-4.623180000	-0.898916000	0.524931000
C	3.460374000	1.196801000	-0.240752000
C	-3.393815000	-1.981821000	4.231013000
H	-3.797687000	-2.258909000	5.201319000
C	-5.313322000	-0.158032000	-2.720073000
H	-6.158556000	-0.013100000	-3.387648000
C	2.649339000	-0.855948000	1.570848000
C	3.977073000	-1.302504000	1.677046000
H	4.684467000	-1.089246000	0.880461000
C	1.429454000	-0.953051000	-2.513159000
H	0.792749000	-0.110265000	-2.749236000
C	2.193689000	-1.796906000	3.768043000
H	1.497961000	-1.974850000	4.583726000
C	-2.286592000	-0.374800000	2.799257000
H	-1.838393000	0.602718000	2.658618000
C	-2.809930000	-0.728477000	4.046153000
H	-2.757439000	-0.022163000	4.870518000
C	-5.523947000	-0.460309000	-1.374567000
H	-6.535652000	-0.550717000	-0.987477000
C	2.827567000	-2.387901000	-1.360122000
H	3.412879000	-2.841009000	-0.572289000
C	-0.533363000	-4.271050000	-1.788465000
H	-0.497870000	-4.967593000	-2.616183000
C	-2.935160000	-2.529273000	1.918317000
H	-2.987006000	-3.237611000	1.096841000
C	2.547699000	-2.967123000	-2.632466000
H	2.887813000	-3.935996000	-2.975477000
C	4.401716000	-2.001663000	2.807336000
H	5.431710000	-2.342263000	2.875966000
C	5.557423000	3.021941000	-0.686081000
H	6.366000000	3.727091000	-0.859116000
C	5.295761000	2.012527000	-1.612488000
H	5.902485000	1.925431000	-2.510360000
C	4.255919000	1.104192000	-1.392315000
H	4.067624000	0.321459000	-2.120620000
C	-4.007746000	-0.030676000	-3.200655000
H	-3.830963000	0.220549000	-4.243245000
C	4.770587000	3.123424000	0.464777000
H	4.964104000	3.908256000	1.191190000
C	-0.103778000	-3.231399000	0.224838000
H	0.307470000	-3.015301000	1.200990000
C	3.728913000	2.223194000	0.683088000
H	3.119245000	2.320332000	1.577873000
C	-1.260152000	-3.043984000	-1.762351000
H	-1.882644000	-2.661664000	-2.558629000
C	1.688571000	-2.078670000	-3.346218000
H	1.268129000	-2.252019000	-4.328344000
C	0.174515000	-4.390597000	-0.556438000
H	0.844020000	-5.194521000	-0.278965000

```
Zero-point correction=                 0.624187 (Hartree/Particle)
 Thermal correction to Energy=          0.668790
 Thermal correction to Enthalpy=         0.669734
 Thermal correction to Gibbs Free Energy=     0.541748
 Sum of electronic and zero-point Energies=     -3254.748381
 Sum of electronic and thermal Energies=       -3254.703779
 Sum of electronic and thermal Enthalpies=      -3254.702834
 Sum of electronic and thermal Free Energies=    -3254.830821
```

CPCM (Toluene) M06L/6-311++G(d,p) with LANL2DZ (for Ni, Fe) E = -3255.82653337

PhSCF$_3$

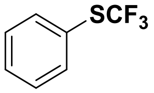

C	0.729372000	-0.209529000	-0.487894000
C	1.272085000	1.081403000	-0.530660000
H	0.679653000	1.901871000	-0.921719000
C	2.568658000	1.301258000	-0.065303000
H	2.987494000	2.303372000	-0.092778000
C	3.328515000	0.236565000	0.425638000
C	2.789931000	-1.051234000	0.457590000
H	3.379164000	-1.880601000	0.838760000
C	1.488854000	-1.277276000	0.005339000
H	1.059925000	-2.273887000	0.033876000
H	4.340604000	0.410705000	0.780556000
S	-0.919286000	-0.522166000	-1.128507000
C	-1.913848000	0.093974000	0.270862000
F	-3.207156000	-0.086284000	-0.044825000
F	-1.663543000	-0.557490000	1.418234000
F	-1.720375000	1.404022000	0.511480000

```
Zero-point correction=                    0.106571 (Hartree/Particle)
 Thermal correction to Energy=          0.116127
 Thermal correction to Enthalpy=         0.117072
 Thermal correction to Gibbs Free Energy=     0.069469
 Sum of electronic and zero-point Energies=      -967.342843
 Sum of electronic and thermal Energies=       -967.333287
 Sum of electronic and thermal Enthalpies=      -967.332342
 Sum of electronic and thermal Free Energies=     -967.379945

CPCM (Toluene) M06L/6-311++G(d,p) E = -967.557940477
```

TS-oa-Ph-OMe

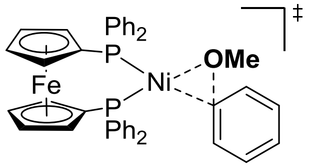

C	-1.111061000	2.801637000	-0.626691000
C	-2.416431000	3.031953000	-1.119187000
C	-0.608854000	3.648233000	0.386383000
C	-3.216476000	4.006570000	-0.538286000
C	-1.437524000	4.616070000	0.971763000
H	0.416464000	3.535860000	0.733473000
C	-2.743289000	4.801197000	0.521446000
H	-2.793862000	2.431208000	-1.939756000
H	-4.227763000	4.153038000	-0.912446000
Fe	0.684982000	-2.907359000	-0.485324000
P	1.996279000	0.228554000	-0.019785000
C	-2.898878000	-0.538986000	-1.351730000
C	-2.690045000	-0.155055000	1.538742000
C	-2.508112000	-0.165699000	-2.647209000
H	-1.526285000	0.275920000	-2.801161000
C	2.121288000	-1.446311000	-0.739126000
C	1.359330000	0.271345000	2.714280000
H	0.394886000	0.632481000	2.366421000
C	2.831737000	-0.349706000	4.531291000
H	3.013520000	-0.479739000	5.594897000
C	-1.123597000	-2.122943000	0.190303000
C	-4.045083000	-1.024871000	3.364358000
H	-4.484243000	-1.873891000	3.882214000
C	-4.190108000	-1.051668000	-1.156138000

H	-4.519956000	-1.330389000	-0.160057000
C	3.569892000	1.023620000	-0.599060000
C	-4.254201000	0.271746000	3.839303000
H	-4.857141000	0.436182000	4.728693000
C	-4.661240000	-0.839796000	-3.522140000
H	-5.344597000	-0.954036000	-4.359602000
C	2.365219000	-0.013101000	1.778489000
C	3.617981000	-0.447842000	2.243905000
H	4.421454000	-0.635911000	1.537389000
C	1.522790000	-1.783218000	-2.004962000
H	0.953889000	-1.101732000	-2.623928000
C	1.589693000	0.101303000	4.082467000
H	0.799691000	0.328071000	4.793377000
C	-2.901815000	1.144022000	2.028916000
H	-2.456947000	1.993101000	1.523042000
C	-3.681383000	1.353292000	3.168576000
H	-3.834980000	2.366690000	3.529571000
C	-5.065057000	-1.198930000	-2.234024000
H	-6.064338000	-1.592480000	-2.065909000
C	2.726451000	-2.645050000	-0.228746000
H	3.218435000	-2.744215000	0.728680000
C	-0.758301000	-4.383333000	-0.203381000
H	-0.736270000	-5.350877000	-0.688000000
C	-3.269496000	-1.237487000	2.223341000
H	-3.109466000	-2.251694000	1.870866000
C	2.496974000	-3.696904000	-1.162412000
H	2.789335000	-4.731309000	-1.034640000
C	3.847102000	-0.620177000	3.609341000
H	4.820632000	-0.958613000	3.954702000
C	5.854668000	2.388815000	-1.521921000
H	6.736176000	2.915037000	-1.878111000
C	5.392322000	1.256811000	-2.193202000
H	5.914869000	0.894013000	-3.074665000
C	4.259862000	0.577566000	-1.736007000
H	3.917893000	-0.305371000	-2.266889000
C	-3.381044000	-0.321740000	-3.726516000
H	-3.063647000	-0.024942000	-4.722771000
C	5.175724000	2.841686000	-0.387250000
H	5.528167000	3.721566000	0.144805000
C	-0.357636000	-2.663353000	1.282037000
H	0.014404000	-2.105347000	2.130197000
C	4.042321000	2.168805000	0.066619000
H	3.525596000	2.533208000	0.951041000
C	-1.363611000	-3.202418000	-0.724853000
H	-1.885209000	-3.118150000	-1.667910000
C	1.758212000	-3.164013000	-2.261479000
H	1.396049000	-3.721754000	-3.115362000
C	-0.145272000	-4.051992000	1.041493000
H	0.425978000	-4.722655000	1.670428000
Ni	-0.107345000	1.163583000	-0.540395000
P	-1.651118000	-0.364336000	0.016140000
O	0.033747000	2.454868000	-1.977791000
C	1.082628000	3.392365000	-2.128744000
H	1.145063000	3.653480000	-3.194044000
H	0.876058000	4.312847000	-1.562172000
H	2.051855000	2.995377000	-1.804721000
H	-1.043305000	5.234590000	1.775940000
H	-3.377741000	5.564562000	0.962961000

```
Zero-point correction=                           0.649714 (Hartree/Particle)
 Thermal correction to Energy=                    0.691759
 Thermal correction to Enthalpy=                  0.692703
 Thermal correction to Gibbs Free Energy=         0.571872
 Sum of electronic and zero-point Energies=          -2633.997603
 Sum of electronic and thermal Energies=             -2633.955558
 Sum of electronic and thermal Enthalpies=           -2633.954614
 Sum of electronic and thermal Free Energies=        -2634.075445
```

CPCM (Toluene) M06L/6-311++G(d,p) with LANL2DZ (for Ni, Fe) E = -2635.03699782

PhOMe

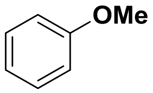

C	0.455115000	-0.271338000	-0.000417000
C	0.032808000	1.063677000	-0.000244000
C	-0.495172000	-1.303847000	0.000034000
C	-1.336290000	1.351961000	-0.000046000
C	-1.852295000	-1.000660000	0.000185000
C	-2.283926000	0.330785000	0.000105000
H	0.750611000	1.876148000	-0.000200000
H	-0.142786000	-2.330711000	0.000124000
H	-1.655989000	2.390975000	0.000087000
H	-2.578715000	-1.809227000	0.000463000
H	-3.344445000	0.565049000	0.000348000
O	1.761726000	-0.671832000	-0.000387000
C	2.768830000	0.324805000	0.000456000
H	2.711674000	0.959986000	-0.893971000
H	2.710635000	0.959840000	0.894878000
H	3.720793000	-0.209709000	0.000923000

```
Zero-point correction=                           0.133703 (Hartree/Particle)
 Thermal correction to Energy=                    0.140545
 Thermal correction to Enthalpy=                  0.141489
 Thermal correction to Gibbs Free Energy=         0.102663
 Sum of electronic and zero-point Energies=           -346.631156
 Sum of electronic and thermal Energies=              -346.624314
 Sum of electronic and thermal Enthalpies=            -346.623370
 Sum of electronic and thermal Free Energies=         -346.662196

CPCM (Toluene) M06L/6-311++G(d,p) E = -346.813389427
```

TS-oa-Ph-OPiv

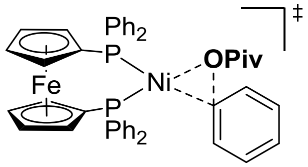

C	-0.393027000	2.089261000	1.860635000
C	-1.661730000	2.515473000	2.315860000
C	0.586423000	1.678817000	2.800057000
C	-1.982660000	2.381742000	3.658662000
C	0.225729000	1.526937000	4.152823000
H	1.625602000	1.563434000	2.500700000
C	-1.051993000	1.867991000	4.584424000
H	0.974604000	1.193794000	4.868863000
H	-1.318977000	1.780744000	5.634081000
H	-2.384901000	2.906472000	1.607384000
H	-2.976523000	2.667069000	3.997594000
Fe	-0.105741000	-1.798661000	-2.615762000
P	1.664767000	-1.003848000	0.198441000
C	-2.893499000	1.300247000	-0.818847000
C	-3.009575000	-0.974512000	1.030110000
C	-2.214085000	2.293061000	-1.545690000
H	-1.133188000	2.239328000	-1.639257000
C	1.646711000	-1.389015000	-1.585064000
C	0.469923000	-2.989300000	1.782267000
H	-0.329362000	-2.264074000	1.901337000
C	1.405552000	-5.166527000	2.270217000
H	1.333796000	-6.136059000	2.756117000
C	-1.815643000	-1.244461000	-1.533313000
C	-4.906325000	-2.434043000	1.473068000

H	-5.731548000	-3.036734000	1.102373000
C	-4.282147000	1.411103000	-0.660268000
H	-4.826612000	0.668157000	-0.086890000
C	3.413763000	-0.491118000	0.522833000
C	-4.668042000	-2.339837000	2.846906000
H	-5.308346000	-2.868840000	3.548106000
C	-4.296360000	3.450957000	-1.967451000
H	-4.839223000	4.282969000	-2.408073000
C	1.594952000	-2.659813000	1.012778000
C	2.635274000	-3.597720000	0.896832000
H	3.526764000	-3.347165000	0.328723000
C	1.413063000	-0.387630000	-2.592857000
H	1.266142000	0.664027000	-2.388301000
C	0.373531000	-4.237129000	2.404640000
H	-0.505442000	-4.475239000	2.997669000
C	-2.776694000	-0.892350000	2.411708000
H	-1.949365000	-0.295034000	2.781079000
C	-3.602698000	-1.568969000	3.313354000
H	-3.408255000	-1.491327000	4.379743000
C	-4.976689000	2.481965000	-1.228443000
H	-6.052326000	2.557399000	-1.090598000
C	1.752778000	-2.653343000	-2.257963000
H	1.881899000	-3.613898000	-1.779184000
C	-1.864177000	-2.148313000	-3.672009000
H	-1.914341000	-2.233425000	-4.749906000
C	-4.083203000	-1.758299000	0.571507000
H	-4.271233000	-1.849292000	-0.494297000
C	1.589421000	-2.429821000	-3.655971000
H	1.574489000	-3.195186000	-4.421368000
C	2.538717000	-4.843071000	1.517832000
H	3.350192000	-5.559552000	1.419705000
C	5.995247000	0.450909000	1.130353000
H	6.992538000	0.813897000	1.364031000
C	5.540027000	0.446307000	-0.188581000
H	6.183605000	0.803318000	-0.988600000
C	4.258409000	-0.020504000	-0.492053000
H	3.918928000	-0.019181000	-1.522629000
C	-2.912180000	3.352661000	-2.126306000
H	-2.371825000	4.110577000	-2.687600000
C	5.160411000	-0.015452000	2.149293000
H	5.506121000	-0.019007000	3.179818000
C	-1.576419000	-2.659714000	-1.440489000
H	-1.385772000	-3.208711000	-0.528405000
C	3.879890000	-0.478684000	1.848782000
H	3.242074000	-0.841897000	2.650845000
C	-1.982488000	-0.939379000	-2.924404000
H	-2.150248000	0.047312000	-3.333504000
C	1.386407000	-1.031976000	-3.862361000
H	1.194525000	-0.551594000	-4.813144000
C	-1.620515000	-3.212731000	-2.754082000
H	-1.450528000	-4.250649000	-3.009958000
Ni	0.145531000	0.711331000	0.693473000
P	-1.862598000	-0.069390000	-0.109993000
O	1.272605000	2.017675000	-0.411630000
C	1.090469000	3.230315000	-0.092498000
O	0.327712000	3.612022000	0.842924000
C	1.885498000	4.325860000	-0.841064000
C	2.416897000	3.799034000	-2.183819000
C	3.072265000	4.707329000	0.074921000
H	2.714719000	5.078608000	1.040533000
H	3.722720000	3.843607000	0.255068000
H	3.673025000	5.493690000	-0.398303000
H	3.060031000	2.926810000	-2.040457000
H	2.997523000	4.581269000	-2.688169000
H	1.597024000	3.507286000	-2.850813000
C	0.990617000	5.558402000	-1.069788000
H	0.135118000	5.314169000	-1.711436000
H	0.600467000	5.941123000	-0.123757000
H	1.564464000	6.352764000	-1.562861000

```
 Zero-point correction=                           0.744274 (Hartree/Particle)
 Thermal correction to Energy=                    0.792127
 Thermal correction to Enthalpy=                  0.793071
 Thermal correction to Gibbs Free Energy=         0.659937
 Sum of electronic and zero-point Energies=          -2865.219396
 Sum of electronic and thermal Energies=             -2865.171544
 Sum of electronic and thermal Enthalpies=           -2865.170599
 Sum of electronic and thermal Free Energies=        -2865.303733
```

CPCM (Toluene) M06L/6-311++G(d,p) with LANL2DZ (for Ni, Fe) E = -2866.40242598

PhOPiv

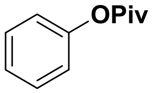

C	2.484791000	0.104325000	0.051496000
C	3.497826000	-0.852028000	-0.597567000
H	4.517419000	-0.513177000	-0.381830000
H	3.383425000	-1.870739000	-0.214620000
H	3.367629000	-0.891059000	-1.683220000
C	2.677280000	0.125252000	1.584722000
H	3.692265000	0.464266000	1.822250000
H	1.966128000	0.801175000	2.067578000
H	2.548476000	-0.874992000	2.014601000
C	2.660600000	1.531288000	-0.515908000
H	2.515656000	1.546856000	-1.602370000
H	1.951714000	2.230434000	-0.063705000
H	3.676329000	1.886733000	-0.307508000
C	1.070684000	-0.392305000	-0.269468000
O	0.794517000	-1.362317000	-0.933042000
O	0.131872000	0.428629000	0.304704000
C	-1.234092000	0.186479000	0.145499000
C	-1.807982000	-1.065614000	0.368015000
C	-2.019172000	1.295019000	-0.167916000
C	-3.193780000	-1.197542000	0.264605000
H	-1.184892000	-1.918061000	0.607283000
C	-3.403392000	1.149512000	-0.263914000
H	-1.538255000	2.255043000	-0.327328000
C	-3.994450000	-0.097367000	-0.050250000
H	-3.647494000	-2.170350000	0.433111000
H	-4.017379000	2.012528000	-0.506292000
H	-5.072010000	-0.211256000	-0.127115000

```
Zero-point correction=                           0.227693 (Hartree/Particle)
 Thermal correction to Energy=                    0.240650
 Thermal correction to Enthalpy=                  0.241594
 Thermal correction to Gibbs Free Energy=         0.187245
 Sum of electronic and zero-point Energies=           -577.828928
 Sum of electronic and thermal Energies=              -577.815972
 Sum of electronic and thermal Enthalpies=            -577.815027
 Sum of electronic and thermal Free Energies=         -577.869376
```

CPCM (Toluene) M06L/6-311++G(d,p) E = -578.14601752

TS-oa-Ph-OTs

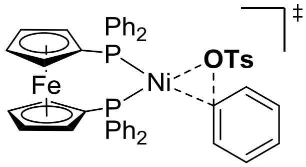

Fe	2.062586000	-0.017664000	-2.756838000
C	2.650569000	2.047529000	0.646160000
C	0.231774000	3.487749000	0.144280000
C	3.157082000	1.132434000	1.578328000
H	2.540880000	0.297732000	1.894541000
C	2.157320000	-1.660339000	-1.468564000
C	-1.152257000	-3.231069000	-0.853942000
H	-1.589890000	-2.242575000	-0.947882000
C	-1.360528000	-5.634973000	-1.079983000
H	-1.947676000	-6.508387000	-1.351851000
C	1.213496000	1.637003000	-1.845886000
C	-1.225628000	5.265885000	-0.635228000
H	-1.903378000	5.653043000	-1.390983000
C	3.445823000	3.149384000	0.283744000
H	3.053931000	3.898486000	-0.397650000
C	2.346553000	-2.462447000	1.297637000
C	-0.952216000	6.011987000	0.511572000
H	-1.409971000	6.987694000	0.651999000
C	5.230043000	2.371258000	1.723583000
H	6.227421000	2.496472000	2.136925000
C	0.157920000	-3.378541000	-0.370437000
C	0.694559000	-4.669805000	-0.240232000
H	1.700491000	-4.807360000	0.142942000
C	3.433707000	-1.002181000	-1.565841000
H	3.967658000	-0.535629000	-0.749474000
C	-1.901575000	-4.355871000	-1.211310000
H	-2.913650000	-4.225456000	-1.585501000
C	0.497617000	4.242710000	1.299990000
H	1.177963000	3.861408000	2.055684000
C	-0.089303000	5.495846000	1.480459000
H	0.132191000	6.068925000	2.377315000
C	4.726741000	3.305474000	0.812963000
H	5.329722000	4.161497000	0.521179000
C	1.800404000	-2.088671000	-2.789699000
H	0.880372000	-2.589483000	-3.057863000
C	2.065274000	1.654145000	-4.004024000
H	2.742050000	1.774332000	-4.840341000
C	-0.641048000	4.011831000	-0.819607000
H	-0.889397000	3.436619000	-1.703329000
C	2.845526000	-1.712874000	-3.683296000
H	2.849762000	-1.870550000	-4.753967000
C	-0.061017000	-5.788866000	-0.590781000
H	0.366594000	-6.782096000	-0.479942000
C	4.082335000	-3.376980000	3.314295000
H	4.754781000	-3.728311000	4.092505000
C	4.415886000	-3.549821000	1.969389000
H	5.348565000	-4.036977000	1.696845000
C	3.554815000	-3.097462000	0.967326000
H	3.829882000	-3.233409000	-0.073792000
C	4.440447000	1.288582000	2.110852000
H	4.813541000	0.564181000	2.829782000
C	2.882595000	-2.748145000	3.653630000
H	2.615401000	-2.606013000	4.697437000
C	0.256215000	1.011324000	-2.720728000
H	-0.684781000	0.581227000	-2.405960000
C	2.022001000	-2.291242000	2.653667000
H	1.096533000	-1.791147000	2.925134000
C	2.333705000	2.031849000	-2.656935000
H	3.248414000	2.482647000	-2.300149000
C	3.857522000	-1.050222000	-2.926773000

H	4.766521000	-0.614426000	-3.320927000
C	0.783549000	1.028309000	-4.042039000
H	0.312879000	0.595618000	-4.915326000
P	1.130257000	-1.851586000	0.042537000
P	0.950108000	1.791566000	-0.041179000
Ni	-0.144252000	-0.031487000	0.783479000
C	-1.318345000	0.643709000	2.050858000
O	-2.080265000	-0.212615000	-0.641924000
C	-1.561124000	-0.735203000	2.299882000
C	-1.086842000	1.542267000	3.116512000
S	-3.001333000	0.943836000	-0.372416000
H	-1.997642000	-1.370278000	1.535312000
C	-1.444009000	-1.211840000	3.631840000
H	-0.983471000	2.602341000	2.911617000
C	-0.950532000	1.034154000	4.395920000
O	-2.751116000	1.467770000	1.048079000
O	-2.991495000	1.989032000	-1.408455000
C	-1.124507000	-0.345454000	4.662310000
H	-0.703740000	1.710020000	5.211817000
H	-1.047904000	-0.710558000	5.682851000
H	-1.662220000	-2.257533000	3.839598000
C	-4.668232000	0.277521000	-0.307376000
C	-5.499063000	0.426986000	-1.417842000
C	-5.112054000	-0.405686000	0.826783000
C	-6.779895000	-0.124407000	-1.392283000
C	-6.394256000	-0.950914000	0.835592000
C	-7.246055000	-0.825280000	-0.272079000
H	-5.145959000	0.984956000	-2.278393000
H	-4.470679000	-0.487557000	1.698360000
H	-7.430398000	-0.000958000	-2.255142000
H	-6.744415000	-1.474138000	1.722530000
C	-8.624837000	-1.443230000	-0.261915000
H	-8.594138000	-2.485527000	-0.607289000
H	-9.054950000	-1.448499000	0.745409000
H	-9.311563000	-0.902482000	-0.921588000

```
Zero-point correction=                           0.740849 (Hartree/Particle)
 Thermal correction to Energy=                    0.790847
 Thermal correction to Enthalpy=                  0.791791
 Thermal correction to Gibbs Free Energy=         0.652923
 Sum of electronic and zero-point Energies=          -3413.562821
 Sum of electronic and thermal Energies=             -3413.512823
 Sum of electronic and thermal Enthalpies=           -3413.511879
 Sum of electronic and thermal Free Energies=        -3413.650747
```

CPCM (Toluene) M06L/6-311++G(d,p) with LANL2DZ (for Ni, Fe) E = -3414.78260973

PhOTs

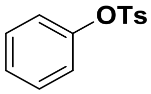

C	2.266980000	0.000005000	-0.513395000
C	2.945197000	1.217407000	-0.538879000
C	2.945208000	-1.217392000	-0.538860000
C	4.339435000	1.210157000	-0.600570000
C	4.339446000	-1.210130000	-0.600550000
C	5.036889000	0.000016000	-0.632241000
H	2.381513000	2.143362000	-0.509755000
H	2.381534000	-2.143352000	-0.509721000
H	4.879899000	2.152310000	-0.622553000
H	4.879918000	-2.152279000	-0.622517000
H	6.122181000	0.000021000	-0.680924000
O	0.861694000	0.000000000	-0.526123000
S	0.104572000	-0.000016000	0.966237000
O	0.395897000	1.269390000	1.628664000
O	0.395893000	-1.269440000	1.628633000

C	-1.569900000	-0.000006000	0.362996000
C	-2.214061000	1.217248000	0.136755000
C	-2.214052000	-1.217253000	0.136686000
C	-3.525257000	1.206060000	-0.333230000
C	-3.525247000	-1.206049000	-0.333298000
C	-4.200130000	0.000010000	-0.574270000
H	-1.696933000	2.150116000	0.333649000
H	-1.696916000	-2.150128000	0.333528000
H	-4.034427000	2.149399000	-0.513557000
H	-4.034409000	-2.149382000	-0.513679000
C	-5.632354000	0.000016000	-1.052761000
H	-5.853904000	0.886941000	-1.655149000
H	-5.853862000	-0.886818000	-1.655298000
H	-6.328520000	-0.000073000	-0.203519000

```
Zero-point correction=                           0.223432 (Hartree/Particle)
 Thermal correction to Energy=                    0.238774
 Thermal correction to Enthalpy=                  0.239718
 Thermal correction to Gibbs Free Energy=         0.176457
 Sum of electronic and zero-point Energies=          -1126.166183
 Sum of electronic and thermal Energies=             -1126.150841
 Sum of electronic and thermal Enthalpies=           -1126.149897
 Sum of electronic and thermal Free Energies=        -1126.213158

CPCM (Toluene) M06L/6-311++G(d,p) E = -1126.5188804
```

TS-oa-Ph-OMs

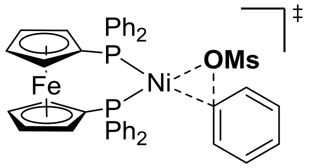

Fe	-0.583780000	-1.983580000	-2.327212000
C	1.336796000	-2.258849000	1.197655000
C	3.395172000	-0.560167000	0.160162000
C	0.336159000	-2.231681000	2.178119000
H	-0.277642000	-1.344072000	2.287018000
C	-2.146689000	-1.266966000	-1.140616000
C	-2.598061000	2.368326000	-1.546977000
H	-1.528537000	2.411425000	-1.725927000
C	-4.814293000	3.280643000	-1.908147000
H	-5.465830000	4.016133000	-2.372859000
C	1.274296000	-1.440999000	-1.590506000
C	5.483737000	0.090263000	-0.891672000
H	6.019061000	0.420415000	-1.777509000
C	2.151710000	-3.400750000	1.097835000
H	2.964544000	-3.424639000	0.378234000
C	-2.888444000	-0.462512000	1.529833000
C	6.162710000	-0.089160000	0.313916000
H	7.232757000	0.092330000	0.371661000
C	0.925359000	-4.466340000	2.893075000
H	0.766525000	-5.320709000	3.545813000
C	-3.130404000	1.382048000	-0.700403000
C	-4.513619000	1.363880000	-0.460557000
H	-4.943716000	0.617618000	0.199439000
C	-1.883182000	-2.654797000	-0.866042000
H	-1.569747000	-3.062595000	0.085065000
C	-3.440447000	3.305859000	-2.151087000
H	-3.015675000	4.061922000	-2.806439000
C	4.084477000	-0.731429000	1.373116000
H	3.552941000	-1.062956000	2.260342000
C	5.458473000	-0.499080000	1.447808000
H	5.977388000	-0.642878000	2.392179000
C	1.942726000	-4.497128000	1.934305000
H	2.579619000	-5.373228000	1.843510000

C	-2.495310000	-1.169041000	-2.528326000
H	-2.719265000	-0.251411000	-3.054573000
C	0.977193000	-2.766894000	-3.470315000
H	0.874950000	-3.633746000	-4.110778000
C	4.109715000	-0.141243000	-0.971009000
H	3.598729000	0.032001000	-1.910604000
C	-2.458489000	-2.476363000	-3.095463000
H	-2.641889000	-2.720573000	-4.133741000
C	-5.348006000	2.308907000	-1.058630000
H	-6.416226000	2.285363000	-0.858892000
C	-4.228391000	-1.253361000	3.873319000
H	-4.744393000	-1.561896000	4.778722000
C	-4.546972000	-1.845076000	2.649123000
H	-5.312872000	-2.614642000	2.598081000
C	-3.883209000	-1.452805000	1.484808000
H	-4.136536000	-1.924314000	0.540472000
C	0.127055000	-3.329375000	3.018376000
H	-0.656641000	-3.287408000	3.769915000
C	-3.242173000	-0.266397000	3.928799000
H	-2.985482000	0.197879000	4.877291000
C	0.916167000	-0.592193000	-2.696550000
H	0.783603000	0.479935000	-2.637437000
C	-2.574443000	0.125215000	2.766738000
H	-1.801142000	0.886684000	2.819798000
C	1.308013000	-2.790852000	-2.084872000
H	1.497681000	-3.678085000	-1.498327000
C	-2.088379000	-3.394223000	-2.067972000
H	-1.940449000	-4.459767000	-2.187060000
C	0.738582000	-1.411369000	-3.846053000
H	0.429438000	-1.067895000	-4.824741000
P	-1.972071000	0.133573000	0.037003000
P	1.560663000	-0.789788000	0.094863000
Ni	0.164425000	0.951720000	0.531285000
C	1.192414000	2.151596000	1.491986000
O	0.568912000	2.446090000	-1.390011000
C	-0.028044000	2.876158000	1.555925000
C	1.990028000	1.969691000	2.642545000
S	1.951506000	3.019063000	-1.266233000
H	-0.505513000	3.244169000	0.652679000
C	-0.492153000	3.305257000	2.826514000
H	2.956500000	1.485001000	2.554934000
C	1.491006000	2.379002000	3.866587000
O	2.448986000	2.881186000	0.176160000
O	2.918025000	2.543623000	-2.268186000
C	0.245920000	3.044777000	3.967767000
H	-1.407820000	3.890255000	2.885052000
C	1.769051000	4.800088000	-1.497758000
H	1.387772000	4.977735000	-2.505680000
H	1.072652000	5.180088000	-0.747665000
H	2.749892000	5.263643000	-1.376156000
H	-0.104600000	3.386251000	4.937906000
H	2.067654000	2.181905000	4.767726000

```
Zero-point correction=                           0.660486 (Hartree/Particle)
 Thermal correction to Energy=                   0.705345
 Thermal correction to Enthalpy=                 0.706289
 Thermal correction to Gibbs Free Energy=        0.581248
 Sum of electronic and zero-point Energies=          -3182.597212
 Sum of electronic and thermal Energies=             -3182.552353
 Sum of electronic and thermal Enthalpies=           -3182.551409
 Sum of electronic and thermal Free Energies=        -3182.676450
```

CPCM (Toluene) M06L/6-311++G(d,p) with LANL2DZ (for Ni, Fe) E = -3183.70829609

PhOMs

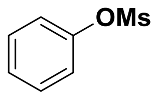

C	3.128306000	-0.103379000	0.870186000
H	3.062463000	-1.050742000	1.406456000
H	4.032254000	-0.068599000	0.258380000
H	3.095951000	0.743552000	1.556669000
S	1.740308000	0.017528000	-0.261593000
O	1.659806000	-1.184877000	-1.085314000
O	1.718047000	1.339441000	-0.880881000
O	0.566039000	-0.052206000	0.922017000
C	-0.769956000	-0.025140000	0.482698000
C	-1.405922000	1.203393000	0.315360000
C	-1.437013000	-1.231914000	0.283175000
C	-2.752148000	1.217457000	-0.052780000
H	-0.848777000	2.120493000	0.471731000
C	-2.783410000	-1.202807000	-0.082907000
H	-0.902713000	-2.166455000	0.414929000
C	-3.441205000	0.018313000	-0.250520000
H	-3.261229000	2.167845000	-0.185602000
H	-3.316607000	-2.136242000	-0.239297000
H	-4.489307000	0.035296000	-0.535637000

```
Zero-point correction=                           0.142995 (Hartree/Particle)
 Thermal correction to Energy=                    0.153280
 Thermal correction to Enthalpy=                  0.154224
 Thermal correction to Gibbs Free Energy=         0.104770
 Sum of electronic and zero-point Energies=           -895.199379
 Sum of electronic and thermal Energies=              -895.189094
 Sum of electronic and thermal Enthalpies=            -895.188150
 Sum of electronic and thermal Free Energies=         -895.237605
```

```
CPCM (Toluene) M06L/6-311++G(d,p) E = -895.444087975
```

TS-oa-Ph-OTf

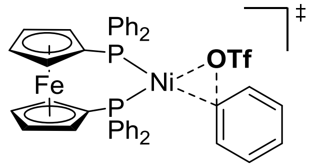

Fe	1.741295000	1.252862000	-2.518150000
C	0.692827000	2.906262000	0.987327000
C	-2.062427000	2.667481000	0.226425000
C	1.608748000	2.366486000	1.901635000
H	1.620698000	1.295385000	2.077873000
C	2.672311000	-0.158529000	-1.299388000
C	0.621517000	-3.219068000	-1.359404000
H	-0.236604000	-2.559542000	-1.443318000
C	1.646596000	-5.367175000	-1.808633000
H	1.597142000	-6.361468000	-2.244638000
C	-0.001952000	1.980605000	-1.678930000
C	-4.212257000	3.324747000	-0.688566000
H	-4.906421000	3.336334000	-1.523766000
C	0.666918000	4.299584000	0.801141000
H	-0.064044000	4.743027000	0.131517000
C	3.071583000	-1.167950000	1.367711000
C	-4.565161000	3.908711000	0.527705000
H	-5.533981000	4.387064000	0.643630000
C	2.481429000	4.571243000	2.381150000
H	3.172673000	5.215730000	2.917684000

C	1.773325000	-2.801775000	-0.672316000
C	2.856664000	-3.686410000	-0.554442000
H	3.750538000	-3.386453000	-0.016840000
C	3.295198000	1.132220000	-1.162357000
H	3.346781000	1.716677000	-0.254219000
C	0.565012000	-4.493451000	-1.929618000
H	-0.332755000	-4.802111000	-2.458326000
C	-2.427948000	3.251696000	1.451627000
H	-1.737195000	3.238693000	2.290002000
C	-3.669213000	3.870518000	1.598636000
H	-3.934637000	4.324132000	2.550148000
C	1.558625000	5.123625000	1.487993000
H	1.528056000	6.198754000	1.331761000
C	2.794433000	-0.543384000	-2.675894000
H	2.398076000	-1.450985000	-3.109433000
C	0.844917000	2.717101000	-3.708160000
H	1.379002000	3.299331000	-4.448254000
C	-2.969029000	2.708487000	-0.841043000
H	-2.721081000	2.246616000	-1.790260000
C	3.485963000	0.489959000	-3.372464000
H	3.698091000	0.504761000	-4.433547000
C	2.790629000	-4.961775000	-1.117228000
H	3.634197000	-5.639243000	-1.013161000
C	4.901689000	-1.335023000	3.495519000
H	5.609779000	-1.397586000	4.317580000
C	5.358067000	-1.120829000	2.193010000
H	6.422274000	-1.017253000	1.997989000
C	4.450465000	-1.037307000	1.135275000
H	4.816651000	-0.863346000	0.128167000
C	2.500582000	3.192222000	2.592107000
H	3.202901000	2.753695000	3.295802000
C	3.532710000	-1.464724000	3.737195000
H	3.168915000	-1.628490000	4.748075000
C	-0.314894000	1.001314000	-2.686829000
H	-0.842764000	0.073361000	-2.509846000
C	2.622269000	-1.378839000	2.682151000
H	1.558243000	-1.475893000	2.878874000
C	0.717934000	3.042884000	-2.327291000
H	1.140888000	3.910304000	-1.841182000
C	3.802623000	1.519523000	-2.437052000
H	4.297572000	2.455439000	-2.661651000
C	0.206333000	1.460492000	-3.928630000
H	0.164654000	0.924561000	-4.867803000
P	1.804082000	-1.086735000	0.025542000
P	-0.452605000	1.773869000	0.079718000
Ni	-0.288266000	-0.374724000	0.758317000
C	-1.763069000	-0.708412000	1.817401000
O	-2.082988000	-1.382526000	-1.213232000
C	-1.192173000	-2.011148000	1.780576000
C	-2.129329000	-0.101207000	3.040144000
S	-3.422413000	-1.015929000	-0.692101000
H	-1.207419000	-2.599882000	0.868920000
C	-0.908490000	-2.652053000	3.019814000
H	-2.629825000	0.861474000	3.033927000
C	-1.792973000	-0.739250000	4.218944000
O	-3.367690000	-0.531236000	0.752923000
O	-4.281318000	-0.200899000	-1.552102000
C	-4.301280000	-2.650168000	-0.531760000
C	-1.181349000	-2.018675000	4.216326000
H	-2.002313000	-0.248278000	5.166681000
F	-4.438639000	-3.208190000	-1.740859000
F	-5.509810000	-2.481819000	0.009650000
F	-3.594320000	-3.487119000	0.244771000
H	-0.971436000	-2.514383000	5.160061000
H	-0.528463000	-3.671301000	3.009694000

```
Zero-point correction=                           0.637038 (Hartree/Particle)
 Thermal correction to Energy=                    0.684143
 Thermal correction to Enthalpy=                  0.685088
 Thermal correction to Gibbs Free Energy=         0.553478
```

```
 Sum of electronic and zero-point Energies=          -3480.321906
 Sum of electronic and thermal Energies=             -3480.274801
 Sum of electronic and thermal Enthalpies=           -3480.273857
 Sum of electronic and thermal Free Energies=        -3480.405466

CPCM (Toluene) M06L/6-311++G(d,p) with LANL2DZ (for Ni, Fe) E = -3481.47790231
```

PhOTf

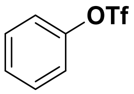

C	-4.213133000	0.032795000	0.002983000
C	-3.509771000	1.239845000	-0.014725000
C	-3.536859000	-1.176699000	-0.174722000
C	-2.127688000	1.245799000	-0.210060000
H	-4.034752000	2.180353000	0.124373000
C	-2.155048000	-1.187968000	-0.372624000
H	-4.083006000	-2.115337000	-0.160435000
C	-1.477906000	0.028104000	-0.388563000
H	-1.557371000	2.168018000	-0.226828000
H	-1.606316000	-2.112531000	-0.514444000
O	-0.088813000	0.032950000	-0.659243000
S	0.915415000	-0.111535000	0.638427000
H	-5.288591000	0.034766000	0.155062000
O	0.874062000	-1.467571000	1.164634000
O	0.792389000	1.049259000	1.507130000
C	2.468230000	0.075378000	-0.384715000
F	2.475557000	1.261051000	-0.985219000
F	2.534345000	-0.894659000	-1.290790000
F	3.502914000	-0.014318000	0.449545000

```
Zero-point correction=                           0.119425 (Hartree/Particle)
 Thermal correction to Energy=                   0.131880
 Thermal correction to Enthalpy=                 0.132824
 Thermal correction to Gibbs Free Energy=        0.077090
 Sum of electronic and zero-point Energies=          -1192.911815
 Sum of electronic and thermal Energies=             -1192.899361
 Sum of electronic and thermal Enthalpies=           -1192.898417
 Sum of electronic and thermal Free Energies=        -1192.954150

CPCM (Toluene) M06L/6-311++G(d,p) E = -1193.1979145
```

TS-oa-Ph-ONf

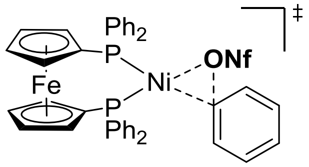

Fe	2.430491000	-0.493341000	-2.866377000
C	3.735741000	1.703524000	0.261526000
C	1.483644000	3.441593000	-0.101229000
C	4.227475000	0.751131000	1.165079000
H	3.550662000	0.013791000	1.584929000
C	2.394338000	-2.038889000	-1.464691000
C	-1.095406000	-2.723068000	-0.454581000
H	-1.275290000	-1.661550000	-0.589864000
C	-1.934868000	-4.994233000	-0.471935000
H	-2.751343000	-5.695555000	-0.622592000
C	1.970564000	1.335511000	-2.015451000

C	0.210220000	5.355353000	-0.882090000
H	-0.472833000	5.788267000	-1.607137000
C	4.618514000	2.674817000	-0.242415000
H	4.249210000	3.447483000	-0.910141000
C	2.718882000	-2.779244000	1.298778000
C	0.668967000	6.115519000	0.193327000
H	0.352008000	7.148668000	0.307147000
C	6.446356000	1.702951000	1.013165000
H	7.494040000	1.703673000	1.302025000
C	0.174556000	-3.183097000	-0.069640000
C	0.372756000	-4.560109000	0.117868000
H	1.342910000	-4.934891000	0.428050000
C	3.744245000	-1.622222000	-1.740460000
H	4.439689000	-1.211144000	-1.021510000
C	-2.141686000	-3.626929000	-0.658970000
H	-3.119768000	-3.253434000	-0.947385000
C	1.936855000	4.212376000	0.983446000
H	2.619403000	3.781032000	1.710148000
C	1.534518000	5.540171000	1.126631000
H	1.899510000	6.124978000	1.967090000
C	5.963758000	2.670085000	0.126438000
H	6.634366000	3.426667000	-0.272481000
C	1.828028000	-2.485260000	-2.704617000
H	0.813163000	-2.832203000	-2.840673000
C	2.561523000	1.072862000	-4.242486000
H	3.147474000	1.028339000	-5.151778000
C	0.614343000	4.027418000	-1.031067000
H	0.234501000	3.450321000	-1.866976000
C	2.816045000	-2.355184000	-3.723719000
H	2.675993000	-2.581174000	-4.772841000
C	-0.676574000	-5.458109000	-0.081170000
H	-0.510214000	-6.521184000	0.072653000
C	4.451512000	-4.003867000	3.144460000
H	5.123089000	-4.476043000	3.856597000
C	4.539989000	-4.315256000	1.785853000
H	5.280049000	-5.030923000	1.437362000
C	3.680383000	-3.708202000	0.868294000
H	3.763354000	-3.953783000	-0.185819000
C	5.575059000	0.747239000	1.536563000
H	5.936009000	-0.000289000	2.237787000
C	3.499446000	-3.081467000	3.582650000
H	3.425093000	-2.831451000	4.637655000
C	0.831815000	0.824194000	-2.733344000
H	-0.126057000	0.585342000	-2.290881000
C	2.640491000	-2.470275000	2.667029000
H	1.905711000	-1.750203000	3.015767000
C	3.038992000	1.485451000	-2.965512000
H	4.047122000	1.803294000	-2.741086000
C	4.001065000	-1.830928000	-3.127371000
H	4.920198000	-1.585605000	-3.643382000
C	1.201027000	0.668838000	-4.098845000
H	0.569893000	0.269125000	-4.881855000
P	1.531971000	-1.941180000	0.154791000
P	1.950528000	1.656997000	-0.216835000
Ni	0.761721000	0.110811000	0.934303000
C	-0.202349000	1.025524000	2.224285000
O	-1.555684000	0.543455000	-0.493401000
C	-0.673080000	-0.300919000	2.444878000
C	0.294885000	1.813156000	3.289608000
S	-2.164274000	1.761186000	0.094692000
H	-1.336670000	-0.783921000	1.735492000
C	-0.544734000	-0.847094000	3.754202000
H	0.580254000	2.843871000	3.107276000
C	0.447534000	1.224695000	4.529820000
O	-1.481114000	2.166836000	1.399166000
O	-2.440022000	2.886999000	-0.796255000
C	-3.847748000	1.177079000	0.712950000
C	0.026621000	-0.108620000	4.770903000
H	0.898410000	1.797541000	5.337037000
F	-4.600170000	2.263386000	0.982218000

F	-3.665844000	0.472763000	1.850860000
H	0.115125000	-0.526280000	5.769976000
H	-0.955581000	-1.834433000	3.953621000
C	-4.599578000	0.270765000	-0.297567000
C	-6.112475000	0.101803000	0.016874000
C	-6.794975000	-1.064678000	-0.745602000
F	-6.757559000	1.239493000	-0.317387000
F	-6.273117000	-0.132203000	1.341018000
F	-4.028506000	-0.962294000	-0.272042000
F	-4.490726000	0.782402000	-1.542229000
F	-6.533255000	-0.987495000	-2.057399000
F	-6.369693000	-2.247677000	-0.284641000
F	-8.119467000	-0.986723000	-0.564905000

```
Zero-point correction=                           0.673948 (Hartree/Particle)
 Thermal correction to Energy=                    0.729578
 Thermal correction to Enthalpy=                  0.730522
 Thermal correction to Gibbs Free Energy=         0.579426
 Sum of electronic and zero-point Energies=          -4193.620062
 Sum of electronic and thermal Energies=             -4193.564432
 Sum of electronic and thermal Enthalpies=           -4193.563488
 Sum of electronic and thermal Free Energies=        -4193.714584

CPCM (Toluene) M06L/6-311++G(d,p) with LANL2DZ (for Ni, Fe) E = -4194.96025405
```

PhONf

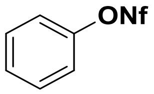

C	-6.341231000	0.138568000	0.135541000
C	-5.588084000	1.296554000	0.345475000
C	-5.725116000	-1.024010000	-0.334003000
C	-4.215983000	1.299191000	0.090116000
H	-6.066201000	2.200217000	0.712013000
C	-4.354528000	-1.036548000	-0.598934000
H	-6.309886000	-1.924387000	-0.498021000
C	-3.626924000	0.130693000	-0.382625000
H	-3.607524000	2.182945000	0.248280000
H	-3.853065000	-1.923989000	-0.969614000
O	-2.251435000	0.152884000	-0.711488000
S	-1.213516000	-0.441440000	0.426169000
H	-7.408417000	0.142521000	0.337562000
O	-1.117110000	-1.889297000	0.310916000
O	-1.448161000	0.216203000	1.702081000
C	0.307534000	0.319742000	-0.396670000
F	0.274849000	1.640219000	-0.151982000
F	0.234122000	0.094215000	-1.714371000
C	1.620535000	-0.298249000	0.160378000
C	2.876023000	0.566501000	-0.144561000
C	4.223127000	-0.177719000	0.053043000
F	1.505340000	-0.427069000	1.501259000
F	1.780577000	-1.517754000	-0.394604000
F	2.868614000	1.638820000	0.674230000
F	2.817832000	0.984022000	-1.431689000
F	5.221582000	0.710537000	-0.007120000
F	4.247616000	-0.780140000	1.248935000
F	4.398677000	-1.091840000	-0.905497000

```
Zero-point correction=                           0.156188 (Hartree/Particle)
 Thermal correction to Energy=                    0.177263
 Thermal correction to Enthalpy=                  0.178207
 Thermal correction to Gibbs Free Energy=         0.102357
 Sum of electronic and zero-point Energies=          -1906.207431
 Sum of electronic and thermal Energies=             -1906.186356
 Sum of electronic and thermal Enthalpies=           -1906.185412
 Sum of electronic and thermal Free Energies=        -1906.261262

CPCM (Toluene) M06L/6-311++G(d,p) E = -1906.67690889
```

TS-oa-Cyclohexenyl-SCF$_3$

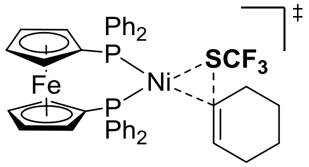

Ni	-0.134893000	-0.992336000	0.114251000
S	0.140147000	-3.010115000	-0.847492000
C	-1.138308000	-3.228356000	-2.116648000
F	-1.934378000	-4.300608000	-1.894436000
F	-1.956722000	-2.165553000	-2.268839000
F	-0.535975000	-3.440006000	-3.309748000
Fe	1.021229000	3.039927000	-0.446353000
P	-1.583357000	0.792045000	-0.110485000
P	2.036783000	-0.133496000	0.162160000
C	-2.196302000	1.509509000	1.488065000
C	-3.165816000	0.627395000	-1.075028000
C	-1.614813000	1.060282000	2.683080000
H	-0.855047000	0.284564000	2.637840000
C	2.271186000	1.663665000	0.448673000
C	2.454351000	-0.779540000	-2.552583000
H	1.382745000	-0.949000000	-2.570922000
C	4.595371000	-0.805170000	-3.680377000
H	5.184725000	-0.959723000	-4.580439000
C	-0.829416000	2.256012000	-0.930477000
C	-4.361656000	0.671294000	-3.194831000
H	-4.362428000	0.877214000	-4.262116000
C	-3.203694000	2.487171000	1.557022000
H	-3.689299000	2.829109000	0.647725000
C	3.108416000	-0.903260000	1.468624000
C	-5.520201000	0.206989000	-2.569989000
H	-6.427378000	0.046622000	-3.146276000
C	-3.003014000	2.568372000	3.968902000
H	-3.315669000	2.977920000	4.925826000
C	3.073111000	-0.410524000	-1.349952000
C	4.470212000	-0.260231000	-1.325882000
H	4.968959000	0.001818000	-0.396967000
C	1.558047000	2.420713000	1.444779000
H	0.850705000	2.019774000	2.156655000
C	3.210729000	-0.971772000	-3.712062000
H	2.715779000	-1.263876000	-4.634402000
C	-4.337492000	0.157490000	-0.458183000
H	-4.349217000	-0.036125000	0.610240000
C	-5.503189000	-0.048513000	-1.197877000
H	-6.399299000	-0.407003000	-0.697718000
C	-3.601161000	3.013891000	2.786833000
H	-4.381610000	3.769676000	2.822579000
C	3.046509000	2.604826000	-0.312704000
H	3.662155000	2.363879000	-1.167460000
C	-0.142526000	4.391955000	-1.536692000
H	0.004949000	5.464358000	-1.528069000
C	-3.195912000	0.878166000	-2.455791000

H	-2.307849000	1.243917000	-2.961527000
C	2.807780000	3.911125000	0.204842000
H	3.216128000	4.832590000	-0.190046000
C	5.225043000	-0.452570000	-2.482965000
H	6.304941000	-0.333266000	-2.449081000
C	4.621561000	-2.210694000	3.451520000
H	5.205145000	-2.714895000	4.217143000
C	4.383026000	-0.839367000	3.538837000
H	4.784209000	-0.267834000	4.372084000
C	3.633667000	-0.188322000	2.554055000
H	3.465302000	0.881091000	2.632608000
C	-2.011330000	1.588060000	3.915292000
H	-1.549691000	1.227108000	4.830732000
C	4.105971000	-2.932523000	2.371005000
H	4.288821000	-4.000885000	2.290613000
C	0.070271000	2.154361000	-2.048918000
H	0.412240000	1.230563000	-2.494000000
C	3.353358000	-2.286627000	1.391197000
H	2.959710000	-2.861030000	0.556147000
C	-0.952577000	3.655245000	-0.624204000
H	-1.520537000	4.073386000	0.194396000
C	1.894945000	3.796707000	1.294706000
H	1.488844000	4.614134000	1.876326000
C	0.486718000	3.465397000	-2.419781000
H	1.195552000	3.707624000	-3.200891000
C	-1.139851000	-2.433876000	0.878809000
C	-0.435021000	-2.545142000	2.036591000
C	-2.603992000	-2.787895000	0.772069000
C	-1.097331000	-2.765838000	3.385305000
H	0.651680000	-2.500227000	2.028091000
C	-3.296768000	-2.627864000	2.140205000
H	-3.105742000	-2.182574000	0.013688000
H	-2.695756000	-3.830974000	0.443502000
C	-2.512564000	-3.348017000	3.243807000
H	-1.145225000	-1.816069000	3.942459000
H	-0.472674000	-3.433346000	3.994641000
H	-3.377944000	-1.561013000	2.390076000
H	-4.320053000	-3.019393000	2.074036000
H	-3.043918000	-3.281294000	4.201690000
H	-2.442293000	-4.415598000	2.993285000

```
Zero-point correction=                           0.670995 (Hartree/Particle)
 Thermal correction to Energy=                    0.716341
 Thermal correction to Enthalpy=                  0.717285
 Thermal correction to Gibbs Free Energy=         0.590082
 Sum of electronic and zero-point Energies=          -3257.107089
 Sum of electronic and thermal Energies=             -3257.061743
 Sum of electronic and thermal Enthalpies=           -3257.060799
 Sum of electronic and thermal Free Energies=        -3257.188002
```

CPCM (Toluene) M06L/6-311++G(d,p) with LANL2DZ (for Ni, Fe) E = -3258.23455713

Cyclohexenyl-SCF$_3$

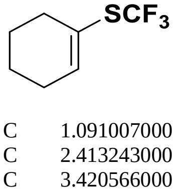

C	1.091007000	-1.089602000	-0.582271000
C	2.413243000	-1.397698000	0.140473000
C	3.420566000	-0.255484000	-0.037804000
C	2.869115000	1.049295000	0.556213000
C	1.423436000	1.281657000	0.193371000
C	0.633282000	0.331407000	-0.329469000
H	0.313799000	-1.792179000	-0.263210000
H	1.200905000	-1.238623000	-1.666647000
H	2.824748000	-2.341652000	-0.236075000

H	2.216250000	-1.542953000	1.211520000
H	4.379387000	-0.505821000	0.431016000
H	3.618719000	-0.111308000	-1.108812000
H	3.468007000	1.908589000	0.224773000
H	2.958677000	1.035406000	1.654034000
H	1.019901000	2.277265000	0.364429000
S	-0.992534000	0.827333000	-0.913084000
C	-2.084866000	-0.117665000	0.200099000
F	-2.074943000	-1.447549000	-0.036130000
F	-1.775512000	0.047351000	1.496785000
F	-3.340050000	0.318252000	0.000972000

```
Zero-point correction=                           0.153118 (Hartree/Particle)
 Thermal correction to Energy=                   0.163740
 Thermal correction to Enthalpy=                 0.164684
 Thermal correction to Gibbs Free Energy=        0.115008
 Sum of electronic and zero-point Energies=          -969.700361
 Sum of electronic and thermal Energies=             -969.689739
 Sum of electronic and thermal Enthalpies=           -969.688795
 Sum of electronic and thermal Free Energies=        -969.738471

CPCM (Toluene) M06L/6-311++G(d,p) E = -969.961852266
```

TS-oa-Cyclohexenyl-OPiv

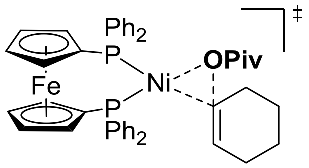

Fe	0.196105000	-1.891129000	-2.577931000
P	1.802048000	-0.734130000	0.212705000
C	-3.045668000	0.765196000	-0.850216000
C	-2.803160000	-1.459920000	1.040994000
C	-2.534586000	1.793892000	-1.660030000
H	-1.461470000	1.877750000	-1.800701000
C	1.846550000	-1.162643000	-1.560248000
C	0.934645000	-2.877828000	1.804453000
H	0.017344000	-2.302794000	1.884697000
C	2.225119000	-4.846809000	2.361664000
H	2.311463000	-5.806219000	2.864991000
C	-1.580933000	-1.597379000	-1.507054000
C	-4.495533000	-3.143764000	1.513629000
H	-5.251361000	-3.838499000	1.156442000
C	-4.429143000	0.702895000	-0.631427000
H	-4.847586000	-0.068571000	0.006187000
C	3.450528000	0.053681000	0.512332000
C	-4.235301000	-3.034893000	2.882778000
H	-4.789242000	-3.643727000	3.592506000
C	-4.768431000	2.639763000	-2.046293000
H	-5.434689000	3.364637000	-2.506529000
C	2.005764000	-2.364139000	1.059829000
C	3.198434000	-3.104178000	0.991557000
H	4.049113000	-2.708503000	0.443760000
C	1.450407000	-0.242317000	-2.593619000
H	1.121937000	0.771853000	-2.412049000
C	1.040905000	-4.113716000	2.448266000
H	0.199889000	-4.495889000	3.020867000
C	-2.544128000	-1.369719000	2.416462000
H	-1.768058000	-0.692577000	2.761833000
C	-3.256216000	-2.148619000	3.332641000
H	-3.041438000	-2.063878000	4.394789000
C	-5.282907000	1.637023000	-1.223506000
H	-6.352623000	1.578106000	-1.039175000
C	2.172055000	-2.407298000	-2.199127000

H	2.460808000	-3.318693000	-1.694443000
C	-1.478878000	-2.558098000	-3.618860000
H	-1.513400000	-2.680667000	-4.693752000
C	-3.784044000	-2.365557000	0.599831000
H	-3.986656000	-2.468306000	-0.462283000
C	1.978559000	-2.251641000	-3.602479000
H	2.097796000	-3.027930000	-4.347599000
C	3.305285000	-4.337663000	1.634789000
H	4.233840000	-4.899367000	1.573636000
C	5.877278000	1.362853000	1.072533000
H	6.814675000	1.868644000	1.288139000
C	5.462507000	1.184881000	-0.247377000
H	6.077487000	1.549799000	-1.066072000
C	4.257539000	0.534213000	-0.527641000
H	3.947931000	0.402526000	-1.559199000
C	-3.390873000	2.714520000	-2.264386000
H	-2.979128000	3.502043000	-2.890212000
C	5.079131000	0.887244000	2.116486000
H	5.393973000	1.019349000	3.148457000
C	-1.112775000	-2.951422000	-1.374946000
H	-0.835330000	-3.437045000	-0.449355000
C	3.874308000	0.242851000	1.838886000
H	3.263059000	-0.122161000	2.660571000
C	-1.796341000	-1.364344000	-2.905374000
H	-2.125601000	-0.431576000	-3.341585000
C	1.540118000	-0.914936000	-3.845378000
H	1.273096000	-0.499747000	-4.808668000
C	-1.063335000	-3.540864000	-2.672357000
H	-0.723317000	-4.543065000	-2.899500000
Ni	0.035475000	0.683935000	0.730664000
P	-1.807427000	-0.402824000	-0.115886000
O	-0.438733000	3.630561000	0.497357000
O	0.878732000	2.139026000	-0.538698000
C	1.024106000	4.443617000	-1.243604000
C	-0.707391000	1.976266000	1.837424000
C	0.427069000	1.905599000	2.594382000
C	-2.067603000	2.317152000	2.372828000
C	0.380629000	1.896416000	4.114419000
H	1.404257000	2.044576000	2.135069000
C	-1.973875000	2.794091000	3.838047000
H	-2.504589000	3.095001000	1.740091000
H	-2.738024000	1.450760000	2.302266000
C	-1.060254000	1.868236000	4.654713000
H	0.885113000	2.805715000	4.480335000
H	0.955280000	1.051410000	4.520023000
H	-1.567598000	3.814340000	3.860327000
H	-2.977846000	2.837017000	4.279204000
H	-1.063265000	2.155544000	5.713699000
H	-1.455159000	0.842801000	4.608444000
C	0.139151000	5.699897000	-1.203413000
H	0.589152000	6.498262000	-1.807358000
H	-0.860565000	5.492959000	-1.601850000
H	0.015822000	6.062499000	-0.179879000
C	2.416933000	4.763717000	-0.654158000
H	2.905830000	5.551003000	-1.242008000
H	2.331732000	5.118370000	0.379826000
H	3.058206000	3.876380000	-0.660214000
C	1.182224000	3.958945000	-2.696311000
H	0.211896000	3.696939000	-3.137307000
H	1.622687000	4.751890000	-3.313807000
H	1.829331000	3.079796000	-2.751529000
C	0.421110000	3.315810000	-0.361975000

```
 Zero-point correction=                           0.791084 (Hartree/Particle)
 Thermal correction to Energy=                    0.839878
 Thermal correction to Enthalpy=                  0.840822
 Thermal correction to Gibbs Free Energy=         0.706692
 Sum of electronic and zero-point Energies=          -2867.582065
 Sum of electronic and thermal Energies=             -2867.533271
 Sum of electronic and thermal Enthalpies=           -2867.532327
 Sum of electronic and thermal Free Energies=        -2867.666457
```

CPCM (Toluene) M06L/6-311++G(d,p) with LANL2DZ (for Ni, Fe) E = -2868.81452521

Cyclohexenyl-OPiv

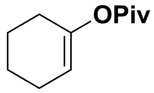

C	-1.816336000	1.278593000	-0.191696000
C	-1.097818000	0.221412000	0.186002000
C	-1.651280000	-1.146906000	0.476856000
C	-3.089076000	-1.281328000	-0.052604000
C	-3.922484000	-0.035789000	0.277588000
C	-3.313939000	1.220926000	-0.366771000
H	-1.006242000	-1.903939000	0.018661000
H	-3.056218000	-1.416585000	-1.141960000
H	-3.554865000	-2.181551000	0.365422000
H	-4.958458000	-0.165586000	-0.057901000
H	-3.956067000	0.097182000	1.367965000
H	-3.557030000	1.248910000	-1.440600000
H	-3.769572000	2.123802000	0.062265000
H	-1.620594000	-1.321969000	1.563377000
H	-1.306552000	2.221590000	-0.375651000
O	0.274977000	0.399280000	0.446807000
C	2.614746000	0.059685000	0.082761000
C	1.178222000	-0.198558000	-0.386686000
O	0.856726000	-0.838961000	-1.361638000
C	2.791972000	-0.528259000	1.501033000
H	2.108556000	-0.059133000	2.214436000
H	2.608900000	-1.609257000	1.508037000
H	3.819410000	-0.360216000	1.844356000
C	2.869831000	1.583438000	0.115248000
H	3.899556000	1.778037000	0.437253000
H	2.738897000	2.028205000	-0.878207000
H	2.190265000	2.086103000	0.809016000
C	3.582586000	-0.619568000	-0.898782000
H	3.463420000	-0.222388000	-1.911466000
H	4.616788000	-0.448919000	-0.578521000
H	3.407634000	-1.698714000	-0.945532000

```
 Zero-point correction=                           0.274456 (Hartree/Particle)
 Thermal correction to Energy=                    0.288424
 Thermal correction to Enthalpy=                  0.289368
 Thermal correction to Gibbs Free Energy=         0.233451
 Sum of electronic and zero-point Energies=           -580.186250
 Sum of electronic and thermal Energies=              -580.172282
 Sum of electronic and thermal Enthalpies=            -580.171338
 Sum of electronic and thermal Free Energies=         -580.227255
```

CPCM (Toluene) M06L/6-311++G(d,p) E = -580.549636595

TS-oa-Cyclohexenyl-OMs

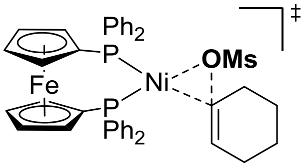

Fe	0.485711000	-2.181159000	-2.235687000
P	1.926306000	-0.137997000	0.126691000
C	-3.339148000	-0.137201000	-0.543796000
C	-2.077035000	-1.623088000	1.610597000
C	-3.385266000	0.709210000	-1.663741000
H	-2.476270000	1.060728000	-2.139321000
C	1.966972000	-0.918307000	-1.525927000
C	1.762981000	-2.071277000	2.167109000
H	0.729129000	-1.753228000	2.247808000
C	3.573649000	-3.487663000	2.921337000
H	3.949302000	-4.270320000	3.575239000
C	-1.261557000	-2.048875000	-1.090663000
C	-3.024905000	-3.536044000	2.779174000
H	-3.522676000	-4.501634000	2.744440000
C	-4.544170000	-0.510093000	0.072213000
H	-4.538367000	-1.148228000	0.948794000
C	3.343718000	1.052922000	0.082442000
C	-2.691551000	-2.963590000	4.009828000
H	-2.928467000	-3.482927000	4.934668000
C	-5.804259000	0.763669000	-1.557307000
H	-6.756945000	1.113412000	-1.946794000
C	2.606051000	-1.459458000	1.229418000
C	3.949235000	-1.867939000	1.161418000
H	4.624260000	-1.388771000	0.458178000
C	1.349166000	-0.355279000	-2.699316000
H	0.811320000	0.581877000	-2.728174000
C	2.241647000	-3.081655000	3.006041000
H	1.571919000	-3.542481000	3.727341000
C	-1.745119000	-1.061988000	2.852870000
H	-1.227345000	-0.107338000	2.875398000
C	-2.050910000	-1.724545000	4.044679000
H	-1.783704000	-1.273212000	4.996664000
C	-5.766670000	-0.060610000	-0.431763000
H	-6.689800000	-0.357322000	0.059935000
C	2.536484000	-2.181625000	-1.908348000
H	3.035978000	-2.874985000	-1.246482000
C	-1.061495000	-3.338323000	-3.010270000
H	-1.118684000	-3.657054000	-4.042971000
C	-2.721408000	-2.872266000	1.589805000
H	-2.985673000	-3.328204000	0.640745000
C	2.272679000	-2.389173000	-3.292868000
H	2.535759000	-3.272347000	-3.861033000
C	4.427406000	-2.876214000	1.998508000
H	5.468659000	-3.181407000	1.934366000
C	5.432287000	2.935889000	0.117680000
H	6.240314000	3.662606000	0.130571000
C	5.026661000	2.357704000	-1.085085000
H	5.517511000	2.632386000	-2.015232000
C	3.989384000	1.421215000	-1.104636000
H	3.680741000	0.983544000	-2.047956000
C	-4.610019000	1.146562000	-2.169996000
H	-4.618097000	1.805617000	-3.033577000
C	4.792329000	2.575827000	1.307058000
H	5.101266000	3.020216000	2.249791000
C	-0.517141000	-3.238698000	-0.770134000
H	-0.103971000	-3.484946000	0.198189000
C	3.753919000	1.645896000	1.288286000
H	3.262671000	1.374535000	2.219477000
C	-1.583159000	-2.119017000	-2.486080000
H	-2.115650000	-1.363640000	-3.046937000

C	1.546853000	-1.260834000	-3.779680000
H	1.163942000	-1.135525000	-4.784241000
C	-0.407460000	-4.032594000	-1.949814000
H	0.120795000	-4.973730000	-2.031923000
Ni	-0.096441000	0.917319000	0.548238000
P	-1.671900000	-0.672871000	0.069033000
O	-1.242113000	2.765975000	-2.965313000
S	-0.574272000	3.197400000	-1.720777000
O	-1.531686000	3.327485000	-0.537520000
O	0.627721000	2.391908000	-1.337914000
C	0.022117000	4.878760000	-1.995941000
C	-1.052813000	2.366979000	1.186862000
C	0.209113000	2.731214000	1.605477000
C	-2.325752000	2.596567000	1.943602000
C	0.449078000	3.158634000	3.048711000
H	0.973955000	2.988065000	0.878205000
C	-2.062733000	3.462874000	3.194290000
H	-3.045469000	3.080036000	1.276886000
H	-2.773037000	1.636677000	2.231861000
C	-0.800264000	2.989237000	3.930779000
H	0.743667000	4.220322000	3.047902000
H	1.297487000	2.610495000	3.480004000
H	-1.930735000	4.510061000	2.889642000
H	-2.936602000	3.427946000	3.856608000
H	-0.667652000	3.545180000	4.867145000
H	-0.920256000	1.931702000	4.208647000
H	0.731605000	4.857007000	-2.825839000
H	0.509840000	5.227346000	-1.083591000
H	-0.831303000	5.513619000	-2.241622000

```
Zero-point correction=                           0.707383 (Hartree/Particle)
 Thermal correction to Energy=                    0.753188
 Thermal correction to Enthalpy=                  0.754132
 Thermal correction to Gibbs Free Energy=         0.627192
 Sum of electronic and zero-point Energies=          -3184.962567
 Sum of electronic and thermal Energies=             -3184.916762
 Sum of electronic and thermal Enthalpies=           -3184.915818
 Sum of electronic and thermal Free Energies=        -3185.042758

CPCM (Toluene) M06L/6-311++G(d,p) with LANL2DZ (for Ni, Fe) E = -3186.12290942
```

Cyclohexenyl-OMs

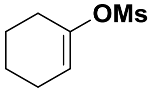

C	1.232547000	1.201555000	-0.261374000
C	0.655700000	0.016179000	-0.464533000
C	1.319527000	-1.324046000	-0.316090000
C	2.667120000	-1.189367000	0.414653000
C	3.460982000	0.019517000	-0.099838000
C	2.684728000	1.327632000	0.126070000
H	0.648268000	-1.994718000	0.232493000
H	2.479371000	-1.067031000	1.489769000
H	3.246519000	-2.112438000	0.296984000
H	4.439085000	0.074624000	0.392525000
H	3.653303000	-0.105404000	-1.174507000
H	2.751348000	1.631389000	1.182267000
H	3.144481000	2.145267000	-0.445266000
H	1.459663000	-1.770879000	-1.312205000
H	0.641670000	2.105477000	-0.379510000
O	-0.686074000	-0.040749000	-0.922421000
S	-1.866561000	0.056837000	0.238218000
C	-3.233966000	-0.318447000	-0.863410000
O	-1.687096000	-1.027019000	1.202838000
O	-1.979631000	1.436075000	0.704590000

H	-3.260477000	0.431548000	-1.654963000
H	-4.143073000	-0.272077000	-0.260116000
H	-3.092605000	-1.319743000	-1.271891000

```
Zero-point correction=                           0.189709 (Hartree/Particle)
 Thermal correction to Energy=                    0.201000
 Thermal correction to Enthalpy=                  0.201944
 Thermal correction to Gibbs Free Energy=         0.151581
 Sum of electronic and zero-point Energies=           -897.556849
 Sum of electronic and thermal Energies=              -897.545558
 Sum of electronic and thermal Enthalpies=            -897.544614
 Sum of electronic and thermal Free Energies=         -897.594977

CPCM (Toluene) M06L/6-311++G(d,p) E = -897.847901818
```

TS-oa-Cyclohexenyl-OTf

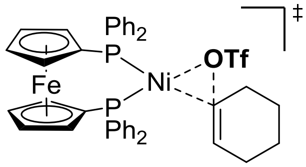

Fe	1.829638000	1.103237000	-2.561865000
C	0.987595000	2.893523000	0.980152000
C	-1.790279000	2.850729000	0.246108000
C	1.889398000	2.291728000	1.868551000
H	1.836523000	1.221572000	2.039113000
C	2.622885000	-0.417730000	-1.371432000
C	0.365818000	-3.266156000	-1.326241000
H	-0.413287000	-2.523189000	-1.467927000
C	1.166323000	-5.529544000	-1.651762000
H	1.024911000	-6.529839000	-2.052471000
C	0.205192000	2.020810000	-1.666146000
C	-3.920247000	3.600350000	-0.645414000
H	-4.634206000	3.616129000	-1.463549000
C	1.050893000	4.285991000	0.795785000
H	0.337654000	4.777666000	0.141020000
C	3.016991000	-1.336472000	1.323791000
C	-4.205254000	4.255678000	0.552273000
H	-5.140070000	4.797115000	0.670383000
C	2.915004000	4.432198000	2.334400000
H	3.659178000	5.028038000	2.856168000
C	1.530782000	-2.946705000	-0.608354000
C	2.504104000	-3.937899000	-0.410898000
H	3.405376000	-3.716242000	0.151220000
C	3.427861000	0.772553000	-1.291399000
H	3.627476000	1.343289000	-0.394563000
C	0.192658000	-4.549286000	-1.850895000
H	-0.713097000	-4.780565000	-2.404868000
C	-2.088606000	3.505812000	1.452889000
H	-1.379384000	3.484913000	2.275634000
C	-3.285560000	4.206576000	1.602113000
H	-3.497833000	4.714222000	2.539633000
C	2.010096000	5.047132000	1.464024000
H	2.047107000	6.122420000	1.310280000
C	2.604899000	-0.824936000	-2.746553000
H	2.060093000	-1.670547000	-3.142933000
C	1.106899000	2.704499000	-3.689571000
H	1.697473000	3.237606000	-4.423807000
C	-2.720481000	2.904216000	-0.800519000
H	-2.529147000	2.389012000	-1.735012000
C	3.394754000	0.093493000	-3.498008000
H	3.543539000	0.069446000	-4.569693000
C	2.319501000	-5.221679000	-0.927107000

H	3.079025000	-5.981247000	-0.760738000
C	4.894947000	-1.549747000	3.407965000
H	5.621585000	-1.630352000	4.211960000
C	5.321374000	-1.523806000	2.078079000
H	6.381042000	-1.584584000	1.844491000
C	4.390811000	-1.417342000	1.042741000
H	4.736053000	-1.390352000	0.013831000
C	2.849918000	3.053843000	2.539760000
H	3.539859000	2.566710000	3.223607000
C	3.532298000	-1.466097000	3.699949000
H	3.192499000	-1.480260000	4.732192000
C	-0.243448000	1.117214000	-2.693134000
H	-0.878048000	0.255415000	-2.529701000
C	2.601467000	-1.357460000	2.664842000
H	1.542137000	-1.276632000	2.891245000
C	1.043752000	3.002508000	-2.298139000
H	1.576658000	3.797604000	-1.796584000
C	3.908873000	1.074639000	-2.599233000
H	4.518379000	1.928073000	-2.866840000
C	0.310767000	1.545679000	-3.931867000
H	0.184951000	1.047840000	-4.884612000
P	1.716380000	-1.212706000	0.015736000
P	-0.249147000	1.840908000	0.095115000
Ni	-0.291774000	-0.322811000	0.786384000
O	-2.119959000	-1.161675000	-1.304975000
S	-3.458136000	-0.722244000	-0.829266000
O	-3.439884000	-0.252960000	0.606342000
O	-4.231395000	0.131711000	-1.735578000
C	-4.421725000	-2.311941000	-0.729120000
F	-4.534940000	-2.860282000	-1.945635000
F	-5.645787000	-2.092826000	-0.238017000
F	-3.794083000	-3.192128000	0.072551000
C	-1.784596000	-0.552771000	1.832018000
C	-2.353917000	0.091586000	3.050446000
C	-1.744920000	-0.601193000	4.295633000
C	-1.883506000	-2.126913000	4.199689000
C	-1.125342000	-2.705025000	2.990060000
C	-1.336082000	-1.853132000	1.735290000
H	-2.170612000	1.169950000	3.067089000
H	-3.440380000	-0.052982000	3.051636000
H	-2.251995000	-0.222784000	5.191634000
H	-0.684904000	-0.326044000	4.391193000
H	-1.526676000	-2.600511000	5.123130000
H	-2.949590000	-2.375847000	4.108647000
H	-1.468474000	-3.727933000	2.791525000
H	-0.054930000	-2.788930000	3.223500000
H	-1.426313000	-2.379746000	0.788410000

```
Zero-point correction=                           0.684176 (Hartree/Particle)
 Thermal correction to Energy=                    0.732232
 Thermal correction to Enthalpy=                  0.733176
 Thermal correction to Gibbs Free Energy=         0.599765
 Sum of electronic and zero-point Energies=          -3482.692007
 Sum of electronic and thermal Energies=             -3482.643951
 Sum of electronic and thermal Enthalpies=           -3482.643007
 Sum of electronic and thermal Free Energies=        -3482.776418
```

CPCM (Toluene) M06L/6-311++G(d,p) with LANL2DZ (for Ni, Fe) E = -3483.8979038

Cyclohexenyl-OTf

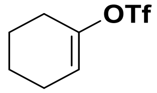

C	2.023587000	-1.044380000	-0.630044000
C	1.359590000	0.090877000	-0.416916000
C	1.940417000	1.388773000	0.057357000
C	3.476517000	1.358139000	-0.035514000
C	4.034677000	0.021284000	0.472632000
C	3.503583000	-1.156256000	-0.361883000
H	1.529531000	2.207502000	-0.547284000
H	3.777754000	1.502374000	-1.081929000
H	3.896737000	2.195054000	0.533416000
H	5.130514000	0.030166000	0.450190000
H	3.739050000	-0.117575000	1.521199000
H	4.037677000	-1.214565000	-1.322888000
H	3.708112000	-2.106882000	0.147738000
H	1.610999000	1.573639000	1.089628000
H	1.490369000	-1.913931000	-1.004615000
O	-0.039940000	0.152506000	-0.699328000
S	-1.034796000	-0.522691000	0.413448000
C	-2.569923000	0.347244000	-0.210144000
F	-3.600190000	-0.096636000	0.508728000
F	-2.443457000	1.661520000	-0.048757000
F	-2.763052000	0.065883000	-1.495280000
O	-1.196169000	-1.947159000	0.161934000
O	-0.728192000	-0.018312000	1.745422000

```
Zero-point correction=                           0.166000 (Hartree/Particle)
 Thermal correction to Energy=                   0.179506
 Thermal correction to Enthalpy=                 0.180450
 Thermal correction to Gibbs Free Energy=        0.123571
 Sum of electronic and zero-point Energies=         -1195.284369
 Sum of electronic and thermal Energies=            -1195.270864
 Sum of electronic and thermal Enthalpies=          -1195.269919
 Sum of electronic and thermal Free Energies=       -1195.326798
```

CPCM (Toluene) M06L/6-311++G(d,p) E = -1195.60341418

TS-oa-Cyclohexenyl-ONf

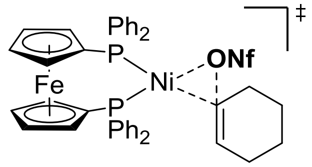

Fe	2.437466000	-0.510213000	-2.880432000
C	3.808855000	1.704970000	0.213869000
C	1.534156000	3.427857000	-0.106270000
C	4.328859000	0.750491000	1.098997000
H	3.667736000	0.003980000	1.527070000
C	2.412151000	-2.051506000	-1.477110000
C	-1.042042000	-2.718760000	-0.451209000
H	-1.210600000	-1.660667000	-0.624424000
C	-1.907550000	-4.979966000	-0.399264000
H	-2.730027000	-5.677001000	-0.536633000
C	2.003064000	1.321123000	-2.022591000
C	0.199991000	5.312957000	-0.854857000
H	-0.523168000	5.724136000	-1.552996000
C	4.673938000	2.683965000	-0.305702000
H	4.284737000	3.456724000	-0.961826000
C	2.798038000	-2.773978000	1.274351000
C	0.696605000	6.094849000	0.187850000

H	0.370927000	7.125341000	0.301650000
C	6.537180000	1.717533000	0.901788000
H	7.591367000	1.723452000	1.165785000
C	0.217285000	-3.179230000	-0.032125000
C	0.396455000	-4.550403000	0.208446000
H	1.357068000	-4.926461000	0.545158000
C	3.761303000	-1.649047000	-1.777380000
H	4.473847000	-1.243742000	-1.071939000
C	-2.095121000	-3.617888000	-0.638864000
H	-3.063835000	-3.242963000	-0.955265000
C	2.023232000	4.219345000	0.946782000
H	2.742621000	3.808650000	1.649841000
C	1.610073000	5.544025000	1.089420000
H	2.003696000	6.144854000	1.905346000
C	6.027428000	2.686057000	0.031958000
H	6.683693000	3.448804000	-0.378863000
C	1.819906000	-2.495442000	-2.705850000
H	0.799381000	-2.832183000	-2.823758000
C	2.554688000	1.054908000	-4.258664000
H	3.124799000	1.006663000	-5.177799000
C	0.615991000	3.988899000	-1.003866000
H	0.203856000	3.396046000	-1.812716000
C	2.791036000	-2.376499000	-3.742369000
H	2.630054000	-2.602843000	-4.788401000
C	-0.661075000	-5.443088000	0.027503000
H	-0.509452000	-6.501477000	0.223289000
C	4.581676000	-3.986238000	3.080907000
H	5.272309000	-4.454481000	3.777100000
C	4.594859000	-4.347328000	1.731541000
H	5.295917000	-5.097488000	1.375223000
C	3.710866000	-3.746182000	0.833575000
H	3.736413000	-4.029239000	-0.214133000
C	5.684711000	0.752638000	1.439089000
H	6.067194000	0.002228000	2.125776000
C	3.681591000	-3.018118000	3.529923000
H	3.668091000	-2.727977000	4.577217000
C	0.848967000	0.816972000	-2.720033000
H	-0.103046000	0.585560000	-2.259932000
C	2.799077000	-2.415102000	2.631606000
H	2.107939000	-1.651862000	2.976691000
C	3.056731000	1.464480000	-2.990038000
H	4.070538000	1.776172000	-2.783020000
C	3.991317000	-1.862228000	-3.168277000
H	4.903285000	-1.626160000	-3.701056000
C	1.194247000	0.659784000	-4.091494000
H	0.546938000	0.264442000	-4.863442000
P	1.581912000	-1.939845000	0.158555000
P	2.013473000	1.645723000	-0.224360000
Ni	0.823305000	0.111732000	0.962168000
O	-1.609391000	0.502567000	-0.613372000
S	-2.202698000	1.745021000	-0.057554000
O	-1.521945000	2.195852000	1.215799000
O	-2.486420000	2.828869000	-1.000559000
C	-0.185612000	1.012178000	2.210052000
C	0.108219000	1.969499000	3.316551000
C	0.474955000	1.153275000	4.580831000
C	-0.589577000	0.085955000	4.870885000
C	-0.703470000	-0.946097000	3.733345000
C	-0.662680000	-0.276055000	2.356718000
H	0.906329000	2.668367000	3.049363000
H	-0.789787000	2.563935000	3.520615000
H	0.571765000	1.842657000	5.428453000
H	1.456825000	0.678344000	4.444271000
H	-0.369555000	-0.425654000	5.816499000
H	-1.558554000	0.586938000	5.000595000
H	-1.643071000	-1.503775000	3.832430000
H	0.097101000	-1.692986000	3.823490000
H	-1.302645000	-0.697531000	1.586270000
C	-3.884389000	1.183889000	0.581860000
F	-4.638477000	2.278955000	0.818819000

F	-3.701687000	0.519754000	1.747172000
C	-4.648359000	0.241260000	-0.384431000
F	-4.090621000	-0.996382000	-0.306803000
F	-4.543345000	0.693716000	-1.651828000
C	-6.161774000	0.101912000	-0.056190000
F	-6.322093000	-0.055732000	1.279877000
F	-6.800275000	1.223403000	-0.452592000
C	-6.856259000	-1.100630000	-0.748177000
F	-6.588643000	-1.110465000	-2.061113000
F	-8.180997000	-0.994778000	-0.579970000
F	-6.449436000	-2.257672000	-0.211442000

Zero-point correction= 0.721496 (Hartree/Particle)
Thermal correction to Energy= 0.777956
Thermal correction to Enthalpy= 0.778900
Thermal correction to Gibbs Free Energy= 0.626670
Sum of electronic and zero-point Energies= -4195.990227
Sum of electronic and thermal Energies= -4195.933767
Sum of electronic and thermal Enthalpies= -4195.932822
Sum of electronic and thermal Free Energies= -4196.085052

CPCM (Toluene) M06L/6-311++G(d,p) with LANL2DZ (for Ni, Fe) E = -4197.38083319

Cyclohexenyl-ONf

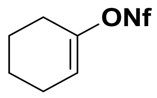

C	3.213701000	-1.190317000	-0.759361000
C	3.282362000	0.100056000	-0.433917000
C	4.398319000	0.769749000	0.313340000
C	5.627545000	-0.154127000	0.387212000
C	5.218205000	-1.594087000	0.727374000
C	4.280602000	-2.172439000	-0.345747000
H	4.648826000	1.711119000	-0.192133000
H	6.145985000	-0.143999000	-0.581089000
H	6.334431000	0.232865000	1.129686000
H	6.104352000	-2.231005000	0.830002000
H	4.705116000	-1.603778000	1.698440000
H	4.856715000	-2.470806000	-1.235378000
H	3.804791000	-3.092011000	0.019962000
H	4.050851000	1.041655000	1.319113000
H	2.370932000	-1.547845000	-1.344691000
O	2.269948000	0.978157000	-0.923652000
S	1.045982000	1.439936000	0.055394000
C	-0.178934000	0.038017000	-0.291310000
F	-0.080213000	-0.294326000	-1.588752000
F	0.177193000	-1.013152000	0.470590000
O	1.434130000	1.342093000	1.454288000
O	0.496888000	2.643658000	-0.539017000
C	-1.641773000	0.451378000	0.026696000
C	-2.605408000	-0.763159000	0.149214000
C	-4.110220000	-0.390056000	0.089903000
F	-4.376041000	0.601581000	0.949810000
F	-4.834781000	-1.463450000	0.426669000
F	-4.451520000	-0.008195000	-1.144116000
F	-2.376177000	-1.378955000	1.327722000
F	-2.353771000	-1.627552000	-0.863309000
F	-1.655051000	1.123381000	1.199898000
F	-2.079842000	1.258832000	-0.961133000

Zero-point correction= 0.203062 (Hartree/Particle)
Thermal correction to Energy= 0.225067
Thermal correction to Enthalpy= 0.226011

```
 Thermal correction to Gibbs Free Energy=         0.149402
 Sum of electronic and zero-point Energies=          -1908.567241
 Sum of electronic and thermal Energies=             -1908.545236
 Sum of electronic and thermal Enthalpies=           -1908.544292
 Sum of electronic and thermal Free Energies=        -1908.620901
```

```
CPCM (Toluene) M06L/6-311++G(d,p) E = -1909.08535185
```

TS-oa-Phenyl trifluoromethyl ketone

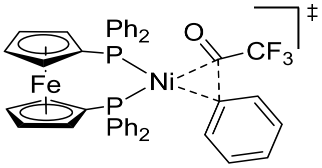

Fe	-0.666530000	-2.908362000	-1.252377000
P	1.701827000	-0.690376000	-0.156581000
C	-3.214911000	0.657121000	-0.852357000
C	-2.591113000	0.047927000	1.943349000
C	-2.953202000	0.787744000	-2.225954000
H	-1.969664000	0.541442000	-2.614798000
C	1.174650000	-1.995129000	-1.325302000
C	1.321714000	-1.331800000	2.547449000
H	0.524858000	-0.594818000	2.495214000
C	2.606462000	-2.933375000	3.826529000
H	2.807504000	-3.448819000	4.761915000
C	-2.006943000	-1.805906000	-0.126182000
C	-4.028364000	-0.859138000	3.683637000
H	-4.738513000	-1.613028000	4.013626000
C	-4.475034000	1.029631000	-0.366206000
H	-4.694297000	0.951863000	0.693345000
C	3.401729000	-0.294684000	-0.779907000
C	-3.632205000	0.161375000	4.551881000
H	-4.032766000	0.203499000	5.561517000
C	-5.193144000	1.616107000	-2.603874000
H	-5.957609000	1.989808000	-3.279762000
C	2.090450000	-1.600954000	1.405059000
C	3.138592000	-2.532061000	1.499112000
H	3.769759000	-2.724287000	0.636302000
C	0.430357000	-1.709428000	-2.524434000
H	0.120779000	-0.723856000	-2.847086000
C	1.575502000	-1.996069000	3.750621000
H	0.970929000	-1.774132000	4.625862000
C	-2.205189000	1.076319000	2.818738000
H	-1.532787000	1.857494000	2.476570000
C	-2.726426000	1.128202000	4.114580000
H	-2.423357000	1.933130000	4.778973000
C	-5.455225000	1.510076000	-1.237827000
H	-6.425257000	1.802065000	-0.844224000
C	1.338998000	-3.421452000	-1.265691000
H	1.814889000	-3.967422000	-0.463242000
C	-2.525965000	-3.848148000	-1.102257000
H	-2.895215000	-4.587929000	-1.800739000
C	-3.512617000	-0.916231000	2.388667000
H	-3.826957000	-1.715457000	1.724942000
C	0.699479000	-3.996411000	-2.402056000
H	0.610335000	-5.055015000	-2.609644000
C	3.389889000	-3.197497000	2.699286000
H	4.202914000	-3.916509000	2.757327000
C	5.975986000	0.391510000	-1.683870000
H	6.971810000	0.653106000	-2.031859000
C	5.068010000	-0.219476000	-2.549173000
H	5.353343000	-0.438714000	-3.574876000
C	3.789948000	-0.562152000	-2.101180000
H	3.100473000	-1.048056000	-2.784170000
C	-3.937718000	1.252885000	-3.096875000

H	-3.718145000	1.347775000	-4.157027000
C	5.595374000	0.668611000	-0.368907000
H	6.290710000	1.153585000	0.310922000
C	-1.420030000	-2.856504000	0.663140000
H	-0.814432000	-2.719708000	1.548007000
C	4.317569000	0.335061000	0.078119000
H	4.032060000	0.573316000	1.098384000
C	-2.694358000	-2.437450000	-1.216904000
H	-3.220866000	-1.922122000	-2.007613000
C	0.142132000	-2.939755000	-3.181972000
H	-0.437593000	-3.054577000	-4.088566000
C	-1.746566000	-4.107206000	0.063631000
H	-1.415798000	-5.078509000	0.408039000
Ni	0.151726000	1.050394000	0.106912000
P	-1.871151000	-0.002494000	0.236277000
C	-0.447958000	2.850057000	0.214496000
O	-1.019376000	3.426296000	1.144992000
C	1.400588000	2.596403000	0.457433000
C	2.289027000	3.085363000	-0.516772000
C	1.753043000	2.762413000	1.813583000
C	3.483218000	3.714075000	-0.150515000
C	2.958066000	3.353712000	2.180104000
C	3.825654000	3.843315000	1.194447000
H	2.046589000	2.993405000	-1.569845000
H	1.043334000	2.470931000	2.583215000
H	4.147420000	4.096322000	-0.921701000
H	3.210530000	3.464994000	3.232184000
H	4.752555000	4.335092000	1.479085000
C	-0.711082000	3.463914000	-1.191948000
F	-0.132602000	4.670911000	-1.350816000
F	-0.240554000	2.664678000	-2.198261000
F	-2.029233000	3.617549000	-1.397544000

```
Zero-point correction=                           0.632593 (Hartree/Particle)
 Thermal correction to Energy=                    0.677021
 Thermal correction to Enthalpy=                  0.677965
 Thermal correction to Gibbs Free Energy=         0.552218
 Sum of electronic and zero-point Energies=          -2969.878588
 Sum of electronic and thermal Energies=             -2969.834161
 Sum of electronic and thermal Enthalpies=           -2969.833216
 Sum of electronic and thermal Free Energies=        -2969.958963

CPCM (Toluene) M06L/6-311++G(d,p) with LANL2DZ (for Ni, Fe) E = -2970.97873962
```

Phenyl trifluoromethyl ketone

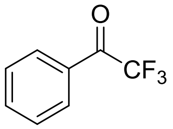

C	-0.659975000	0.307242000	-0.000065000
C	-1.678012000	1.277511000	0.000142000
C	-1.003819000	-1.055856000	-0.000206000
H	-1.393041000	2.324406000	0.000216000
H	-0.236486000	-1.820283000	-0.000350000
C	-3.014277000	0.894541000	0.000226000
C	-2.345328000	-1.433818000	-0.000185000
H	-3.795243000	1.649512000	0.000428000
H	-2.606691000	-2.488043000	-0.000353000
C	-3.349809000	-0.463527000	0.000054000
H	-4.393939000	-0.764620000	0.000098000
C	0.740231000	0.801570000	-0.000185000
O	1.040456000	1.979028000	-0.000449000
C	1.896511000	-0.236786000	0.000140000
F	1.824518000	-1.031913000	-1.091635000
F	3.083944000	0.366682000	0.000741000
F	1.823607000	-1.032376000	1.091342000

```
Zero-point correction=                           0.115541 (Hartree/Particle)
 Thermal correction to Energy=                    0.125289
 Thermal correction to Enthalpy=                  0.126233
 Thermal correction to Gibbs Free Energy=         0.079074
 Sum of electronic and zero-point Energies=           -682.478802
 Sum of electronic and thermal Energies=              -682.469054
 Sum of electronic and thermal Enthalpies=            -682.468110
 Sum of electronic and thermal Free Energies=         -682.515269

CPCM (Toluene) M06L/6-311++G(d,p) E = -682.718796064
```

TS-oa-Acetophenone

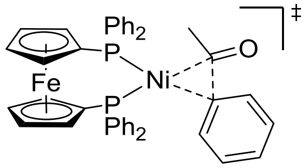

Fe	0.763109000	-2.955401000	0.550092000
P	-1.659935000	-0.560971000	0.078472000
C	3.193132000	0.669121000	1.248762000
C	2.661375000	0.848028000	-1.613895000
C	2.945365000	0.322714000	2.588319000
H	2.012196000	-0.166179000	2.855688000
C	-1.110419000	-2.141173000	0.821407000
C	-1.230845000	-0.429205000	-2.691527000
H	-0.439198000	0.263489000	-2.418968000
C	-2.489853000	-1.606431000	-4.388773000
H	-2.672811000	-1.840634000	-5.434180000
C	2.102498000	-1.544038000	-0.173037000
C	4.142576000	0.517822000	-3.515217000
H	4.861921000	-0.101017000	-4.045584000
C	4.388970000	1.329422000	0.937013000
H	4.596546000	1.620496000	-0.086974000
C	-3.376795000	-0.408854000	0.761309000
C	3.766616000	1.759353000	-4.033379000
H	4.191155000	2.109755000	-4.970775000
C	5.071068000	1.259591000	3.261193000
H	5.796004000	1.489791000	4.037302000
C	-2.020762000	-1.001788000	-1.683398000
C	-3.066309000	-1.864914000	-2.051584000
H	-3.712595000	-2.287625000	-1.287507000
C	-0.408998000	-2.207159000	2.076672000
H	-0.137655000	-1.353349000	2.683658000
C	-1.460828000	-0.732064000	-4.036310000
H	-0.840449000	-0.278349000	-4.804592000
C	2.297719000	2.101495000	-2.134953000
H	1.621261000	2.743752000	-1.577770000
C	2.851071000	2.549808000	-3.337473000
H	2.564978000	3.523955000	-3.725498000
C	5.318402000	1.624814000	1.937869000
H	6.237692000	2.143152000	1.678210000
C	-1.226599000	-3.489124000	0.338145000
H	-1.660858000	-3.782261000	-0.607255000
C	2.655155000	-3.768682000	0.208175000
H	3.029130000	-4.666814000	0.682674000
C	3.596737000	0.065792000	-2.313076000
H	3.899439000	-0.899958000	-1.921252000
C	-0.601331000	-4.360965000	1.276704000
H	-0.484371000	-5.431419000	1.166006000
C	-3.294683000	-2.169189000	-3.394046000
H	-4.106277000	-2.839697000	-3.664735000
C	-5.986566000	-0.070305000	1.758637000
H	-6.995487000	0.058903000	2.141719000
C	-5.136607000	-1.020302000	2.325362000
H	-5.480356000	-1.637746000	3.151442000

C	-3.841162000	-1.190595000	1.829714000
H	-3.196947000	-1.941941000	2.274743000
C	3.880164000	0.604175000	3.584571000
H	3.672608000	0.323622000	4.614029000
C	-5.530969000	0.716962000	0.698296000
H	-6.180591000	1.467269000	0.255665000
C	1.571159000	-2.343416000	-1.245373000
H	0.984878000	-1.977830000	-2.076791000
C	-4.235751000	0.555531000	0.208268000
H	-3.892129000	1.187282000	-0.605662000
C	2.776664000	-2.443674000	0.719881000
H	3.268474000	-2.164501000	1.640585000
C	-0.100646000	-3.570272000	2.353745000
H	0.457535000	-3.935112000	3.206197000
C	1.918159000	-3.705468000	-1.010774000
H	1.626350000	-4.547158000	-1.625527000
Ni	-0.151505000	1.183971000	0.346442000
P	1.895629000	0.285776000	-0.020374000
C	0.450266000	2.930784000	0.908507000
O	1.099670000	3.738941000	0.238375000
C	-1.336251000	2.821261000	0.406488000
C	-2.288088000	3.060234000	1.414010000
C	-1.594107000	3.360213000	-0.873228000
C	-3.446841000	3.801402000	1.159445000
C	-2.765836000	4.060700000	-1.143260000
C	-3.695655000	4.294992000	-0.120485000
H	-2.124572000	2.675934000	2.416925000
H	-0.839804000	3.261835000	-1.649324000
H	-4.158832000	3.981445000	1.961571000
H	-2.944949000	4.455179000	-2.141122000
H	-4.595822000	4.870558000	-0.321703000
C	0.611149000	2.934848000	2.434765000
H	0.235127000	3.880264000	2.845148000
H	0.084991000	2.107383000	2.923638000
H	1.677881000	2.864492000	2.663962000

```
Zero-point correction=                           0.655582 (Hartree/Particle)
 Thermal correction to Energy=                    0.698055
 Thermal correction to Enthalpy=                  0.698999
 Thermal correction to Gibbs Free Energy=         0.578215
 Sum of electronic and zero-point Energies=          -2672.134018
 Sum of electronic and thermal Energies=             -2672.091545
 Sum of electronic and thermal Enthalpies=           -2672.090601
 Sum of electronic and thermal Free Energies=        -2672.211385
```

CPCM (Toluene) M06L/6-311++G(d,p) with LANL2DZ (for Ni, Fe) E = -2673.19350977

Acetophenone

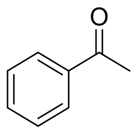

C	-0.205352000	-0.053656000	-0.000113000
C	0.576993000	-1.219859000	0.000016000
C	0.432534000	1.196267000	-0.000100000
H	0.067277000	-2.177826000	0.000042000
H	-0.152916000	2.110688000	-0.000151000
C	1.966064000	-1.138710000	0.000079000
C	1.825395000	1.277733000	-0.000027000
H	2.563041000	-2.046695000	0.000156000
H	2.310460000	2.250028000	-0.000030000
C	2.593348000	0.111567000	0.000035000
H	3.678275000	0.176046000	0.000088000
C	-1.697937000	-0.203639000	-0.000037000

O	-2.212801000	-1.311776000	-0.000078000
C	-2.562335000	1.047575000	0.000125000
H	-2.362291000	1.665957000	-0.883177000
H	-3.611406000	0.746799000	0.000067000
H	-2.362294000	1.665544000	0.883765000

```
Zero-point correction=                           0.138549 (Hartree/Particle)
 Thermal correction to Energy=                   0.146324
 Thermal correction to Enthalpy=                 0.147268
 Thermal correction to Gibbs Free Energy=        0.105935
 Sum of electronic and zero-point Energies=          -384.749860
 Sum of electronic and thermal Energies=             -384.742085
 Sum of electronic and thermal Enthalpies=           -384.741141
 Sum of electronic and thermal Free Energies=        -384.782474
```

CPCM (Toluene) M06L/6-311++G(d,p) E = -384.946833865

TS-oa-Benzonitrile

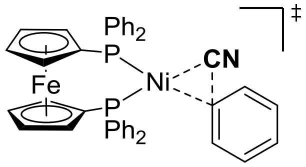

Fe	-0.821311000	-2.870166000	-0.133451000
P	1.661109000	-0.497535000	-0.065135000
C	-2.986576000	0.464297000	-1.509068000
C	-2.984427000	1.232496000	1.281591000
C	-2.370539000	0.681304000	-2.749066000
H	-1.298145000	0.847042000	-2.790150000
C	1.026615000	-2.114517000	-0.639636000
C	1.462572000	-0.147570000	2.711558000
H	0.658385000	0.535750000	2.449780000
C	2.842748000	-1.213720000	4.387095000
H	3.108505000	-1.370300000	5.429157000
C	-2.127274000	-1.392682000	0.486654000
C	-4.392075000	1.253415000	3.265762000
H	-4.883518000	0.710107000	4.069038000
C	-4.381567000	0.313255000	-1.459388000
H	-4.876350000	0.168838000	-0.503000000
C	3.311608000	-0.419679000	-0.902645000
C	-4.526168000	2.638229000	3.170182000
H	-5.120827000	3.181765000	3.899846000
C	-4.513455000	0.557071000	-3.863308000
H	-5.105574000	0.596721000	-4.773831000
C	2.159351000	-0.809921000	1.689573000
C	3.221136000	-1.661017000	2.037867000
H	3.797334000	-2.152514000	1.259083000
C	0.205806000	-2.260681000	-1.813514000
H	-0.131245000	-1.450851000	-2.446858000
C	1.798623000	-0.350094000	4.052577000
H	1.249942000	0.172506000	4.831619000
C	-3.124849000	2.629831000	1.192415000
H	-2.637937000	3.174989000	0.389840000
C	-3.893045000	3.321613000	2.128659000
H	-3.994567000	4.400247000	2.041884000
C	-5.139365000	0.357016000	-2.629795000
H	-6.218941000	0.241901000	-2.578267000
C	1.176155000	-3.424546000	-0.068319000
H	1.701790000	-3.653309000	0.848256000
C	-2.651584000	-3.656406000	0.496982000
H	-3.034238000	-4.622921000	0.195525000
C	-3.629071000	0.553323000	2.326881000
H	-3.543338000	-0.524614000	2.413268000
C	0.454111000	-4.352539000	-0.873520000
H	0.337818000	-5.409553000	-0.671281000

C	3.555491000	-1.865926000	3.377051000
H	4.378249000	-2.528761000	3.632210000
C	5.817858000	-0.185082000	-2.159887000
H	6.787239000	-0.096559000	-2.643280000
C	4.888929000	-1.121098000	-2.615572000
H	5.131540000	-1.767924000	-3.454836000
C	3.644565000	-1.239554000	-1.991376000
H	2.937543000	-1.980289000	-2.351140000
C	-3.129025000	0.721920000	-3.920952000
H	-2.638804000	0.898744000	-4.874491000
C	5.492475000	0.640099000	-1.080815000
H	6.205305000	1.378010000	-0.722418000
C	-1.484893000	-2.011660000	1.617251000
H	-0.831215000	-1.518350000	2.322941000
C	4.247930000	0.530135000	-0.461668000
H	4.007281000	1.187601000	0.368716000
C	-2.850918000	-2.427994000	-0.197674000
H	-3.413576000	-2.299212000	-1.111260000
C	-0.140460000	-3.635279000	-1.954352000
H	-0.784705000	-4.051289000	-2.717922000
C	-1.814874000	-3.397953000	1.622641000
H	-1.449233000	-4.132147000	2.328744000
Ni	0.153814000	1.240933000	-0.290567000
P	-1.938713000	0.371943000	0.010821000
C	-0.322789000	2.941160000	-0.871369000
N	-1.042201000	3.739323000	-1.373631000
C	1.292519000	2.917118000	-0.432117000
C	2.226583000	3.123315000	-1.471119000
C	1.599140000	3.417303000	0.853636000
C	3.421169000	3.794058000	-1.228073000
C	2.810077000	4.065652000	1.094429000
C	3.723943000	4.263925000	0.055134000
H	1.990884000	2.779898000	-2.473989000
H	0.868610000	3.321946000	1.652238000
H	4.124227000	3.946739000	-2.043127000
H	3.030081000	4.438543000	2.091976000
H	4.655526000	4.792366000	0.239241000

```
Zero-point correction=                           0.616810 (Hartree/Particle)
 Thermal correction to Energy=                    0.657622
 Thermal correction to Enthalpy=                  0.658566
 Thermal correction to Gibbs Free Energy=         0.540379
 Sum of electronic and zero-point Energies=          -2611.781481
 Sum of electronic and thermal Energies=             -2611.740670
 Sum of electronic and thermal Enthalpies=           -2611.739725
 Sum of electronic and thermal Free Energies=        -2611.857912

CPCM (Toluene) M06L/6-311++G(d,p) with LANL2DZ (for Ni, Fe) E = -2612.78756881
```

Benzonitrile

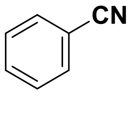

C	0.000000000	1.217722000	-0.091405000
C	0.000000000	0.000000000	0.610564000
C	0.000000000	1.211158000	-1.484263000
H	0.000000000	2.152259000	-2.026401000
C	0.000000000	-1.217722000	-0.091405000
C	0.000000000	0.000000000	-2.181100000
H	0.000000000	-2.153041000	0.458871000
H	0.000000000	0.000000000	-3.267491000
C	0.000000000	-1.211158000	-1.484263000
H	0.000000000	-2.152259000	-2.026401000
H	0.000000000	2.153041000	0.458871000
C	0.000000000	0.000000000	2.045411000
N	0.000000000	0.000000000	3.208759000

```
Zero-point correction=                           0.099482 (Hartree/Particle)
 Thermal correction to Energy=                    0.105579
 Thermal correction to Enthalpy=                  0.106523
 Thermal correction to Gibbs Free Energy=         0.069210
 Sum of electronic and zero-point Energies=           -324.385245
 Sum of electronic and thermal Energies=              -324.379148
 Sum of electronic and thermal Enthalpies=            -324.378204
 Sum of electronic and thermal Free Energies=         -324.415517

CPCM (Toluene) M06L/6-311++G(d,p) E = -324.532572881
```

TS-oa-Dimethylbenzylamine

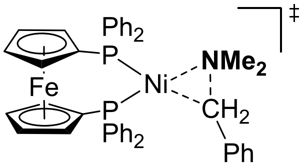

Ni	-0.323634000	0.727185000	0.640799000
Fe	1.586233000	-2.087219000	-1.861555000
P	-1.148064000	-1.318463000	0.162770000
P	1.846761000	0.789270000	0.004802000
C	-2.669024000	-1.388727000	-0.904181000
C	-1.639237000	-2.343434000	1.637702000
C	-2.582786000	-0.972583000	-2.246433000
H	-1.619203000	-0.691751000	-2.662087000
C	2.277949000	-0.161599000	-1.513352000
C	2.544237000	-0.543930000	2.372027000
H	1.466611000	-0.632622000	2.486084000
C	4.784139000	-0.942848000	3.194774000
H	5.453677000	-1.353998000	3.945858000
C	-0.055158000	-2.561005000	-0.668774000
C	-2.337244000	-4.431134000	2.672449000
H	-2.622878000	-5.475740000	2.576976000
C	-3.936426000	-1.711207000	-0.393415000
H	-4.038627000	-2.028880000	0.639063000
C	2.603451000	2.447814000	-0.377280000
C	-2.292628000	-3.829507000	3.934204000
H	-2.544256000	-4.405629000	4.821003000
C	-4.971694000	-1.239043000	-2.534127000
H	-5.857448000	-1.184265000	-3.161270000
C	3.053736000	0.122675000	1.247963000
C	4.446186000	0.264418000	1.123343000
H	4.860426000	0.803126000	0.275739000
C	1.428460000	-0.194073000	-2.674562000
H	0.493889000	0.342084000	-2.776416000
C	3.402316000	-1.078187000	3.337145000
H	2.989551000	-1.593016000	4.200900000
C	-1.593828000	-1.752545000	2.907687000
H	-1.290787000	-0.712254000	2.990476000
C	-1.919218000	-2.490652000	4.050058000
H	-1.877170000	-2.018493000	5.028479000
C	-5.075298000	-1.633489000	-1.199805000
H	-6.045458000	-1.888494000	-0.780646000
C	3.379292000	-1.042532000	-1.785738000
H	4.174680000	-1.285964000	-1.095558000
C	0.959325000	-4.059765000	-2.127680000
H	1.170948000	-4.648692000	-3.011074000
C	-2.010311000	-3.695186000	1.534128000
H	-2.041145000	-4.173238000	0.558785000
C	3.206397000	-1.600311000	-3.086920000
H	3.851545000	-2.335051000	-3.551770000
C	5.304298000	-0.266222000	2.087802000
H	6.379344000	-0.147861000	1.977572000

C	3.509335000	5.076244000	-0.873812000
H	3.859840000	6.086797000	-1.065898000
C	3.092911000	4.262320000	-1.927991000
H	3.121555000	4.635483000	-2.948778000
C	2.644708000	2.961873000	-1.683199000
H	2.337320000	2.342158000	-2.520016000
C	-3.717468000	-0.910016000	-3.055442000
H	-3.622893000	-0.594090000	-4.091362000
C	3.473747000	4.578217000	0.431003000
H	3.801415000	5.198937000	1.261331000
C	1.167456000	-3.031420000	-0.075306000
H	1.570327000	-2.711077000	0.875836000
C	3.021768000	3.281361000	0.676185000
H	3.010613000	2.907453000	1.696591000
C	-0.173069000	-3.211312000	-1.942382000
H	-0.970622000	-3.061289000	-2.655952000
C	2.003138000	-1.071300000	-3.640188000
H	1.576963000	-1.325957000	-4.601985000
C	1.782811000	-3.955734000	-0.968369000
H	2.732885000	-4.450410000	-0.812837000
C	-1.179178000	2.547770000	-0.079368000
H	-0.392687000	3.290842000	0.022601000
H	-0.960993000	1.844197000	-0.905153000
N	-1.092641000	1.867926000	1.925911000
C	-2.453819000	1.745096000	2.422928000
C	-0.417505000	2.985639000	2.567051000
H	-2.994307000	0.972222000	1.874434000
H	-3.024400000	2.686600000	2.362845000
H	0.570604000	3.138669000	2.130447000
H	-0.989520000	3.929963000	2.504936000
C	-2.531196000	3.118885000	-0.188281000
C	-2.772972000	4.462587000	0.161627000
C	-3.616230000	2.357229000	-0.665147000
C	-4.045931000	5.019941000	0.045172000
C	-4.887903000	2.913880000	-0.778859000
C	-5.111547000	4.247279000	-0.422605000
H	-1.946246000	5.076010000	0.511461000
H	-3.452435000	1.322181000	-0.948024000
H	-4.204912000	6.060826000	0.315940000
H	-5.706425000	2.303834000	-1.152480000
H	-6.103689000	4.681070000	-0.515607000
H	-0.282916000	2.761678000	3.637614000
H	-2.421368000	1.450760000	3.484833000

```
Zero-point correction=                           0.718324 (Hartree/Particle)
 Thermal correction to Energy=                    0.762968
 Thermal correction to Enthalpy=                  0.763912
 Thermal correction to Gibbs Free Energy=         0.638359
 Sum of electronic and zero-point Energies=          -2692.670520
 Sum of electronic and thermal Energies=             -2692.625876
 Sum of electronic and thermal Enthalpies=           -2692.624932
 Sum of electronic and thermal Free Energies=        -2692.750484

CPCM (Toluene) M06L/6-311++G(d,p) with LANL2DZ (for Ni, Fe) E = -2693.79020646
```

Dimethylbenzylamine

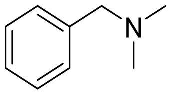

C	2.056925000	-0.054192000	1.459545000
N	2.033592000	-0.279351000	0.019237000
C	3.366634000	-0.160938000	-0.554301000
H	1.054080000	-0.190845000	1.873554000
H	2.406826000	0.962302000	1.730045000
H	3.334558000	-0.399049000	-1.623308000

H	3.805544000	0.850787000	-0.441675000
C	1.075416000	0.593047000	-0.658995000
H	1.267076000	1.664175000	-0.443842000
H	1.233505000	0.465605000	-1.738963000
C	-0.371363000	0.267077000	-0.333795000
C	-0.816964000	-1.062324000	-0.330562000
C	-1.294798000	1.286163000	-0.076273000
C	-2.155738000	-1.361927000	-0.080133000
C	-2.638084000	0.989932000	0.168985000
C	-3.072420000	-0.336033000	0.167683000
H	-0.097462000	-1.855383000	-0.512974000
H	-0.959500000	2.321318000	-0.067515000
H	-2.485945000	-2.397774000	-0.079430000
H	-3.341376000	1.794719000	0.367424000
H	-4.115891000	-0.570098000	0.361898000
H	2.725783000	-0.780784000	1.933634000
H	4.040014000	-0.874340000	-0.066430000

```
Zero-point correction=                           0.202872 (Hartree/Particle)
 Thermal correction to Energy=                    0.212522
 Thermal correction to Enthalpy=                  0.213466
 Thermal correction to Gibbs Free Energy=         0.167613
 Sum of electronic and zero-point Energies=           -405.314953
 Sum of electronic and thermal Energies=              -405.305303
 Sum of electronic and thermal Enthalpies=            -405.304359
 Sum of electronic and thermal Free Energies=         -405.350212

CPCM (Toluene) M06L/6-311++G(d,p) E = -405.567226047
```

TS-oa-2H-chromen-2-one (O-Ar)

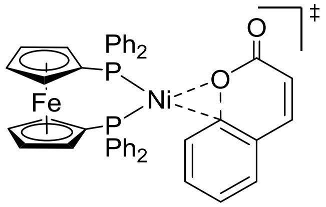

Fe	0.738730000	2.574179000	-1.585622000
P	-1.577481000	0.703995000	0.147114000
C	2.845573000	-1.257666000	-1.286994000
C	3.194237000	-0.276620000	1.421658000
C	2.320730000	-1.287336000	-2.591449000
H	1.461359000	-0.672558000	-2.841218000
C	-1.163659000	1.849432000	-1.217468000
C	-0.535214000	1.428735000	2.655841000
H	0.123787000	0.577147000	2.509612000
C	-1.292721000	3.274494000	4.024457000
H	-1.219000000	3.866303000	4.932937000
C	2.219847000	1.477193000	-0.614734000
C	5.179805000	0.517780000	2.575436000
H	6.028820000	1.195024000	2.623258000
C	3.946906000	-2.073060000	-0.990831000
H	4.365244000	-2.077871000	0.009769000
C	-3.403251000	0.485393000	-0.048565000
C	4.973780000	-0.423584000	3.587917000
H	5.661645000	-0.478065000	4.427940000
C	3.994356000	-2.897248000	-3.269565000
H	4.437382000	-3.531018000	-4.032917000
C	-1.486429000	1.744098000	1.673697000
C	-2.357082000	2.824252000	1.897362000
H	-3.129031000	3.057235000	1.169686000
C	-0.699998000	1.389940000	-2.500051000
H	-0.568920000	0.351755000	-2.776140000
C	-0.436153000	2.191281000	3.822732000
H	0.304807000	1.930748000	4.573470000

C	2.991696000	-1.220318000	2.441683000
H	2.155571000	-1.915633000	2.407944000
C	3.884120000	-1.291931000	3.515511000
H	3.715412000	-2.029931000	4.294918000
C	4.513245000	-2.887290000	-1.975100000
H	5.364436000	-3.514640000	-1.724213000
C	-1.181373000	3.285276000	-1.269266000
H	-1.437013000	3.942715000	-0.450370000
C	2.648697000	3.358640000	-1.905657000
H	2.913917000	3.989903000	-2.743986000
C	4.298742000	0.589258000	1.496610000
H	4.471067000	1.320085000	0.712309000
C	-0.731543000	3.694623000	-2.558062000
H	-0.589626000	4.717431000	-2.882747000
C	-2.256682000	3.586583000	3.061690000
H	-2.936922000	4.419069000	3.221600000
C	-6.166828000	0.031990000	-0.303783000
H	-7.235700000	-0.138419000	-0.401617000
C	-5.417568000	0.442430000	-1.406924000
H	-5.900309000	0.593686000	-2.368894000
C	-4.045192000	0.668911000	-1.281363000
H	-3.477301000	0.994800000	-2.147106000
C	2.895541000	-2.090818000	-3.576190000
H	2.479364000	-2.092619000	-4.580419000
C	-5.535069000	-0.158356000	0.927449000
H	-6.110572000	-0.477400000	1.792718000
C	1.864328000	2.647968000	0.143097000
H	1.438961000	2.651093000	1.137072000
C	-4.163457000	0.059815000	1.053535000
H	-3.684399000	-0.095689000	2.015821000
C	2.707875000	1.934624000	-1.884050000
H	3.037493000	1.302880000	-2.696278000
C	-0.436506000	2.524610000	-3.319181000
H	-0.040062000	2.503166000	-4.325987000
C	2.137832000	3.799429000	-0.649356000
H	1.942288000	4.824853000	-0.363553000
Ni	-0.258460000	-1.091221000	0.250048000
P	2.001784000	-0.237732000	0.010824000
O	0.023711000	-2.946642000	0.756952000
C	-3.595863000	-3.432548000	-0.693192000
C	-3.343691000	-3.544424000	-2.053226000
C	-2.601957000	-2.984127000	0.200206000
H	-4.115711000	-3.902991000	-2.727494000
C	-2.056873000	-3.248606000	-2.541533000
C	-1.361925000	-2.536681000	-0.333416000
H	-1.835995000	-3.384960000	-3.598020000
C	-1.054854000	-2.795918000	-1.691660000
H	-0.053293000	-2.609503000	-2.066025000
H	-4.561598000	-3.726839000	-0.287422000
C	-2.757101000	-3.051556000	1.623485000
H	-3.759487000	-3.204284000	2.021182000
C	-1.702027000	-3.017415000	2.481424000
H	-1.855627000	-3.122883000	3.550690000
C	-0.291052000	-3.079007000	2.079851000
O	0.603156000	-3.321421000	2.885185000

```
Zero-point correction=                           0.644554 (Hartree/Particle)
 Thermal correction to Energy=                    0.687181
 Thermal correction to Enthalpy=                  0.688125
 Thermal correction to Gibbs Free Energy=         0.566363
 Sum of electronic and zero-point Energies=          -2784.263641
 Sum of electronic and thermal Energies=             -2784.221014
 Sum of electronic and thermal Enthalpies=           -2784.220069
 Sum of electronic and thermal Free Energies=        -2784.341832
```

CPCM (Toluene) M06L/6-311++G(d,p) with LANL2DZ (for Ni, Fe) E = -2785.33393683

TS-oa-2H-chromen-2-one (O-CO)

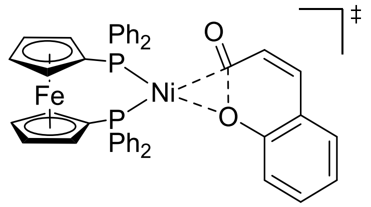

Fe	1.427994000	2.776618000	-0.835482000
P	-1.397797000	0.968731000	-0.030058000
C	3.026628000	-1.314068000	-1.274390000
C	2.747609000	-1.073548000	1.635646000
C	2.693820000	-1.073159000	-2.619133000
H	1.852099000	-0.428989000	-2.859471000
C	-0.583421000	2.322959000	-0.950185000
C	-0.900398000	1.026711000	2.728484000
H	-0.274814000	0.163559000	2.516308000
C	-1.836908000	2.596572000	4.313127000
H	-1.930777000	2.965403000	5.331146000
C	2.531111000	1.215012000	-0.022125000
C	4.526273000	-0.955678000	3.290475000
H	5.481176000	-0.548618000	3.613082000
C	4.096141000	-2.173824000	-0.991863000
H	4.358300000	-2.392964000	0.037384000
C	-3.137816000	1.068902000	-0.664544000
C	3.842970000	-1.869243000	4.097480000
H	4.265758000	-2.175588000	5.050889000
C	4.499397000	-2.502354000	-3.358713000
H	5.068106000	-2.962854000	-4.162072000
C	-1.598071000	1.644762000	1.680035000
C	-2.436990000	2.733032000	1.971877000
H	-3.013614000	3.199028000	1.177723000
C	0.049238000	2.122056000	-2.226631000
H	0.115006000	1.173712000	-2.743298000
C	-1.016028000	1.502117000	4.037510000
H	-0.470827000	1.010896000	4.839018000
C	2.069622000	-1.992701000	2.451430000
H	1.128253000	-2.416390000	2.120258000
C	2.617821000	-2.386317000	3.674887000
H	2.083418000	-3.101328000	4.294669000
C	4.824649000	-2.763986000	-2.027720000
H	5.647004000	-3.433348000	-1.789332000
C	-0.411353000	3.708705000	-0.612625000
H	-0.731573000	4.176026000	0.307984000
C	3.454034000	3.245805000	-0.667130000
H	3.952389000	4.007188000	-1.253223000
C	3.982355000	-0.559510000	2.068531000
H	4.518116000	0.156730000	1.452103000
C	0.316864000	4.341269000	-1.662185000
H	0.643413000	5.373284000	-1.673196000
C	-2.550501000	3.208703000	3.278537000
H	-3.202457000	4.052040000	3.491244000
C	-5.799413000	1.125621000	-1.576263000
H	-6.827643000	1.147881000	-1.927779000
C	-4.823554000	1.894009000	-2.211261000
H	-5.087860000	2.522114000	-3.058347000
C	-3.501818000	1.868550000	-1.758326000
H	-2.756767000	2.479862000	-2.257052000
C	3.430200000	-1.652420000	-3.652890000
H	3.162535000	-1.448108000	-4.686525000
C	-5.445744000	0.323765000	-0.488383000
H	-6.193085000	-0.288918000	0.007415000
C	2.224932000	2.199367000	0.980986000
H	1.638271000	2.030674000	1.873758000
C	-4.126358000	0.290542000	-0.038889000
H	-3.870670000	-0.349145000	0.800968000

C	3.297474000	1.877751000	-1.038163000
H	3.664933000	1.420258000	-1.945882000
C	0.596709000	3.362818000	-2.662553000
H	1.167028000	3.523238000	-3.568312000
C	2.798306000	3.441895000	0.584073000
H	2.707560000	4.379425000	1.117181000
P	1.974566000	-0.541108000	0.039560000
Ni	-0.179433000	-1.008702000	-0.117642000
C	-2.480749000	-2.932581000	0.428511000
C	-3.431568000	-3.212450000	1.425802000
H	-3.110944000	-3.211735000	2.463362000
O	-1.221370000	-2.631586000	0.770188000
C	0.093211000	-2.941629000	-0.383862000
O	0.932330000	-3.707630000	0.031233000
C	-2.869024000	-2.937703000	-0.936608000
C	-4.748524000	-3.489214000	1.071181000
C	-4.212067000	-3.203614000	-1.264152000
H	-4.506987000	-3.199038000	-2.311400000
C	-5.146821000	-3.483184000	-0.275797000
H	-6.176809000	-3.701685000	-0.542680000
H	-5.474977000	-3.713311000	1.848543000
C	-0.513646000	-2.730472000	-1.692930000
C	-1.860068000	-2.682817000	-1.933021000
H	-2.194588000	-2.532127000	-2.958419000
H	0.196965000	-2.668920000	-2.512787000

```
Zero-point correction=                           0.644084 (Hartree/Particle)
 Thermal correction to Energy=                    0.686925
 Thermal correction to Enthalpy=                  0.687870
 Thermal correction to Gibbs Free Energy=         0.565385
 Sum of electronic and zero-point Energies=          -2784.285526
 Sum of electronic and thermal Energies=             -2784.242684
 Sum of electronic and thermal Enthalpies=           -2784.241740
 Sum of electronic and thermal Free Energies=        -2784.364224
```

CPCM (Toluene) M06L/6-311++G(d,p) with LANL2DZ (for Ni, Fe) E = -2785.35523303

2H-chromen-2-one

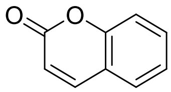

C	-2.158669000	0.474528000	0.000000000
C	-2.784514000	-0.764453000	0.000000000
C	-0.754196000	0.567703000	0.000000000
H	-3.868359000	-0.827817000	0.000000000
C	-2.011783000	-1.936455000	0.000000000
C	0.000000000	-0.622042000	0.000000000
H	-2.500811000	-2.906407000	0.000000000
C	-0.622094000	-1.872691000	0.000000000
H	-0.006602000	-2.766110000	0.000000000
H	-2.745481000	1.389714000	0.000000000
C	-0.026586000	1.812562000	0.000000000
H	-0.587200000	2.744752000	0.000000000
C	1.325605000	1.822926000	0.000000000
H	1.904585000	2.739129000	0.000000000
C	2.103054000	0.586462000	0.000000000
O	3.307954000	0.501634000	0.000000000
O	1.364416000	-0.599697000	0.000000000

```
Zero-point correction=                           0.127810 (Hartree/Particle)
 Thermal correction to Energy=                    0.135482
 Thermal correction to Enthalpy=                  0.136426
 Thermal correction to Gibbs Free Energy=         0.095153
 Sum of electronic and zero-point Energies=           -496.886501
 Sum of electronic and thermal Energies=              -496.878830
 Sum of electronic and thermal Enthalpies=            -496.877886
 Sum of electronic and thermal Free Energies=         -496.919158

CPCM (Toluene) M06L/6-311++G(d,p) E = -497.095188454
```

TS-oa-Diphenylether

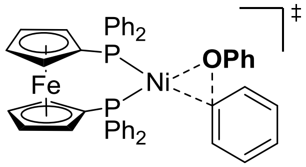

C	0.244760000	2.643667000	0.722718000
C	-0.738002000	3.652338000	0.663335000
C	1.108345000	2.569560000	1.832321000
C	-0.939255000	4.469517000	1.769995000
C	0.869172000	3.383055000	2.948798000
H	1.963261000	1.898079000	1.813755000
C	-0.153044000	4.331104000	2.927230000
H	-1.348952000	3.761173000	-0.225780000
H	-1.722430000	5.223832000	1.736049000
Fe	-0.964053000	-2.608657000	-1.744034000
P	1.424938000	-1.175844000	0.274877000
C	-2.794230000	1.467709000	-1.337571000
C	-3.037109000	0.357950000	1.361698000
C	-2.004227000	2.117716000	-2.299823000
H	-0.922642000	2.015252000	-2.264035000
C	0.984115000	-2.230438000	-1.148918000
C	0.039374000	-1.880143000	2.619342000
H	-0.532662000	-0.978768000	2.420489000
C	0.487193000	-3.810727000	4.007393000
H	0.260675000	-4.417403000	4.880259000
C	-2.337755000	-1.292805000	-0.866624000
C	-5.017985000	-0.337997000	2.594112000
H	-5.937112000	-0.916726000	2.639262000
C	-4.183512000	1.664608000	-1.356267000
H	-4.811639000	1.187914000	-0.610411000
C	3.279087000	-1.217139000	0.250972000
C	-4.624499000	0.439876000	3.685403000
H	-5.236585000	0.469518000	4.583130000
C	-3.979353000	3.110465000	-3.287776000
H	-4.438676000	3.747060000	-4.039557000
C	1.077890000	-2.242353000	1.749482000
C	1.833190000	-3.391262000	2.039320000
H	2.665166000	-3.667208000	1.397588000
C	0.747987000	-1.692904000	-2.463446000
H	0.798428000	-0.643993000	-2.725043000
C	-0.257222000	-2.661034000	3.739765000
H	-1.064561000	-2.363250000	4.403458000
C	-2.647970000	1.130444000	2.468494000
H	-1.724261000	1.698234000	2.431073000
C	-3.438632000	1.172926000	3.619125000
H	-3.120908000	1.778342000	4.463854000
C	-4.770840000	2.481817000	-2.323412000
H	-5.847965000	2.628613000	-2.321728000
C	0.756830000	-3.646418000	-1.225125000
H	0.805028000	-4.335694000	-0.393596000
C	-2.914501000	-2.958689000	-2.379355000
H	-3.165448000	-3.466213000	-3.301848000

C	-4.231033000	-0.379447000	1.441849000
H	-4.542756000	-0.999477000	0.606938000
C	0.389776000	-3.969879000	-2.563859000
H	0.111448000	-4.952040000	-2.924210000
C	1.535858000	-4.172109000	3.156779000
H	2.128074000	-5.058814000	3.368030000
C	6.091563000	-1.124160000	0.273526000
H	7.177559000	-1.085955000	0.280758000
C	5.361082000	-0.529123000	1.306069000
H	5.876685000	-0.025034000	2.119095000
C	3.968476000	-0.570367000	1.290885000
H	3.411817000	-0.098058000	2.097089000
C	-2.595633000	2.928041000	-3.272753000
H	-1.970177000	3.426120000	-4.008967000
C	5.418312000	-1.764067000	-0.767019000
H	5.978074000	-2.231725000	-1.573113000
C	-2.323052000	-2.559120000	-0.184428000
H	-2.063744000	-2.714924000	0.853815000
C	4.020954000	-1.813981000	-0.778179000
H	3.510773000	-2.319831000	-1.591971000
C	-2.698112000	-1.557132000	-2.229946000
H	-2.770118000	-0.814725000	-3.012656000
C	0.389693000	-2.764901000	-3.329380000
H	0.115035000	-2.673547000	-4.372236000
C	-2.690909000	-3.576607000	-1.113053000
H	-2.741365000	-4.637109000	-0.902258000
Ni	0.244325000	0.835494000	0.109549000
P	-1.931067000	0.348478000	-0.128530000
O	1.089152000	2.383369000	-0.884722000
H	1.514274000	3.293184000	3.820214000
H	-0.317365000	4.981531000	3.781649000
C	2.377305000	2.812307000	-0.969653000
C	2.705116000	4.147053000	-0.670107000
C	3.380648000	1.954357000	-1.446375000
C	4.009554000	4.604845000	-0.850739000
C	4.679903000	2.425115000	-1.634815000
C	5.004545000	3.750312000	-1.334450000
H	1.926771000	4.810172000	-0.306280000
H	3.126305000	0.924636000	-1.673376000
H	4.248586000	5.639703000	-0.616620000
H	5.442256000	1.746688000	-2.009878000
H	6.019024000	4.113356000	-1.476083000

```
Zero-point correction=                           0.701776 (Hartree/Particle)
 Thermal correction to Energy=                    0.746987
 Thermal correction to Enthalpy=                  0.747931
 Thermal correction to Gibbs Free Energy=         0.619625
 Sum of electronic and zero-point Energies=          -2825.688069
 Sum of electronic and thermal Energies=             -2825.642858
 Sum of electronic and thermal Enthalpies=           -2825.641914
 Sum of electronic and thermal Free Energies=        -2825.770220
```

CPCM (Toluene) M06L/6-311++G(d,p) with LANL2DZ (for Ni, Fe) E = -2826.80959352

Diphenylether

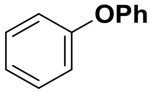

C	-1.201618000	-0.499288000	0.036527000
C	-2.268208000	-1.072412000	-0.660093000
C	-1.388578000	0.660413000	0.795596000
H	-2.093145000	-1.975890000	-1.235653000
H	-0.556335000	1.090489000	1.342991000
C	-3.528920000	-0.478906000	-0.598139000
C	-2.653506000	1.248233000	0.841727000
H	-4.357022000	-0.927261000	-1.140543000
H	-2.798805000	2.149043000	1.432174000
C	-3.727266000	0.685078000	0.148260000
H	-4.709081000	1.147606000	0.191089000
O	-0.000340000	-1.180584000	-0.002653000
C	1.201390000	-0.499734000	-0.038589000
C	2.265480000	-1.071431000	0.662862000
C	1.391081000	0.657931000	-0.800129000
C	3.526619000	-0.478406000	0.603398000
C	2.656235000	1.245353000	-0.843687000
C	3.727592000	0.683625000	-0.145246000
H	2.088331000	-1.973423000	1.240099000
H	0.560575000	1.086568000	-1.351305000
H	4.352897000	-0.925565000	1.149557000
H	2.803818000	2.144579000	-1.435972000
H	4.709667000	1.145783000	-0.186129000

```
Zero-point correction=                           0.185732 (Hartree/Particle)
 Thermal correction to Energy=                    0.195712
 Thermal correction to Enthalpy=                  0.196656
 Thermal correction to Gibbs Free Energy=         0.148388
 Sum of electronic and zero-point Energies=           -538.316561
 Sum of electronic and thermal Energies=              -538.306581
 Sum of electronic and thermal Enthalpies=            -538.305637
 Sum of electronic and thermal Free Energies=         -538.353905
```

CPCM (Toluene) M06L/6-311++G(d,p) E = -538.573893133

4. References

1 E. J. Cho and S. L. Buchwald, *Org. Lett.*, 2011, **13**, 6552–6555.
2 G. Yin, I. Kalvet and F. Schoenebeck, *Angew. Chem. Int. Ed.*, 2015, **54**, 6809–6813.
3 W. Tyrra, D. Naumann, B. Hoge and Y. L. Yagupolskii, *J. Fluor. Chem.*, 2003, **119**, 101–107.
4 F. Proutiere and F. Schoenebeck, *Angew. Chem. Int. Ed.*, 2011, **50**, 8192–8195.
5 Y. Zou, L. Qin, X. Ren, Y. Lu, Y. Li and J. S. Zhou, *Chem. - Eur. J.*, 2013, **19**, 3504–3511.
6 T. Maegawa, Y. Kitamura, S. Sako, T. Udzu, A. Sakurai, A. Tanaka, Y. Kobayashi, K. Endo, U. Bora, T. Kurita, A. Kozaki, Y. Monguchi and H. Sajiki, *Chem. - Eur. J.*, 2007, **13**, 5937–5943.
7 M. Chtchigrovsky, A. Primo, P. Gonzalez, K. Molvinger, M. Robitzer, F. Quignard and F. Taran, *Angew. Chem. Int. Ed.*, 2009, **48**, 5916–5920.
8 C. W. Y. Chung and P. H. Toy, *Tetrahedron*, 2005, **61**, 709–715.
9 J. P. Wolfe and S. L. Buchwald, *J. Org. Chem.*, 1997, **62**, 1264–1267.
10 J. W. W. Chang, E. Y. Chia, C. L. L. Chai and J. Seayad, *Org. Biomol. Chem.*, 2012, **10**, 2289.
11 T. Liu, X. Shao, Y. Wu and Q. Shen, *Angew. Chem. Int. Ed.*, 2012, **51**, 540–543.
12 J. M. Howell, W. Liu, A. J. Young and M. C. White, *J. Am. Chem. Soc.*, 2014, **136**, 5750–5754.
13 K. W. Anderson, M. Mendez-Perez, J. Priego and S. L. Buchwald, *J. Org. Chem.*, 2003, **68**, 9563–9573.
14 M. Uemura, H. Yorimitsu and K. Oshima, *Tetrahedron*, 2008, **64**, 1829–1833.
15 S. P. H. Mee, V. Lee and J. E. Baldwin, *Chem. - Eur. J.*, 2005, **11**, 3294–3308.
16 A. C. Spivey, L. J. Martin, C.-C. Tseng, G. J. Ellames and A. D. Kohler, *Org. Biomol. Chem.*, 2008, **6**, 4093.
17 M. A. Grundl, A. Kaster, E. D. Beaulieu and D. Trauner, *Org. Lett.*, 2006, **8**, 5429–5432.
18 I. V. Magedov, N. M. Evdokimov, M. Karki, A. S. Peretti, D. T. Lima, L. V. Frolova, M. R. Reisenauer, A. E. Romero, P. Tongwa, A. Fonari, J. Altig, S. Rogelj, M. Y. Antipin, C. B. Shuster and A. Kornienko, *Chem. Commun.*, 2012, **48**, 10416.
19 B. Scheiper, M. Bonnekessel, H. Krause and A. Fuerstner, *J. Org. Chem.*, 2004, **69**, 3943–3949.
20 A. Deagostino, M. Migliardi, E. G. Occhiato, C. Prandi, C. Zavattaro and P. Venturello, *Tetrahedron*, 2005, **61**, 3429–3436.
21 R. Pluta, P. Nikolaienko and M. Rueping, *Angew. Chem. Int. Ed.*, 2014, **53**, 1650–1653.
22 C.-P. Zhang and D. A. Vicic, *Chem. - Asian J.*, 2012, **7**, 1756–1758.
23 G. Danoun, B. Bayarmagnai, M. F. Gruenberg and L. J. Goossen, *Chem. Sci.*, 2014, **5**, 1312.
24 F. Baert, J. Colomb and T. Billard, *Angew. Chem. Int. Ed.*, 2012, **51**, 10382–10385.
25 C. Chen, Y. Xie, L. Chu, R.-W. Wang, X. Zhang and F.-L. Qing, *Angew. Chem. Int. Ed.*, 2012, **51**, 2492–2495.
26 Gaussian 09, Revision D.01, Frisch, M. J.; Trucks, G. W.; Schlegel, H. B.; Scuseria, G. E.; Robb, M. A.; Cheeseman, J. R.; Scalmani, G.; Barone, V.; Mennucci, B.; Petersson, G. A.; Nakatsuji, H.; Caricato, M.; Li, X.; Hratchian, H. P.; Izmaylov, A. F.; Bloino, J.; Zheng, G.; Sonnenberg, J. L.; Hada, M.; Ehara, M.; Toyota, K.; Fukuda, R.; Hasegawa, J.; Ishida, M.; Nakajima, T.; Honda, Y.; Kitao, O.; Nakai, H.; Vreven, T.; Montgomery, J. A., Jr.; Peralta, J. E.; Ogliaro, F.; Bearpark, M.; Heyd, J. J.; Brothers, E.; Kudin, K. N.; Staroverov, V. N.; Kobayashi, R.; Normand, J.; Raghavachari, K.; Rendell, A.; Burant, J. C.; Iyengar, S. S.; Tomasi, J.; Cossi, M.; Rega, N.; Millam, J. M.; Klene, M.; Knox, J. E.; Cross, J. B.; Bakken, V.; Adamo, C.; Jaramillo, J.; Gomperts, R.; Stratmann, R. E.; Yazyev, O.; Austin, A. J.; Cammi, R.; Pomelli, C.; Ochterski, J. W.; Martin, R. L.; Morokuma, K.; Zakrzewski, V. G.; Voth, G. A.; Salvador, P.; Dannenberg, J. J.; Dapprich, S.; Daniels, A. D.; Farkas, Ö.; Foresman, J. B.; Ortiz, J. V.; Cioslowski, J.; Fox, D. J. Gaussian, Inc., Wallingford CT, 2009, .

5. NMR Spectra

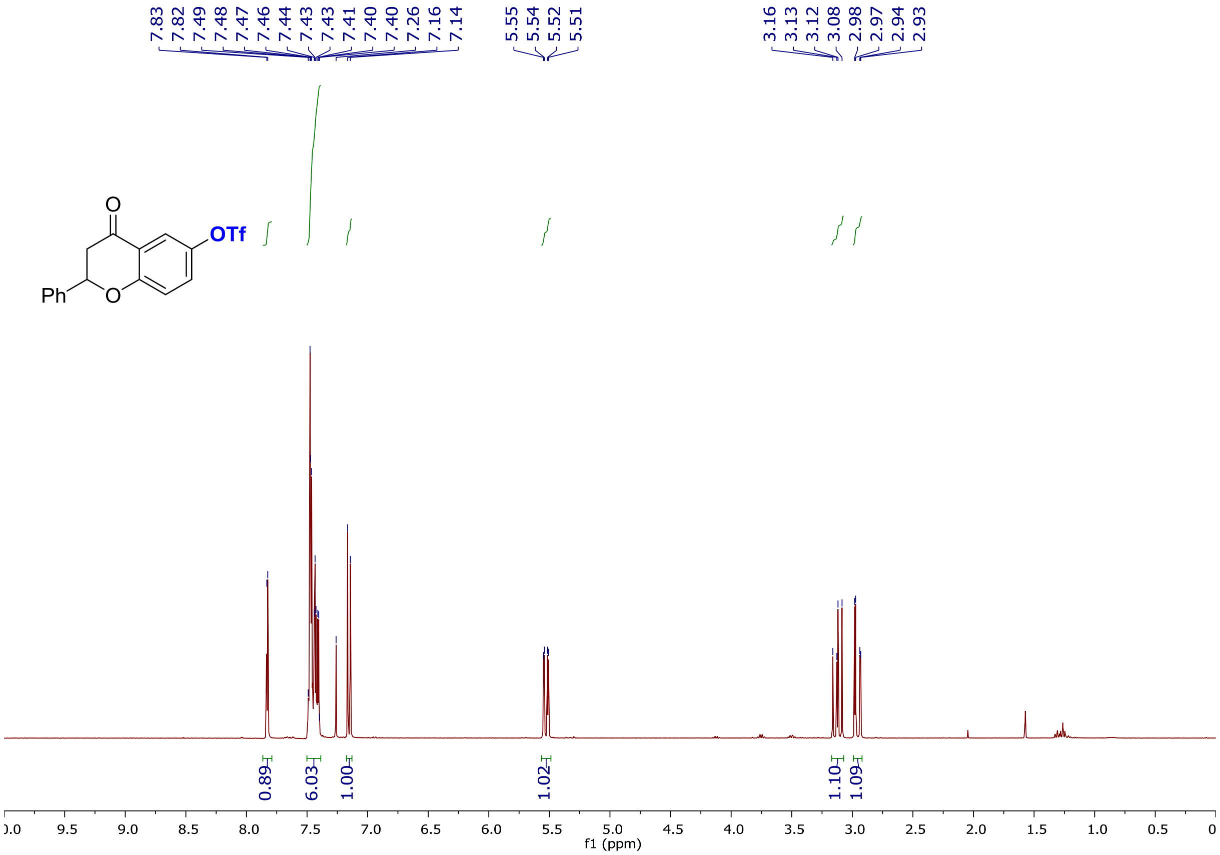

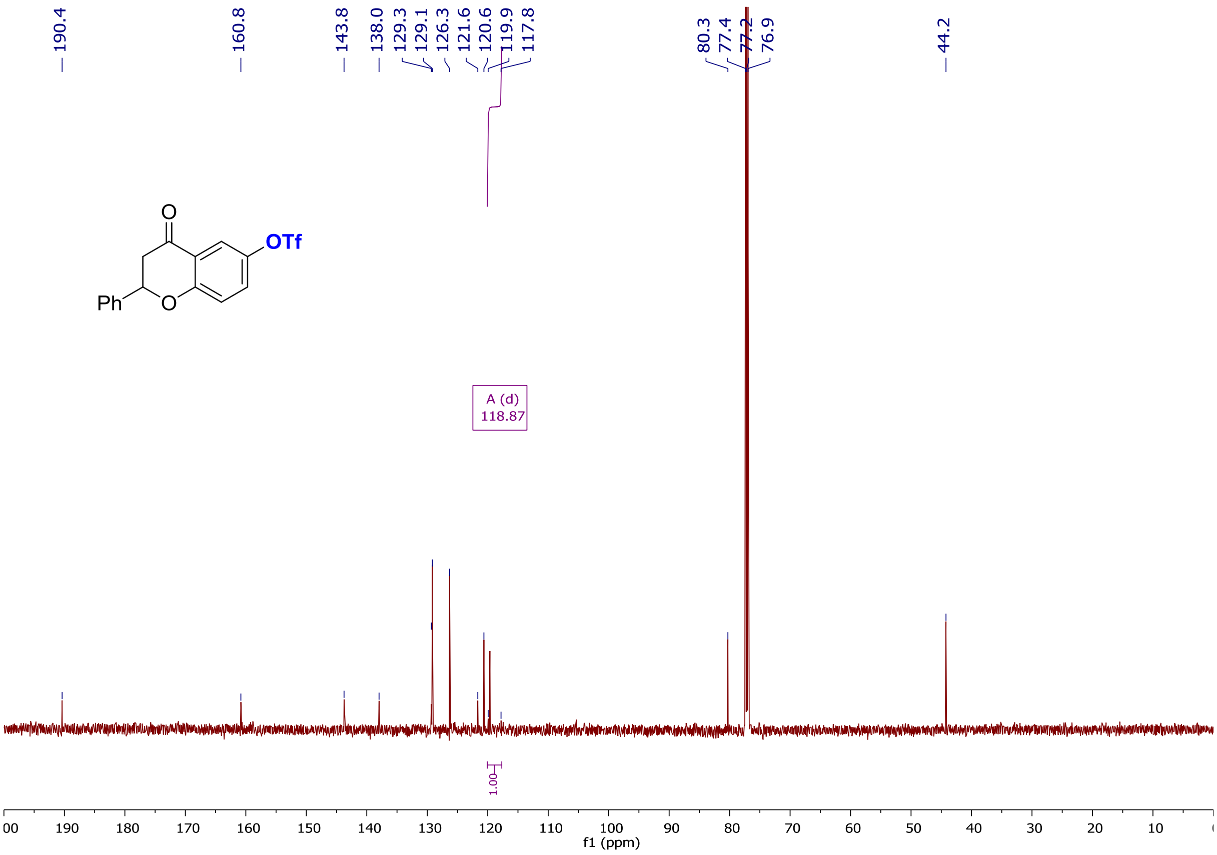
190.4
160.8
143.8
138.0
129.3
129.1
126.3
121.6
120.6
119.9
117.8
80.3
77.4
77.2
76.9
44.2
O
OTf
Ph
O
A (d)
118.87
1.00
00 190 180 170 160 150 140 130 120 110 100 90 80 70 60 50 40 30 20 10
f1 (ppm)

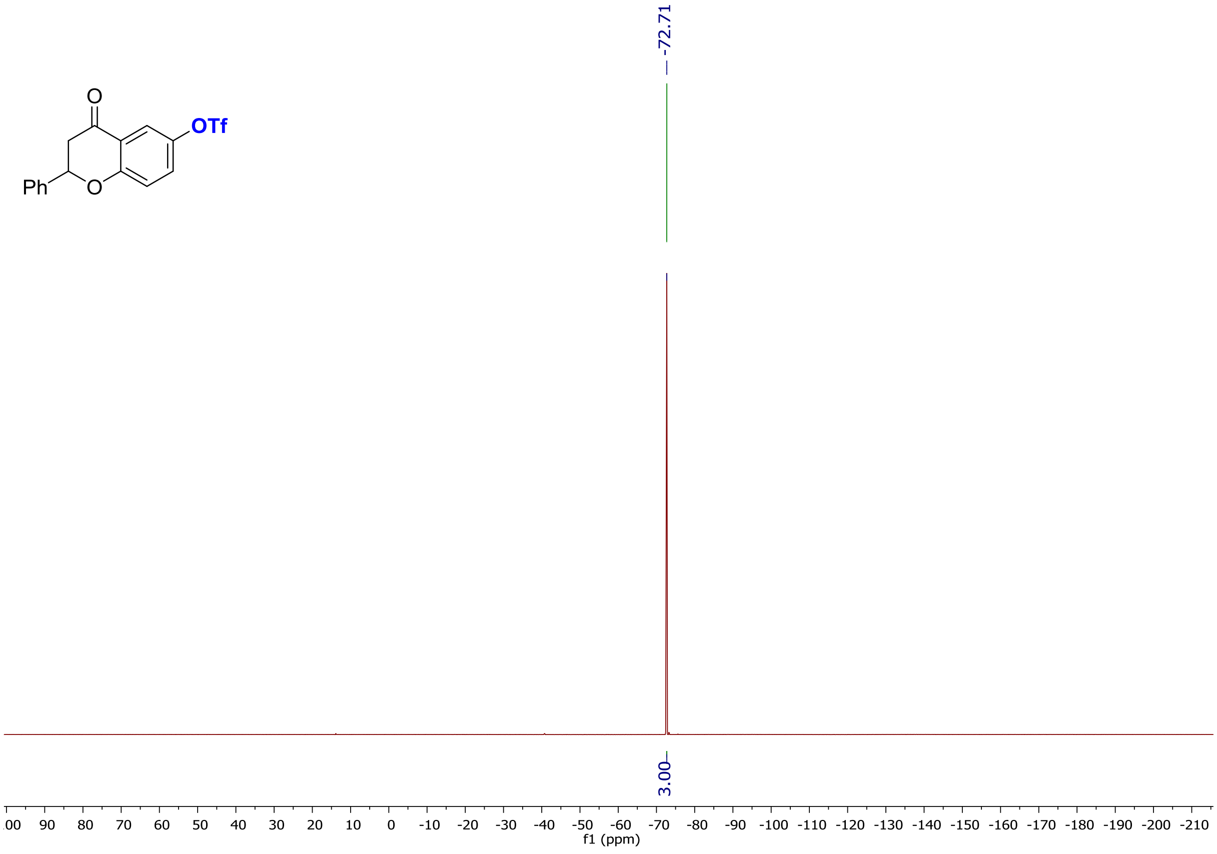
-72.71
O
OTf
Ph
O
3.00
00 90 80 70 60 50 40 30 20 10 0 -10 -20 -30 -40 -50 -60 -70 -80 -90 -100 -110 -120 -130 -140 -150 -160 -170 -180 -190 -200 -210
f1 (ppm)

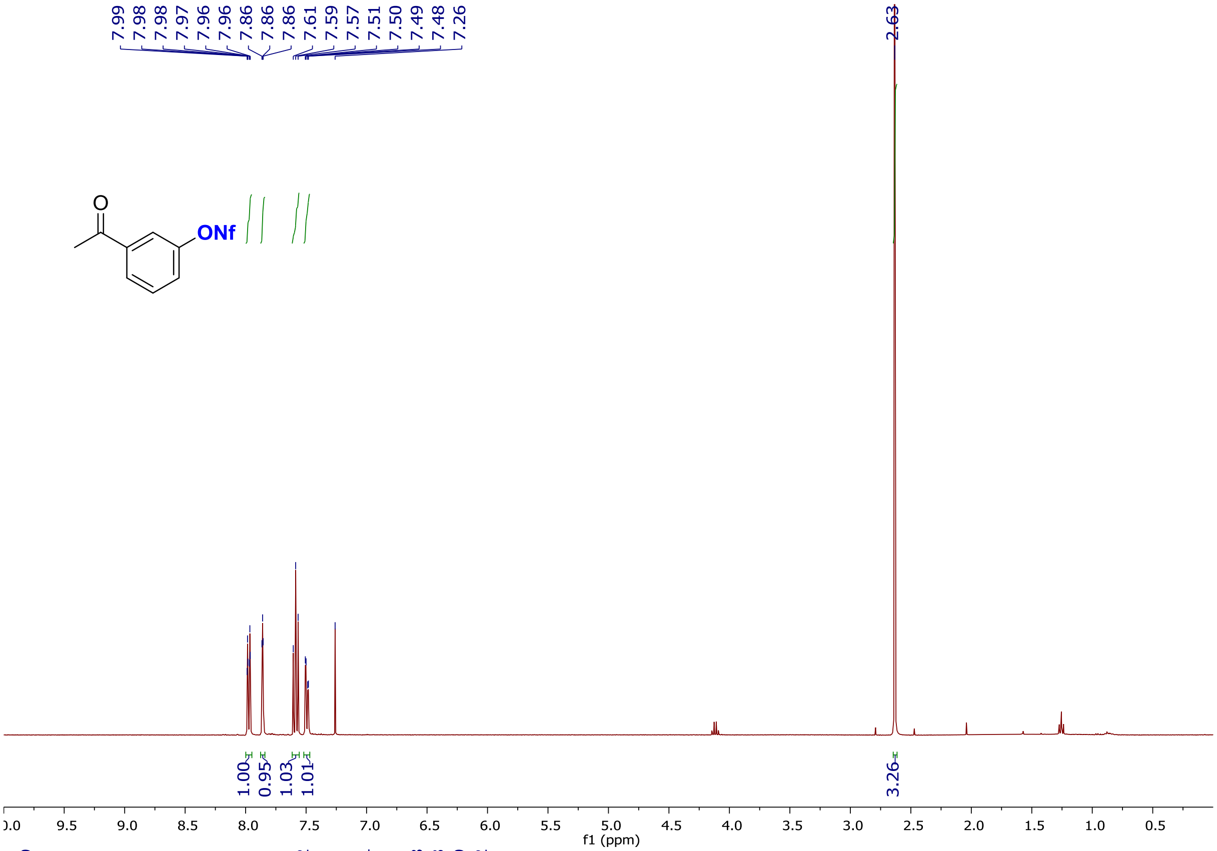
7.99
7.98
7.98
7.97
7.96
7.96
7.86
7.86
7.86
7.61
7.59
7.57
7.51
7.50
7.49
7.48
7.26
2.63
O
ONf
1.00
0.95
1.03
1.01
3.26
f1 (ppm)

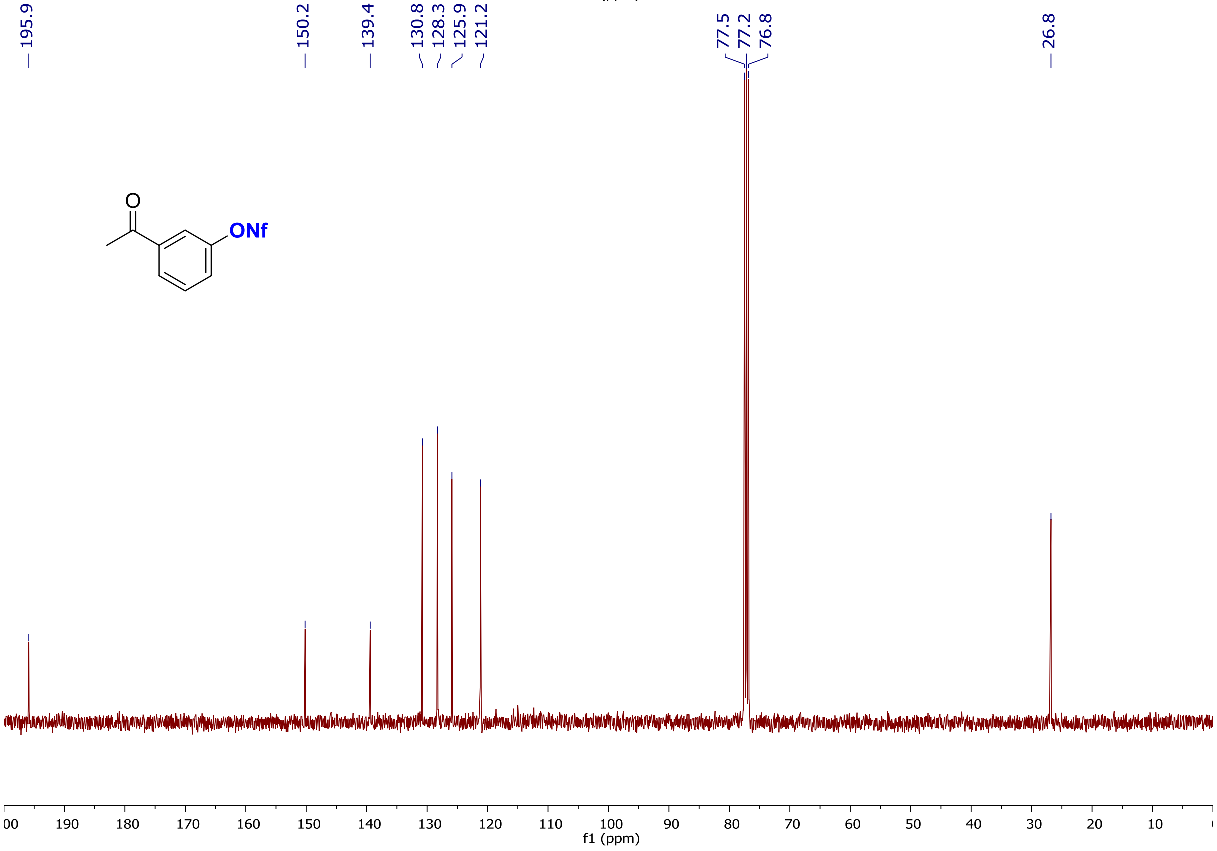
195.9
150.2
139.4
130.8
128.3
125.9
121.2
77.5
77.2
76.8
26.8
O
ONf
f1 (ppm)

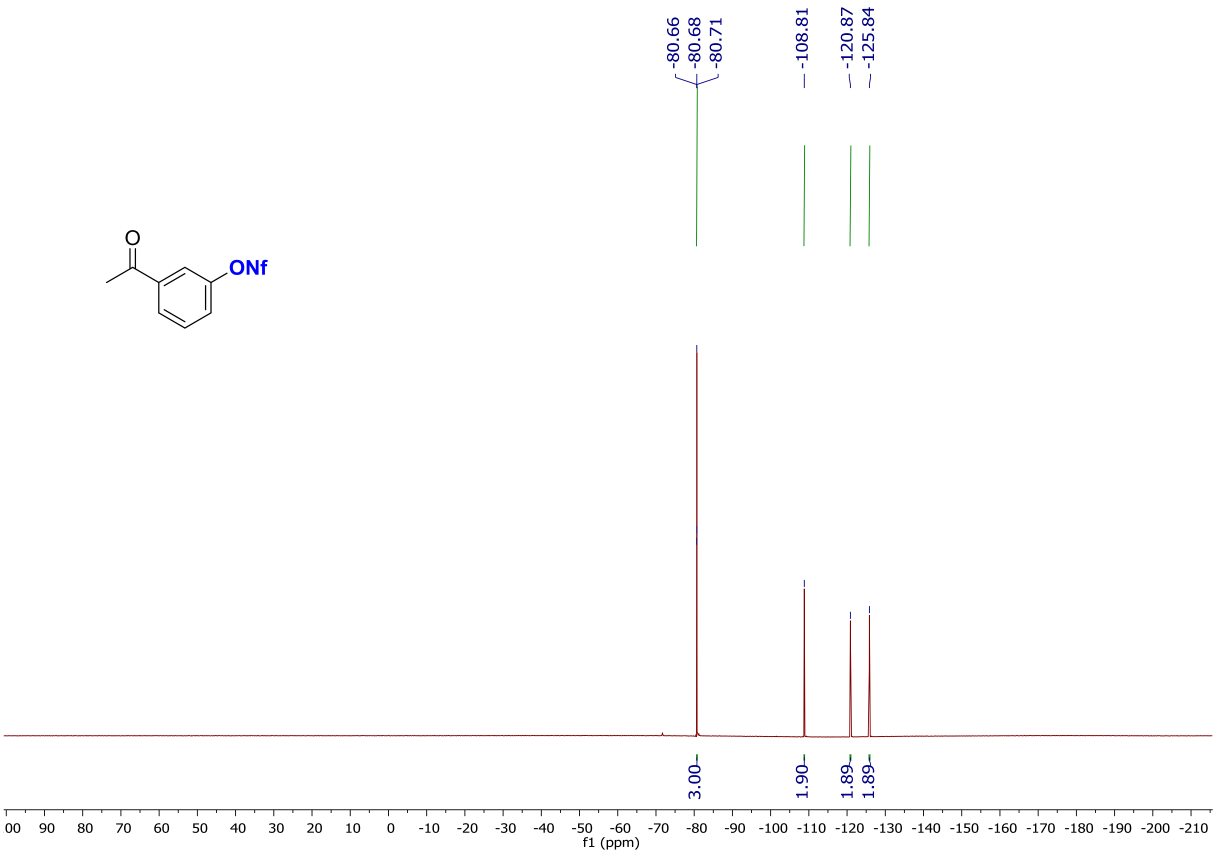
ONf
-80.66
-80.68
-80.71
-108.81
-120.87
-125.84
3.00
1.90
1.89
1.89
00 90 80 70 60 50 40 30 20 10 0 -10 -20 -30 -40 -50 -60 -70 -80 -90 -100 -110 -120 -130 -140 -150 -160 -170 -180 -190 -200 -210
f1 (ppm)

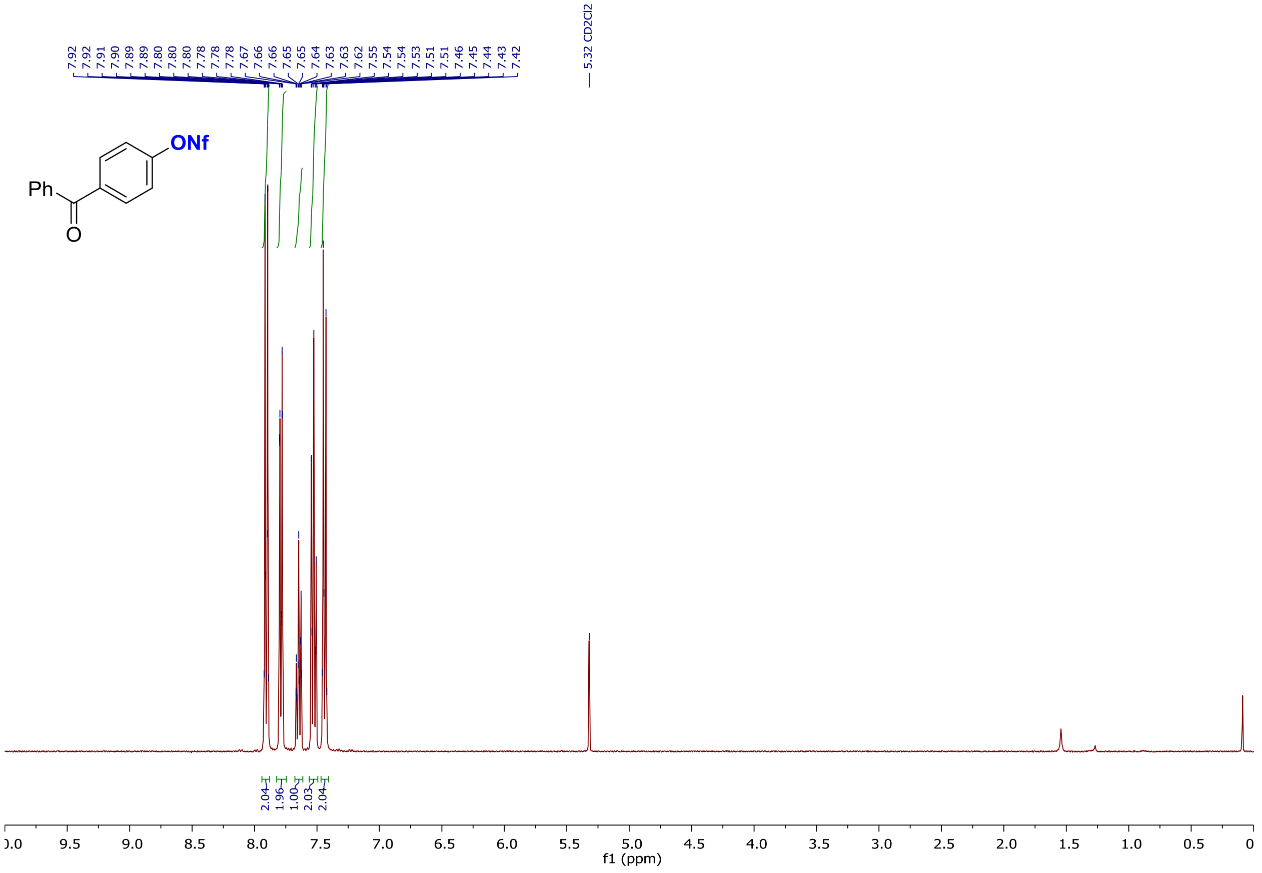
ONf
Ph
O
5.32 CD2Cl2
f1 (ppm)

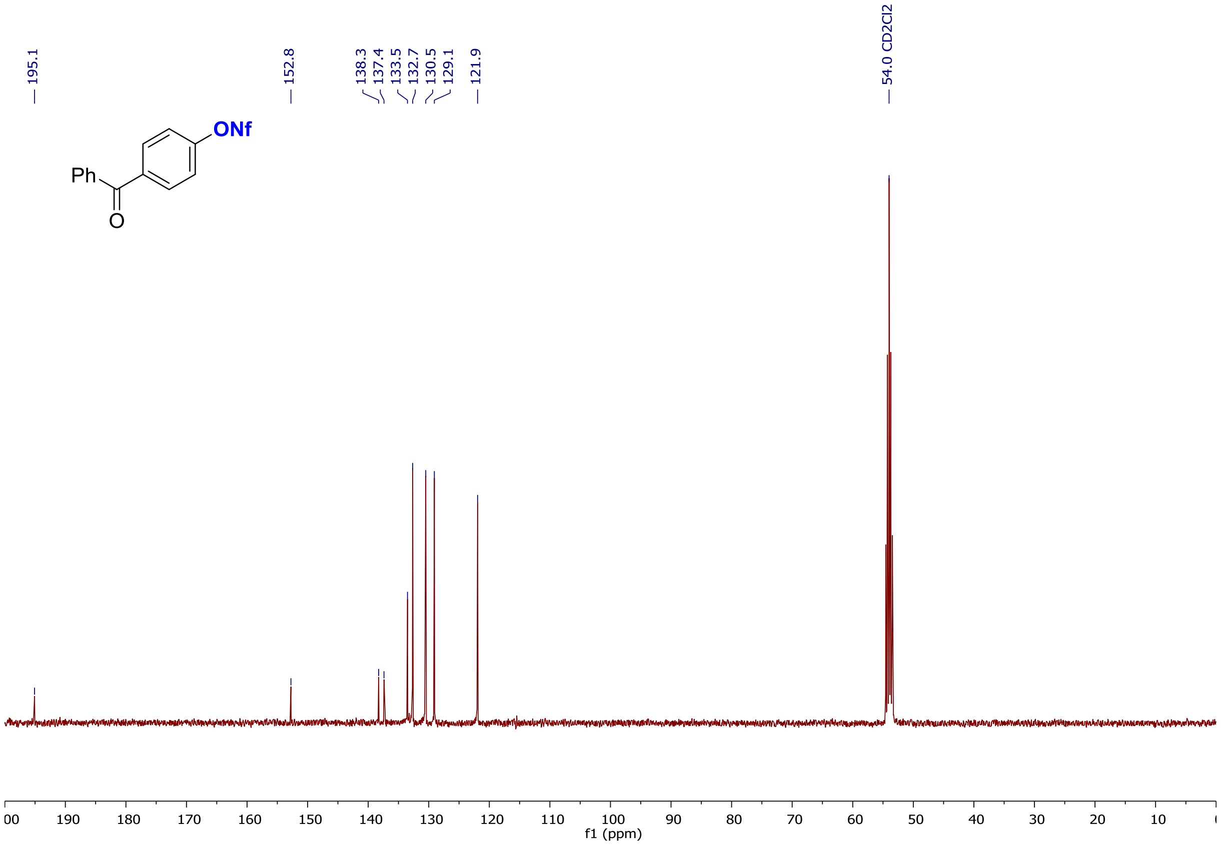
ONf
Ph
O
195.1
152.8
138.3
137.4
133.5
132.7
130.5
129.1
121.9
54.0 CD2Cl2
f1 (ppm)

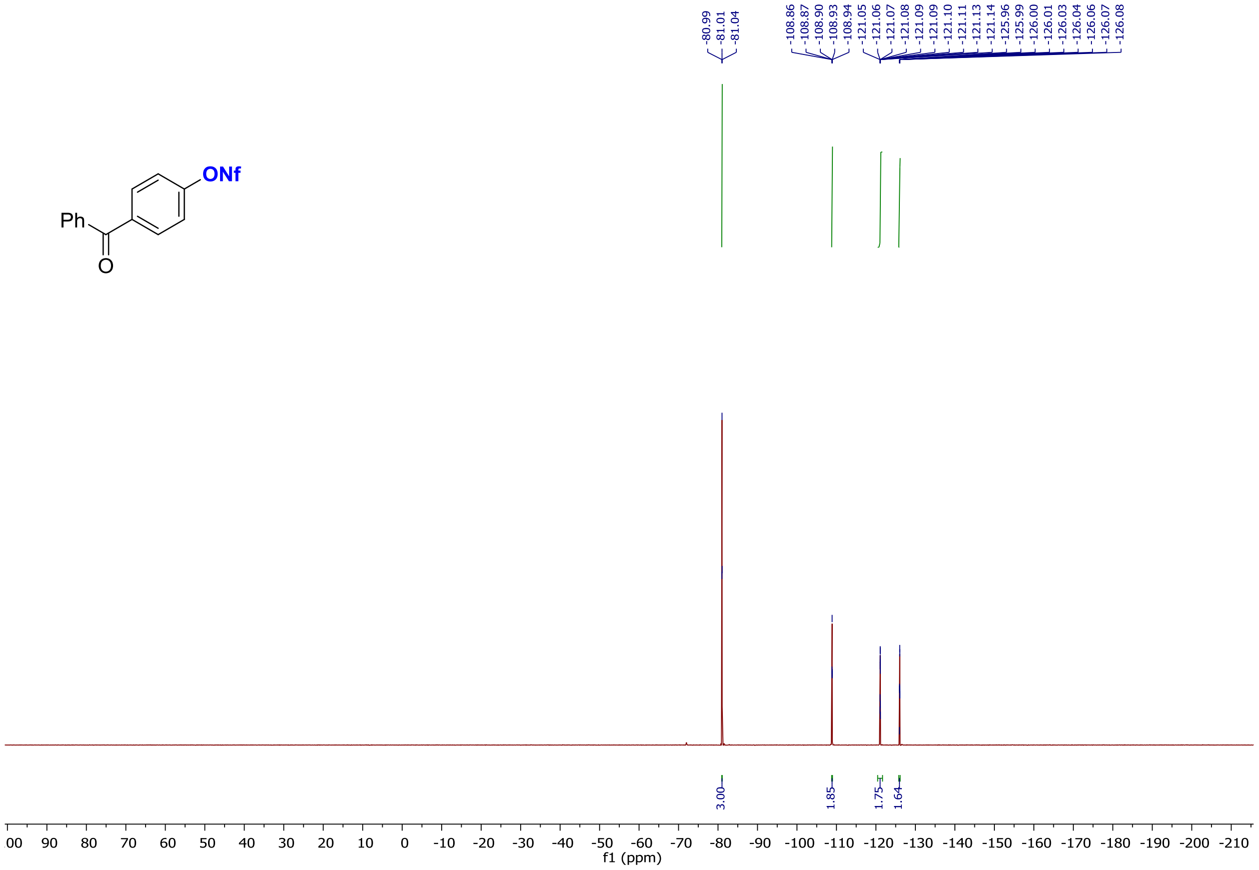
ONf
Ph
O
-80.99
-81.01
-81.04
-108.86
-108.87
-108.90
-108.93
-108.94
-121.05
-121.06
-121.07
-121.08
-121.09
-121.09
-121.10
-121.11
-121.13
-121.14
-125.96
-125.99
-126.00
-126.01
-126.03
-126.04
-126.06
-126.07
-126.08
3.00
1.85
1.75
1.64
00 90 80 70 60 50 40 30 20 10 0 -10 -20 -30 -40 -50 -60 -70 -80 -90 -100 -110 -120 -130 -140 -150 -160 -170 -180 -190 -200 -210
f1 (ppm)

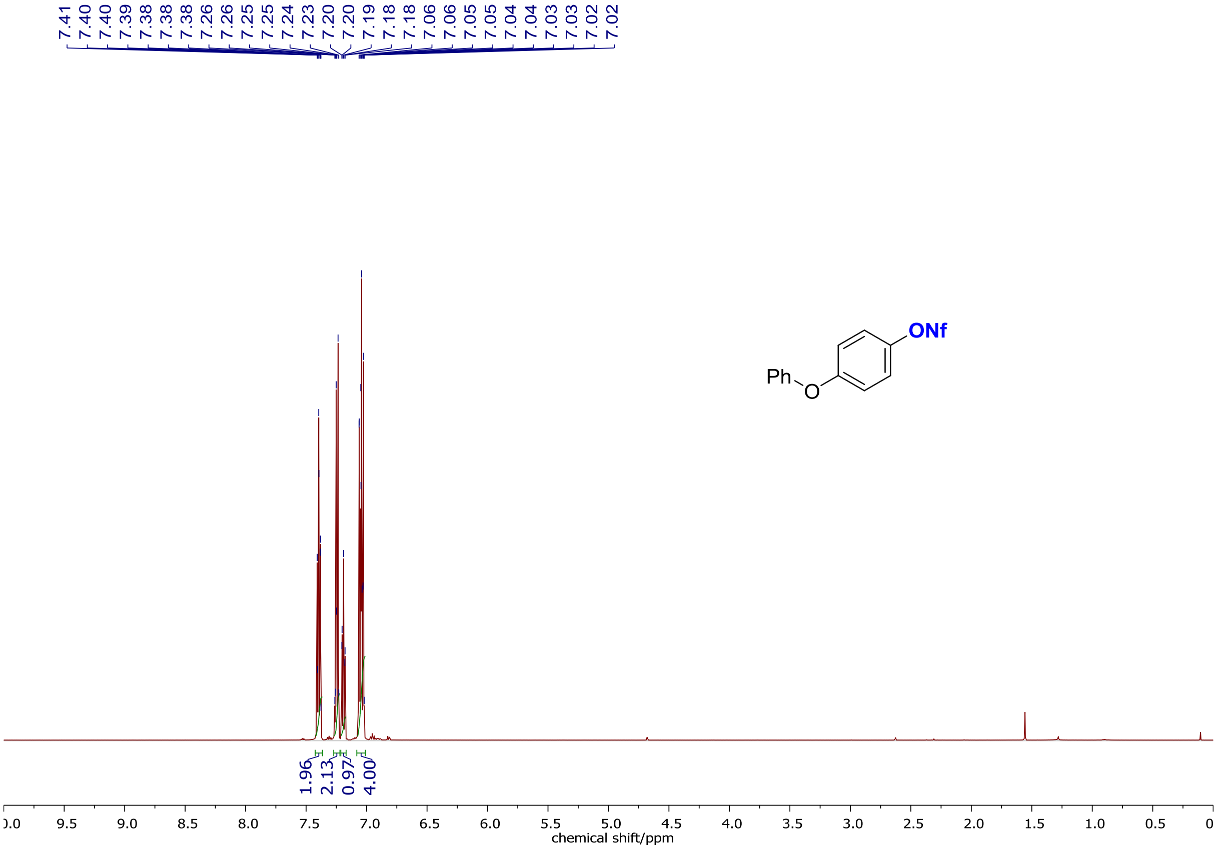
7.41
7.40
7.40
7.39
7.38
7.38
7.38
7.26
7.26
7.25
7.25
7.24
7.23
7.20
7.20
7.19
7.18
7.18
7.06
7.06
7.05
7.05
7.04
7.04
7.03
7.02
7.02
ONf
Ph
O
1.96
2.13
0.97
4.00
chemical shift/ppm

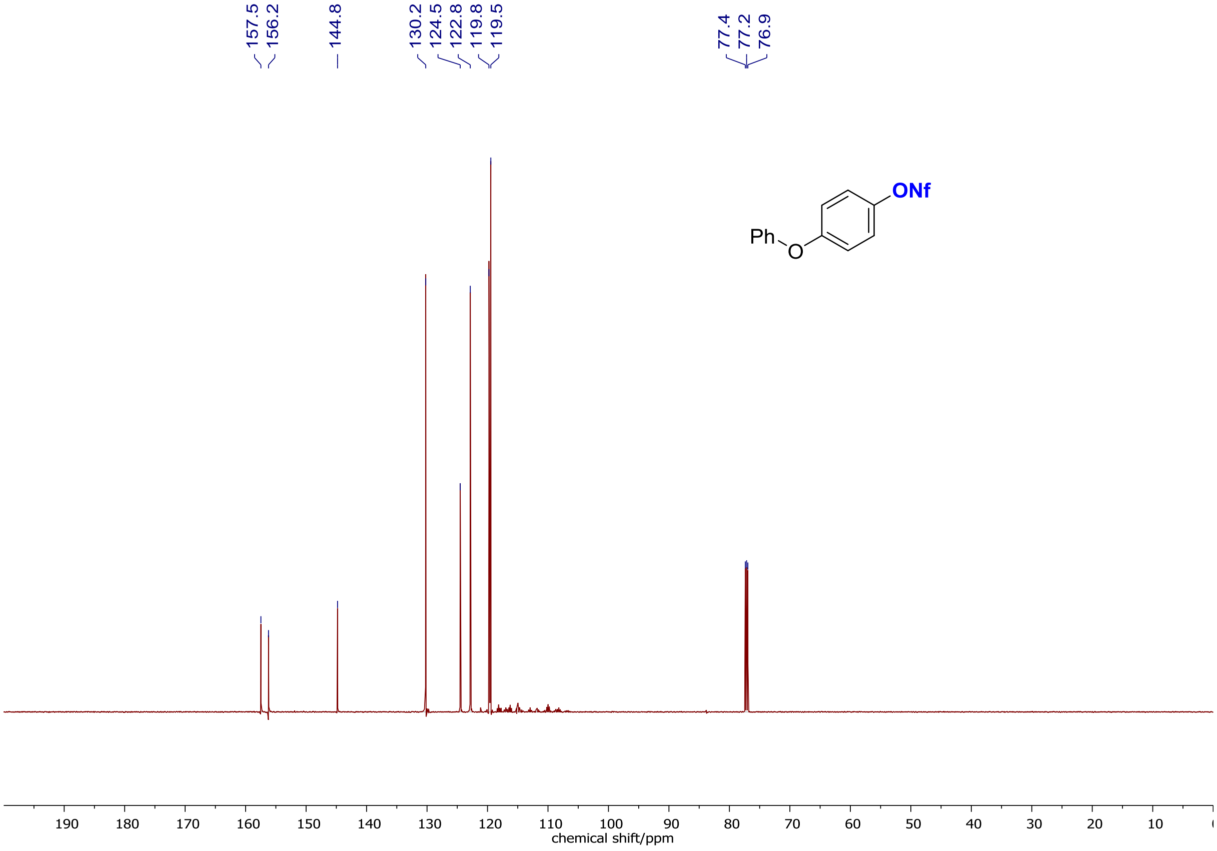
157.5
156.2
144.8
130.2
124.5
122.8
119.8
119.5
77.4
77.2
76.9
ONf
Ph
O
chemical shift/ppm

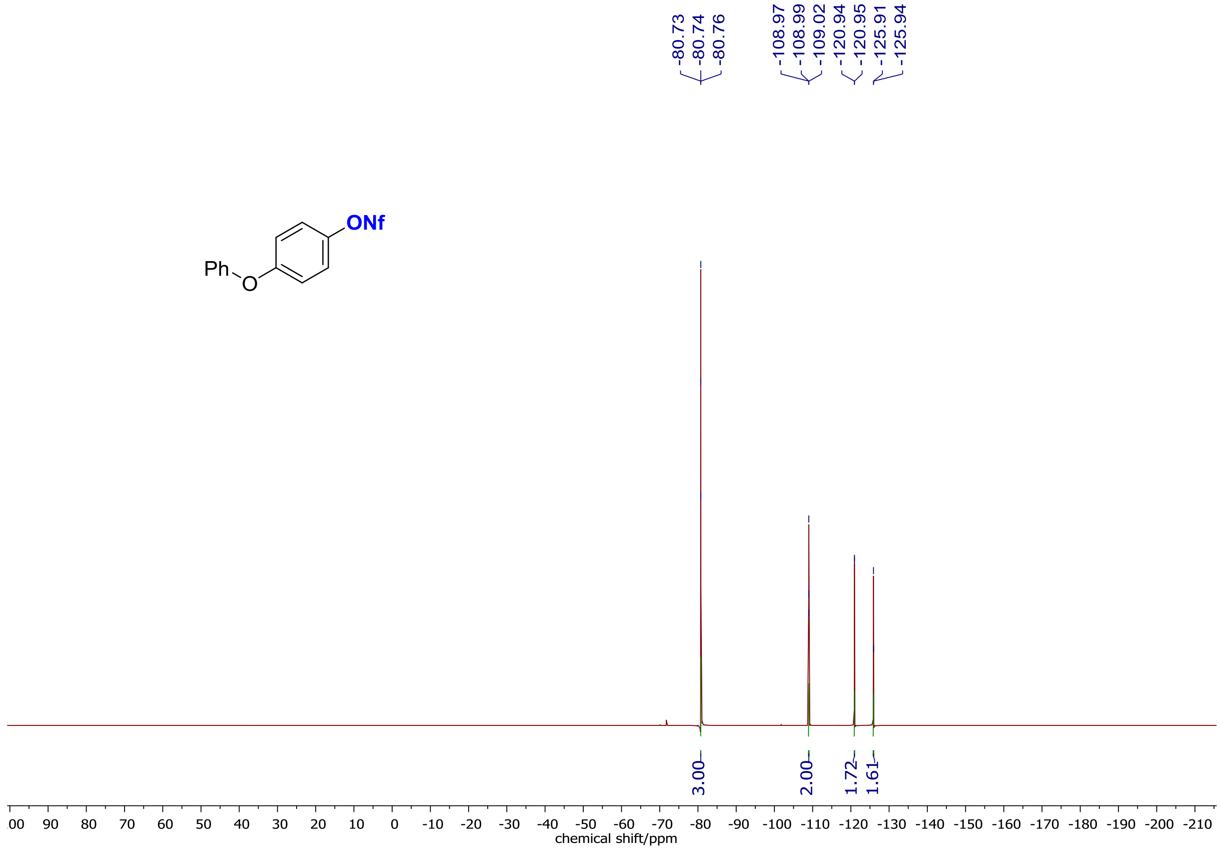

ONf
Ph
O
-80.73
-80.74
-80.76
-108.97
-108.99
-109.02
-120.94
-120.95
-125.91
-125.94
3.00
2.00
1.72
1.61
chemical shift/ppm

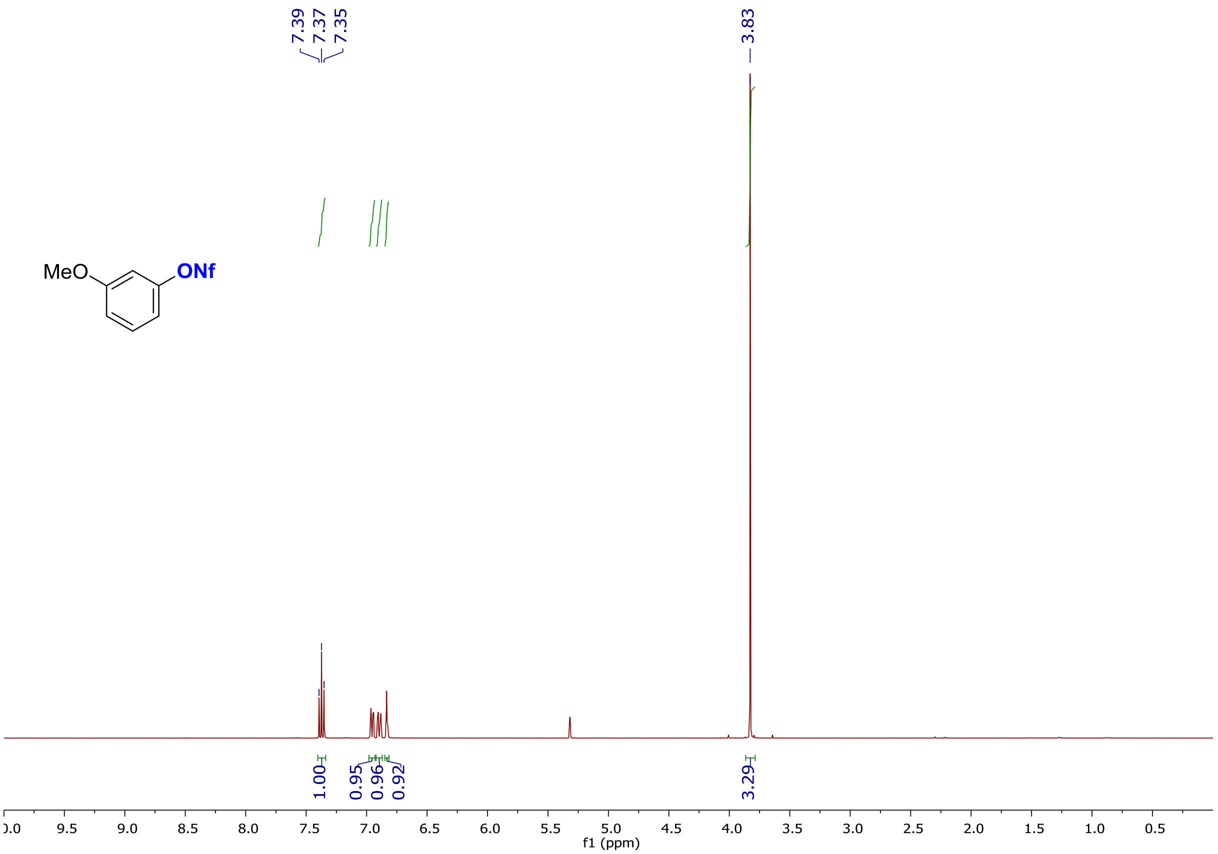
7.39
7.37
7.35
3.83
MeO
ONf
1.00
0.95
0.96
0.92
3.29
f1 (ppm)

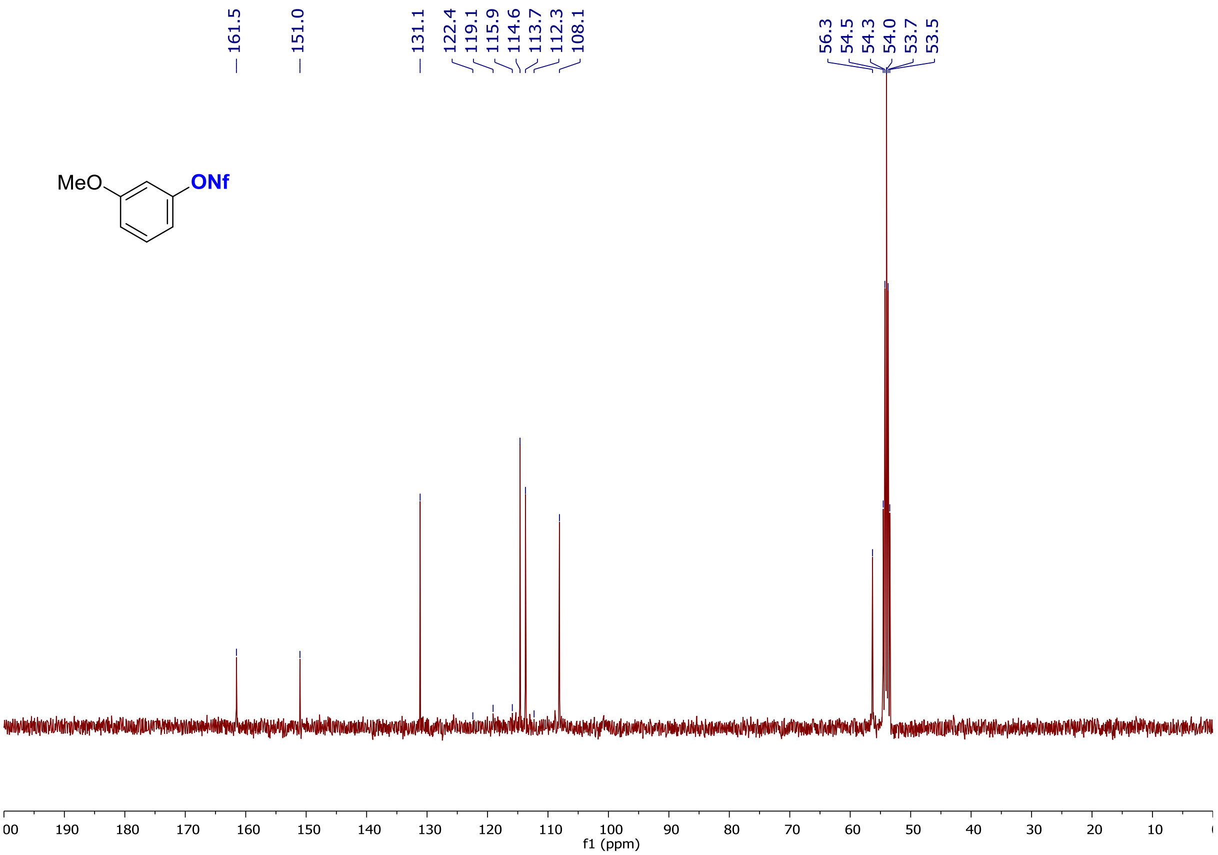
161.5
151.0
131.1
122.4
119.1
115.9
114.6
113.7
112.3
108.1
56.3
54.5
54.3
54.0
53.7
53.5
MeO
ONf
f1 (ppm)

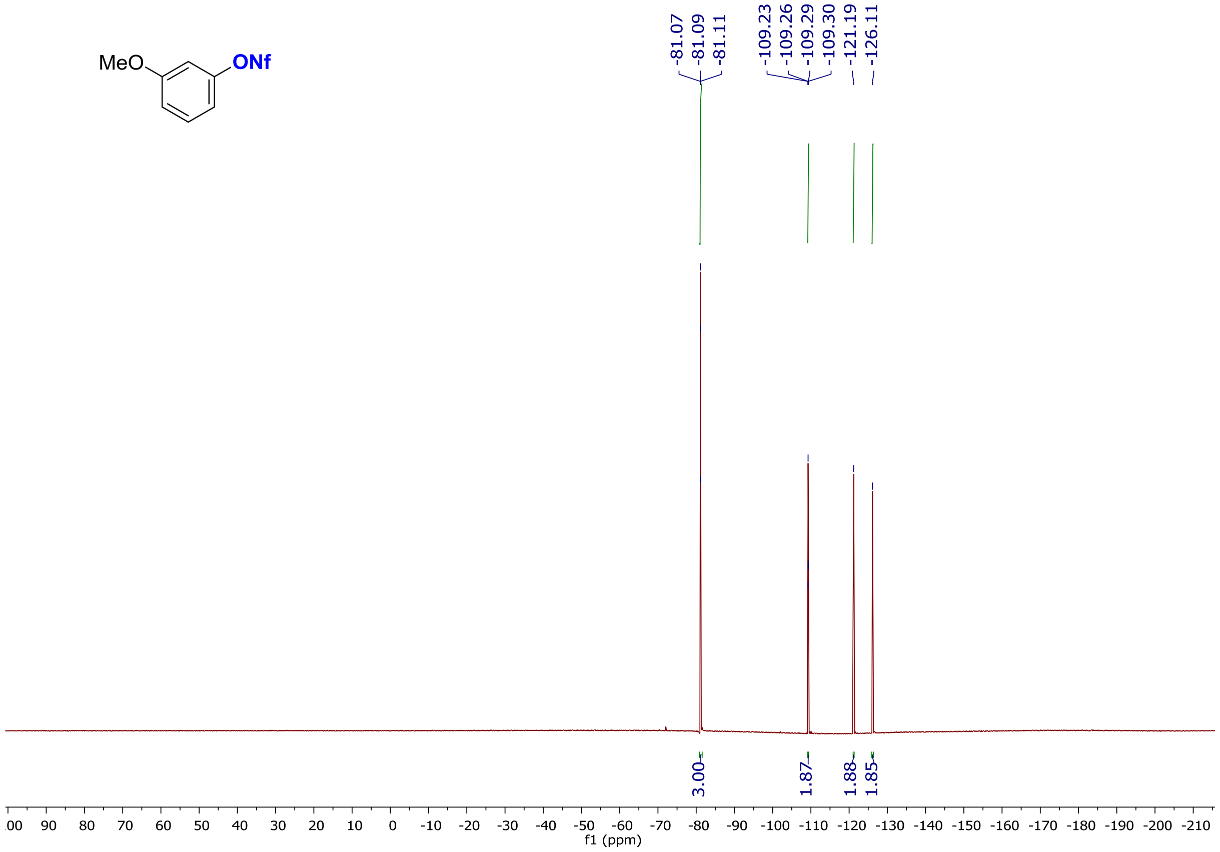

MeO
ONf
-81.07
-81.09
-81.11
-109.23
-109.26
-109.29
-109.30
-121.19
-126.11
3.00
1.87
1.88
1.85
00 90 80 70 60 50 40 30 20 10 0 -10 -20 -30 -40 -50 -60 -70 -80 -90 -100 -110 -120 -130 -140 -150 -160 -170 -180 -190 -200 -210
f1 (ppm)

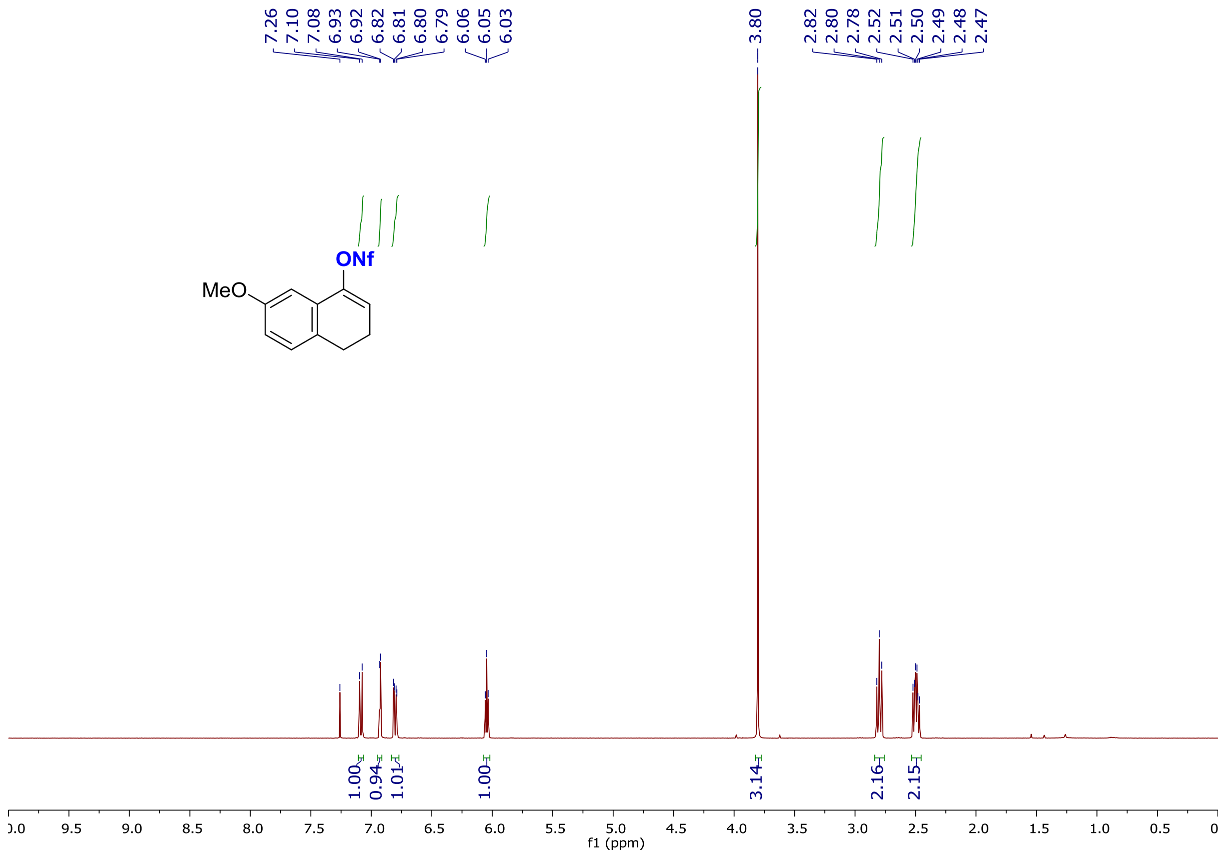

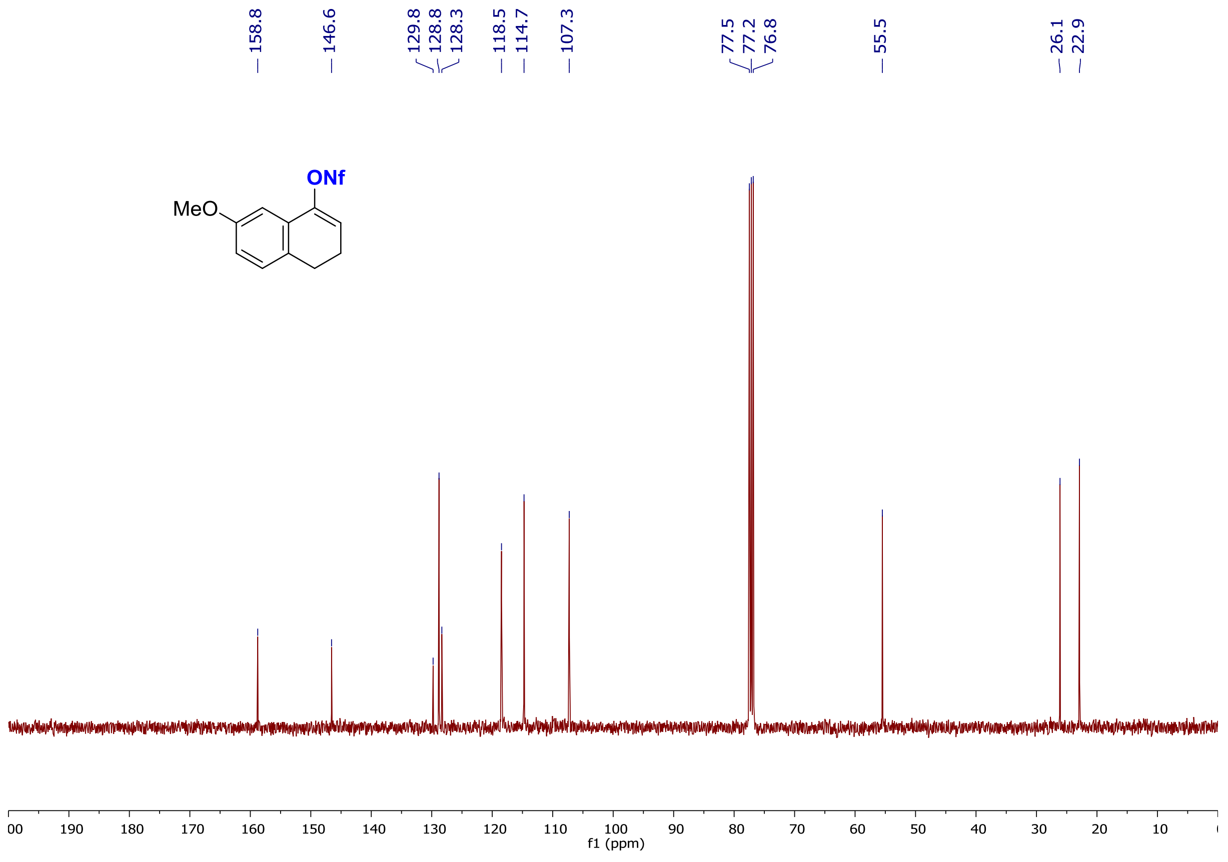

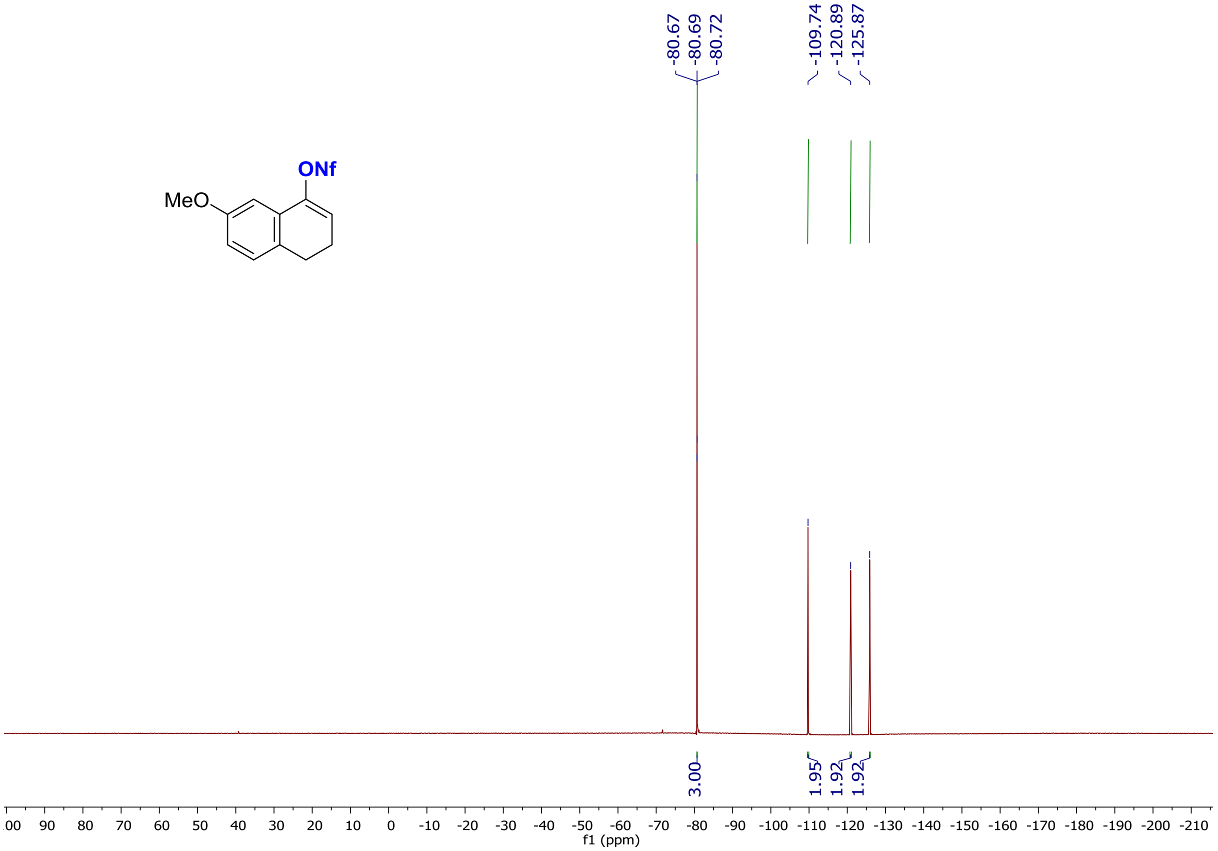

MeO
ONf
-80.67
-80.69
-80.72
-109.74
-120.89
-125.87
3.00
1.95
1.92
1.92
00 90 80 70 60 50 40 30 20 10 0 -10 -20 -30 -40 -50 -60 -70 -80 -90 -100 -110 -120 -130 -140 -150 -160 -170 -180 -190 -200 -210
f1 (ppm)

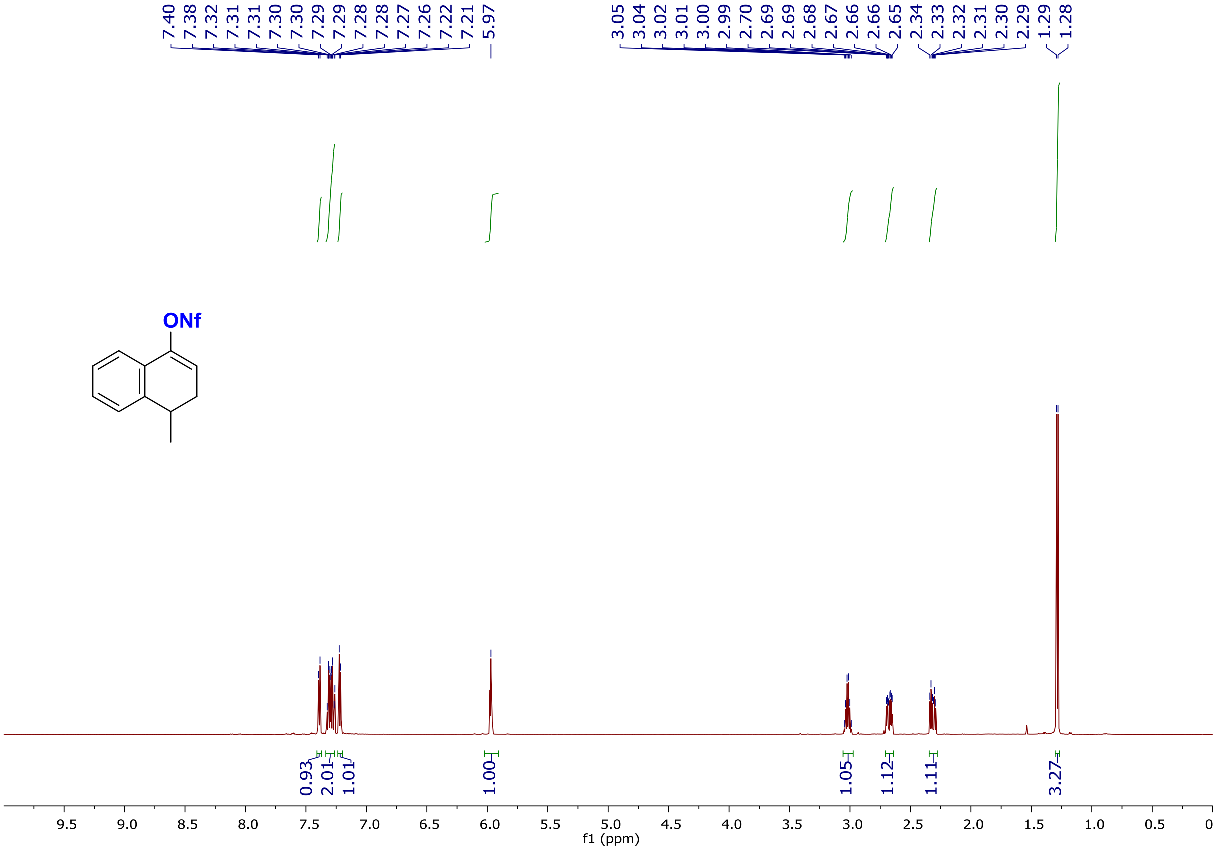
7.40 7.38 7.32 7.31 7.31 7.30 7.30 7.29 7.29 7.28 7.28 7.27 7.26 7.22 7.21 5.97
3.05 3.04 3.02 3.01 3.00 2.99 2.70 2.69 2.69 2.68 2.67 2.66 2.66 2.65 2.34 2.33 2.32 2.31 2.30 2.29 1.29 1.28
ONf
0.93 2.01 1.01 1.00 1.05 1.12 1.11 3.27
f1 (ppm)

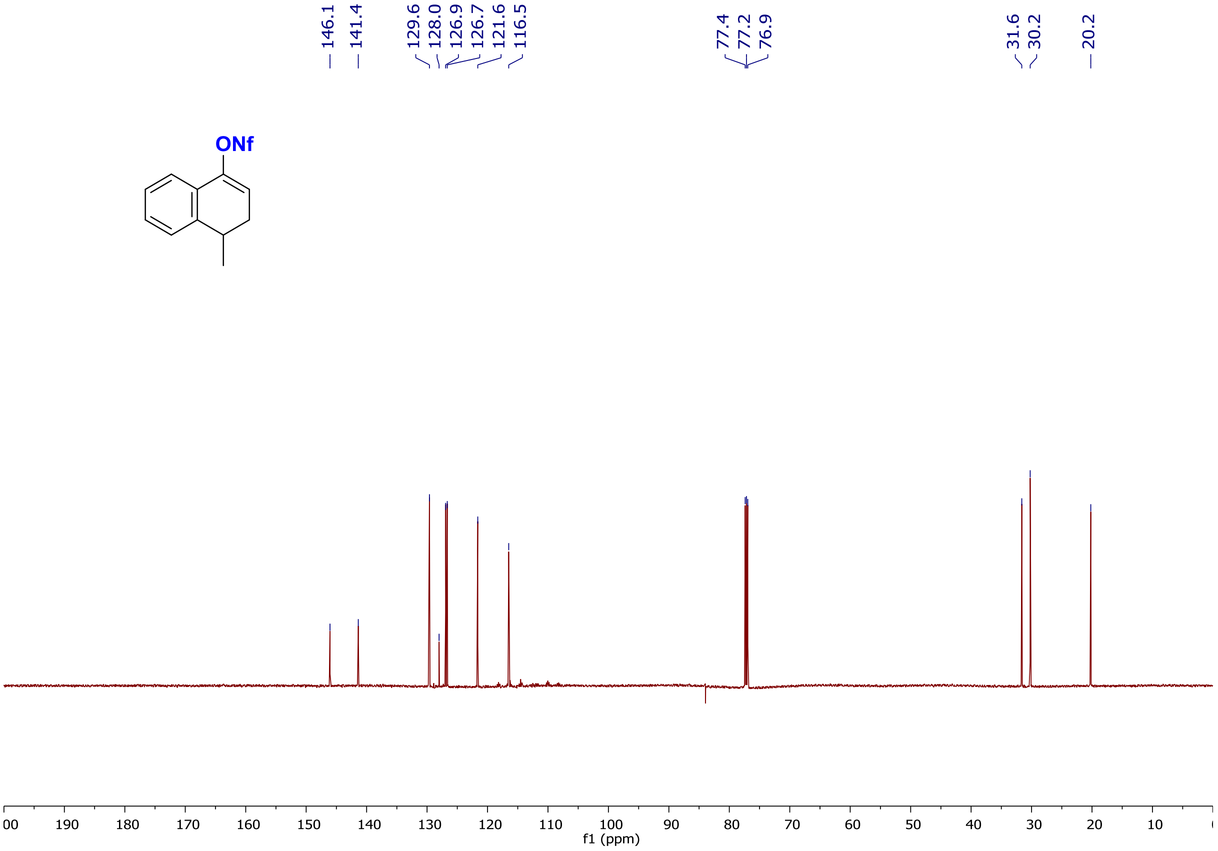
146.1 141.4 129.6 128.0 126.9 126.7 121.6 116.5
77.4 77.2 76.9
31.6 30.2 20.2
ONf
f1 (ppm)

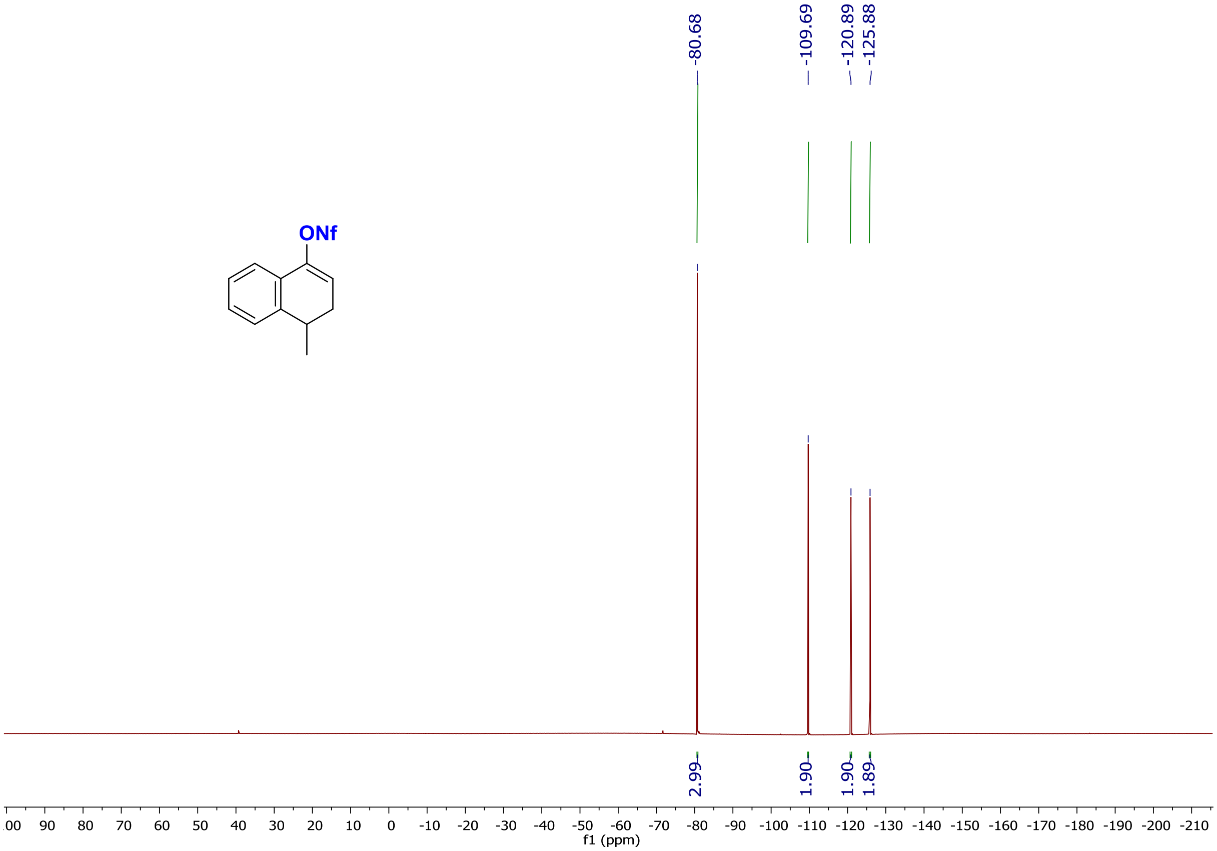
-80.68
-109.69
-120.89
-125.88
ONf
2.99
1.90
1.90
1.89
00 90 80 70 60 50 40 30 20 10 0 -10 -20 -30 -40 -50 -60 -70 -80 -90 -100 -110 -120 -130 -140 -150 -160 -170 -180 -190 -200 -210
f1 (ppm)

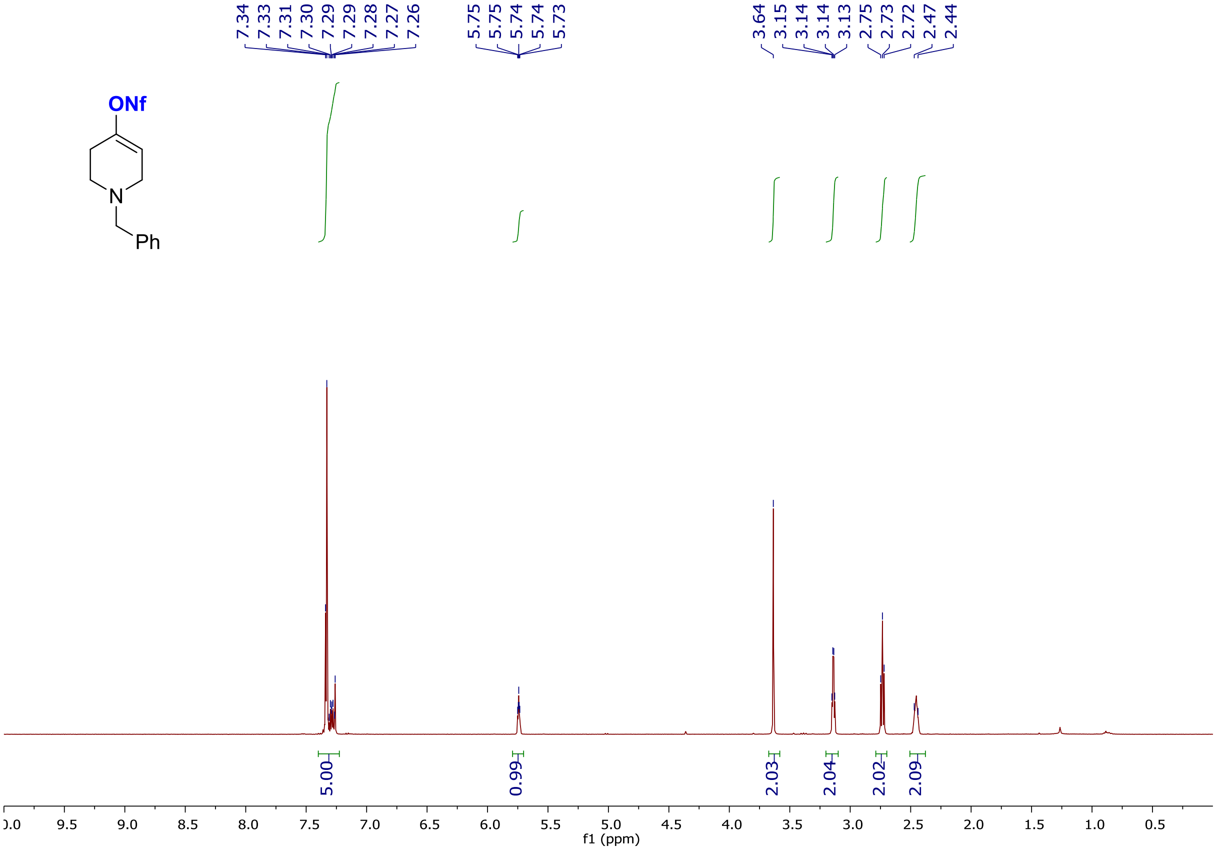

ONf
N
Ph
7.34
7.33
7.31
7.30
7.29
7.29
7.28
7.27
7.26
5.75
5.75
5.74
5.74
5.73
3.64
3.15
3.14
3.14
3.13
2.75
2.73
2.72
2.47
2.44
5.00
0.99
2.03
2.04
2.02
2.09
f1 (ppm)

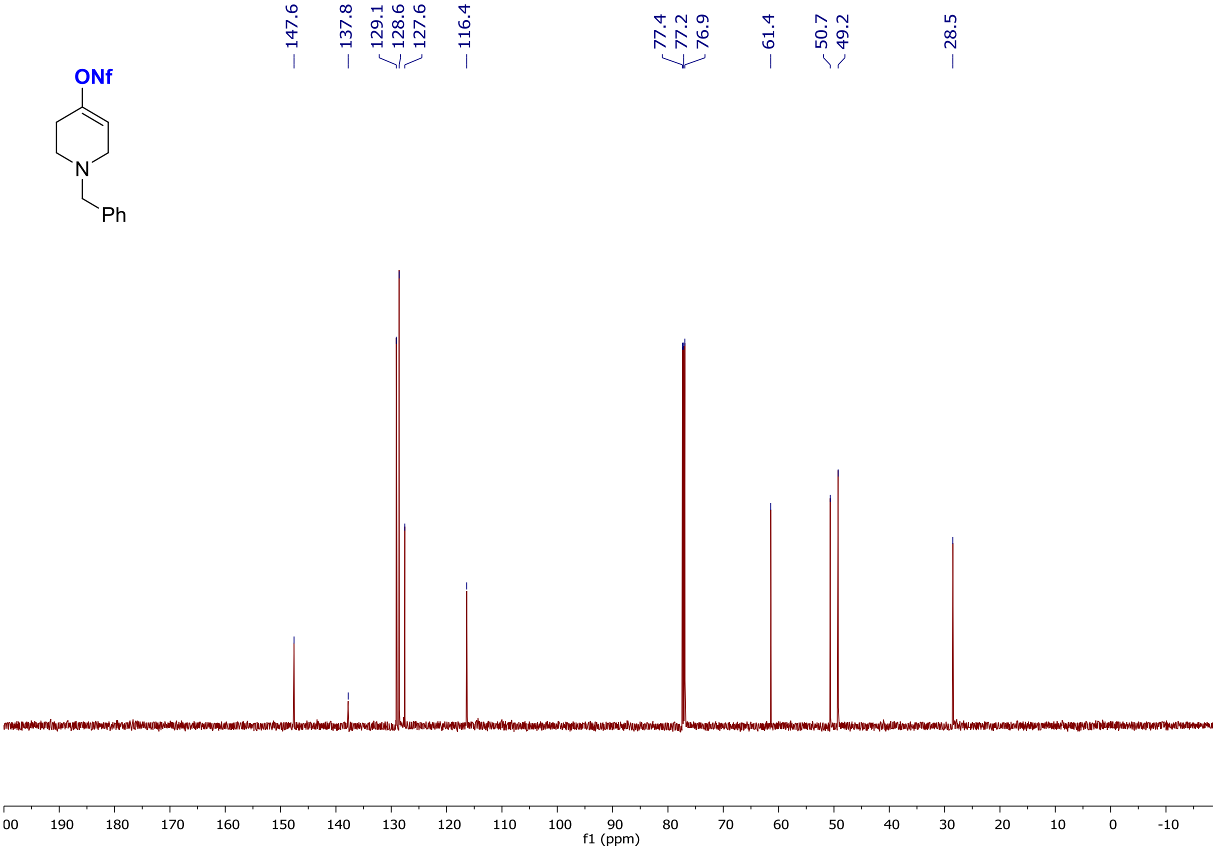

ONf
N
Ph
147.6
137.8
129.1
128.6
127.6
116.4
77.4
77.2
76.9
61.4
50.7
49.2
28.5
f1 (ppm)

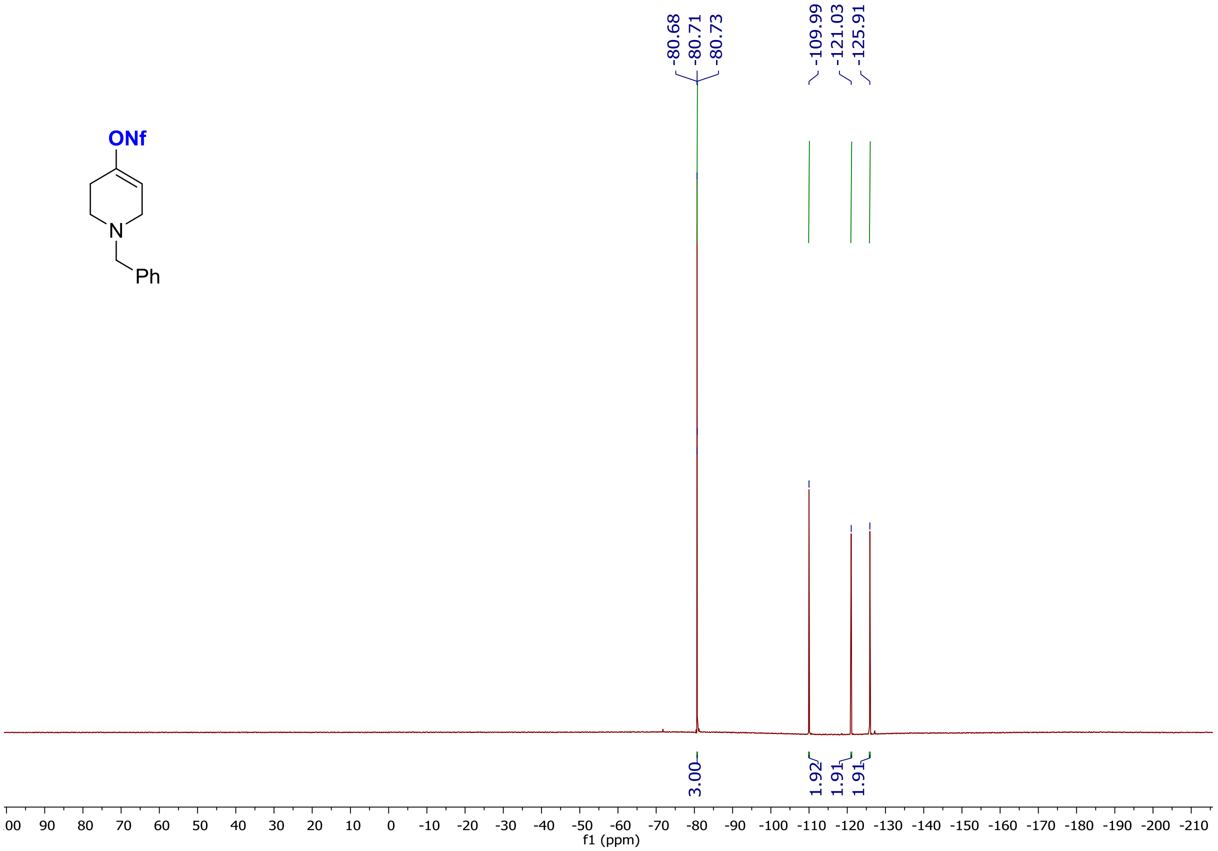
ONf
N
Ph
-80.68
-80.71
-80.73
-109.99
-121.03
-125.91
3.00
1.92
1.91
1.91
00 90 80 70 60 50 40 30 20 10 0 -10 -20 -30 -40 -50 -60 -70 -80 -90 -100 -110 -120 -130 -140 -150 -160 -170 -180 -190 -200 -210
f1 (ppm)

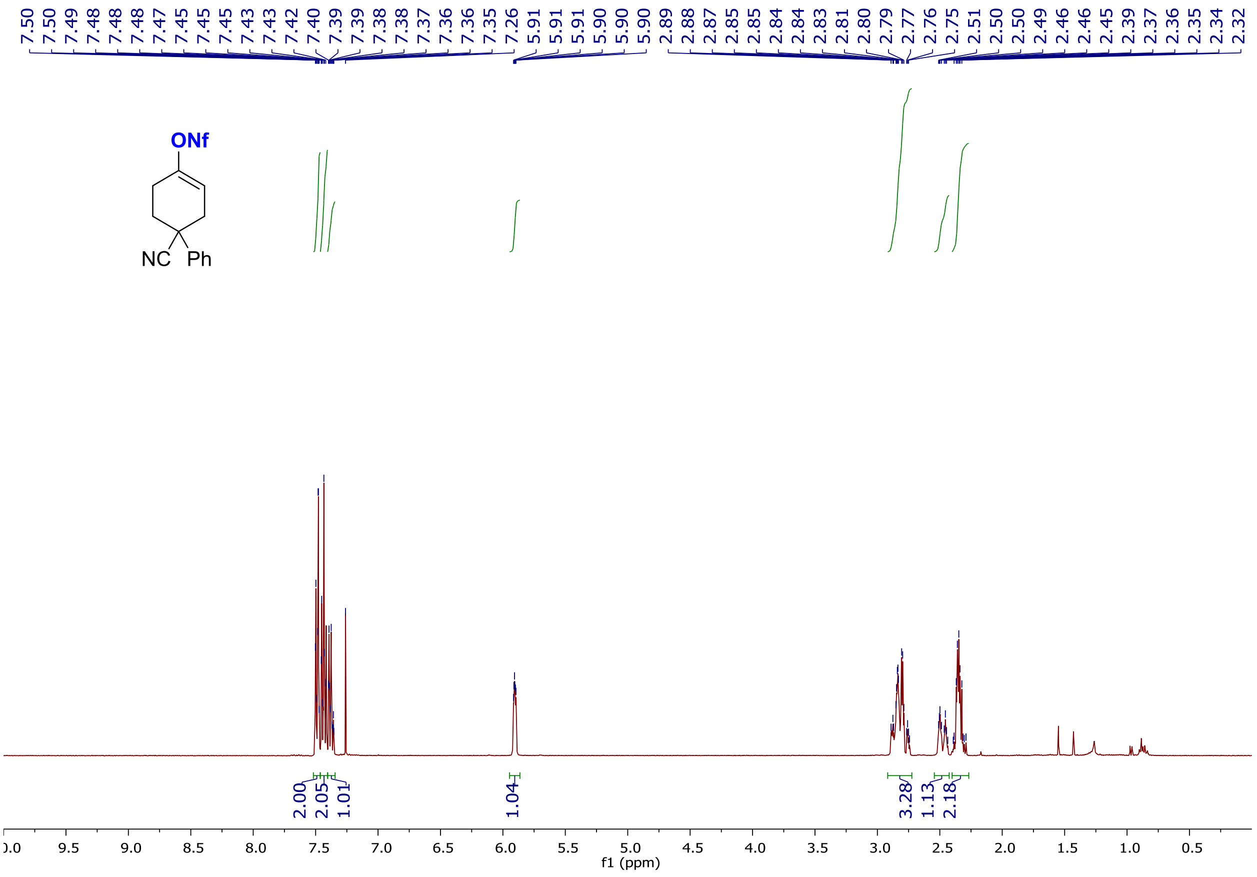

7.50
7.50
7.49
7.48
7.48
7.48
7.47
7.45
7.45
7.45
7.43
7.43
7.42
7.40
7.39
7.39
7.38
7.38
7.37
7.36
7.36
7.35
7.26
5.91
5.91
5.91
5.90
5.90
5.90
2.89
2.88
2.87
2.85
2.85
2.84
2.84
2.83
2.81
2.80
2.79
2.77
2.76
2.75
2.51
2.50
2.50
2.49
2.46
2.46
2.45
2.39
2.37
2.36
2.35
2.34
2.32
ONf
NC
Ph
2.00
2.05
1.01
1.04
3.28
1.13
2.18
9.5
9.0
8.5
8.0
7.5
7.0
6.5
6.0
5.5
5.0
4.5
4.0
3.5
3.0
2.5
2.0
1.5
1.0
0.5
f1 (ppm)

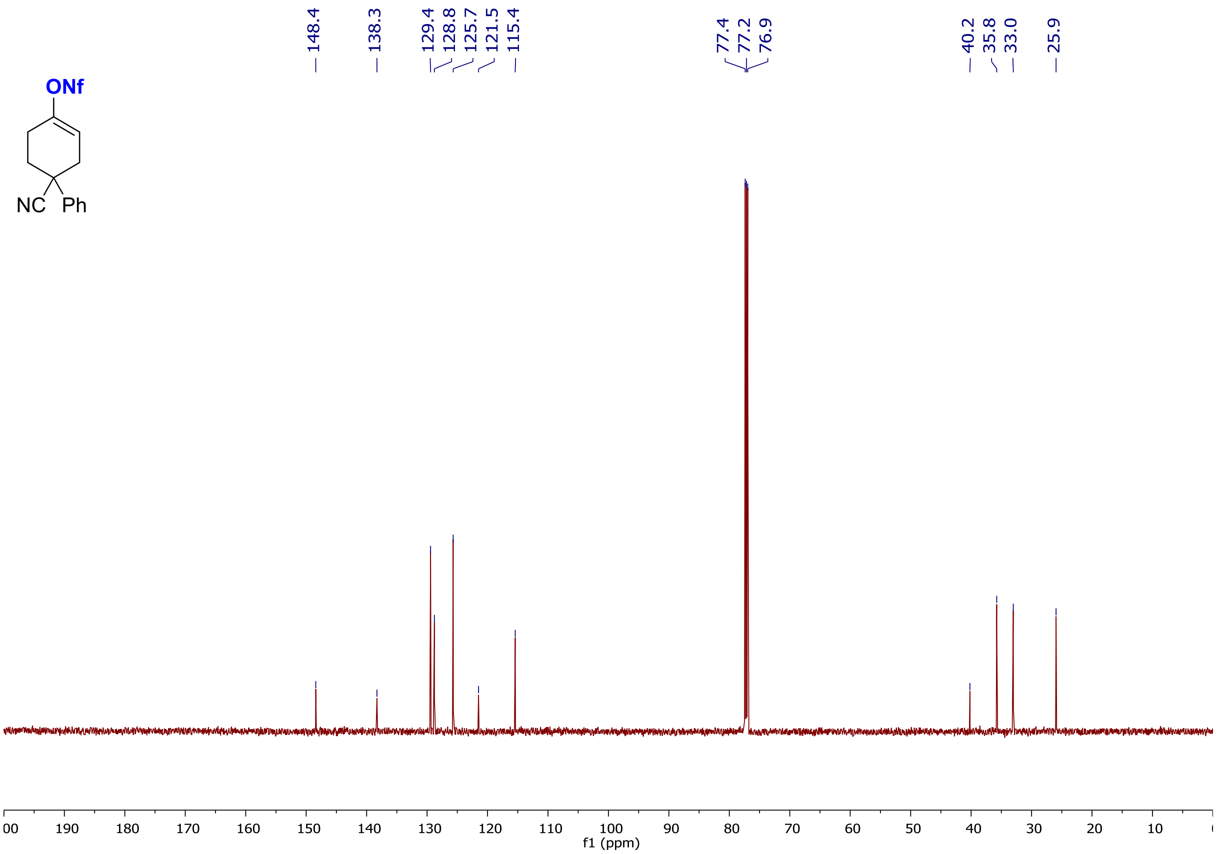
ONf
NC Ph
148.4
138.3
129.4
128.8
125.7
121.5
115.4
77.4
77.2
76.9
40.2
35.8
33.0
25.9
f1 (ppm)

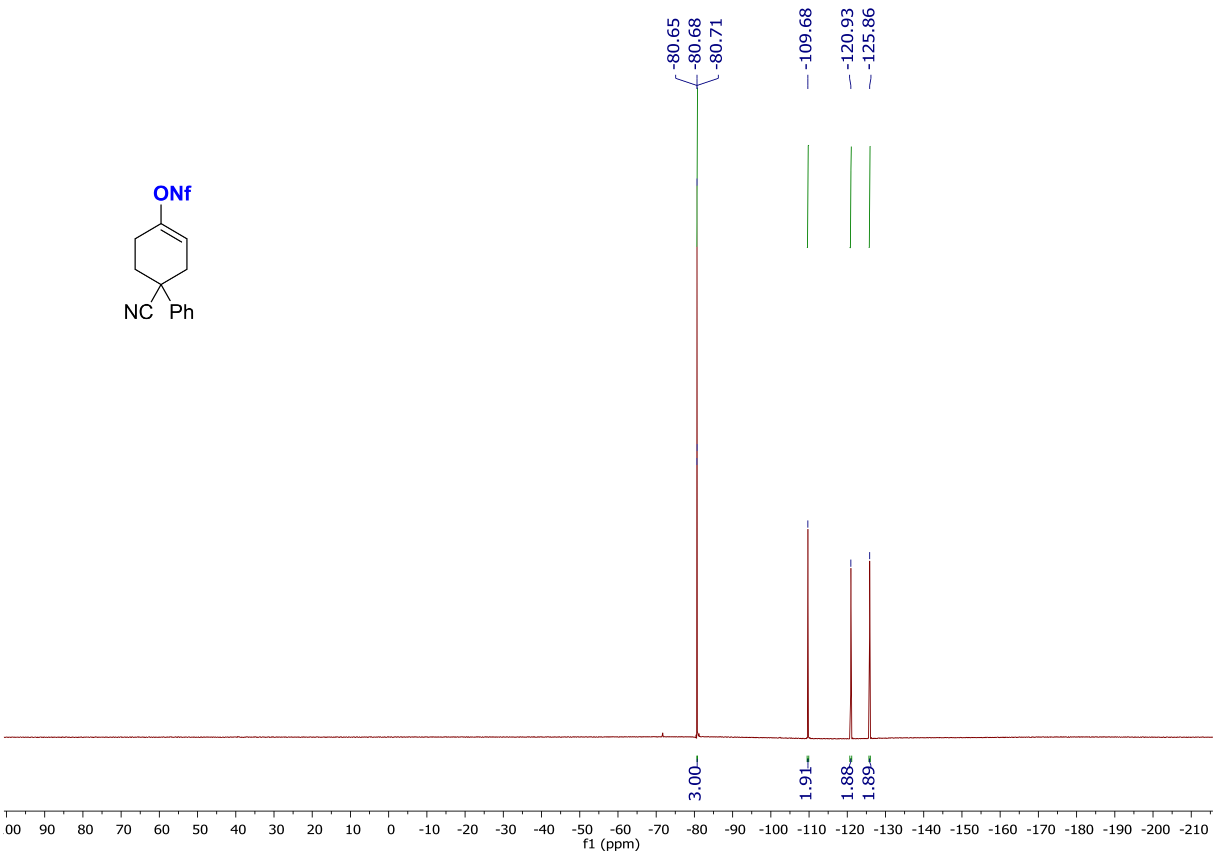
ONf
NC Ph
-80.65
-80.68
-80.71
-109.68
-120.93
-125.86
3.00
1.91
1.88
1.89
f1 (ppm)

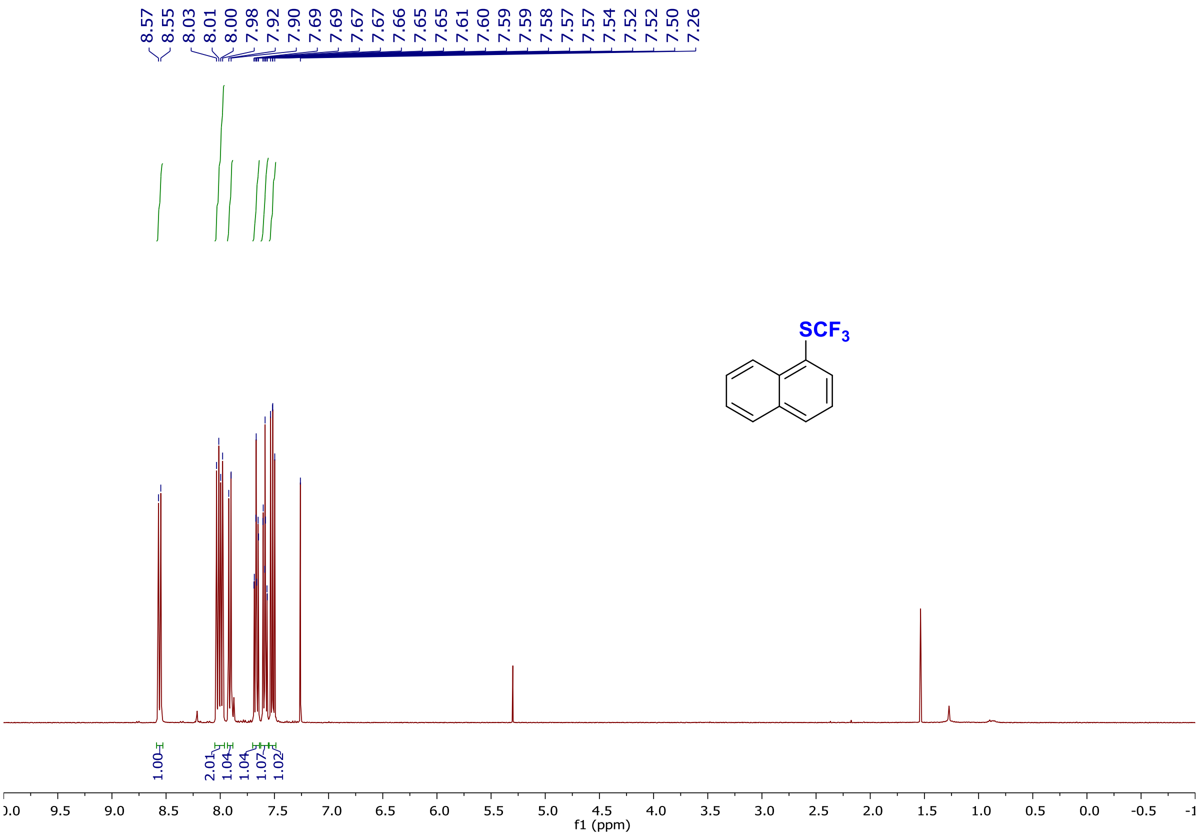

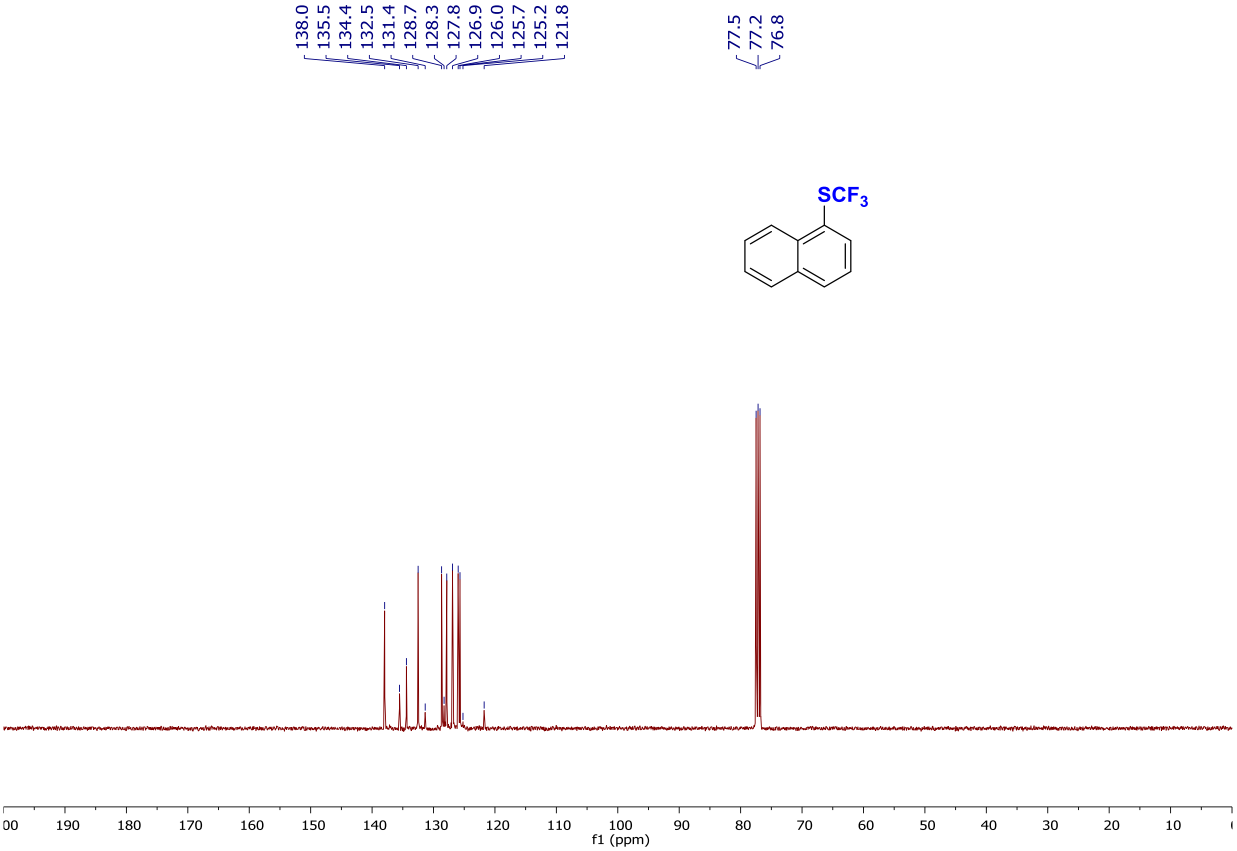

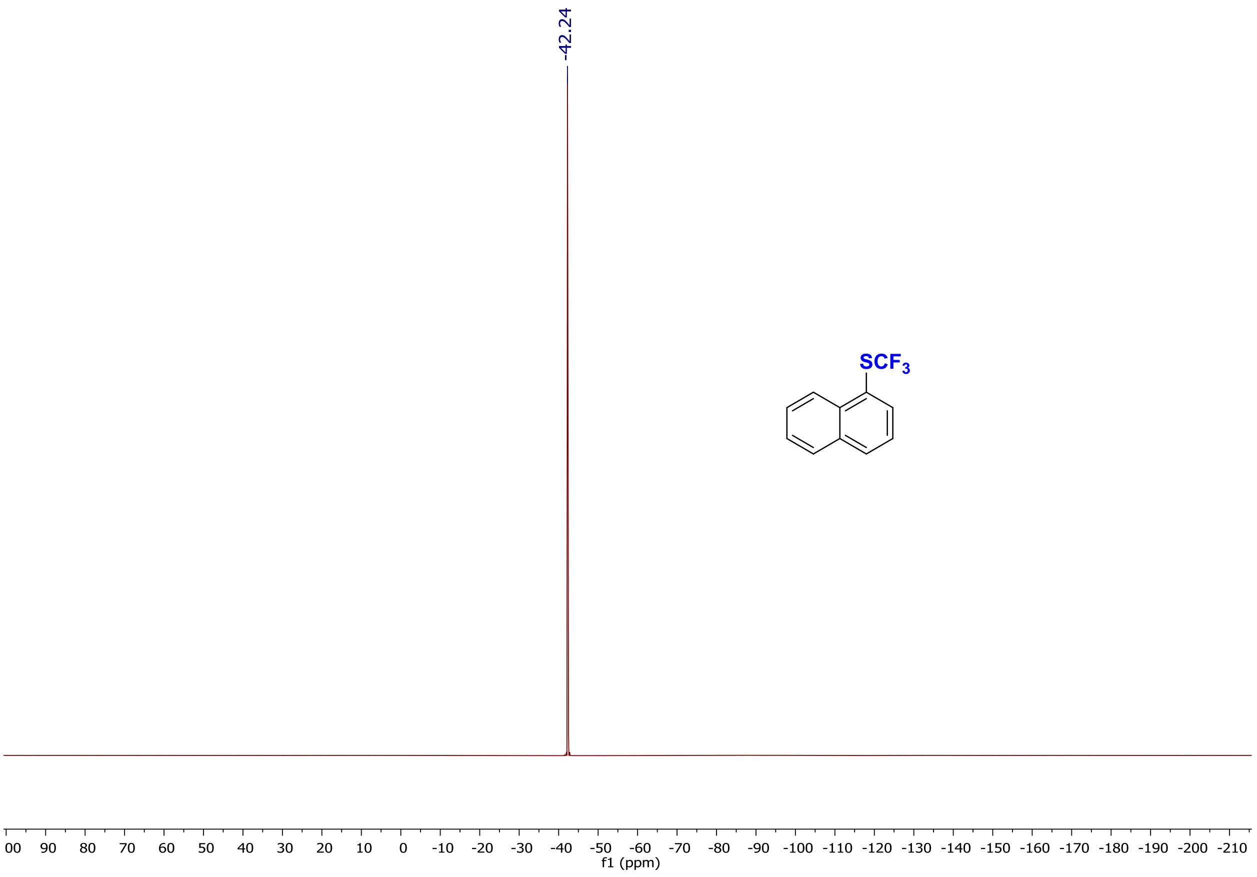

-42.24
SCF3
00 90 80 70 60 50 40 30 20 10 0 -10 -20 -30 -40 -50 -60 -70 -80 -90 -100 -110 -120 -130 -140 -150 -160 -170 -180 -190 -200 -210
f1 (ppm)

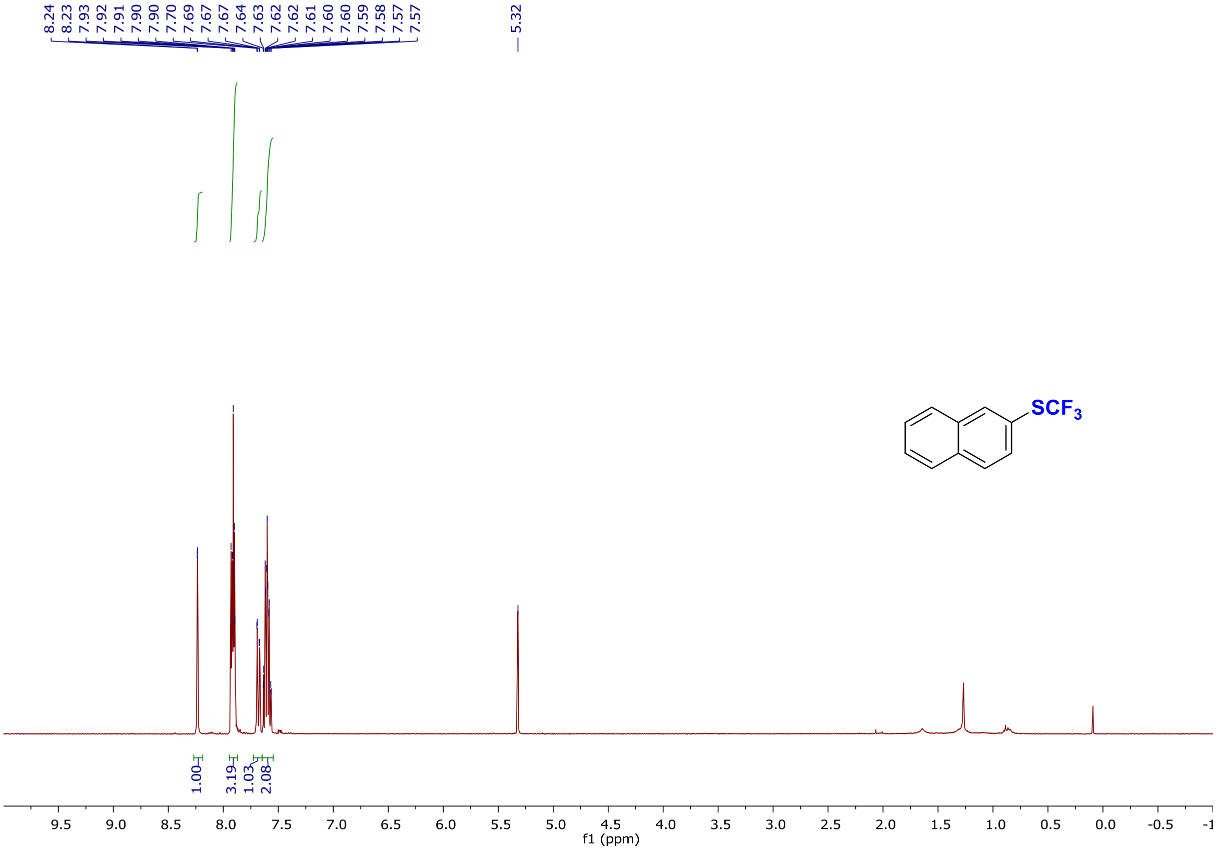
8.24 8.23 7.93 7.92 7.91 7.90 7.90 7.70 7.69 7.67 7.67 7.64 7.63 7.62 7.62 7.61 7.60 7.60 7.59 7.58 7.57 7.57
5.32
SCF3
1.00 3.19 1.03 2.08
9.5 9.0 8.5 8.0 7.5 7.0 6.5 6.0 5.5 5.0 4.5 4.0 3.5 3.0 2.5 2.0 1.5 1.0 0.5 0.0 -0.5 -1
f1 (ppm)

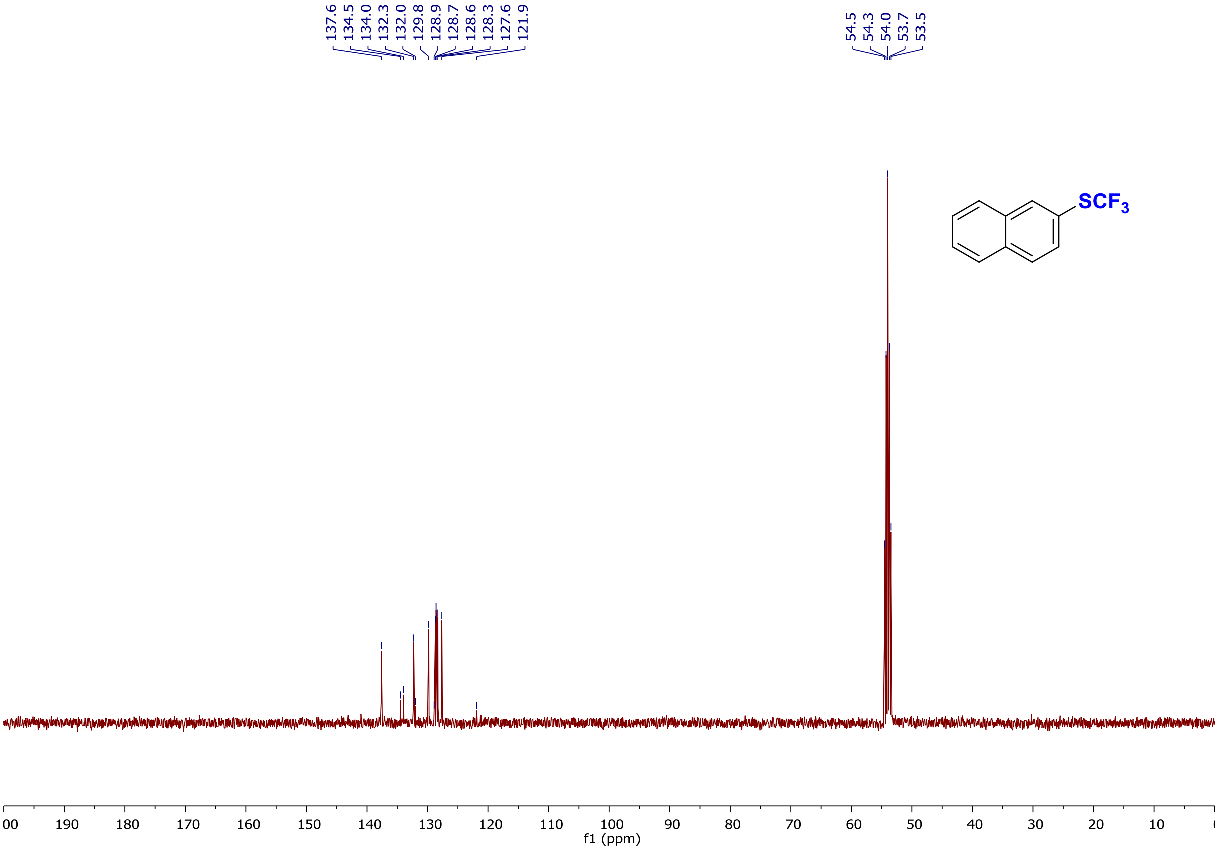
137.6 134.5 134.0 132.3 132.0 129.8 128.9 128.7 128.6 128.3 127.6 121.9
54.5 54.3 54.0 53.7 53.5
SCF3
00 190 180 170 160 150 140 130 120 110 100 90 80 70 60 50 40 30 20 10
f1 (ppm)

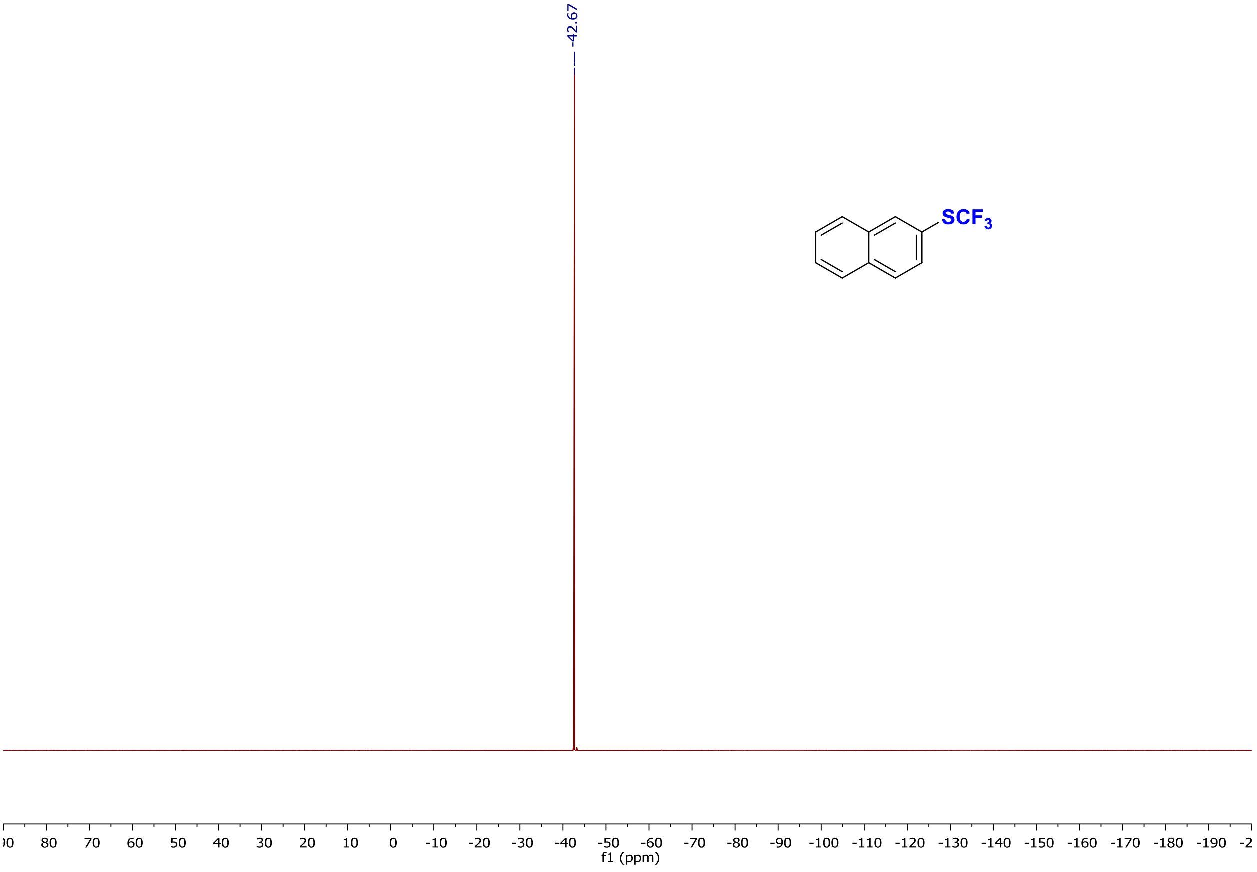
-42.67
SCF3
90 80 70 60 50 40 30 20 10 0 -10 -20 -30 -40 -50 -60 -70 -80 -90 -100 -110 -120 -130 -140 -150 -160 -170 -180 -190
f1 (ppm)

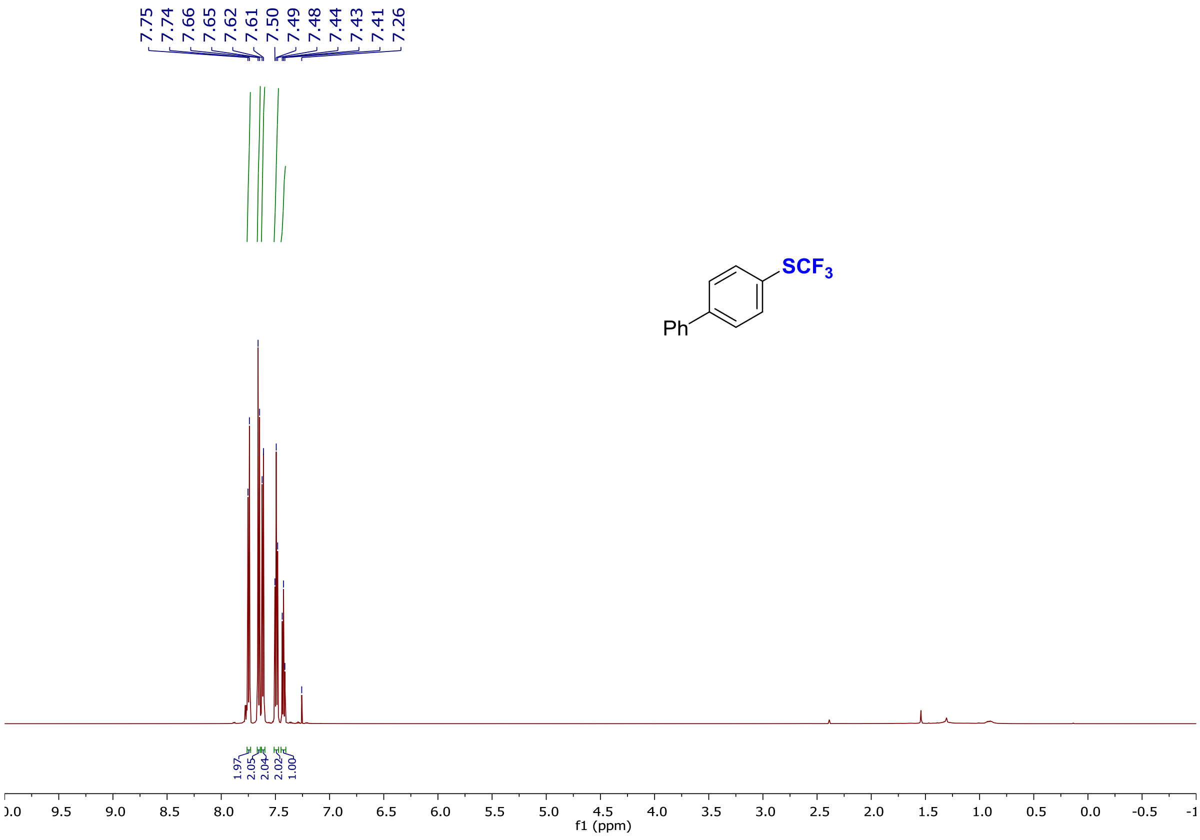
7.75 7.74 7.66 7.65 7.62 7.61 7.50 7.49 7.48 7.44 7.43 7.41 7.26
SCF3
Ph
1.97 2.05 2.04 2.02 1.00
9.5 9.0 8.5 8.0 7.5 7.0 6.5 6.0 5.5 5.0 4.5 4.0 3.5 3.0 2.5 2.0 1.5 1.0 0.5 0.0 -0.5
f1 (ppm)

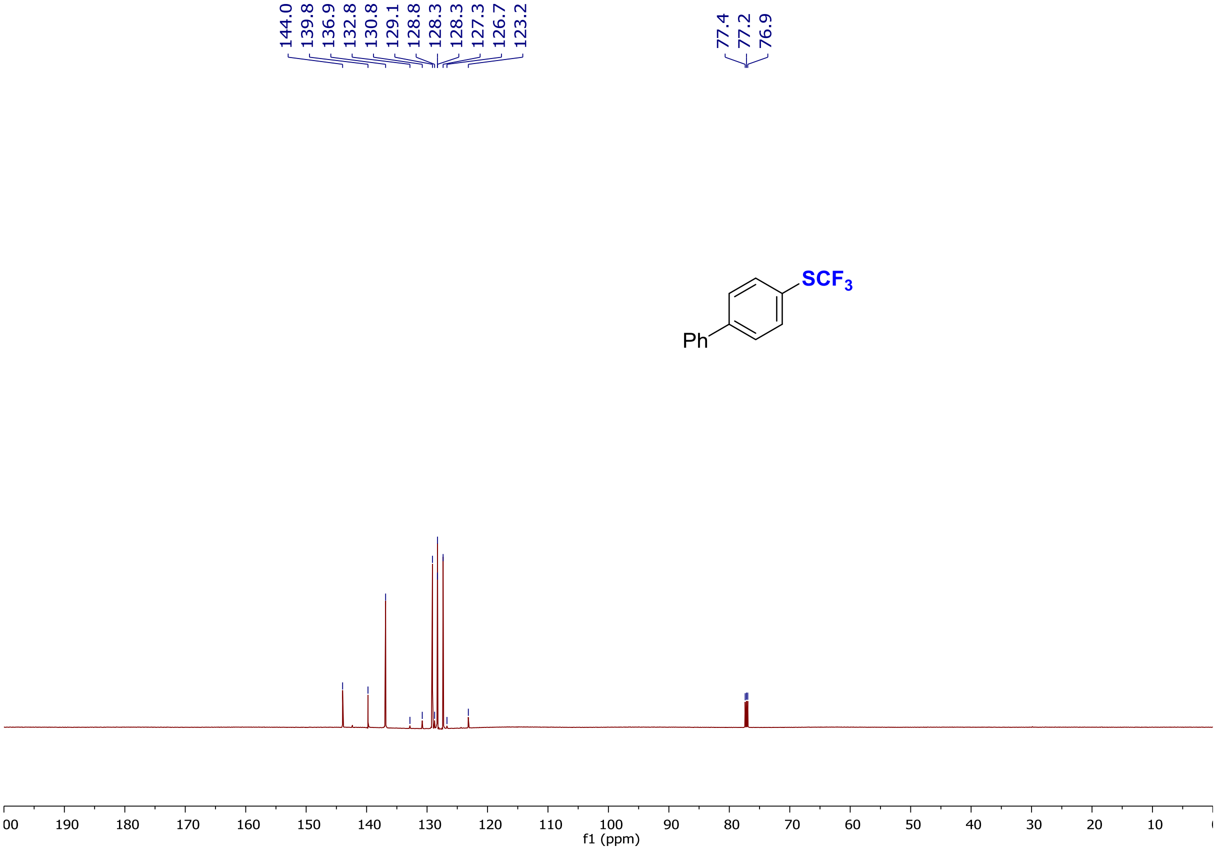
144.0
139.8
136.9
132.8
130.8
129.1
128.8
128.3
128.3
127.3
126.7
123.2
77.4
77.2
76.9
SCF3
Ph
00 190 180 170 160 150 140 130 120 110 100 90 80 70 60 50 40 30 20 10
f1 (ppm)

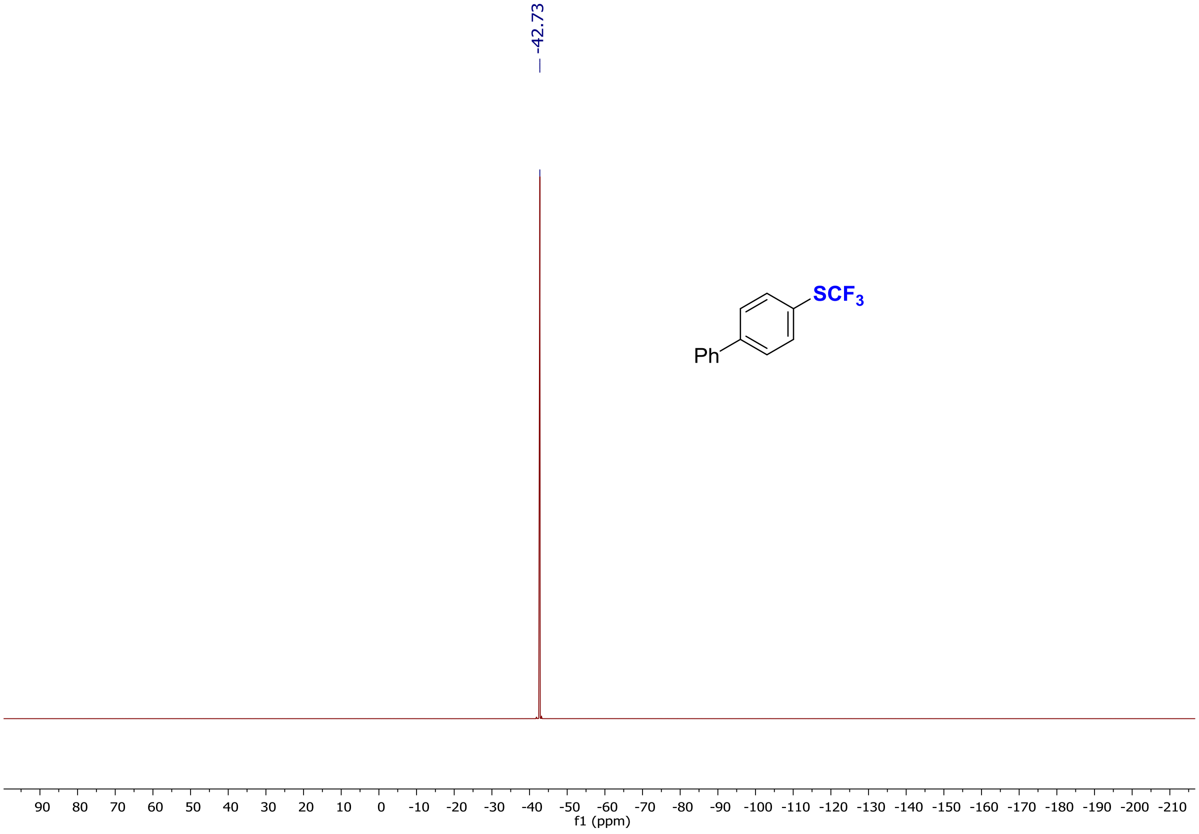
-42.73
SCF3
Ph
90 80 70 60 50 40 30 20 10 0 -10 -20 -30 -40 -50 -60 -70 -80 -90 -100 -110 -120 -130 -140 -150 -160 -170 -180 -190 -200 -210
f1 (ppm)

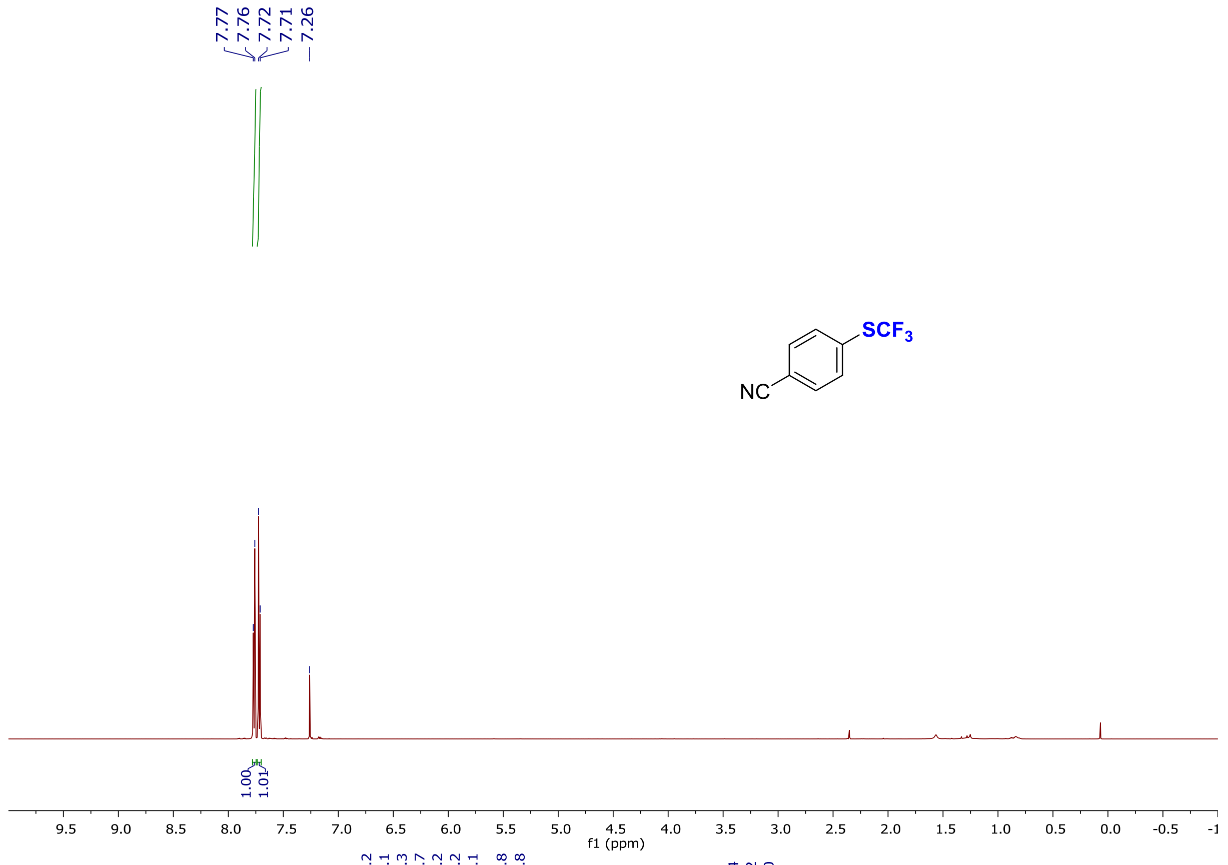
7.77
7.76
7.72
7.71
7.26
SCF3
NC
1.00
1.01
9.5 9.0 8.5 8.0 7.5 7.0 6.5 6.0 5.5 5.0 4.5 4.0 3.5 3.0 2.5 2.0 1.5 1.0 0.5 0.0 -0.5
f1 (ppm)

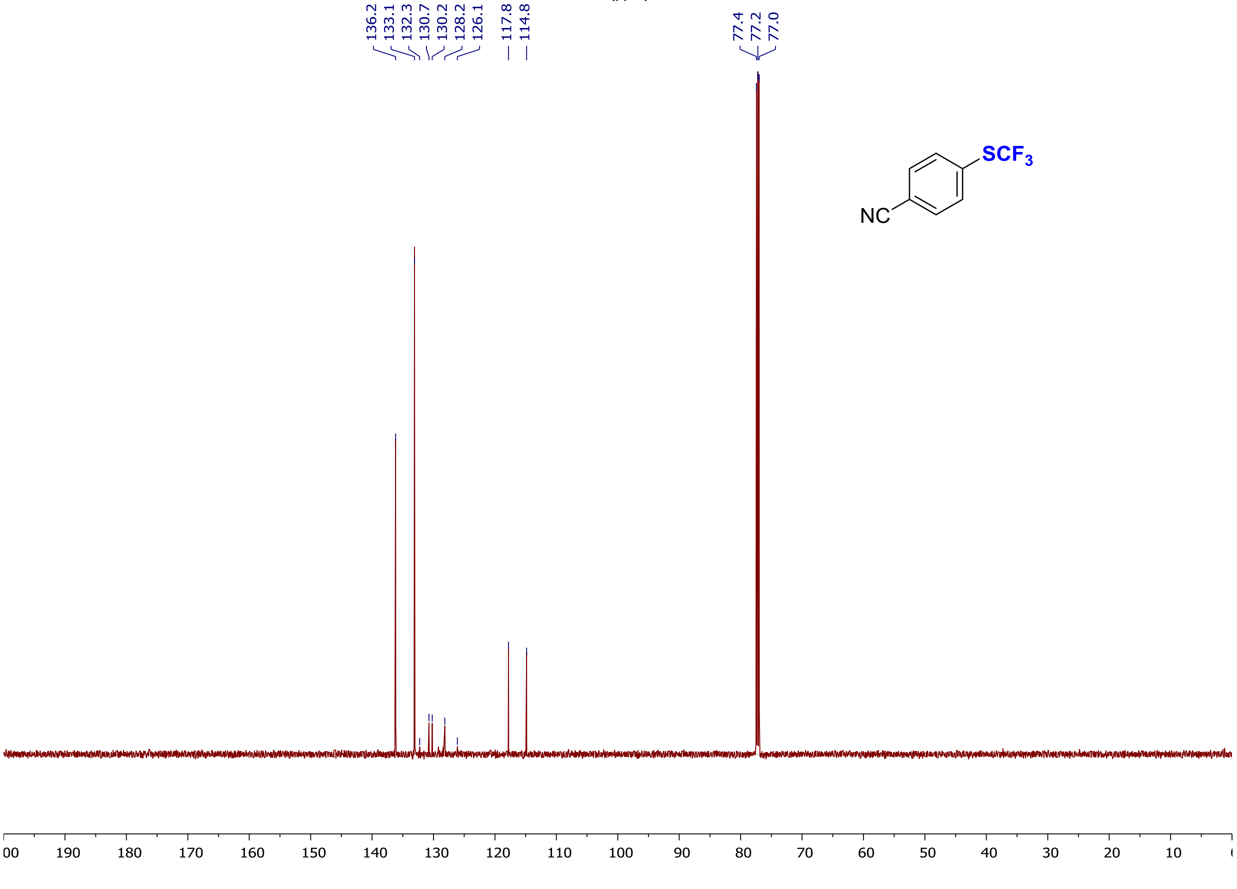
136.2
133.1
132.3
130.7
130.2
128.2
126.1
117.8
114.8
77.4
77.2
77.0
SCF3
NC
190 180 170 160 150 140 130 120 110 100 90 80 70 60 50 40 30 20 10

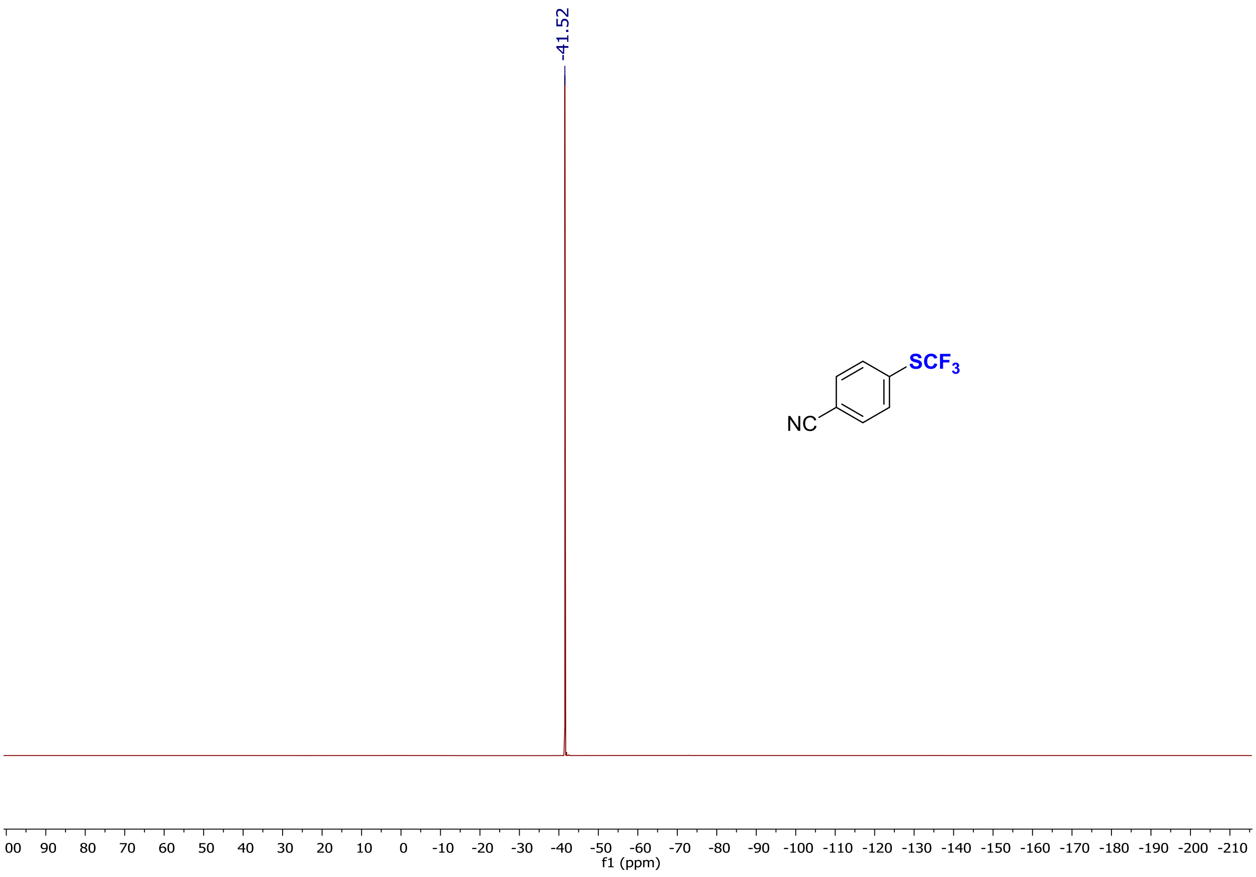

-41.52
SCF3
NC
00 90 80 70 60 50 40 30 20 10 0 -10 -20 -30 -40 -50 -60 -70 -80 -90 -100 -110 -120 -130 -140 -150 -160 -170 -180 -190 -200 -210
f1 (ppm)

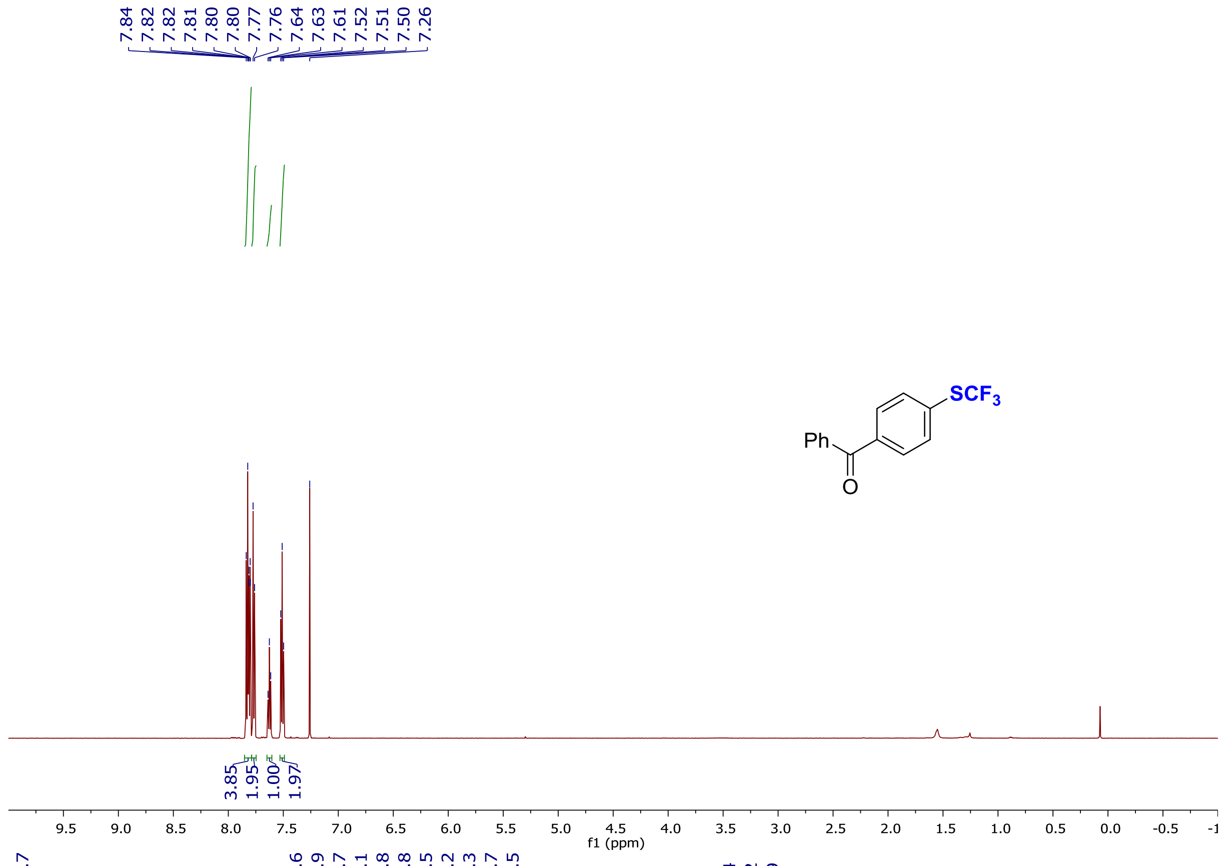
SCF3
Ph
O
f1 (ppm)

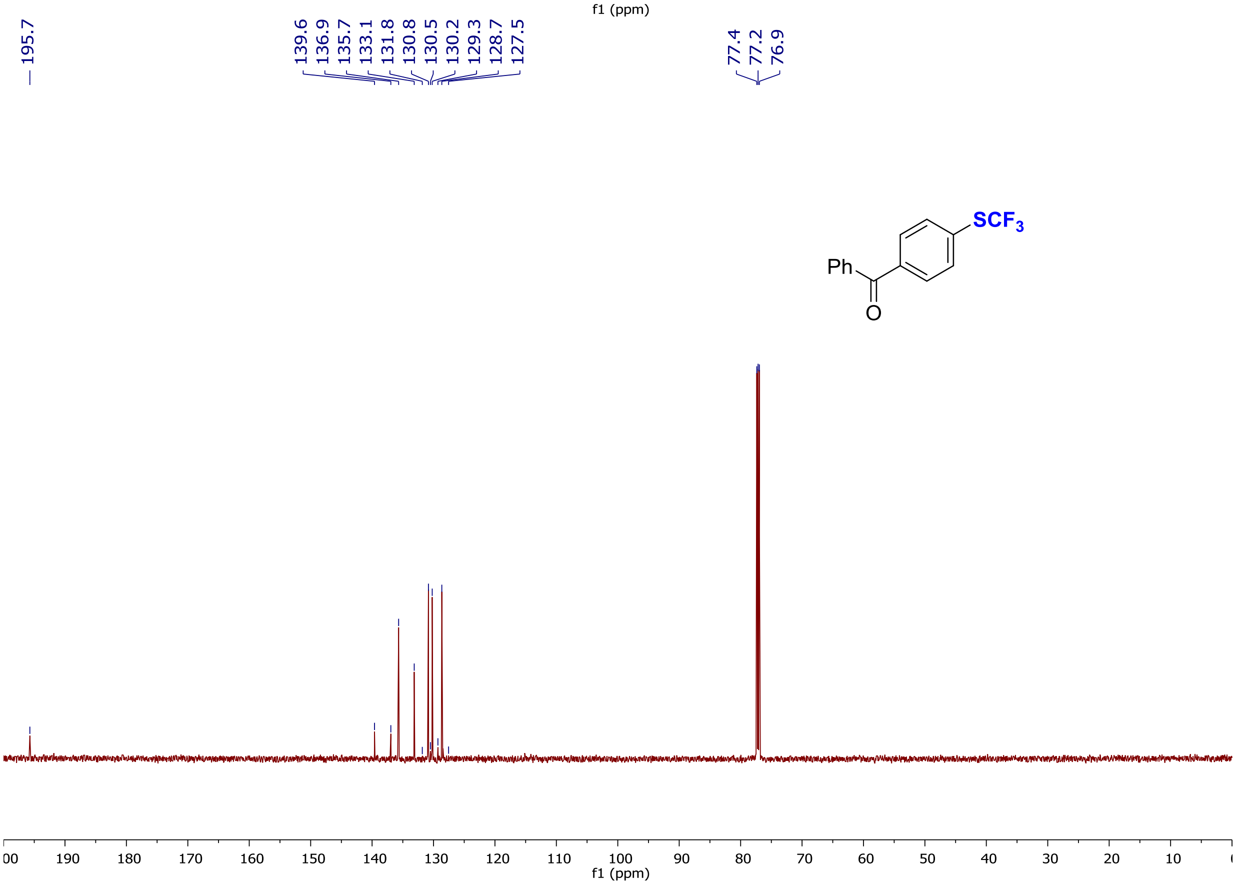
SCF3
Ph
O
f1 (ppm)

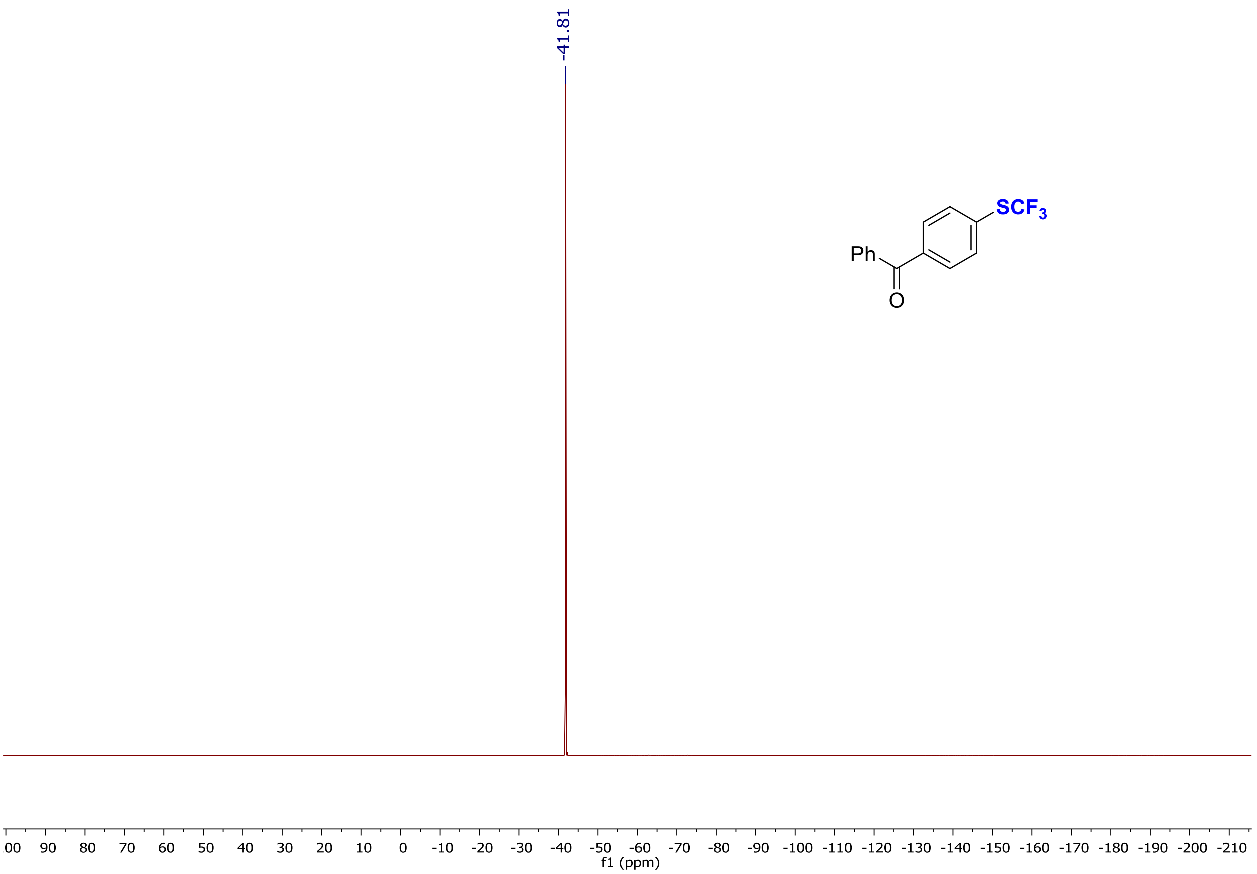
-41.81
SCF3
Ph
O
f1 (ppm)

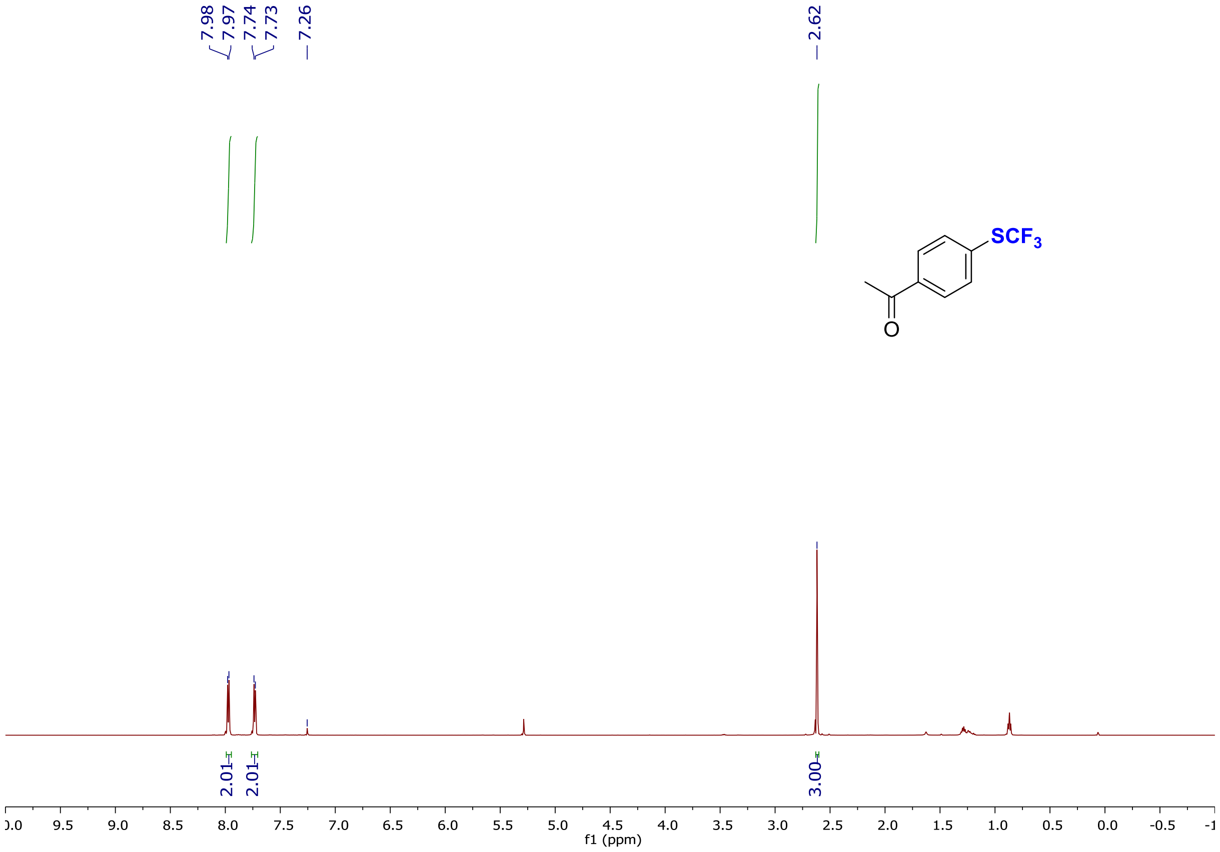
7.98
7.97
7.74
7.73
7.26
2.62
SCF3
O
2.01
2.01
3.00
f1 (ppm)

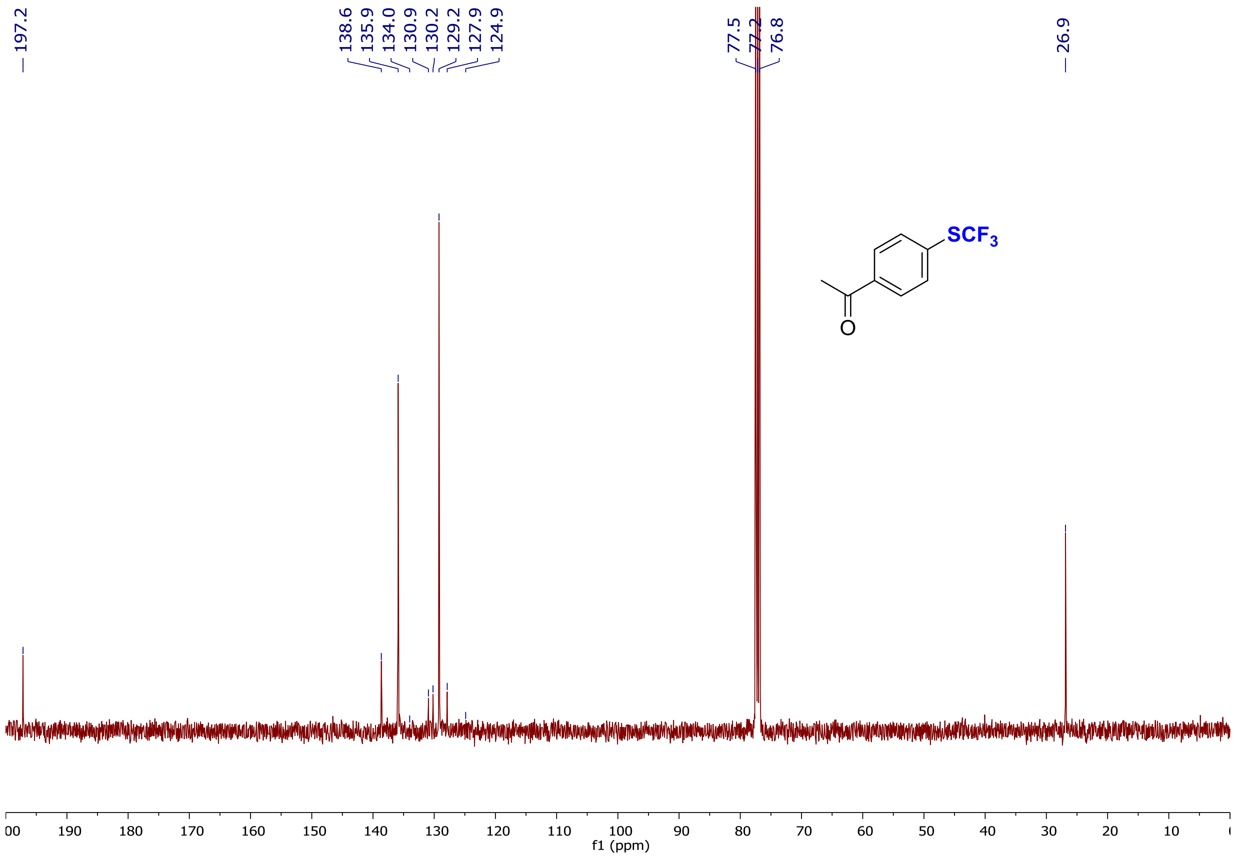

197.2
138.6
135.9
134.0
130.9
130.2
129.2
127.9
124.9
77.5
77.2
76.8
26.9
SCF3
O
f1 (ppm)

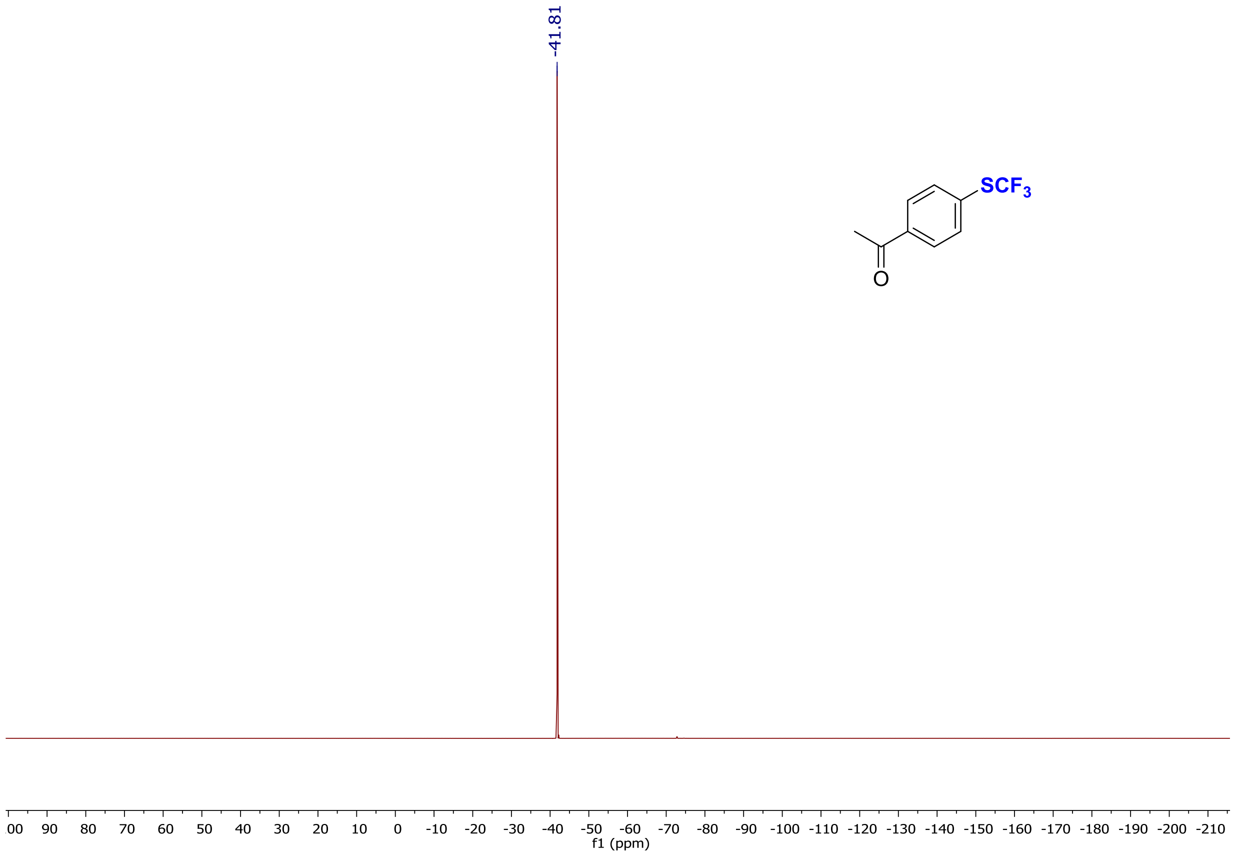

-41.81
SCF3
O
f1 (ppm)

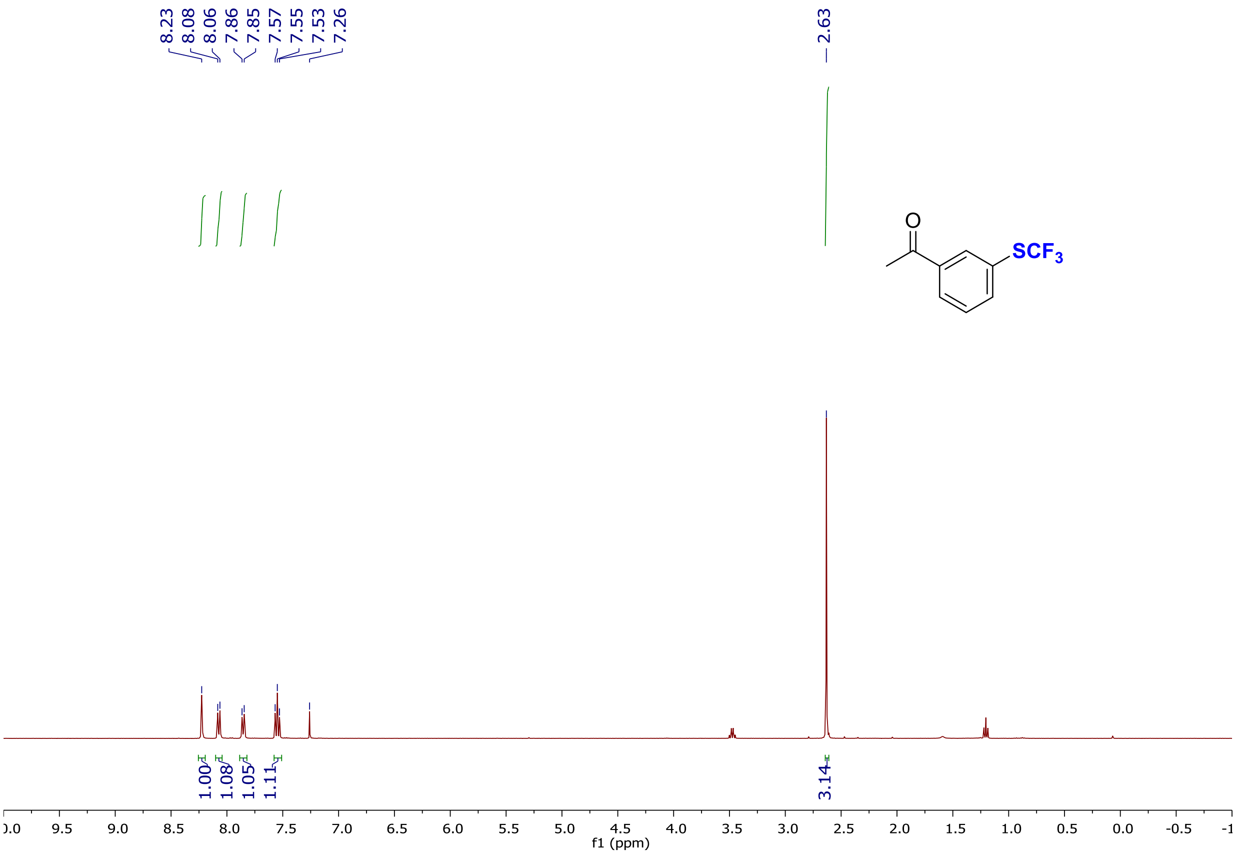
8.23
8.08
8.06
7.86
7.85
7.57
7.55
7.53
7.26
2.63
SCF3
O
1.00
1.08
1.05
1.11
3.14
f1 (ppm)

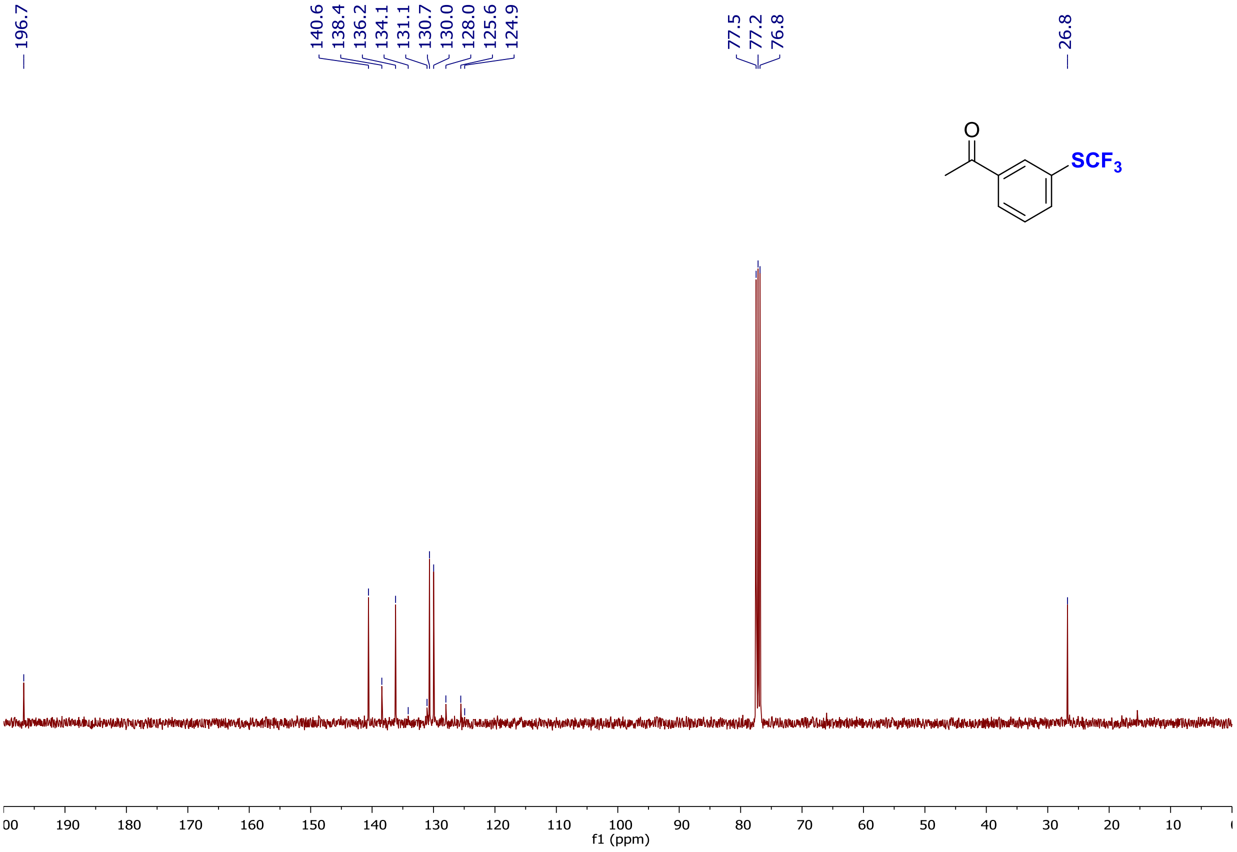
196.7
140.6
138.4
136.2
134.1
131.1
130.7
130.0
128.0
125.6
124.9
77.5
77.2
76.8
26.8
SCF3
O
f1 (ppm)

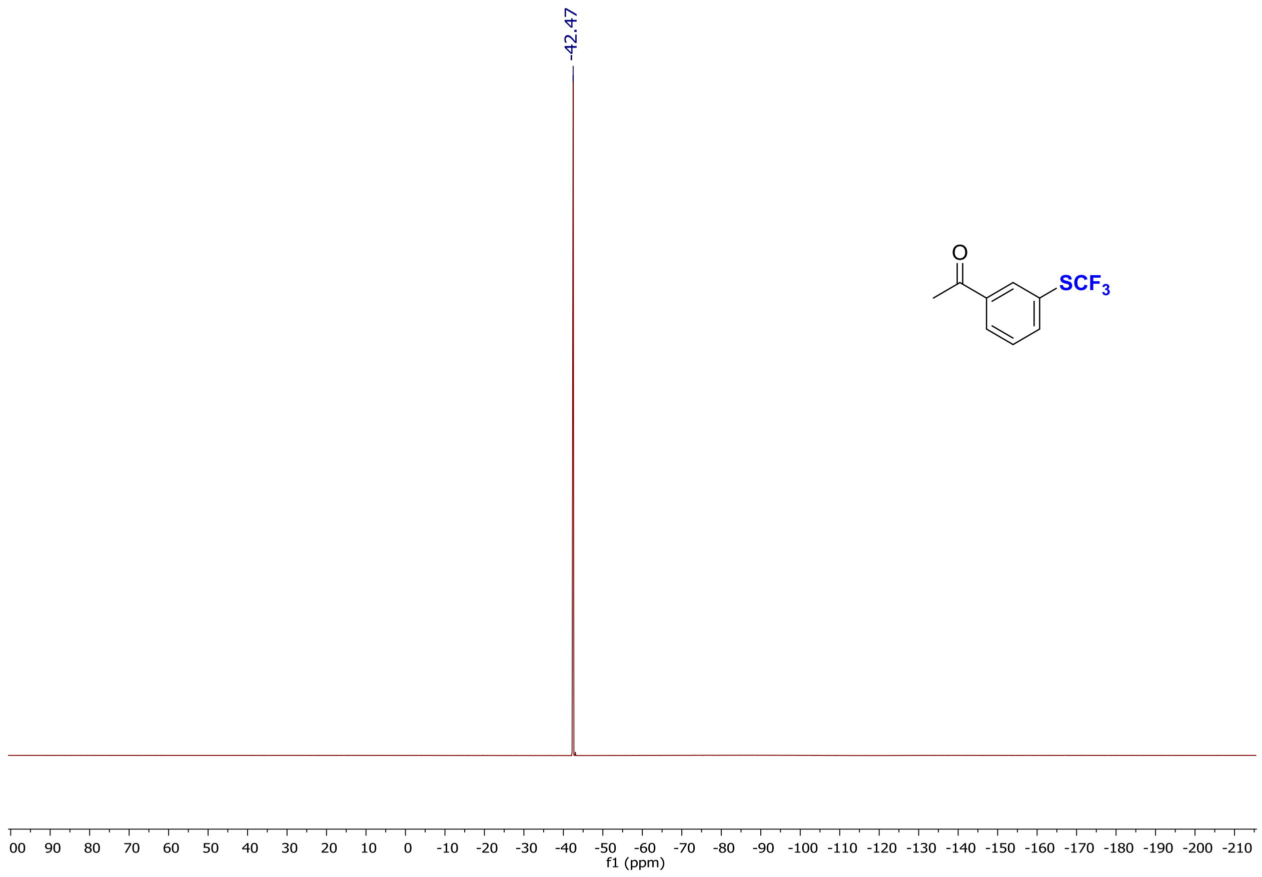
-42.47
SCF3
O
00 90 80 70 60 50 40 30 20 10 0 -10 -20 -30 -40 -50 -60 -70 -80 -90 -100 -110 -120 -130 -140 -150 -160 -170 -180 -190 -200 -210
f1 (ppm)

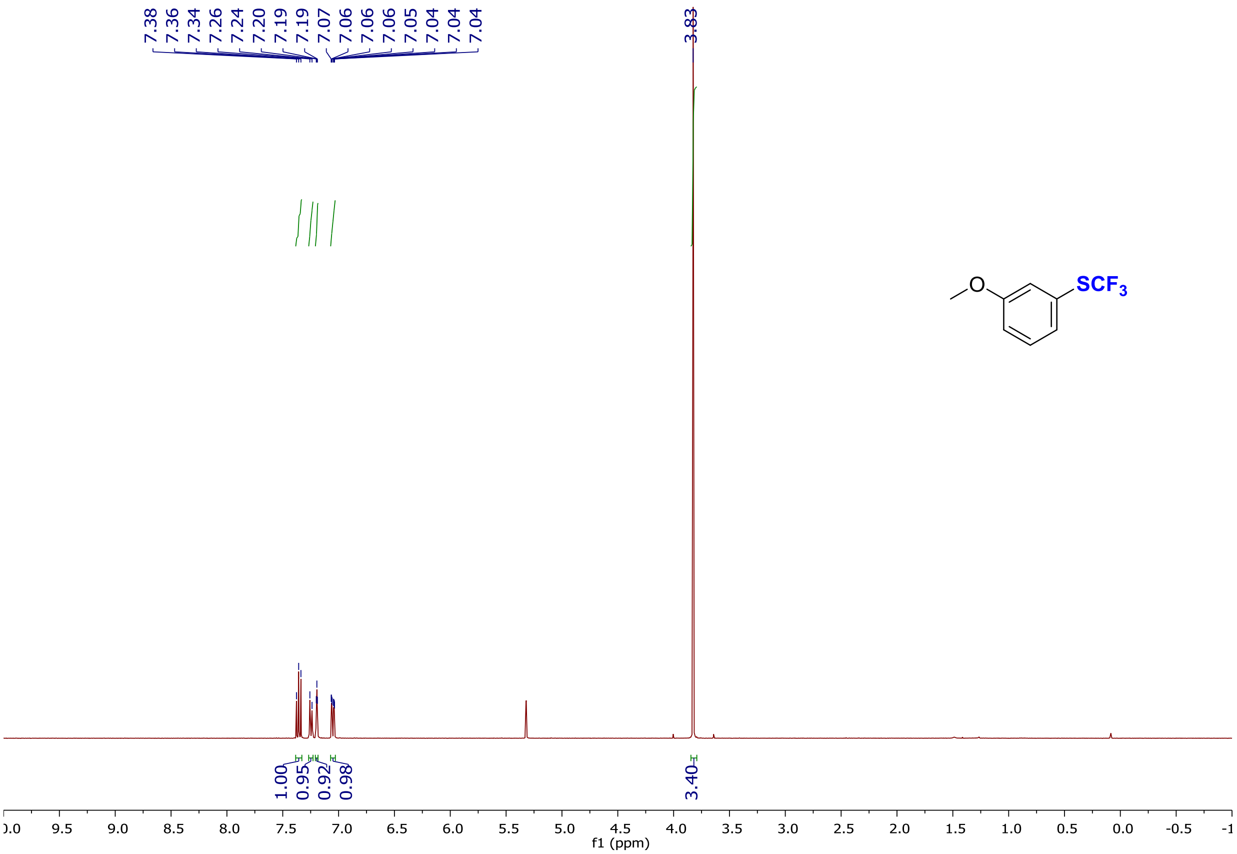
7.38 7.36 7.34 7.26 7.24 7.20 7.19 7.19 7.07 7.06 7.06 7.06 7.05 7.04 7.04 7.04
3.83
SCF3
O
1.00 0.95 0.92 0.98
3.40
f1 (ppm)

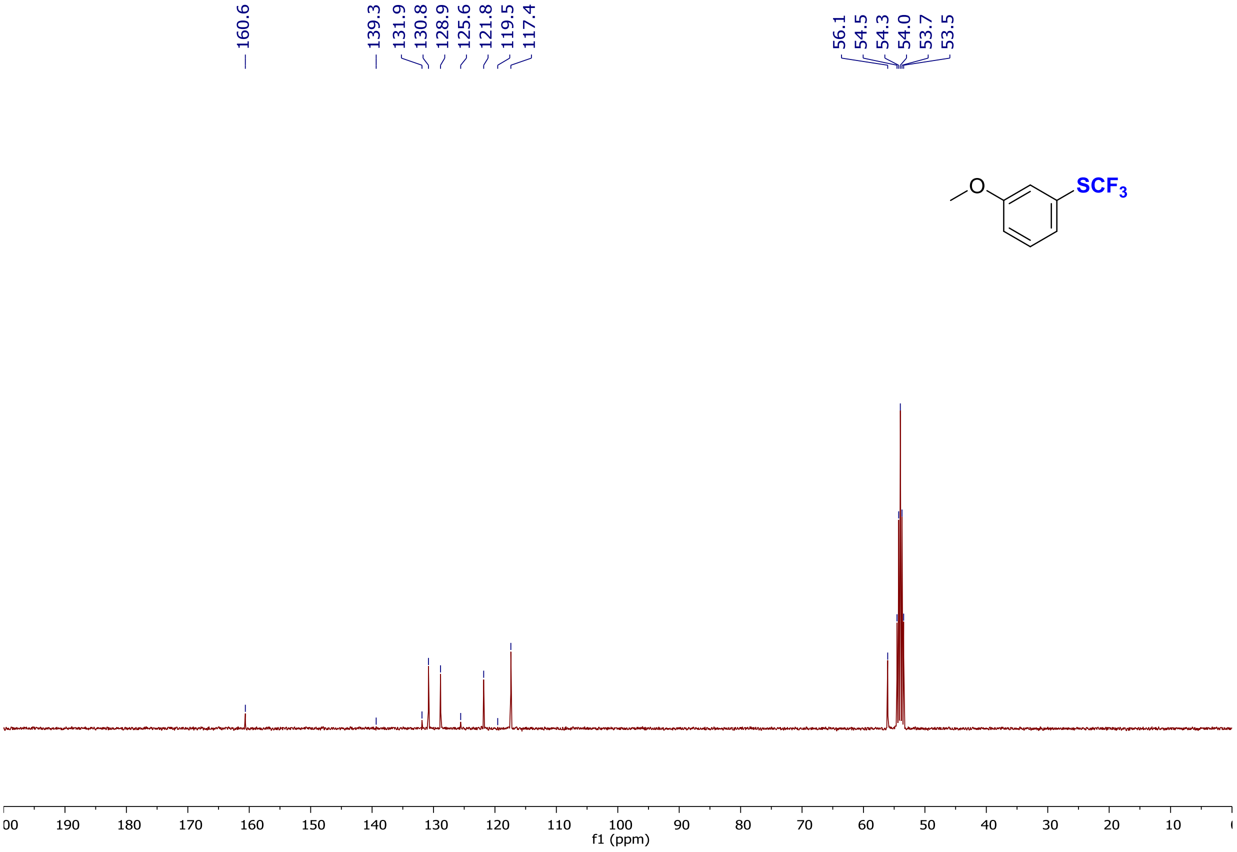
160.6
139.3 131.9 130.8 128.9 125.6 121.8 119.5 117.4
56.1 54.5 54.3 54.0 53.7 53.5
SCF3
O
f1 (ppm)

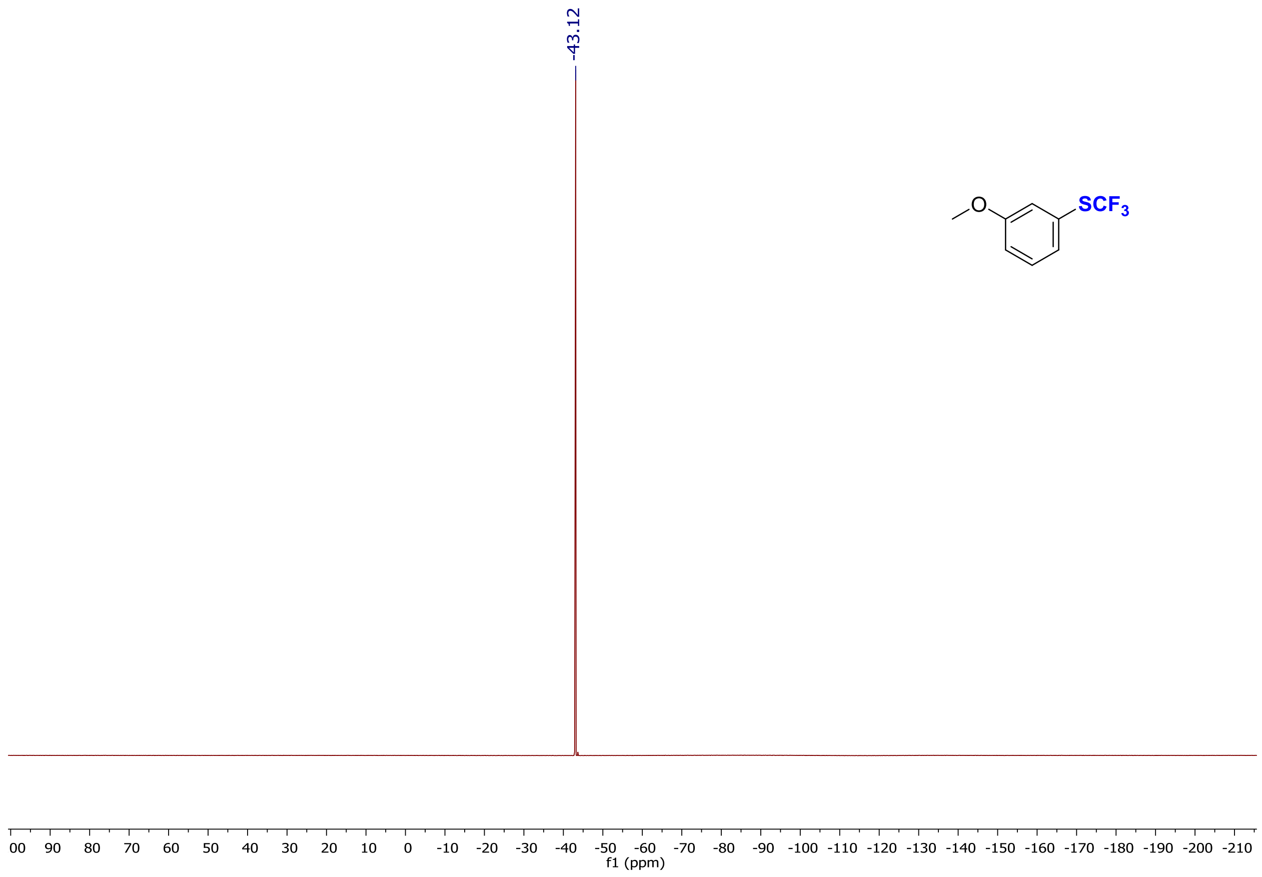
-43.12
SCF3
O
00 90 80 70 60 50 40 30 20 10 0 -10 -20 -30 -40 -50 -60 -70 -80 -90 -100 -110 -120 -130 -140 -150 -160 -170 -180 -190 -200 -210
f1 (ppm)

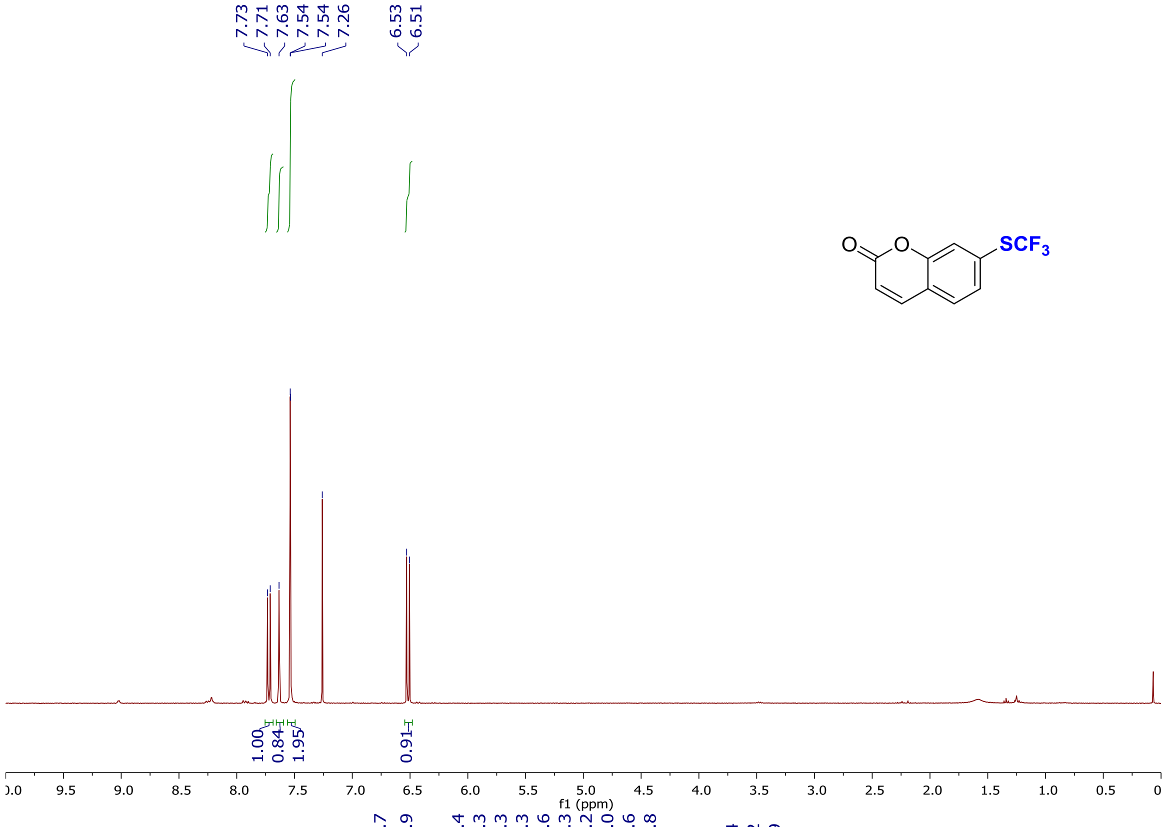
7.73
7.71
7.63
7.54
7.54
7.26
6.53
6.51
O
O
SCF$_3$
1.00
0.84
1.95
0.91
f1 (ppm)

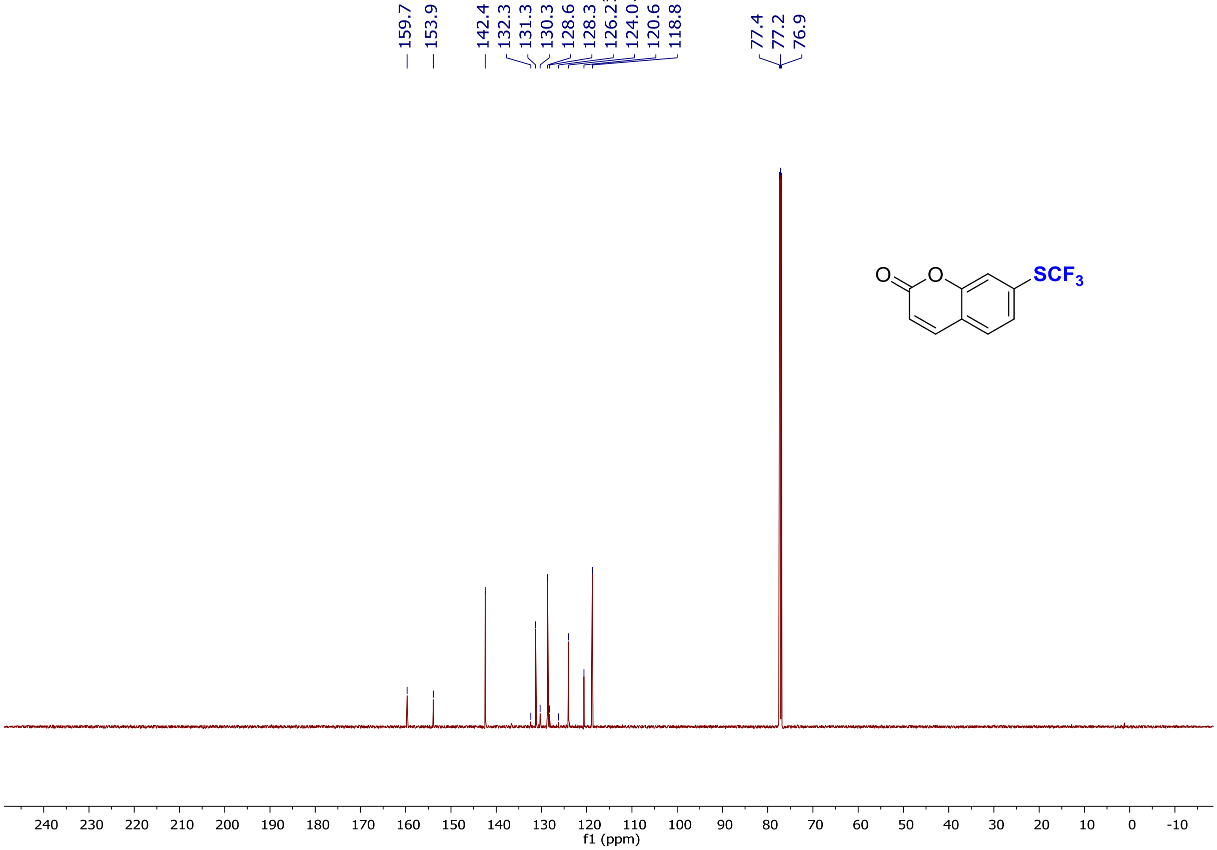
159.7
153.9
142.4
132.3
131.3
130.3
128.6
128.3
126.2
124.0
120.6
118.8
77.4
77.2
76.9
O
O
SCF$_3$
f1 (ppm)

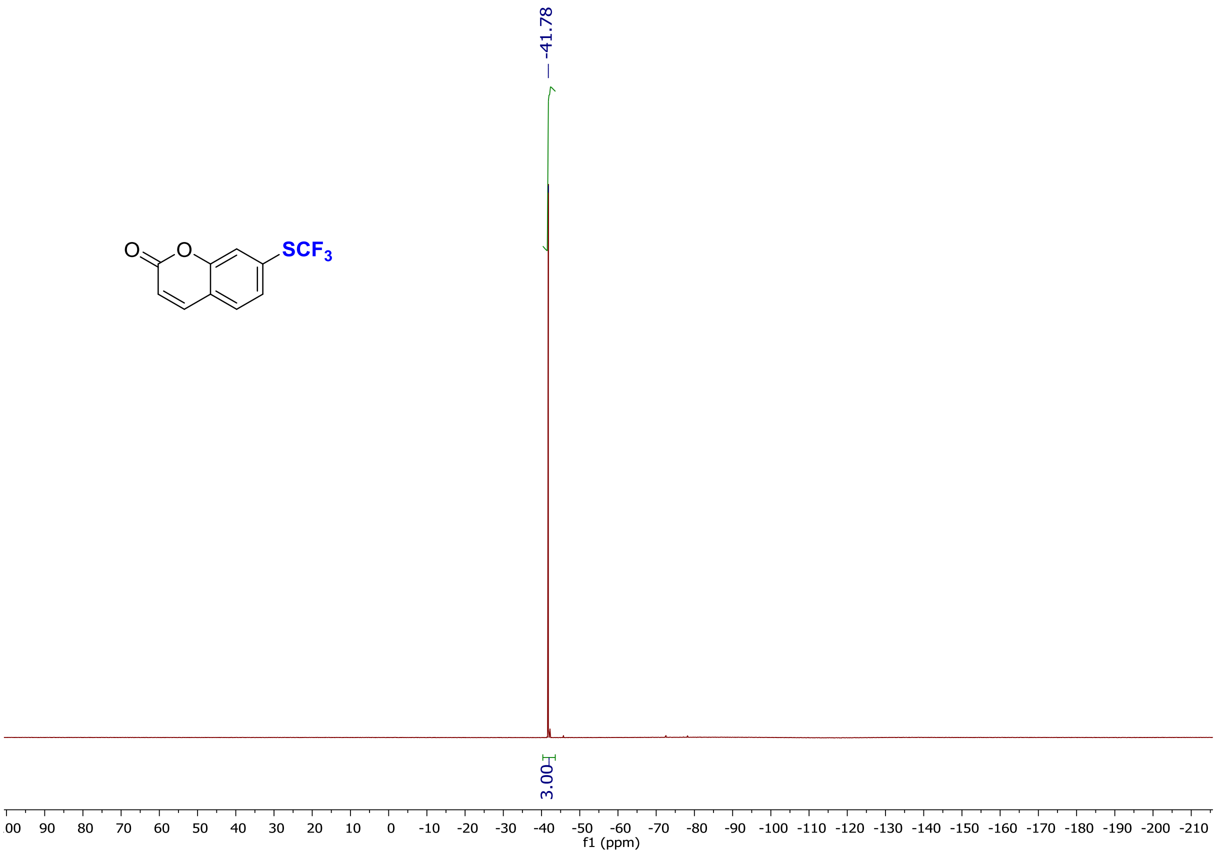
-41.78
SCF3
O
3.00
00 90 80 70 60 50 40 30 20 10 0 -10 -20 -30 -40 -50 -60 -70 -80 -90 -100 -110 -120 -130 -140 -150 -160 -170 -180 -190 -200 -210
f1 (ppm)

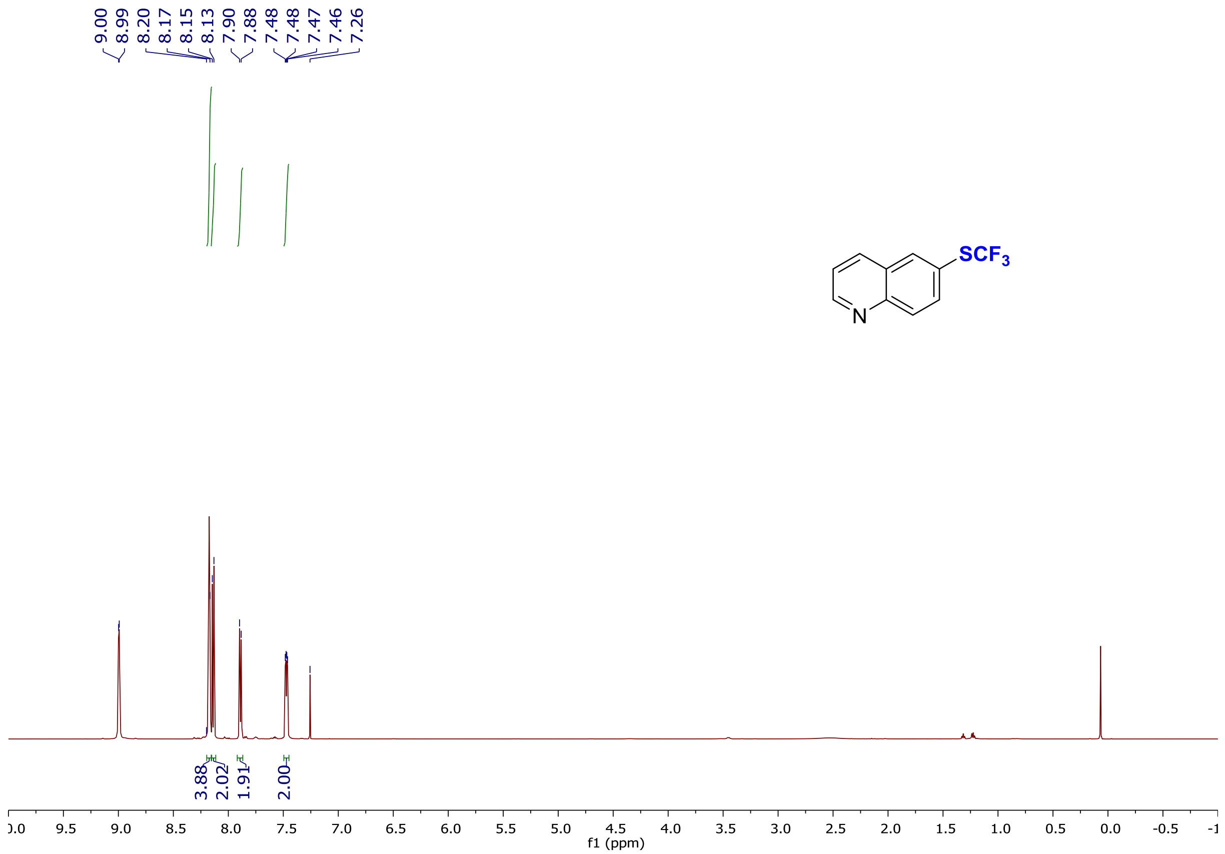
9.00
8.99
8.20
8.17
8.15
8.13
7.90
7.88
7.48
7.48
7.47
7.46
7.26
SCF3
N
3.88
2.02
1.91
2.00
f1 (ppm)

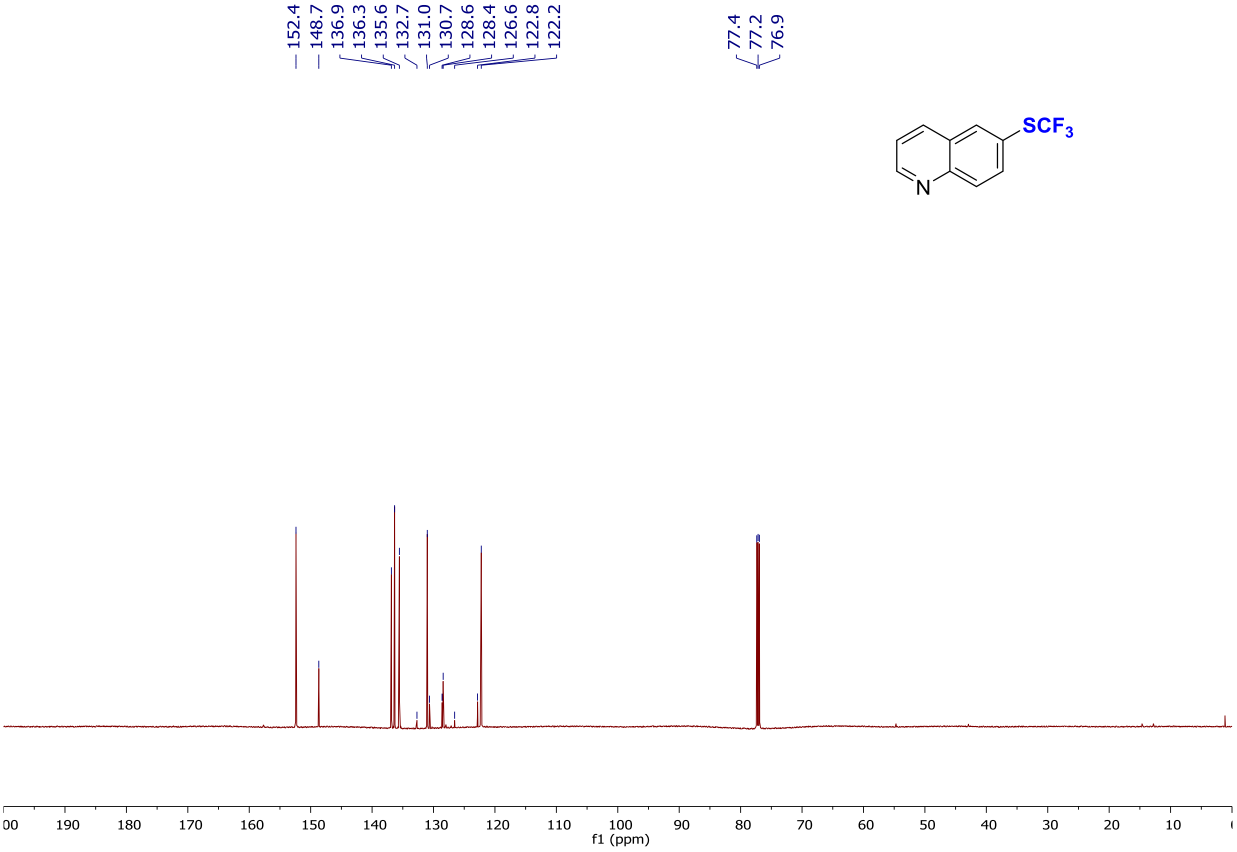
152.4
148.7
136.9
136.3
135.6
132.7
131.0
130.7
128.6
128.4
126.6
122.8
122.2
77.4
77.2
76.9
SCF3
N
f1 (ppm)

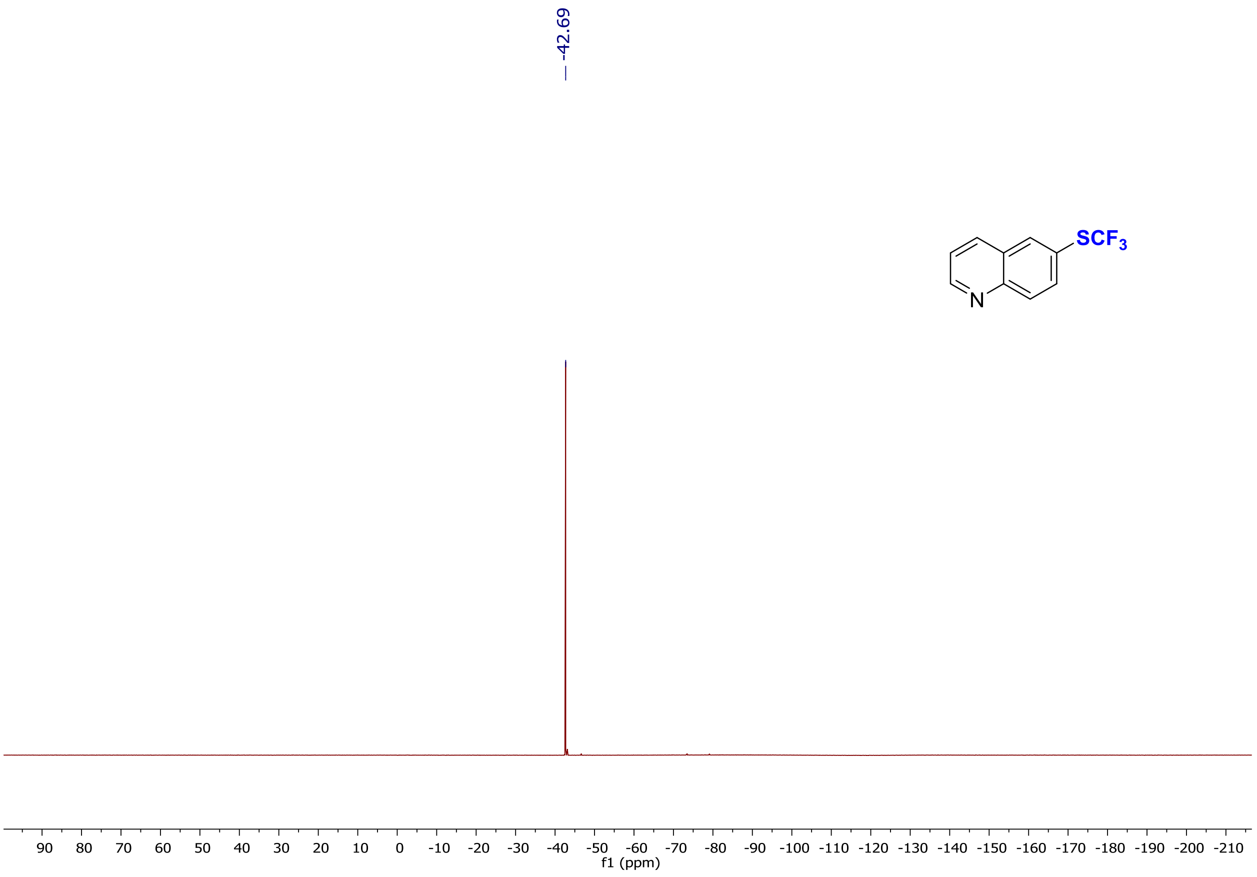

-42.69
SCF3
N
90 80 70 60 50 40 30 20 10 0 -10 -20 -30 -40 -50 -60 -70 -80 -90 -100 -110 -120 -130 -140 -150 -160 -170 -180 -190 -200 -210
f1 (ppm)

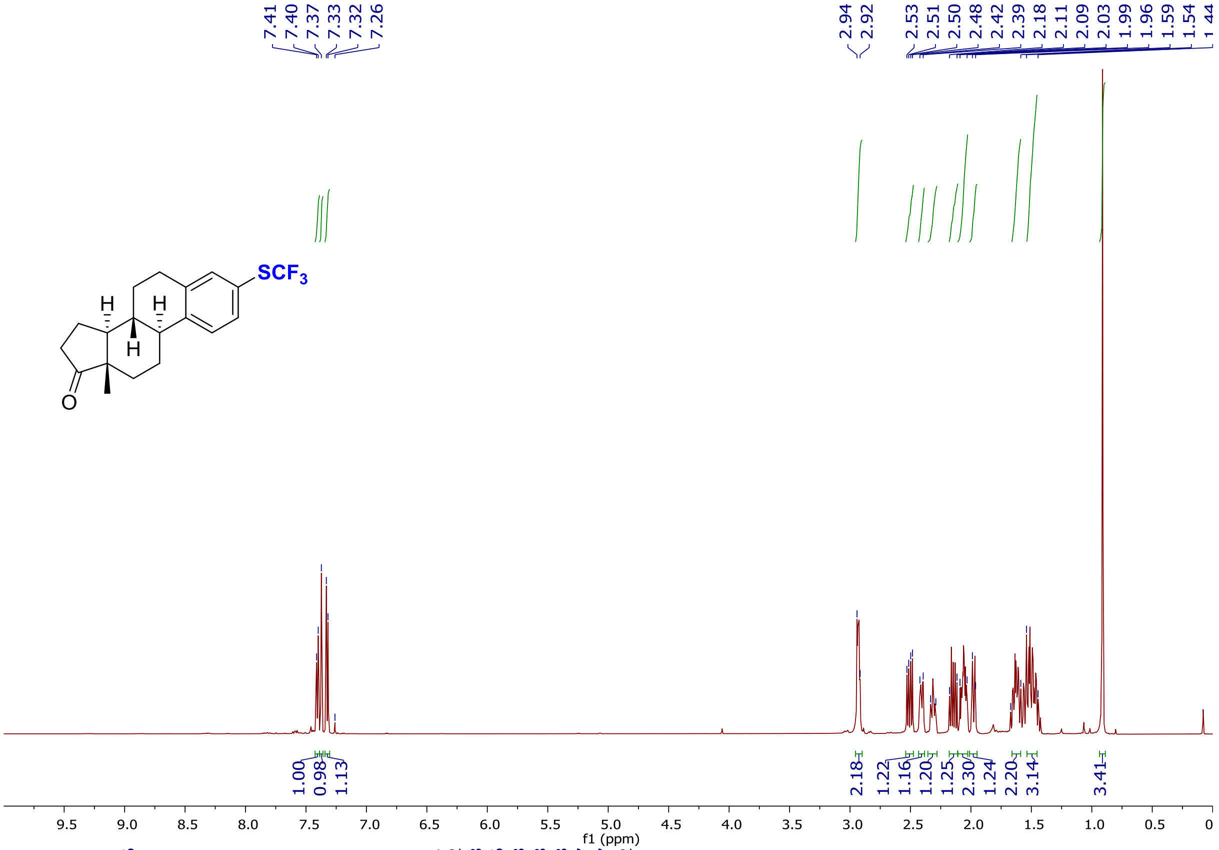
SCF3
H
H
H
O
7.41
7.40
7.37
7.33
7.32
7.26
2.94
2.92
2.53
2.51
2.50
2.48
2.42
2.39
2.18
2.11
2.09
2.03
1.99
1.96
1.59
1.54
1.44
1.00
0.98
1.13
2.18
1.22
1.16
1.20
1.25
2.30
1.24
2.20
3.14
3.41
f1 (ppm)

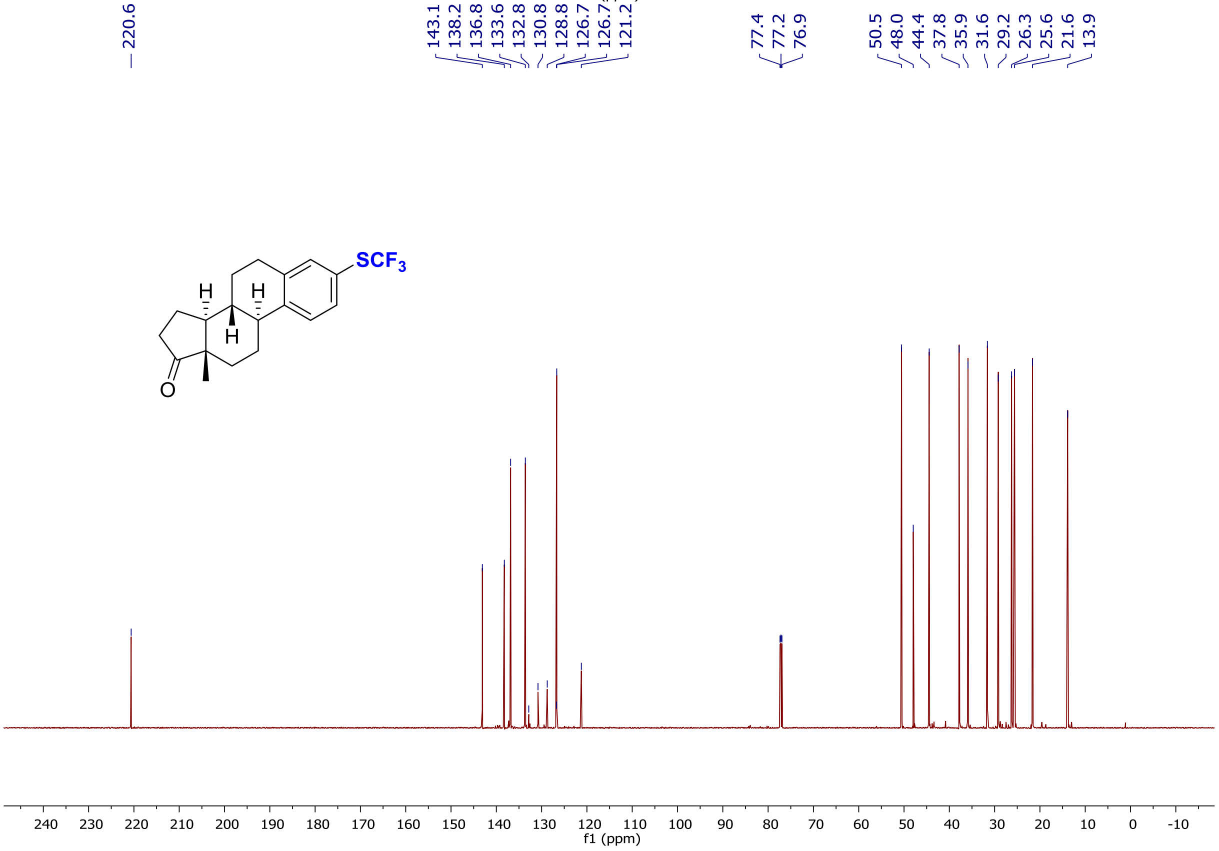
220.6
143.1
138.2
136.8
133.6
132.8
130.8
128.8
126.7
126.7
121.2
77.4
77.2
76.9
50.5
48.0
44.4
37.8
35.9
31.6
29.2
26.3
25.6
21.6
13.9
SCF3
H
H
H
O
f1 (ppm)

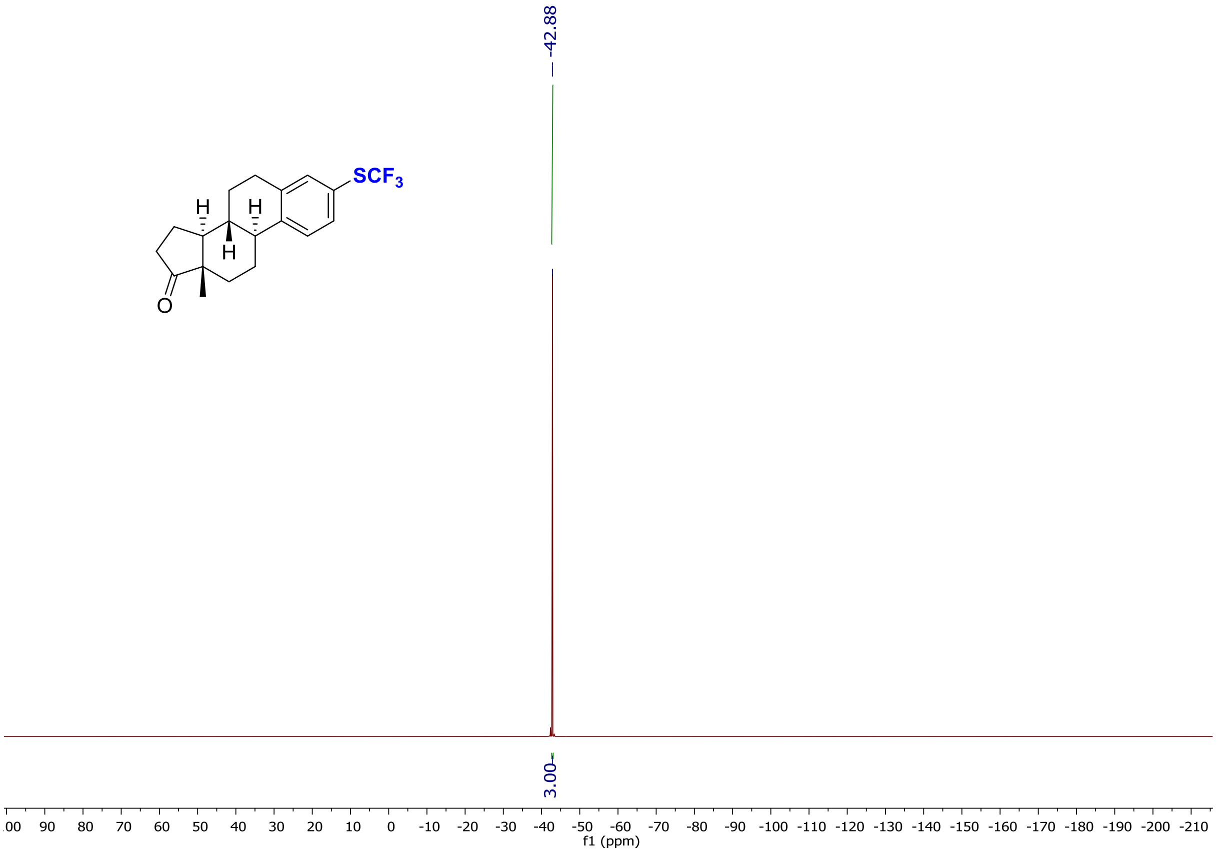

SCF3
H
H
H
O
-42.88
3.00
00 90 80 70 60 50 40 30 20 10 0 -10 -20 -30 -40 -50 -60 -70 -80 -90 -100 -110 -120 -130 -140 -150 -160 -170 -180 -190 -200 -210
f1 (ppm)

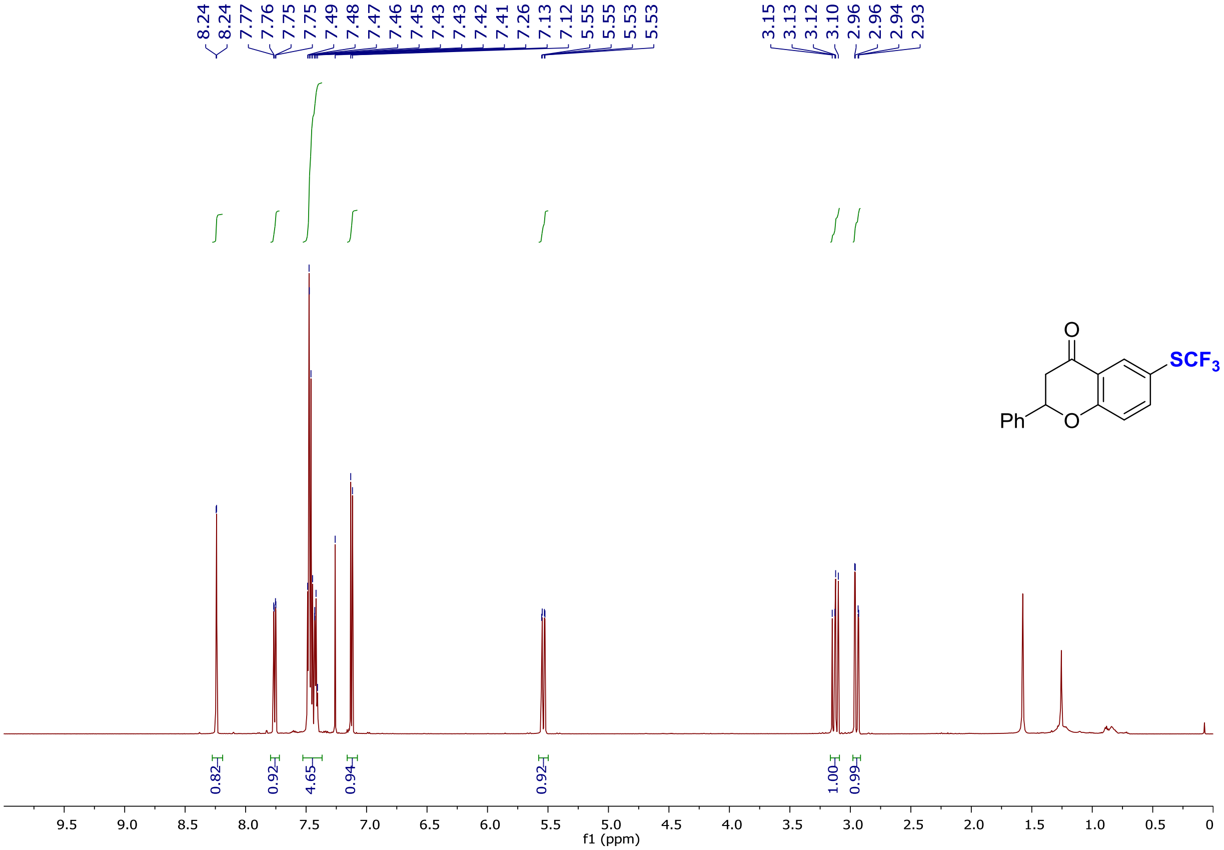
8.24
8.24
7.77
7.76
7.75
7.75
7.49
7.48
7.47
7.46
7.45
7.43
7.43
7.42
7.41
7.26
7.13
7.12
5.55
5.55
5.53
5.53
3.15
3.13
3.12
3.10
2.96
2.96
2.94
2.93
O
SCF3
Ph
O
0.82
0.92
4.65
0.94
0.92
1.00
0.99
9.5
9.0
8.5
8.0
7.5
7.0
6.5
6.0
5.5
5.0
4.5
4.0
3.5
3.0
2.5
2.0
1.5
1.0
0.5
0
f1 (ppm)

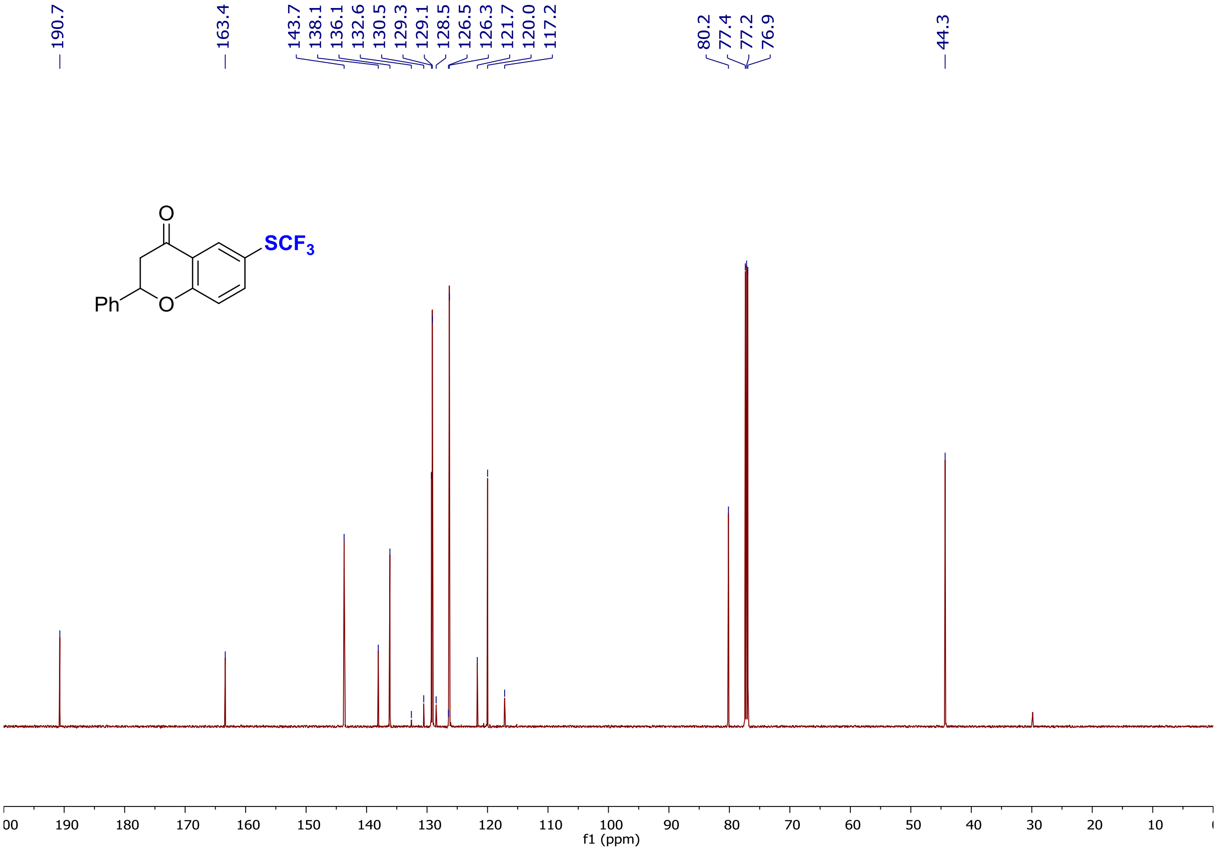
190.7
163.4
143.7
138.1
136.1
132.6
130.5
129.3
129.1
128.5
126.5
126.3
121.7
120.0
117.2
80.2
77.4
77.2
76.9
44.3
O
SCF3
Ph
O
190
180
170
160
150
140
130
120
110
100
90
80
70
60
50
40
30
20
10
f1 (ppm)

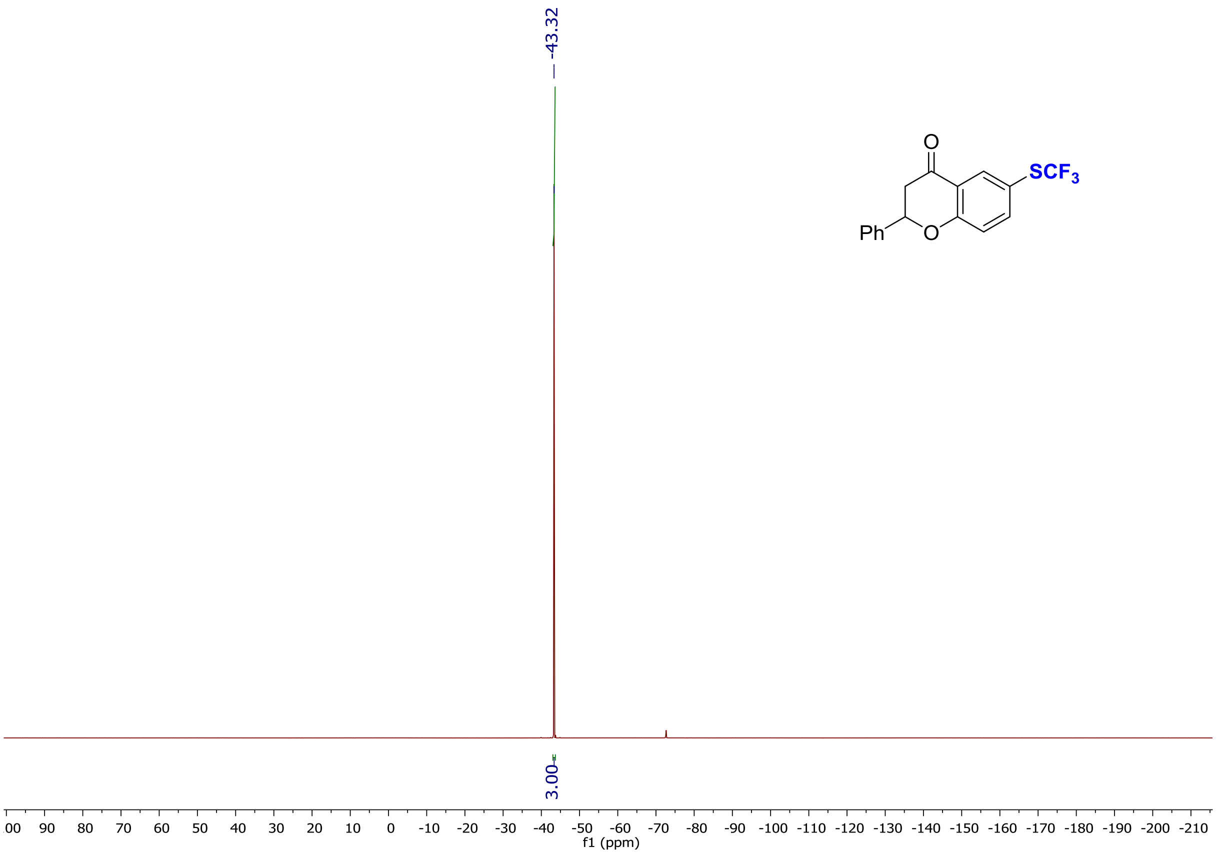

-43.32
O
SCF_3
Ph
O
3.00
00 90 80 70 60 50 40 30 20 10 0 -10 -20 -30 -40 -50 -60 -70 -80 -90 -100 -110 -120 -130 -140 -150 -160 -170 -180 -190 -200 -210
f1 (ppm)

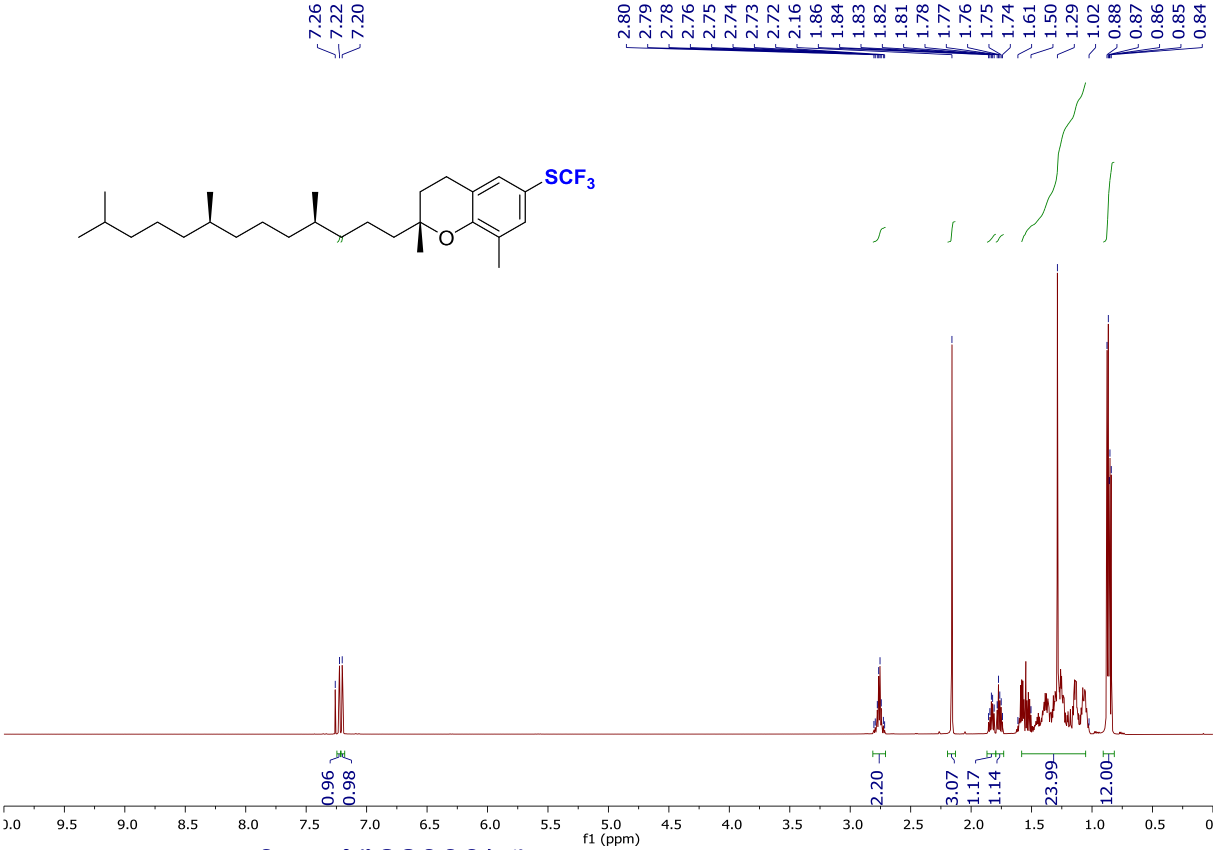
7.26
7.22
7.20
2.80
2.79
2.78
2.76
2.75
2.74
2.73
2.72
2.16
1.86
1.84
1.83
1.82
1.81
1.78
1.77
1.76
1.75
1.74
1.61
1.50
1.29
1.02
0.88
0.87
0.86
0.85
0.84
SCF3
O
0.96
0.98
2.20
3.07
1.17
1.14
23.99
12.00
f1 (ppm)

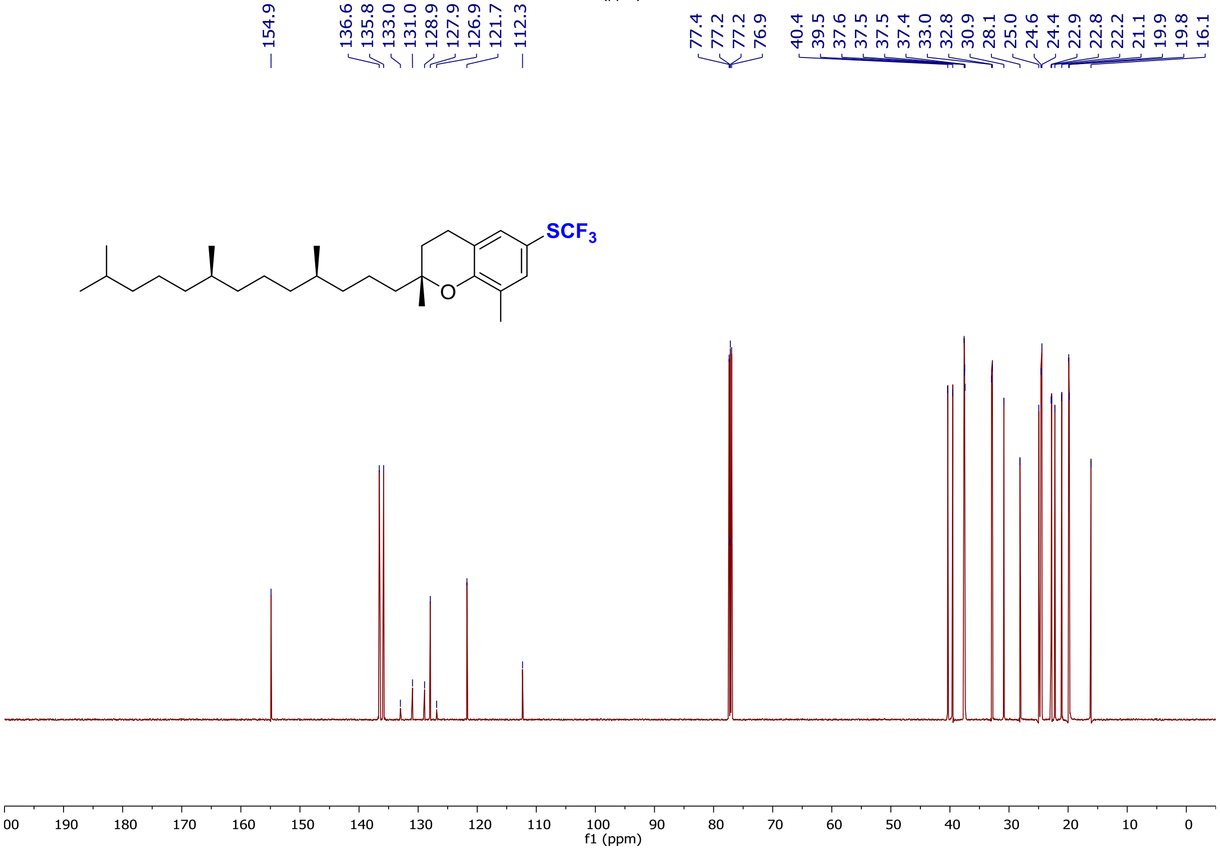
154.9
136.6
135.8
133.0
131.0
128.9
127.9
126.9
121.7
112.3
77.4
77.2
77.2
76.9
40.4
39.5
37.6
37.5
37.4
33.0
32.8
30.9
28.1
25.0
24.6
24.4
22.9
22.8
22.2
21.1
19.9
19.8
16.1
SCF3
O
f1 (ppm)

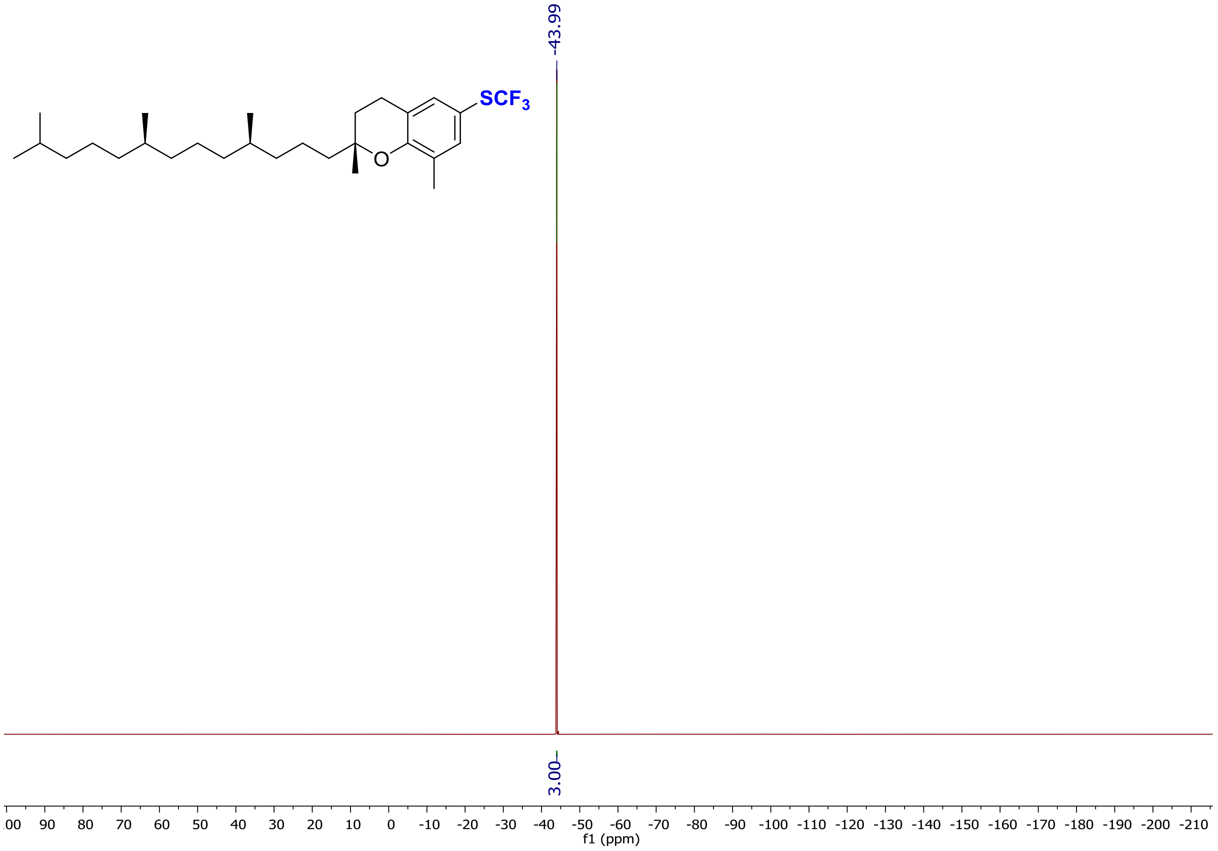

SCF3
O
-43.99
3.00
00
90
80
70
60
50
40
30
20
10
0
-10
-20
-30
-40
-50
-60
-70
-80
-90
-100
-110
-120
-130
-140
-150
-160
-170
-180
-190
-200
-210
f1 (ppm)

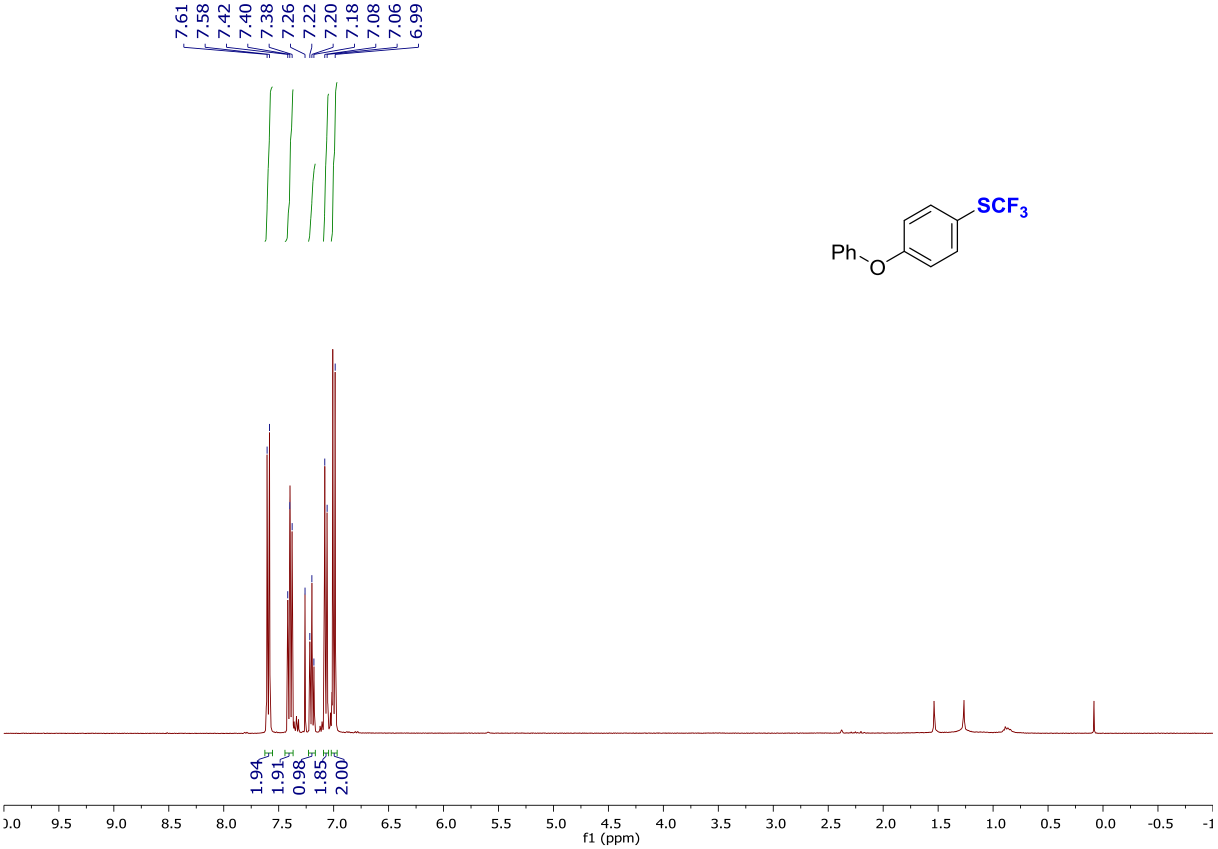
7.61
7.58
7.42
7.40
7.38
7.26
7.22
7.20
7.18
7.08
7.06
6.99
SCF3
Ph
O
1.94
1.91
0.98
1.85
2.00
f1 (ppm)

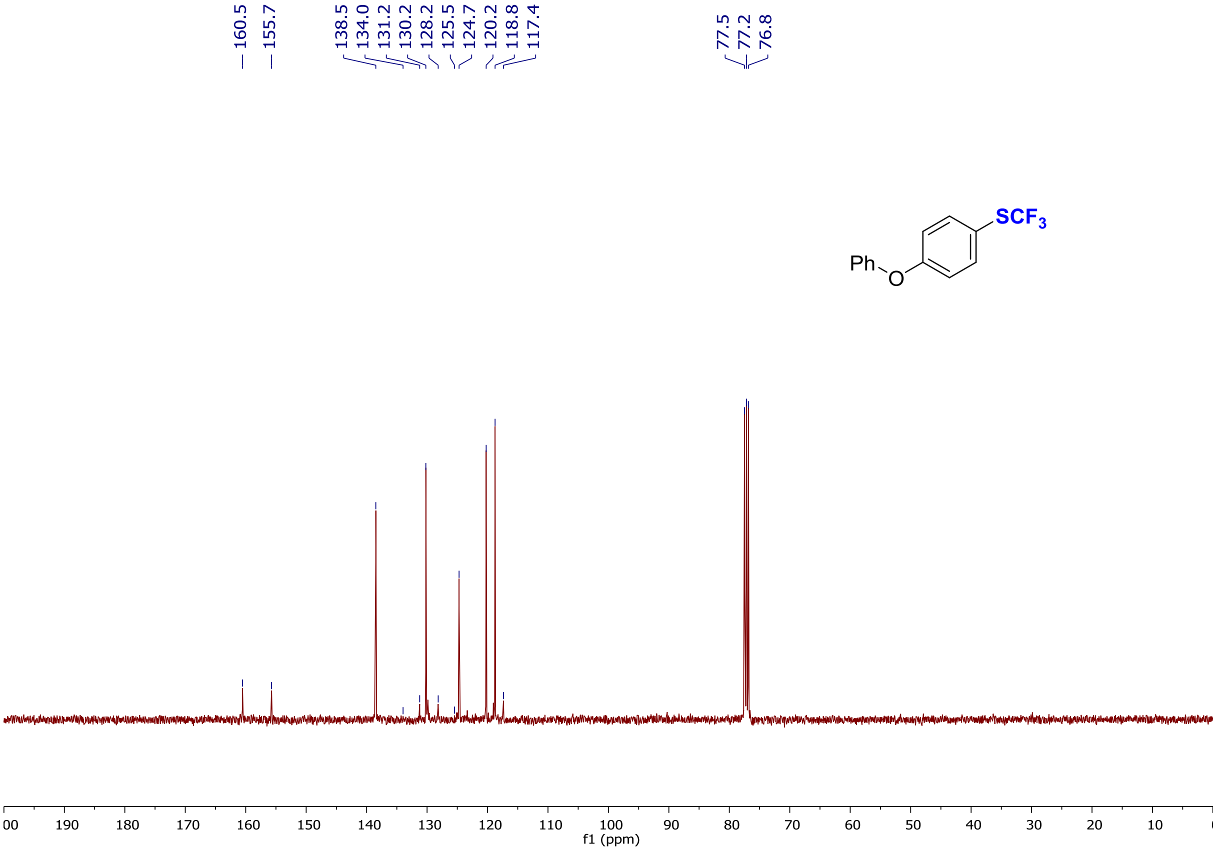
160.5
155.7
138.5
134.0
131.2
130.2
128.2
125.5
124.7
120.2
118.8
117.4
77.5
77.2
76.8
SCF3
Ph
O
f1 (ppm)

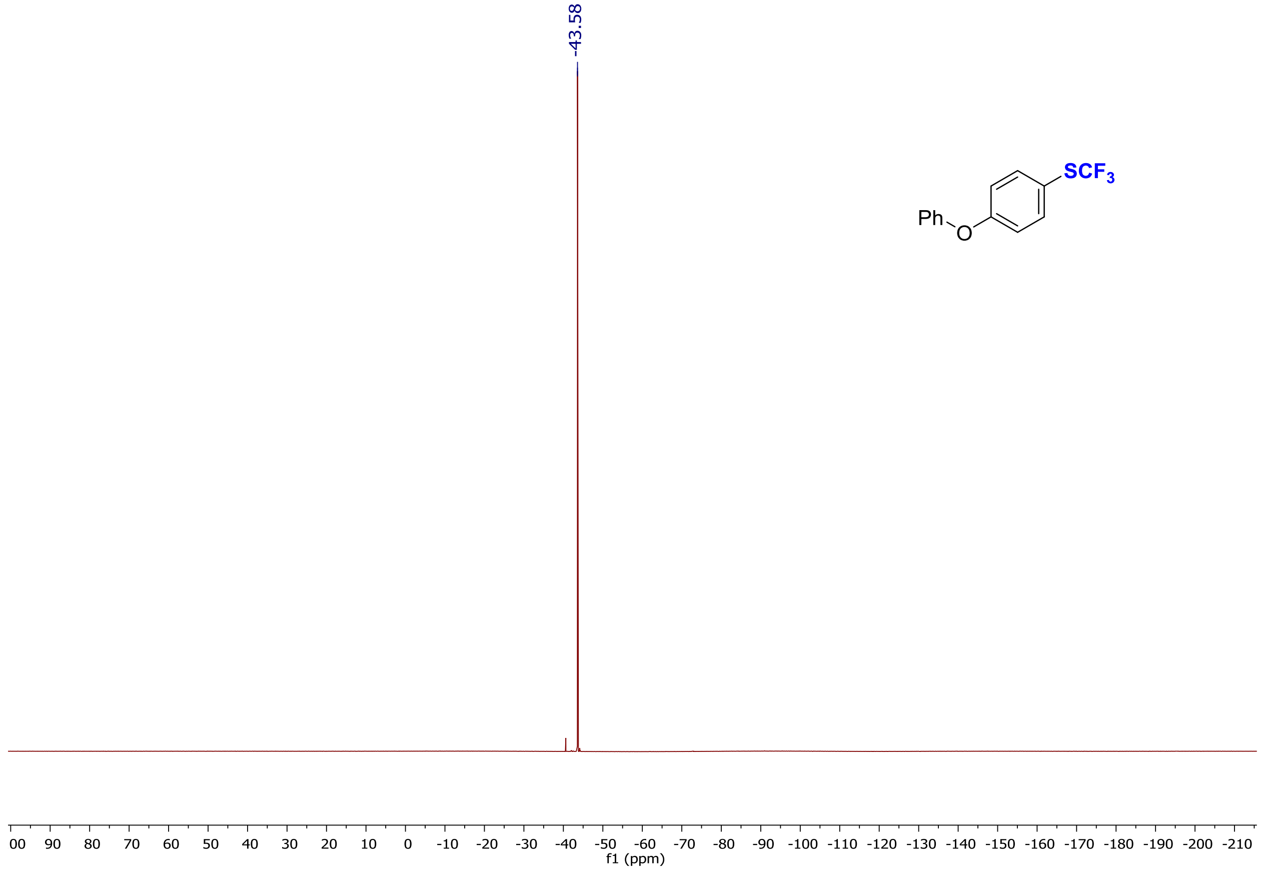
-43.58
SCF3
Ph
O
00 90 80 70 60 50 40 30 20 10 0 -10 -20 -30 -40 -50 -60 -70 -80 -90 -100 -110 -120 -130 -140 -150 -160 -170 -180 -190 -200 -210
f1 (ppm)

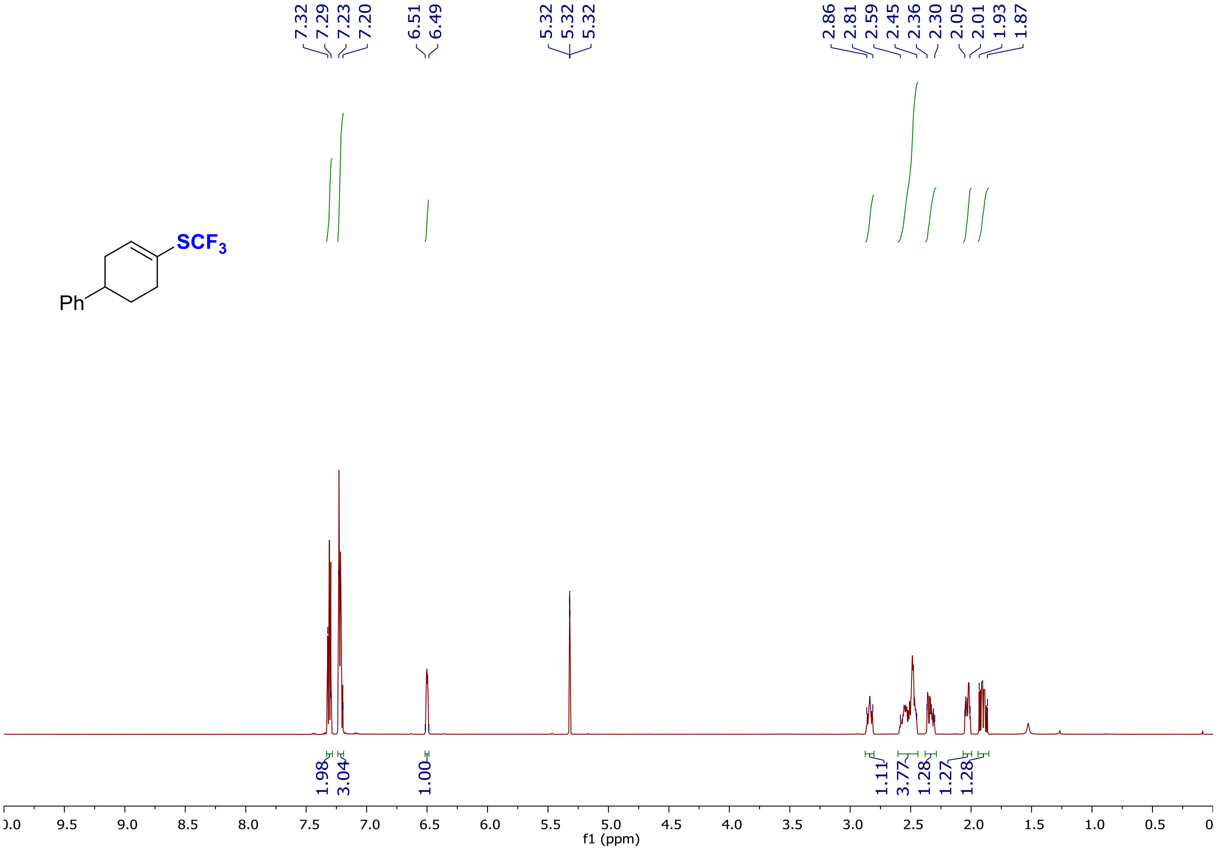
7.32
7.29
7.23
7.20
6.51
6.49
5.32
5.32
5.32
2.86
2.81
2.59
2.45
2.36
2.30
2.05
2.01
1.93
1.87
SCF3
Ph
1.98
3.04
1.00
1.11
3.77
1.28
1.27
1.28
f1 (ppm)

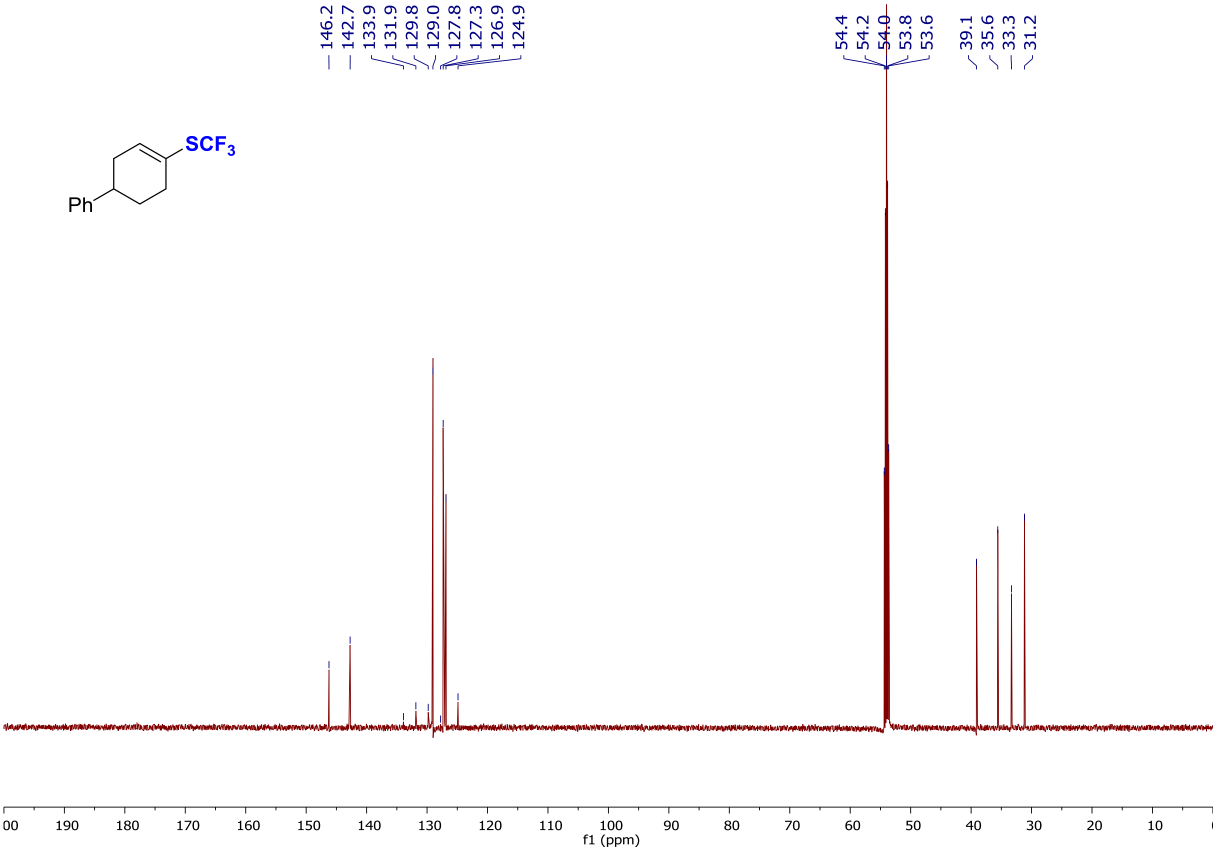
146.2
142.7
133.9
131.9
129.8
129.0
127.8
127.3
126.9
124.9
54.4
54.2
54.0
53.8
53.6
39.1
35.6
33.3
31.2
SCF3
Ph
f1 (ppm)

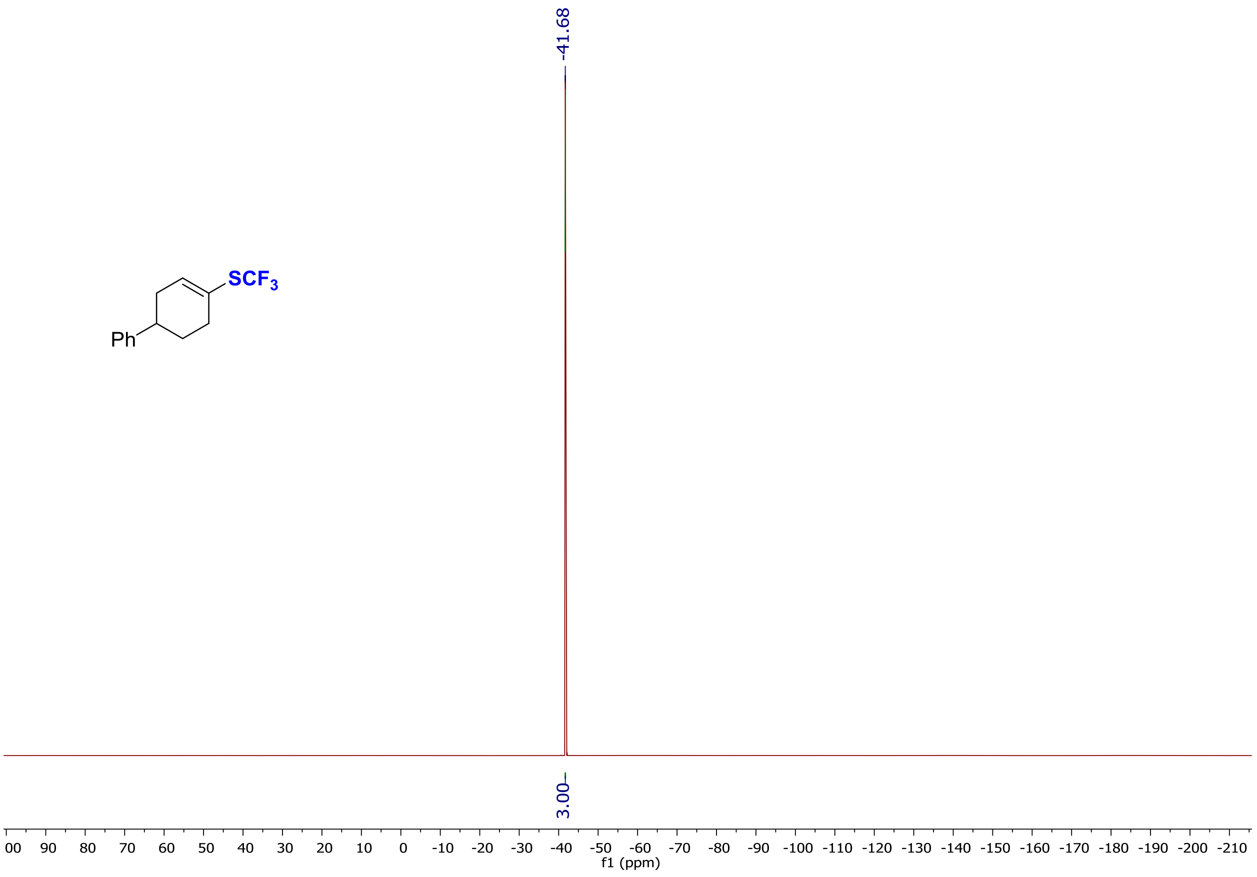

SCF3
Ph
-41.68
3.00
00 90 80 70 60 50 40 30 20 10 0 -10 -20 -30 -40 -50 -60 -70 -80 -90 -100 -110 -120 -130 -140 -150 -160 -170 -180 -190 -200 -210
f1 (ppm)

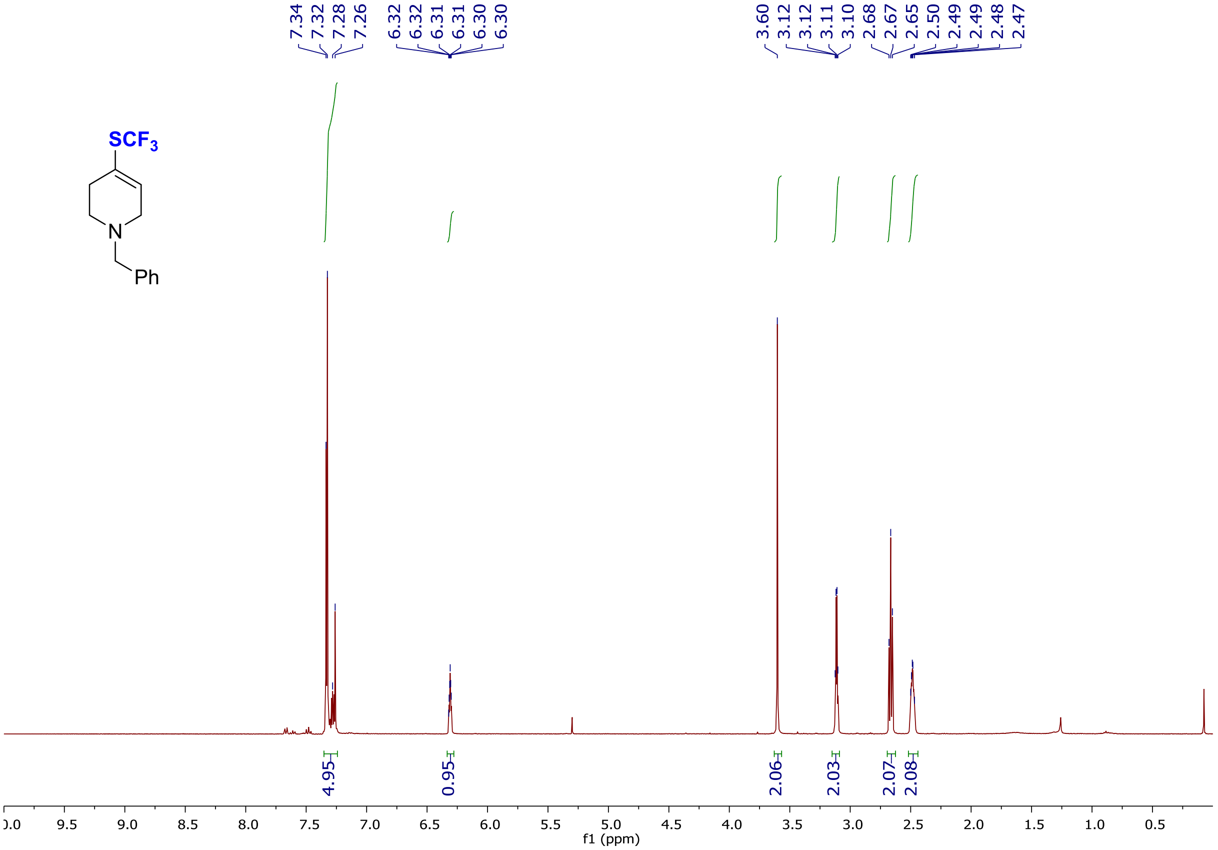

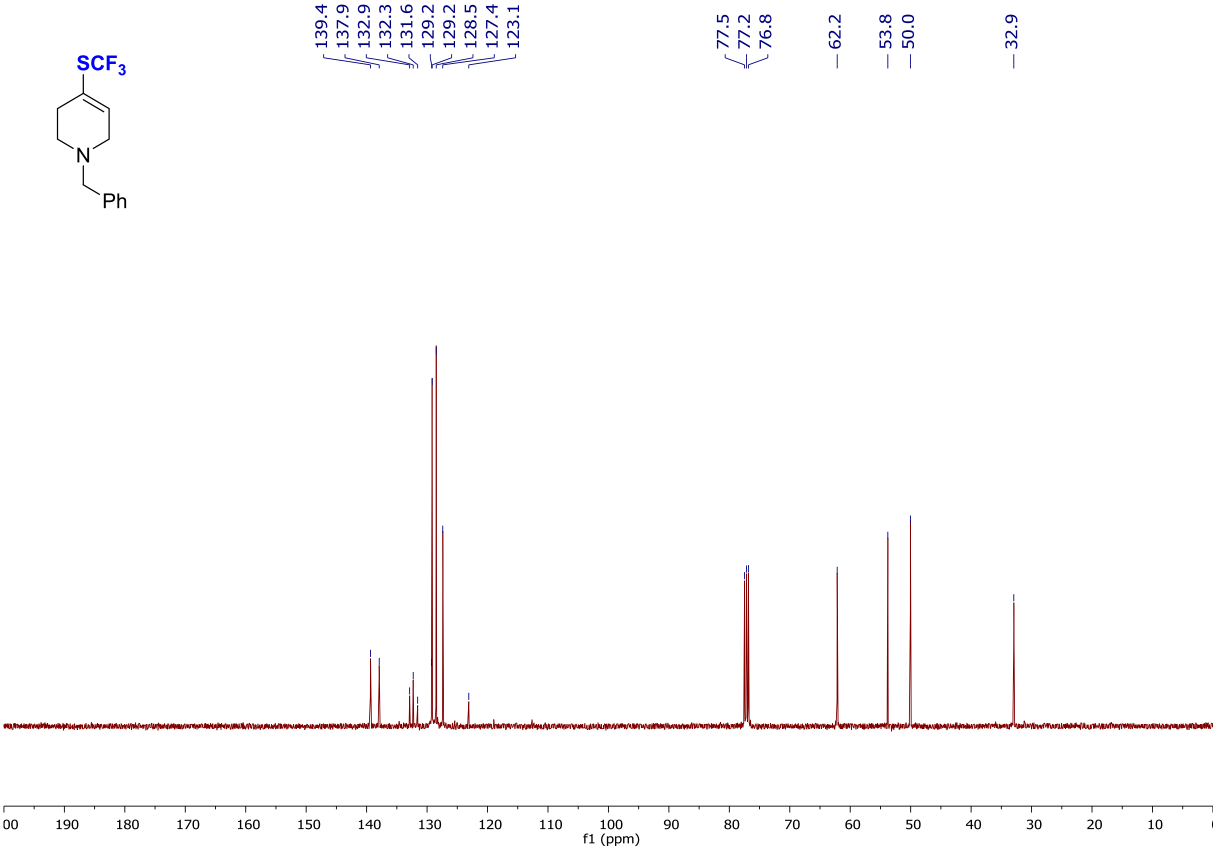

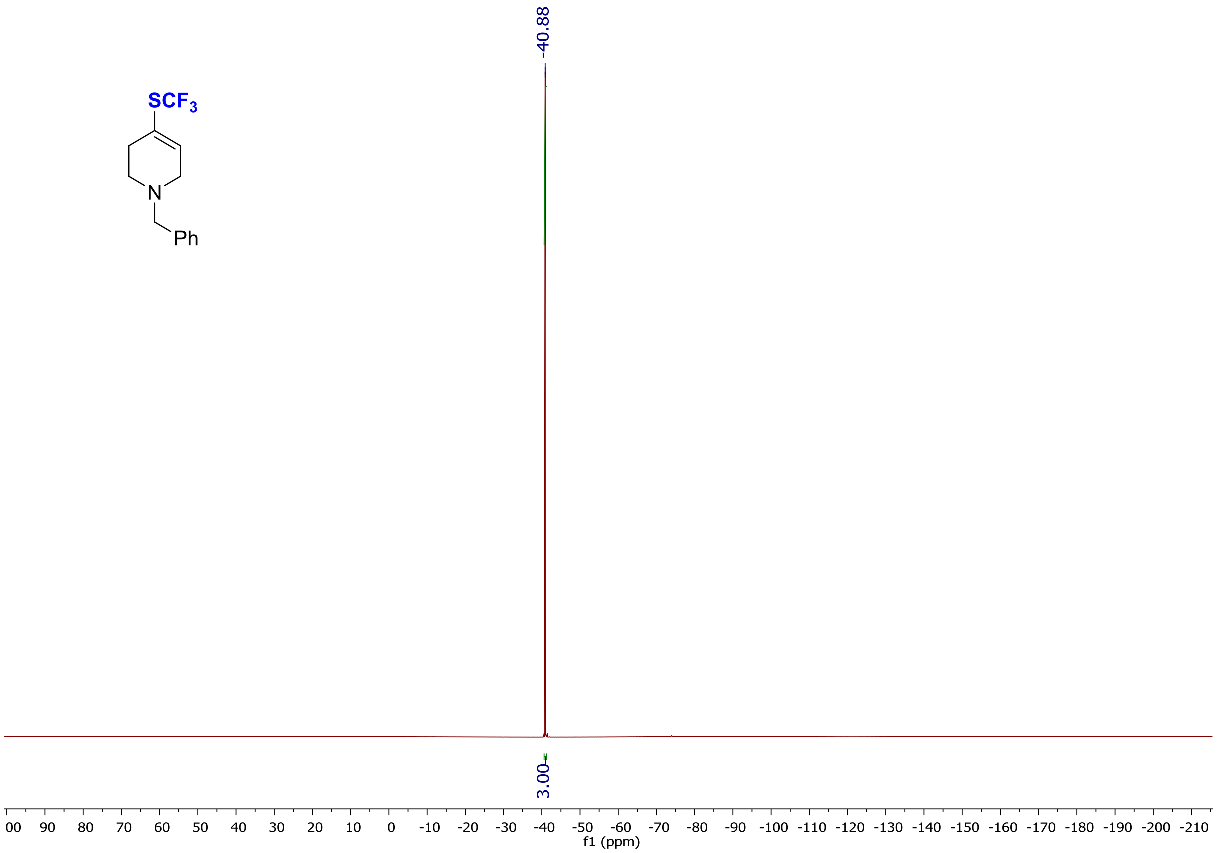
-40.88
SCF3
N
Ph
3.00
00 90 80 70 60 50 40 30 20 10 0 -10 -20 -30 -40 -50 -60 -70 -80 -90 -100 -110 -120 -130 -140 -150 -160 -170 -180 -190 -200 -210
f1 (ppm)

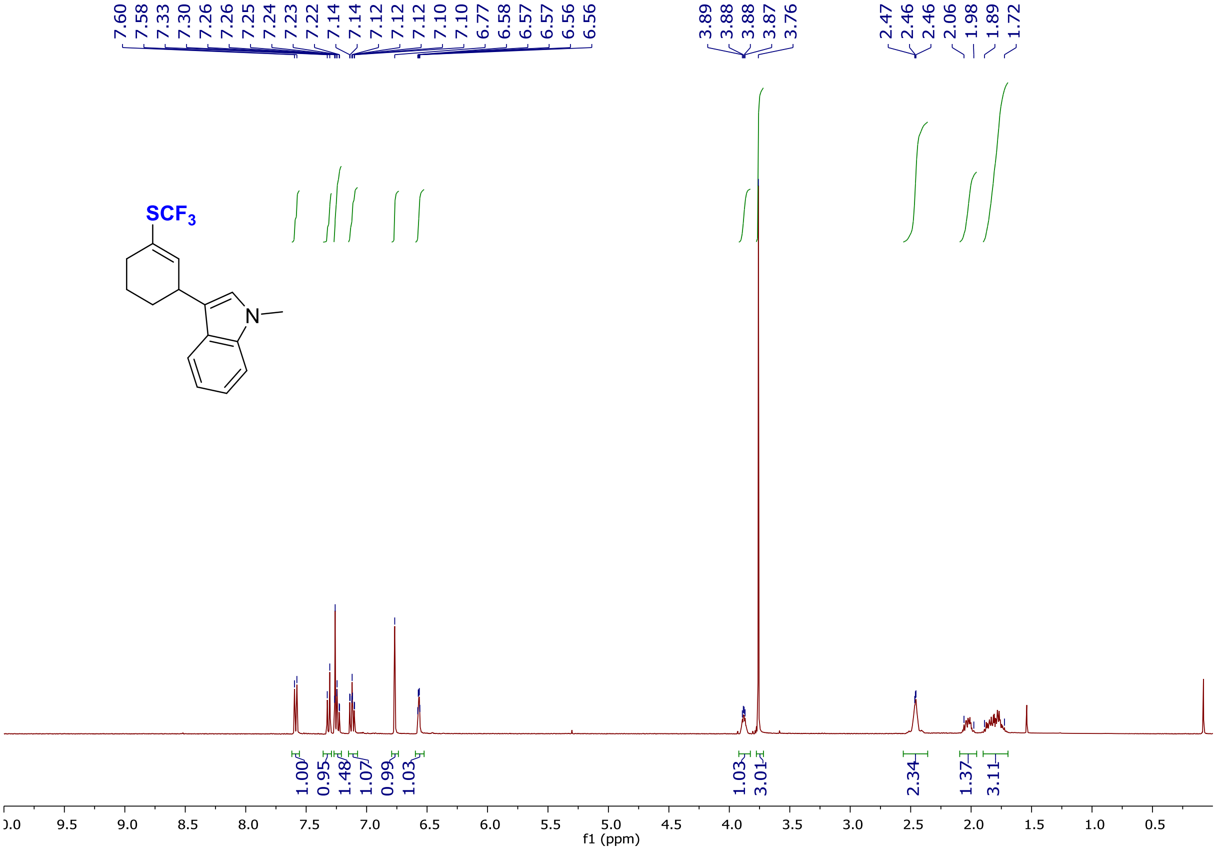
7.60 7.58 7.33 7.30 7.26 7.26 7.25 7.24 7.23 7.22 7.14 7.14 7.12 7.12 7.10 7.10 6.77 6.58 6.57 6.57 6.56 6.56
3.89 3.88 3.88 3.87 3.76
2.47 2.46 2.46 2.06 1.98 1.89 1.72
SCF3
N
1.00 0.95 1.48 1.07 0.99 1.03 1.03 3.01 2.34 1.37 3.11
f1 (ppm)

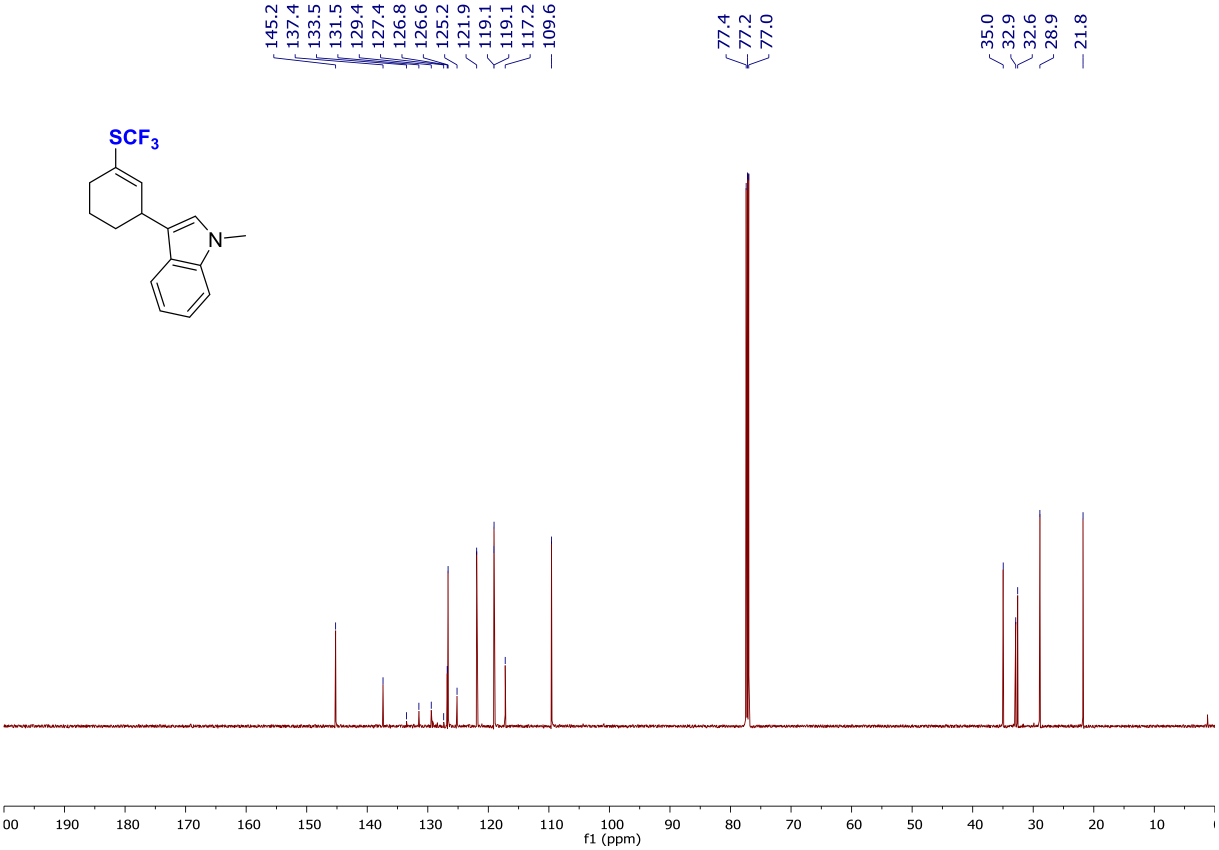
145.2 137.4 133.5 131.5 129.4 127.4 126.8 126.6 125.2 121.9 119.1 119.1 117.2 109.6
77.4 77.2 77.0
35.0 32.9 32.6 28.9 21.8
SCF3
N
f1 (ppm)

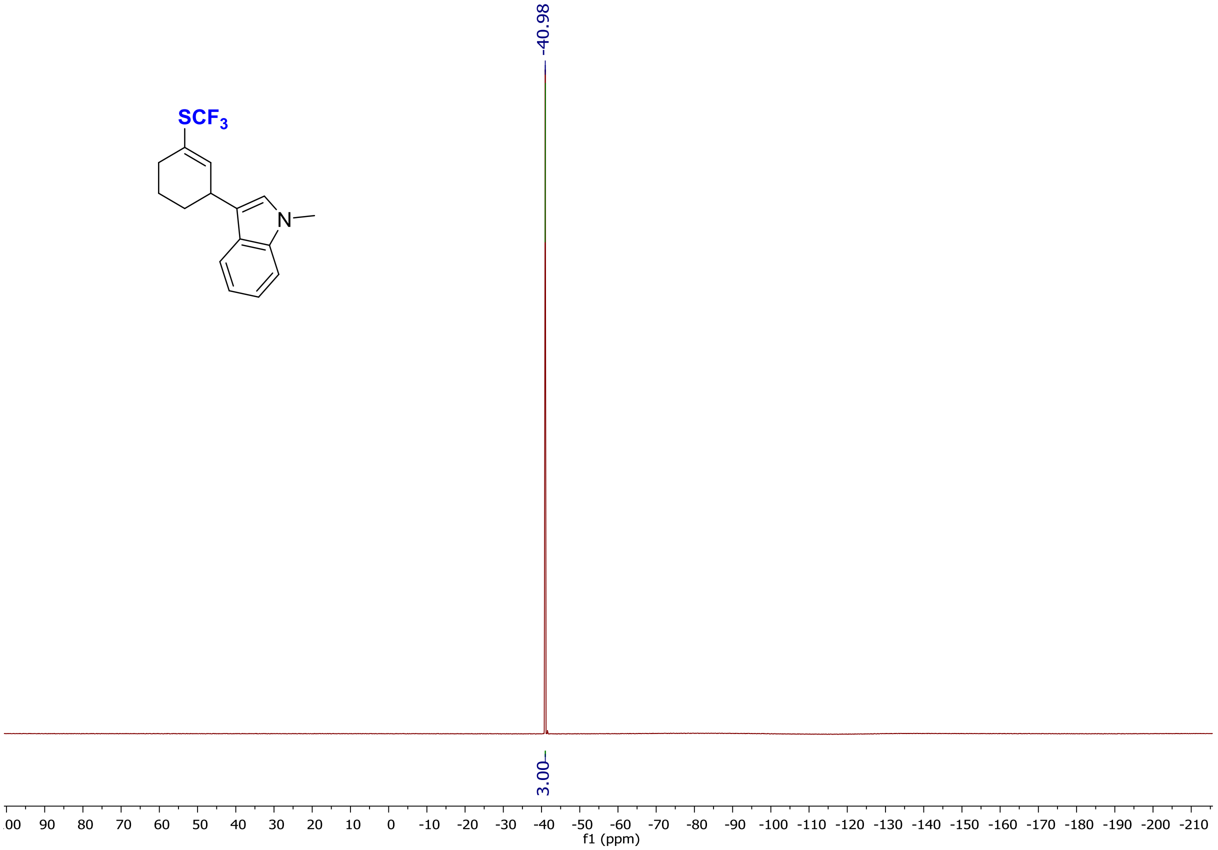
-40.98
SCF3
N
3.00
00 90 80 70 60 50 40 30 20 10 0 -10 -20 -30 -40 -50 -60 -70 -80 -90 -100 -110 -120 -130 -140 -150 -160 -170 -180 -190 -200 -210
f1 (ppm)

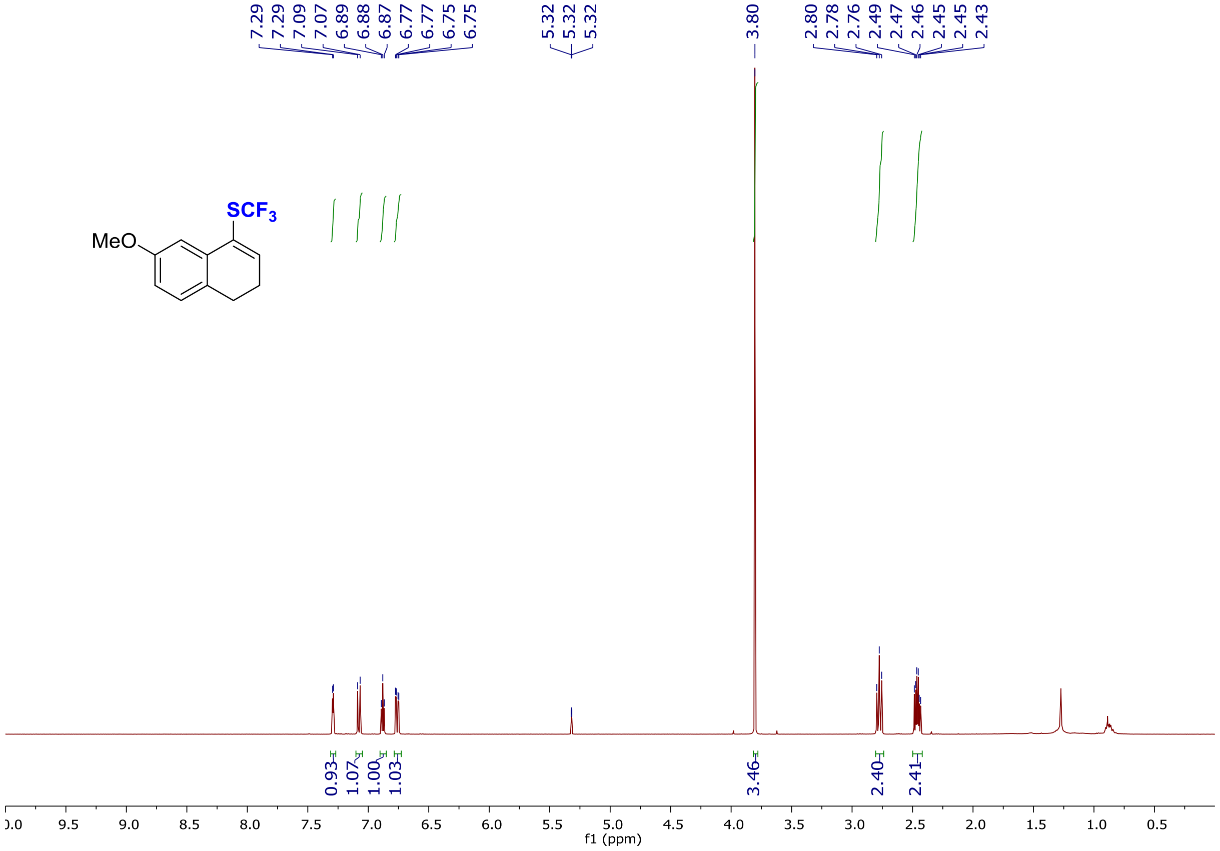
SCF3
MeO
7.29
7.29
7.09
7.07
6.89
6.88
6.87
6.77
6.77
6.75
6.75
5.32
5.32
5.32
3.80
2.80
2.78
2.76
2.49
2.47
2.46
2.45
2.45
2.43
0.93
1.07
1.00
1.03
3.46
2.40
2.41
f1 (ppm)

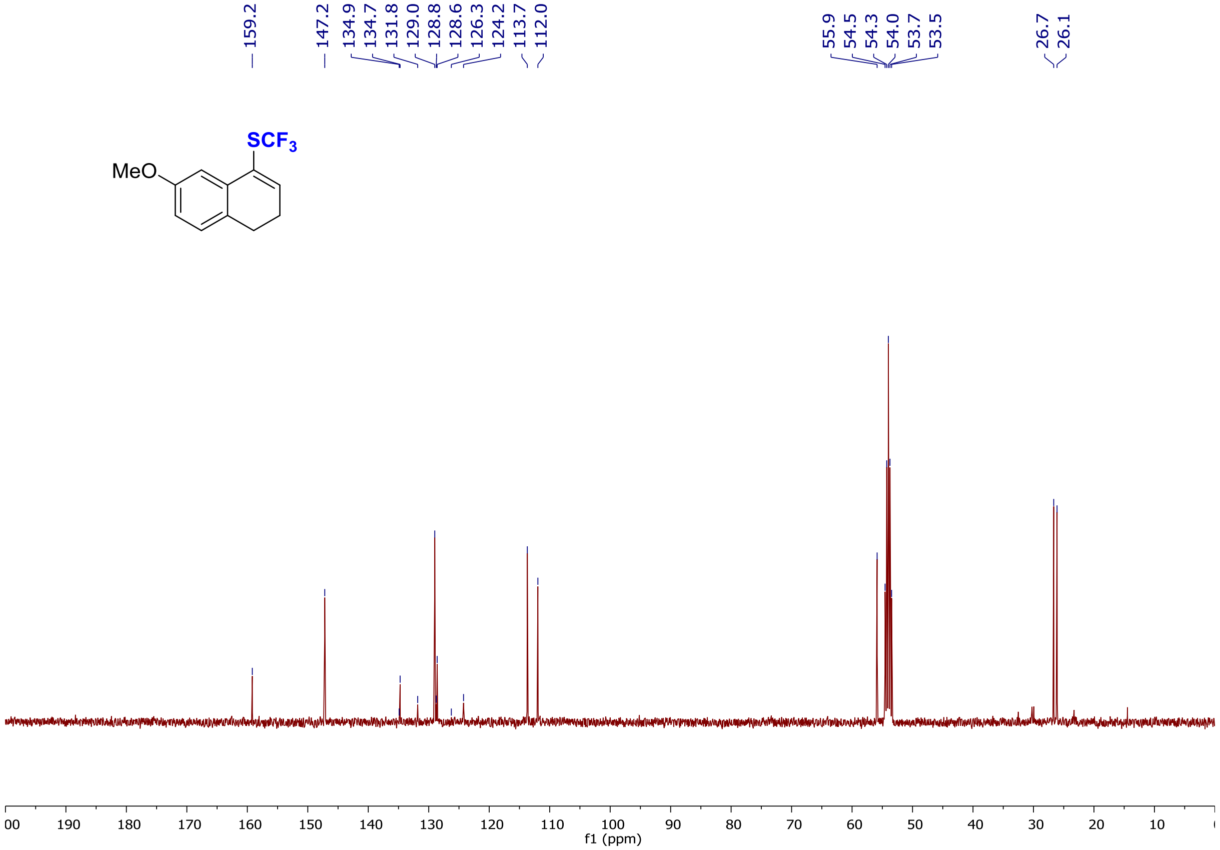
159.2
147.2
134.9
134.7
131.8
129.0
128.8
128.6
126.3
124.2
113.7
112.0
55.9
54.5
54.3
54.0
53.7
53.5
26.7
26.1
SCF3
MeO
f1 (ppm)

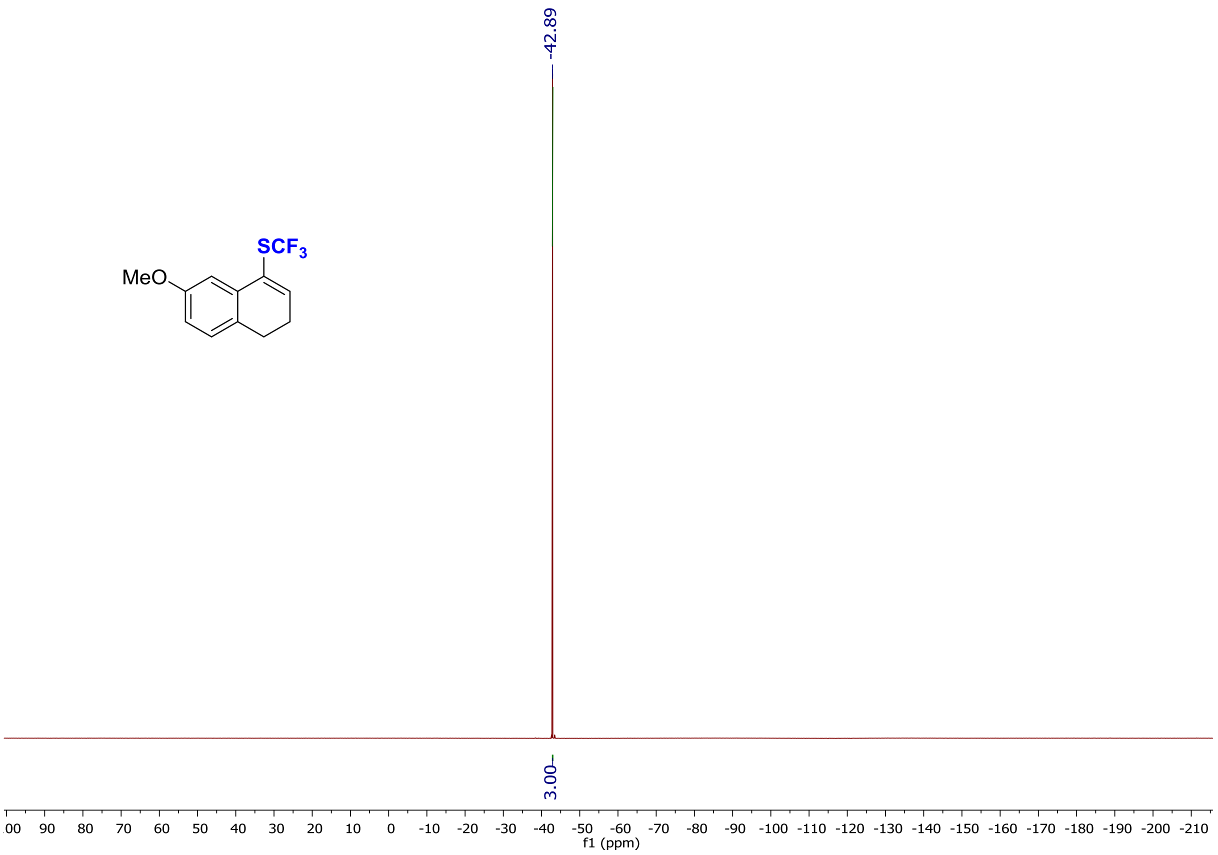
-42.89
SCF3
MeO
3.00
00 90 80 70 60 50 40 30 20 10 0 -10 -20 -30 -40 -50 -60 -70 -80 -90 -100 -110 -120 -130 -140 -150 -160 -170 -180 -190 -200 -210
f1 (ppm)

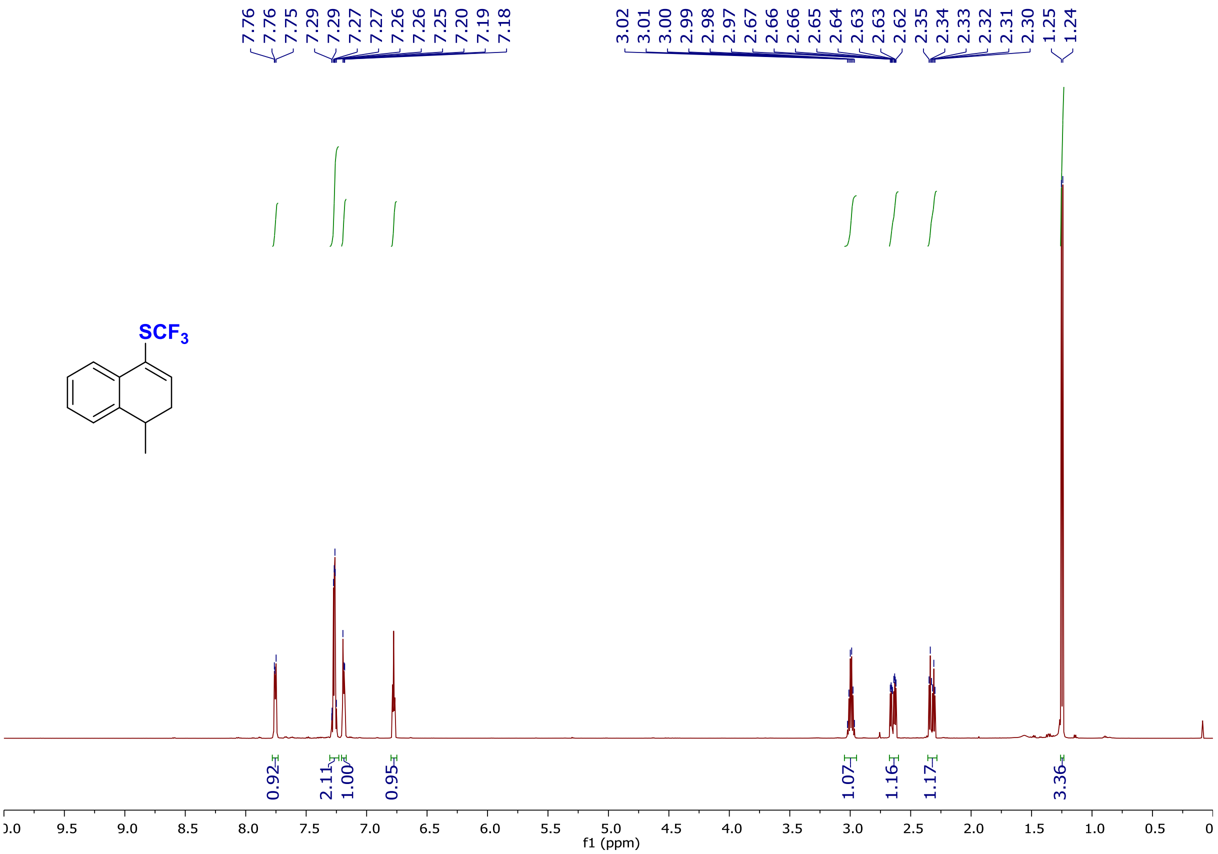

7.76
7.76
7.75
7.29
7.29
7.27
7.27
7.26
7.25
7.20
7.19
7.18
3.02
3.01
3.00
2.99
2.98
2.97
2.67
2.66
2.66
2.65
2.64
2.63
2.63
2.62
2.35
2.34
2.33
2.32
2.31
2.30
1.25
1.24
SCF3
0.92
2.11
1.00
0.95
1.07
1.16
1.17
3.36
f1 (ppm)

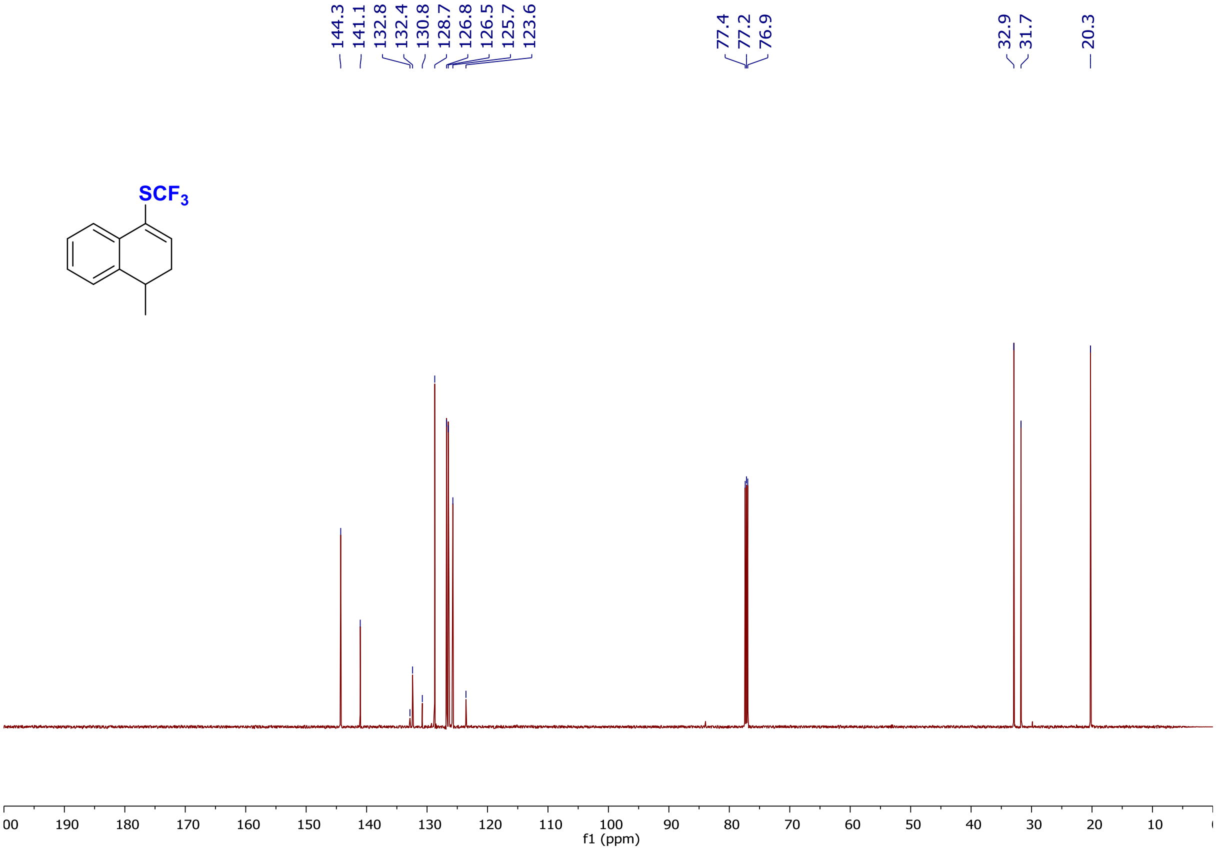

144.3
141.1
132.8
132.4
130.8
128.7
126.8
126.5
125.7
123.6
77.4
77.2
76.9
32.9
31.7
20.3
SCF3
f1 (ppm)

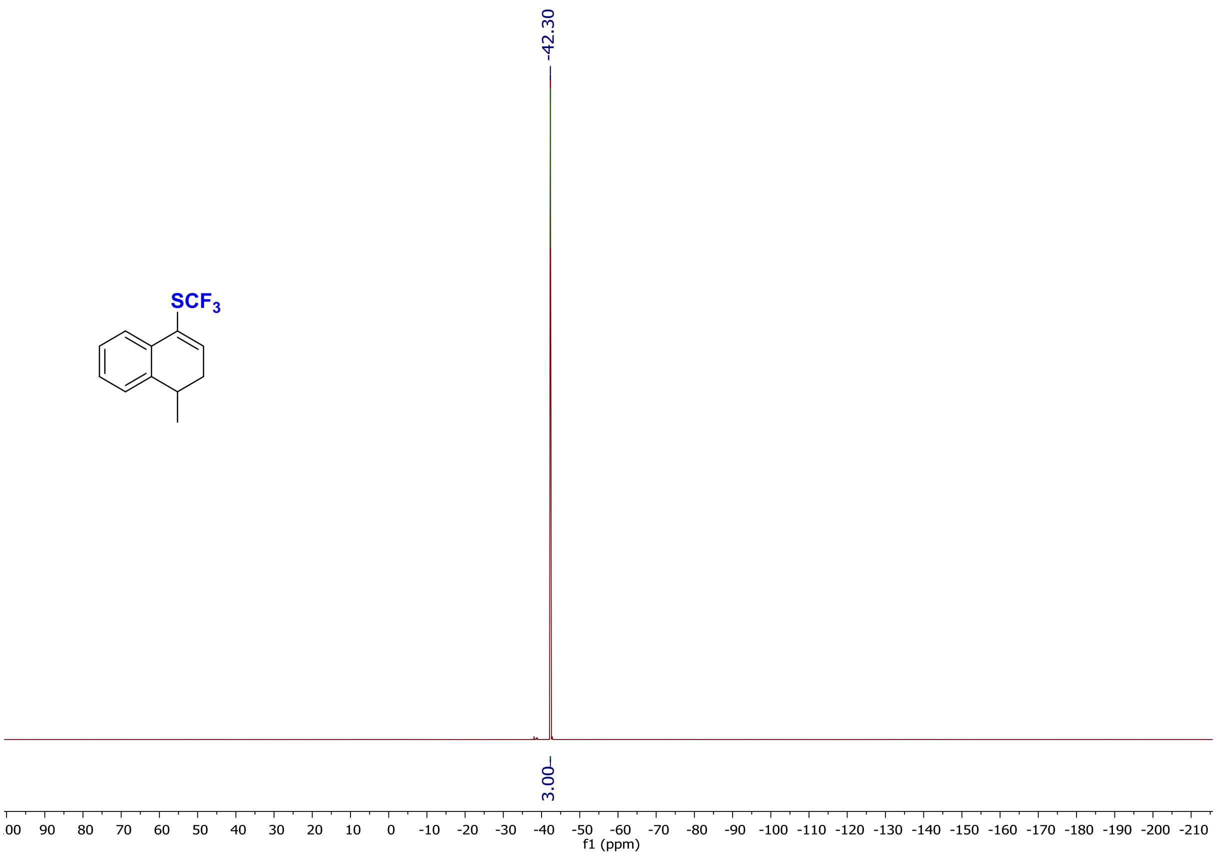
SCF3
-42.30
3.00
100 90 80 70 60 50 40 30 20 10 0 -10 -20 -30 -40 -50 -60 -70 -80 -90 -100 -110 -120 -130 -140 -150 -160 -170 -180 -190 -200 -210
f1 (ppm)

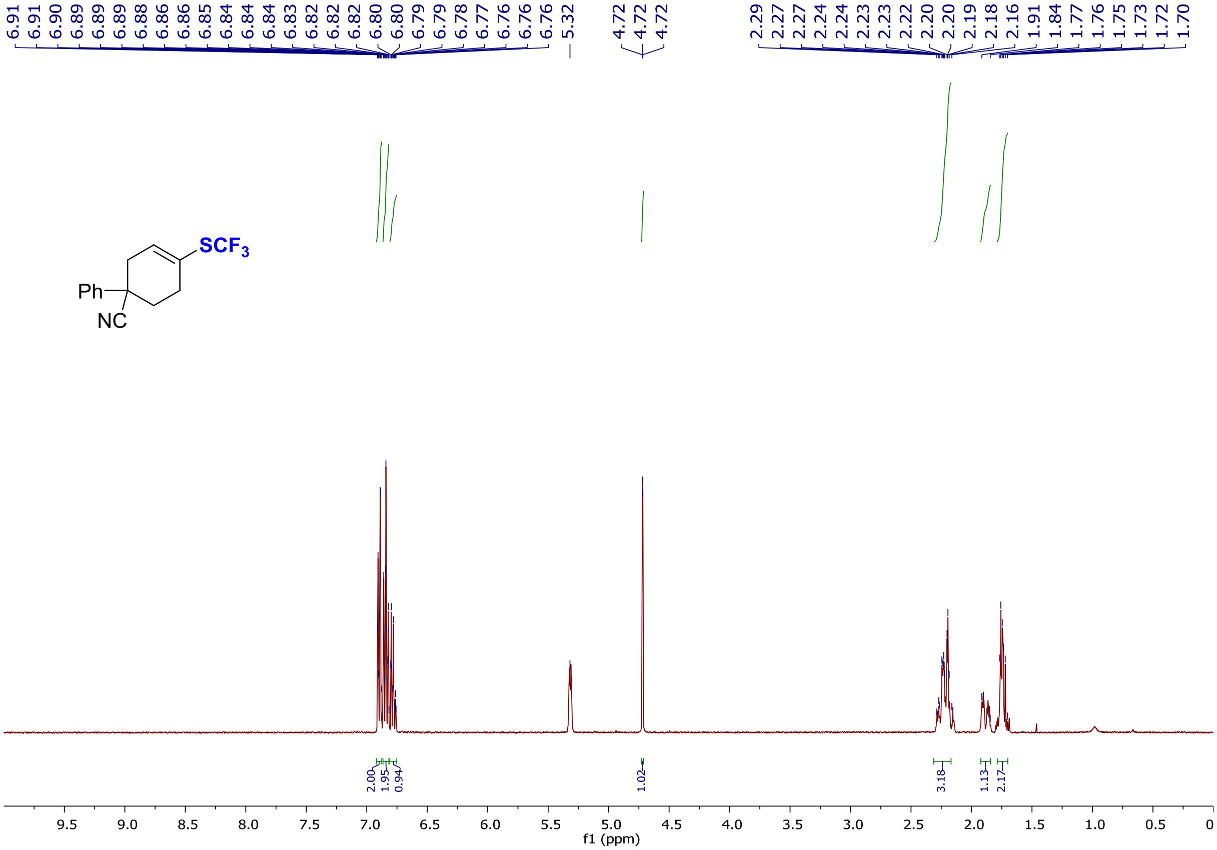
6.91 6.91 6.90 6.89 6.89 6.88 6.86 6.86 6.85 6.84 6.84 6.84 6.83 6.82 6.82 6.82 6.80 6.80 6.79 6.79 6.78 6.77 6.76 6.76 6.76 5.32 4.72 4.72 4.72 2.29 2.27 2.27 2.24 2.24 2.23 2.22 2.20 2.20 2.19 2.18 2.16 1.91 1.84 1.77 1.76 1.75 1.73 1.72 1.70
SCF3
Ph
NC
2.00
1.95
0.94
1.02
3.18
1.13
2.17
f1 (ppm)

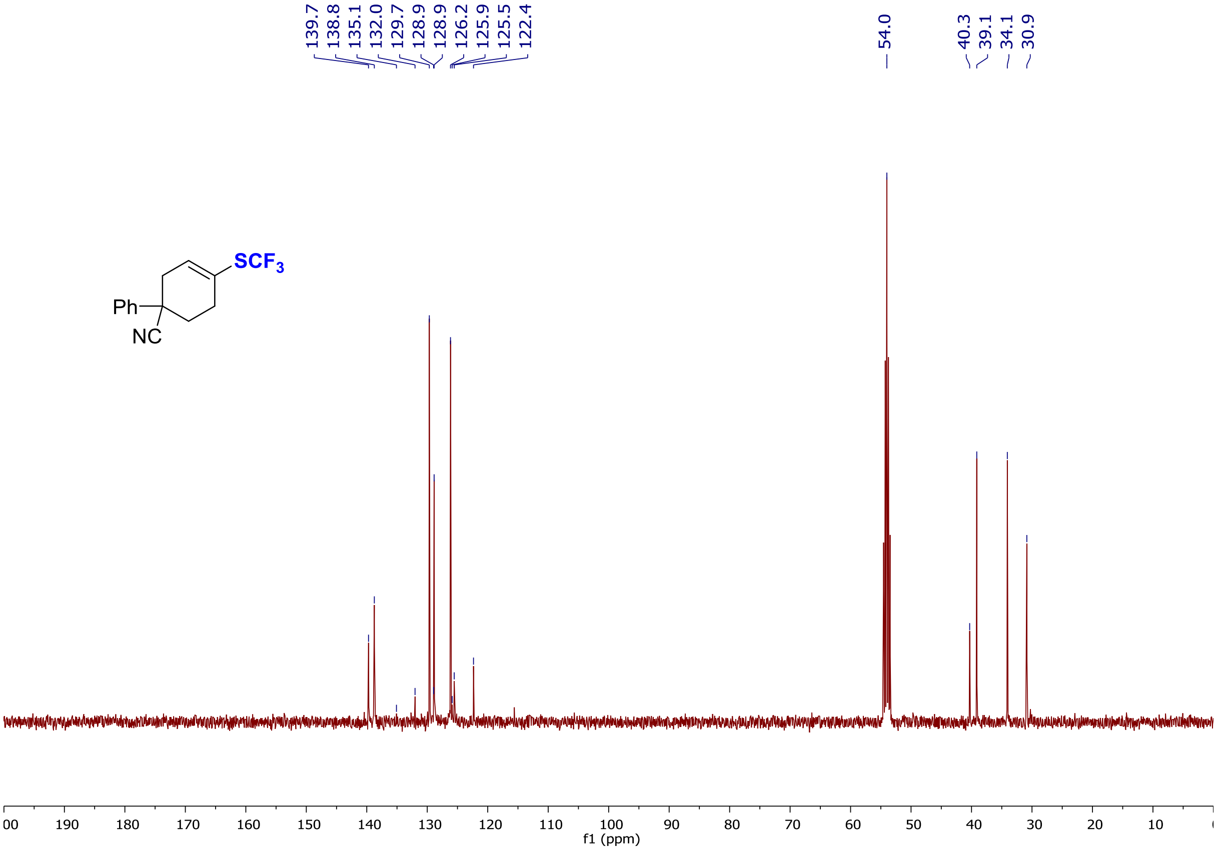
139.7 138.8 135.1 132.0 129.7 128.9 128.9 126.2 125.9 125.5 122.4
54.0
40.3 39.1 34.1 30.9
SCF3
Ph
NC
f1 (ppm)

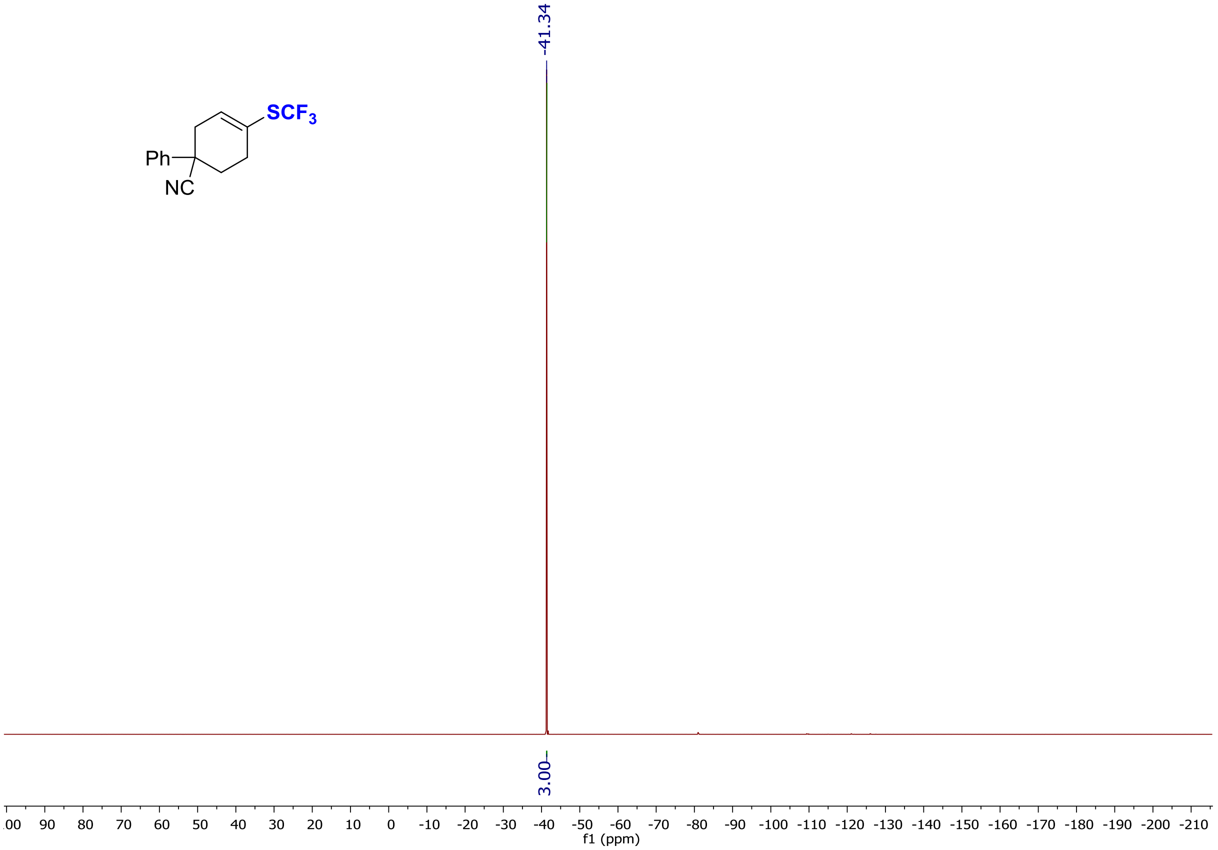
-41.34
SCF3
Ph
NC
3.00
00 90 80 70 60 50 40 30 20 10 0 -10 -20 -30 -40 -50 -60 -70 -80 -90 -100 -110 -120 -130 -140 -150 -160 -170 -180 -190 -200 -210
f1 (ppm)
